# Randomized Controlled Trials of Zhigancao Decoction Combined With Metoprolol in the Treatment of Arrhythmia: A Systematic Review and Meta-Analysis

**DOI:** 10.3389/fcvm.2022.795903

**Published:** 2022-02-23

**Authors:** Yan Yang, Fei-Lin Ge, Qian Huang, Rui Zeng, Xin-Yue Zhang, Ping Liu, Gang Luo, Si-Jin Yang, Qin Sun

**Affiliations:** ^1^Integrated Chinese and Western Medicine School, Southwest Medical University, Luzhou, China; ^2^School of Chinese Materia Medica, Beijing University of Chinese Medicine, Beijing, China; ^3^Pharmacy School, Southwest Medical University, Luzhou, China; ^4^Department of Cardiovascular Medicine, Affiliated Traditional Chinese Medicine Hospital, Southwest Medical University, Luzhou, China; ^5^National Traditional Chinese Medicine Clinical Research Base, Affiliated Traditional Chinese Medicine Hospital, Southwest Medical University, Luzhou, China; ^6^Drug Research Center of Integrated Traditional Chinese and Western Medicine, Affiliated Traditional Chinese Medicine Hospital, Southwest Medical University, Luzhou, China

**Keywords:** zhigancao decoction, metoprolol, arrhythmia, meta-analysis, randomized controlled trial

## Abstract

**Objective:**

Cardiac arrhythmia remains a major public health problem worldwide. Combinations of traditional medicine (TM) and conventional medicine (CM) have been used for arrhythmia treatment, yet the effectiveness and safety of many TM preparations can be controversial. We analyzed the safety and effectiveness of Zhigancao decoction (ZGCD) combined with metoprolol for arrhythmia treatment.

**Methods:**

Systematic searches for randomized clinical trials (RCTs) were conducted in eight databases (January 2010–September 2020) without language restrictions. According to the Cochrane system evaluation method, the overall effectiveness and safety were evaluated by meta-analysis using Review Manager software (version 5.3), and publication bias was qualitatively analyzed using STATA 12.0.

**Results:**

A total of 39 RCTs were incorporated, including 4,260 patients with arrhythmia, with 2,133 patients in the experimental group (ZGCD + metoprolol, ZGCD + BB) and 2,127 patients in the control group (metoprolol only, BB). Meta-analysis revealed that compared with BB, ZGCD + BB could significantly increase the total efficacy (OR = 4.74, 95% CI: 3.78–5.94, *P* < 0.01) and lower the incidences of arrhythmia (MD = −3.39, 95% CI: −4.09 to −2.68, *P* < 0.01). Moreover, mean HR reductions were reported in patients receiving ZGCD + BB the ZGCD + BB group (MD = −8.48, 95% CI: −10.98 to −5.97, *P* < 0.01) and a decrease in TCM symptoms were reported also (MD = −2.92, 95% CI: −3.08 to −2.76, *P* < 0.01). The incidence of adverse events was lower in patients treated with ZGCD + BB (RR = 0.36, 95% CI: 0.26–0.51, *P* < 0.01). These results appeared consistent across common arrhythmias. Nevertheless, the majority of included studies were unable to be formally assessed for bias, and funnel-plot analysis implied a moderate risk of publication bias.

**Conclusion:**

ZGCD + BB appeared to demonstrate good efficacy and fewer adverse reactions compared to BB in the treatment of arrhythmia, and this may represent a useful complementary therapy. However, our findings must be cautiously evaluated because of the small sample size and low quality of the clinic trials cited in the review. Rigorous and large-scale RCTs are warranted in the future to confirm these results.

**Systematic Review Registration:**

https://inplasy.com/inplasy-2021-10-0045/.

## Introduction

Arrhythmia refers to the abnormal origin or conduction of cardiac activation, resulting in an abnormal heart frequency and/or rhythm. Arrhythmia continues to be a common public health problem worldwide. In China, about 520,000 patients with cardiovascular disease (CVD) die from malignant arrhythmias every year (1). Drugs treating arrhythmia are mainly conventional medicine (CM), but many may cause arrhythmias and even fatal adverse events themselves; thus, their application is limited (2). Metoprolol, slows the heart rate and inhibits cardiac contractility by blocking β-adrenoceptors. Metoprolol is widely used to treat arrhythmias clinically, but has known adverse effects, such as nausea, dizziness, headache, and bradycardia (3).

In China, arrhythmia is frequently treated using a combination of CM and traditional medicine (TM). Zhigancao decoction (ZGCD) recorded in Treatise on Febrile Disease by Zhang Zhongjing in the Han dynasty have been widely used in treating palpitation and irregular pulse for thousands of years in China (4). ZGCD has a unique curative effect in arrhythmia treatment that involves a two-way benign regulatory effect. Its regulatory effects on ion channels, hemodynamics, cardiomyocyte electrophysiology, and related processes have been verified (5). Furthermore, some clinical reports have reported that ZGCD combined with metoprolol has advantages in terms of total efficacy and arrhythmia control. However, because of lack of reliable medical evidence, the effectiveness of this combination remains controversial. Hence, this systematic review and meta-analysis of published randomized clinical trials (RCTs) of ZGCD + BB in arrhythmia treatment was performed.

## Methods

### Protocol and Registration

This study protocol was registered and approved by INPLASY (Registration number INPLASY2021100045).

### Search Strategy and Selection Criteria

PubMed, Cochrane Library, Web of Science, Clinical Trials, CNKI, VIP, CBM, and Wanfang databases were searched, and the retrieval time was limited to September 2020. The Chinese keywords were xinlvshichang, zhigancaotang, fumaitang, meituoluoer, beitaleke, and suijiduizhao. Other key words were arrhythmia, arrhythmia, arrhythmias, arrhythmic, cardiac arrhythmia, prepared licorice decoction, roast glycyrrhiza decoction, roasted licorice decoction, zhigancao decoction, metoprolol, and randomized controlled trials. Logical operators were used to formulate retrieval styles using these words as keywords or free words, and manual retrieval methods were employed. If the reviewers had any questions about the studies, the corresponding author was consulted.

### Inclusion Criteria

#### Participants

The study included patients who conformed to the clinical diagnosis of arrhythmia with recurrent symptoms such as palpitation, shortness of breath, and chest tightness and confirmed clinical diagnosis using electrocardiogram, relevant laboratory findings, and imaging examinations. Patients with severe liver and kidney diseases, hematopoietic system diseases, acute infection, and grade IV heart function were excluded from the study. Only RCTs were included in this meta.

#### Intervention Measures

The intervention group was treated with ZGCD + BB, while the control group was treated with BB only. Patients in both groups were administered basic treatment for their primary disease, such as hypotension, lipid lowering, hypoglycemia, anticoagulation, and antiplatelet therapies, and other intervention measures.

#### Outcome Indicators

We have list the outcome indicators in the Table 1.

**Table 1:** The outcome indicators.

**Outcome indicators**	**Criteria**	**Data** **expression**
Total efficacy	(1) Significantly effective events: clinical symptoms and signs essentially disappeared, the number of arrhythmias decreased by more than 90%, and ECG results returned to normal	The number of (1) + (2) cases
	(2) Effective events: clinical symptoms were relieved to a certain extent, the number of arrhythmias was reduced by 50–90%, and ECG results improved	
	(3) Ineffective events: clinical symptoms did not improve or even worsened, the number of arrhythmias decreased by <50%, and there was no significant change in ECG results	
Incidences of arrhythmia	The number of arrhythmias that occurred in the experimental and control groups after treatment was recorded in 24 h	$\bar{x}$ ±
HR of Arrhythmia	Heart rate was measured after wearing the dynamic ECG for 24 h	$\bar{x}$ ± s
TCM syndrome score	According to the main symptoms, such as chest tightness, palpitation, and fatigue sweating, the scores were as follows: asymptomatic, 0; mild, 1; severe, 3; the higher the score, the more serious the condition was.	$\bar{x}$ ± s
Adverse events	Adverse events included nausea, vomiting, dizziness, headache, and bradycardia, among others	The number of cases

### Literature Exclusion Criteria

① Diagnostic method was not clear; ② Experimental and control groups were not consistent with the above intervention measures or the description of the treatment method was not provided; ③ Outcome index could not be counted; ④ Non-RCTs and non-clinical trial studies; ⑤ Duplicate publications or incomplete studies; ⑥ The full text of the publication was not available.

### Data Extraction

Data extraction was independently performed by two researchers, and the relevant studies were extracted. If differences arose during this period, they were resolved through joint discussion, with assistance from a third researcher, if necessary.

### Assessment of Trial Quality

The methodology quality evaluation of the included studies was performed using the “bias risk assessment” tool recommended by Cochrane Handbook 5.0. The quality of the included studies was evaluated in terms of the random allocation method, allocation concealment, blinding method, integrity of the results data, and selective reporting of bias of the research results. We selectively reported the bias of the research results and other aspects of quality evaluation. For each study, the above items were evaluated as “yes” (low bias), “no” (highly biased), or “unclear” (lack of relevant information or uncertainty of bias).

### Statistical Analysis

RevMan 5.3 software, provided by the Cochrane collaboration network, was used in the analysis. The two classification variables used OR as the curative effect analysis statistics, and the numerical variables used the mean difference (MD) as the curative effect analysis statistics. Each effect was expressed as a 95% confidence interval (CI). The chi-square test was used to analyze heterogeneity among the studies. When there was a high degree of statistical heterogeneity among the studies (*P* < 0.1, *I*^2^ > 50%), the random effects model was used; otherwise, the fixed effect model was used.

## Results

### Search Results

**Figure 1:**
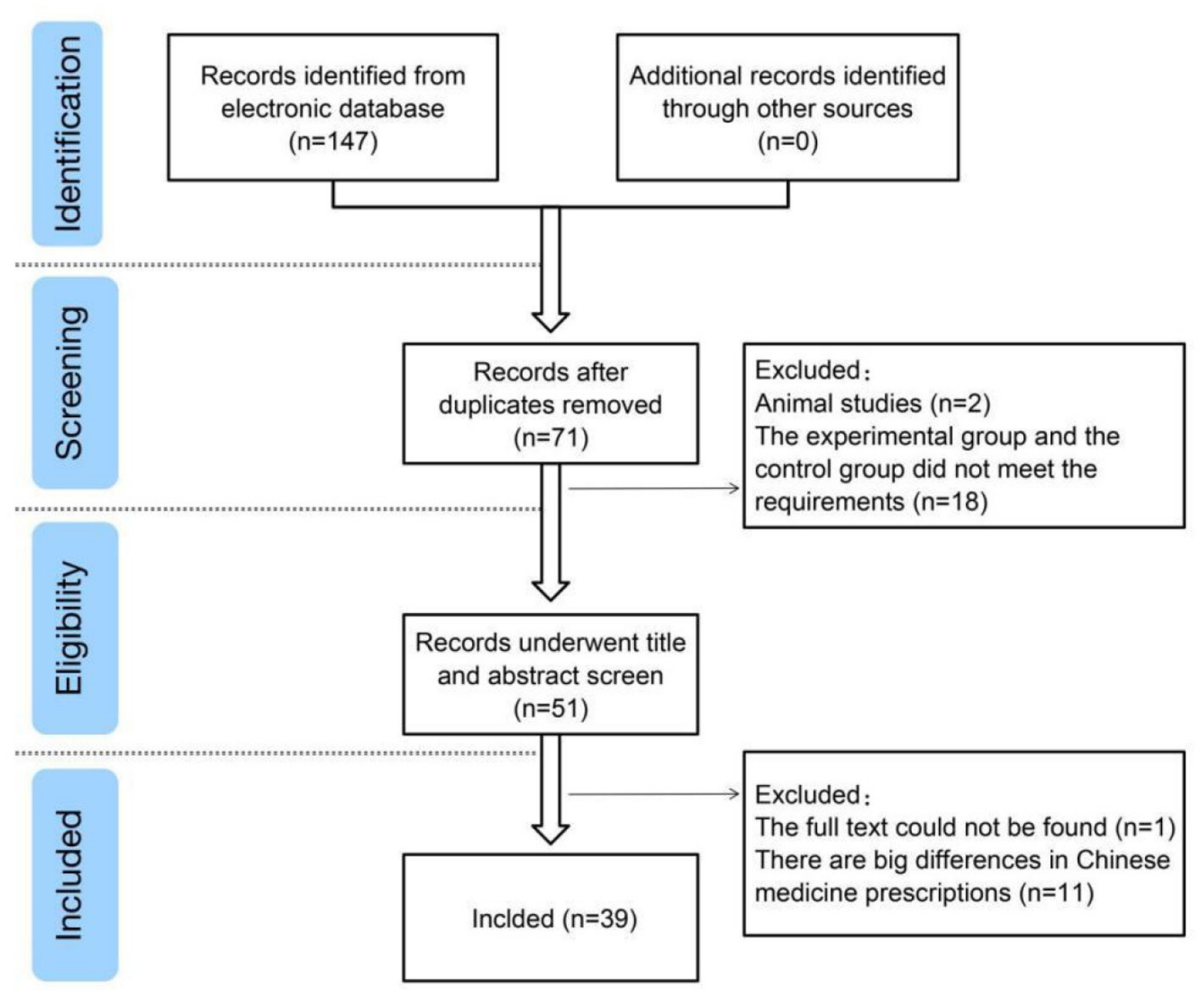
Study flow diagram.

A total of 147 studies were retrieved from the search results, and 39 studies (6–44) were included after reading abstracts and full texts. Exclusions comprised duplicate studies, case reports, reviews, retrospective studies, non-randomized controlled trials, and inconsistent trial bases (Figure 1).

### General Characteristics and Quality Evaluation of the Included Studies

**Figure 2:**
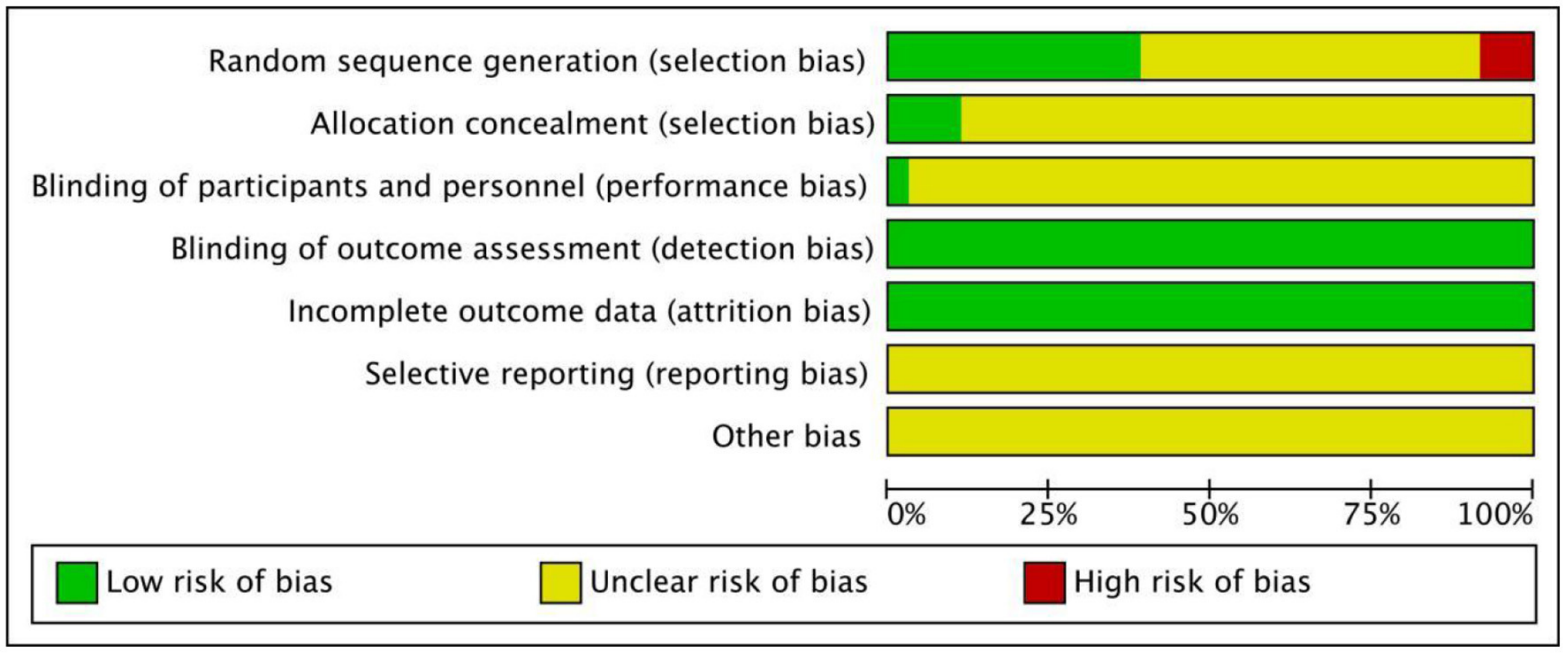
Quality evaluation of the included studies literature.

Among the 39 articles included in the general characteristics and quality evaluation of the study, all mentioned that the baseline of the experimental group was similar to that of the control group and was comparable. The terms referencing “random” were mentioned in all studies, but only 11 mentioned the random number table method, and two used allocation concealment. Because of the inconsistent dosage forms of intervention drugs, none of the studies used blind methods; selective reporting results and other sources of bias were not clear. Basic characteristics of the included studies are summarized in Table 2, and the quality of the included studies is presented in Figure 2.

**Table 2:** Basic characteristics of the included studies.

**References**	**Course of treatment**	**Control (*n*)**	**Trial (*n*)**	**Age**		**Duration**		**Dosage**		**Outcome indicators**
				**C**	**T**	**C**	**T**	**C**	**T**	
Aidufeng (6)	4 W	39	40	55.8 ± 5.1	56.4 ± 4.8	10.8 ± 1.3 M	11.1 ± 1.7 M	Metoprolol 25 mg/dose, bid	ZGCD bid+Metoprolol 25 mg/dose, bid	①②
Caoyunyan (9)	2 W	57	57	72.5 ± 8.4	73.5 ± 8.9			Metoprolol 50 mg/dose, bid	ZGCD bid+Metoprolol 50 mg/dose, bid	①③
Chenting (8)	12 W	30	30	63.7 ± 9.5	62.1 ± 8.9	3.9 ± 1.7 Y	3.7 ± 1.4 Y	Metoprolol 15 mg/dose → 50 mg/dose	ZGCD bid+Metoprolol 15 mg/dose → 50 mg/dose	①③⑤
Duanaijing (11)	2 W	50	50	62.03 ± 3.74	61.25 ± 3.87			Metoprolol 25 mg → 100 mg, bid	ZGCD bid+Metoprolol 25 mg → 100 mg, bid	①③④⑤
Guanhui (13)	1 M	40	40	67.61 ± 7.36	67.55 ± 6.78			Metoprolol 6.25 mg/dose, tid; 6.25–12.5 mg/dose, bid, no more than 300–400 mg/d	ZGCD bid + Metoprolol 6.25 mg/dose, tid; 6.25–12.5 mg/dose, bid, no more than 300–400 mg/d	①
Hedeying (14)	4 W	78	78					Metoprolol 12.5 mg, qd	ZGCD tid + Metoprolol 12.5 mg, qd	①②
Linwenzhi (18)	8 W	30	30	56.01 ± 2.11	55.38 ± 2.67	12.63 ± 2.27 M	12.59 ± 2.43 M	Metoprolol 47.5 mg, qd	ZGCD bid + Metoprolol 47.5 mg, qd	①③⑤
Liumengzhen (19)	4 W	71	71	64.3 ± 5.2	64.8 ± 5.1	5.8 ± 2.6 Y	5.7 ± 2.8 Y	Metoprolol 25 mg/dose, bid	ZGCD bid + Metoprolol 25 mg/dose, bid	①③
Suxin (26)	10 D	35	35	49.2 ± 2.3	48.5 ± 2.4			Metoprolol 25 mg/dose, bid	ZGCD bid + Metoprolol 25 mg/dose, bid	①②
Wanzhimin (33)	4 W	46	46	61.63 ± 5.14	61.56 ± 5.25	16.46 ± 6.21 M	16.52 ± 6.26 M	Metoprolol 23.75–47.5 mg, qd	ZGCD bid + Metoprolol 23.75–47.5 mg, qd	①
Yangzhongfen (36)	1 M	400	400	63.9 ± 5.3	64.1 ± 5.4			Metoprolol 23.75–47.5 mg, qd	ZGCD bid + Metoprolol 23.75–47.5 mg, qd	①③
Chenxiaolin (10)	4 W	47	50	61.3 ± 7.4	59.8 ± 6.7			Metoprolol 25–50 mg/dose, bid	ZGCD bid + Metoprolol 25–50 mg/dose, bid	①③
Fanxiuxia (12)	6 W	50	50					Metoprolol 50 mg/dose, bid	ZGCD tid + Metoprolol 50 mg/dose, bid	①②
Huangxiaoqiang (16)	1 M	48	48	59.8 ± 8.7	61.5 ± 8.2			Metoprolol 25 mg/dose, bid	ZGCD tid + Metoprolol 25 mg/dose, bid	①③
Jiangguo (17)	4 W	47	47					Metoprolol 6.25–25 mg/dose, bid	ZGCD bid + Metoprolol 6.25–25 mg/dose, bid	①②
Peiguoxian (22)	4 W	38	38	41.42 ± 5.18	38.27 ± 5.12	12.58 ± 3.62 M	13.26 ± 2.58 M	Metoprolol 23.75–47.5 mg, qd	ZGCD bid + Metoprolol 23.75–47.5 mg, qd	①
Puqinping (23)		28	28	66 ± 4.3	66 ± 4.7	2.5 ± 1.5 Y	2.5 ± 1.3 Y	Metoprolol iv, 2.5 mg, qd	ZGCD tid + Metoprolol iv, 2.5 mg, qd	①
Sunjunxiong (24)	1 M	40	40	56.1 ± 2.8	56.0 ± 3.5			Metoprolol 6.25–12.5 mg, bid, no more than 300–400 mg/d	ZGCD bid + Metoprolol 6.25–12.5 mg, bid, no more than 300–400 mg/d	①
Tangwansi (27)	10 D	30	30	60.58 ± 4.56	58.83 ± 5.18	5.85 ± 1.34 Y	5.96 ± 1.23 Y	Metoprolol 23.75 mg/dose, qd	ZGCD bid + Metoprolol 23.75 mg/dose, qd	①②③④
Wanglibin (29)	4 W	56	52					Metoprolol 11.875 mg, after 1 week 23.75 mg	ZGCD bid + Metoprolol 11.875 mg, after 1 week 23.75 mg	①⑤
Wanglin (30)	4 W	50	50	60.13 ± 7.33	59.85 ± 8.16			Metoprolol 25–50 mg, bid	ZGCD bid + Metoprolol 25–50 mg, bid	①③
Wangshanshan (31)	2 W	47	47	54.38 ± 3.37	55.1 ± 2.06	3.45 ± 2.08 Y	4.82 ± 1.06 Y	Metoprolol 25–100 mg, bid	ZGCD bid + Metoprolol 25–100 mg, bid	①
Wangzhe (32)	4 W	43	45	55.2 ± 5.6	55.0 ± 5.4	11.3 ± 2.7 M	11.1 ± 2.5 M	Metoprolol 25 mg, bid	ZGCD bid + Metoprolol 25 mg, bid	①②③
Wuyanpeng (34)	4 W	54	54	65.22 ± 3.12	65.26 ± 3.14	9.05 ± 1.51 Y	9.06 ± 1.54 Y	Metoprolol 23.75–47.5 mg, qd	ZGCD bid + Metoprolol 23.75–47.5 mg, qd	①③
Xushan (35)	4 W	75	75	49.15 ± 7.93	51.62 ± 8.09			Metoprolol 25 mg, qd	ZGCD bid + Metoprolol 25 mg, qd	①②
Zhangchunyan (37)	4 W	41	42	71.66 ± 5.43	72.17 ± 5.59	8.85 ± 2.38 M	8.92 ± 2.30 M	Metoprolol 6.25 mg, bid → 50 mg/dose, bid	ZGCD bid + Metoprolol 6.25 mg, bid → 50 mg/dose, bid	①③④
Zhangyanzhen (39)	3 M	60	60	55.0 ± 4.1	58.0 ± 3.7	5 ± 1.7 Y	5 ± 2.9 Y	Metoprolol 100 mg, qd	ZGCD bid + Metoprolol 100 mg, qd	①
Zhangyong (40)	2 W	50	50	54.9 ± 4.5	55.8 ± 2.1	4.5 ± 1.0 Y	4.2 ± 0.8 Y	Metoprolol 25–100 mg, bid	ZGCD bid + Metoprolol 25–100 mg, bid	①③
Zhaobin (41)	4 W	40	40	70.67 ± 6.34	71.64 ± 5.6s8	7.98 ± 3.45 M	8.23 ± 3.57 M	Metoprolol 6 mg, bid, after 1 week 12 mg, bid, no more than 50 mg/dose	ZGCD bid + Metoprolol 6 mg, bid, after 1 week 12 mg, bid, no more than 50 mg/dose	①③④
Meiyongxian (20)	20 D	32	32	51.8 ± 7.4	52.2 ± 7.5			Metoprolol 25–50 mg, 2–3 times/d	ZGCD bid + Metoprolol 25–50 mg, 2–3 times/d	①
Wangjigang (28)	4 W	45	45					Metoprolol 25 mg, bid	ZGCD bid + Metoprolol 25 mg, bid	①②
Zhangxiaopeng (38)	4 W	44	44					Metoprolol 25 mg, bid	ZGCD bid + Metoprolol 25 mg, bid	①⑤
Huangmianting (15)	4 W	30	30	40.73 ± 13.29	38.97 ± 10.36			Metoprolol 12.5 mg, bid	ZGCD bid + Metoprolol 12.5 mg, bid	①②③
Baiyaping (7)	4 W	44	44	58.22 ± 5.03	58.12 ± 5.23	6.32 ± 1.13 Y	6.52 ± 1.23 Y	Metoprolol 12.5–25 mg, bid, after 1 week 25–50 mg, bid	ZGCD bid + Metoprolol 12.5–25 mg, bid, after 1 week 25–50 mg, bid	①②
Oujianzhao (21)	8 W	17	20	61.15 ± 8.32	60.21 ± 7.13			Metoprolol 12.5–25 mg, bid	ZGCD tid + Metoprolol 12.5–25 mg, bid	①②
Sunyanlin (25)	6 W	45	45	67.21 ± 3.23	65.23 ± 2.98			Metoprolol 50 mg, bid	ZGCD tid + Metoprolol 50 mg, bid	①②
Zhangjilei (42)	2 W	80	80	60.48 ± 4.57	59.95 ± 5.16	5.75 ± 1.08 Y	5.94 ± 1.21 Y	Metoprolol 25 mg, bid	ZGCD bid + Metoprolol 25 mg, bid	②④
Cuixiaoting (43)	8w	40	40			2.6 ± 1.3Y	2.8 ± 1.2	Metoprolol 25 mg, bid	ZGCD tid + Metoprolol 25 mg, bid	⑤

*Control (C), metoprolol only; Trial (T), Zhigancao decoction + metoprolol; qd, quaque die; bid, bis in die; tid, ter in die;*

*Outcome indicator: ① Total Efficacy ② Incidences of arrhythmia ③ Adverse Events ④ TCM Syndrome Score ⑤ HR of Arrhythmia*.

### Meta-Analysis Results

#### Total Efficacy

**Figure 3:**
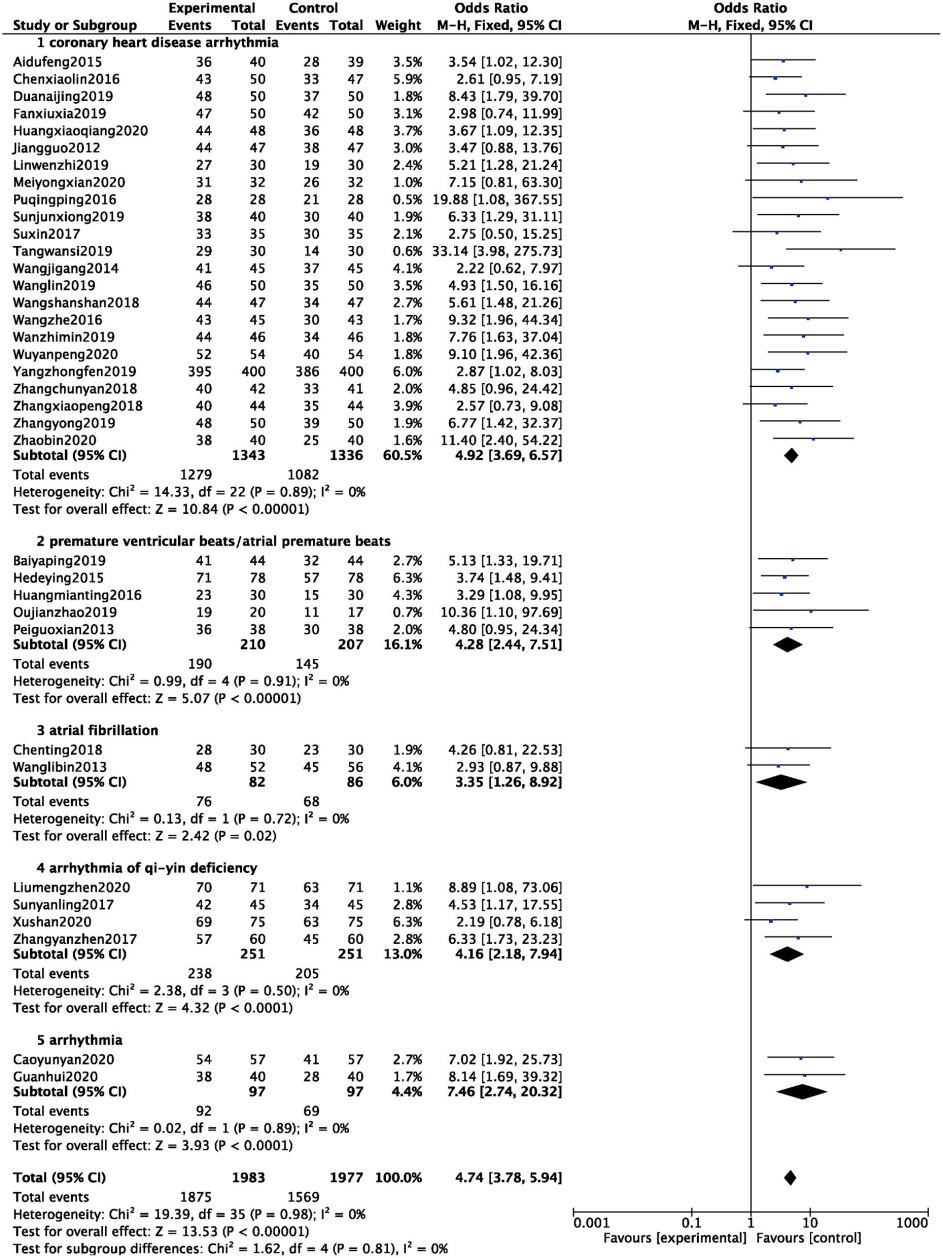
Forest map of total efficacy between experimental group and control group.

A total of 36 studies were included (6–41), and 3,960 patients were analyzed and evaluated, including BB group (*n* = 1,977) and ZGCD+ BB group (*n* = 1,983). There was no statistical heterogeneity among the studies (*I*^2^ = 0%), therefore, we used a fixed-effect model. The statistical results revealed that the total effective rate of ZGCD + BB in the treatment of arrhythmias was higher than that of BB, and the difference was statistically significant (OR = 4.74, 95% CI: 3.78–5.94, *P* < 0.01). Further subgroup analysis demonstrated that there were 23 cases of coronary heart disease arrhythmia, 5 cases of premature ventricular beats/atrial premature beats, 2 cases of atrial fibrillation, 4 cases of arrhythmia of qi-yin deficiency, and 2 cases of arrhythmia. The results demonstrated that the total efficacy of ZGCD + BB was higher for arrhythmias of different pathological types, as depicted in Figure 3.

#### Incidences of Arrhythmia

**Figure 4:**
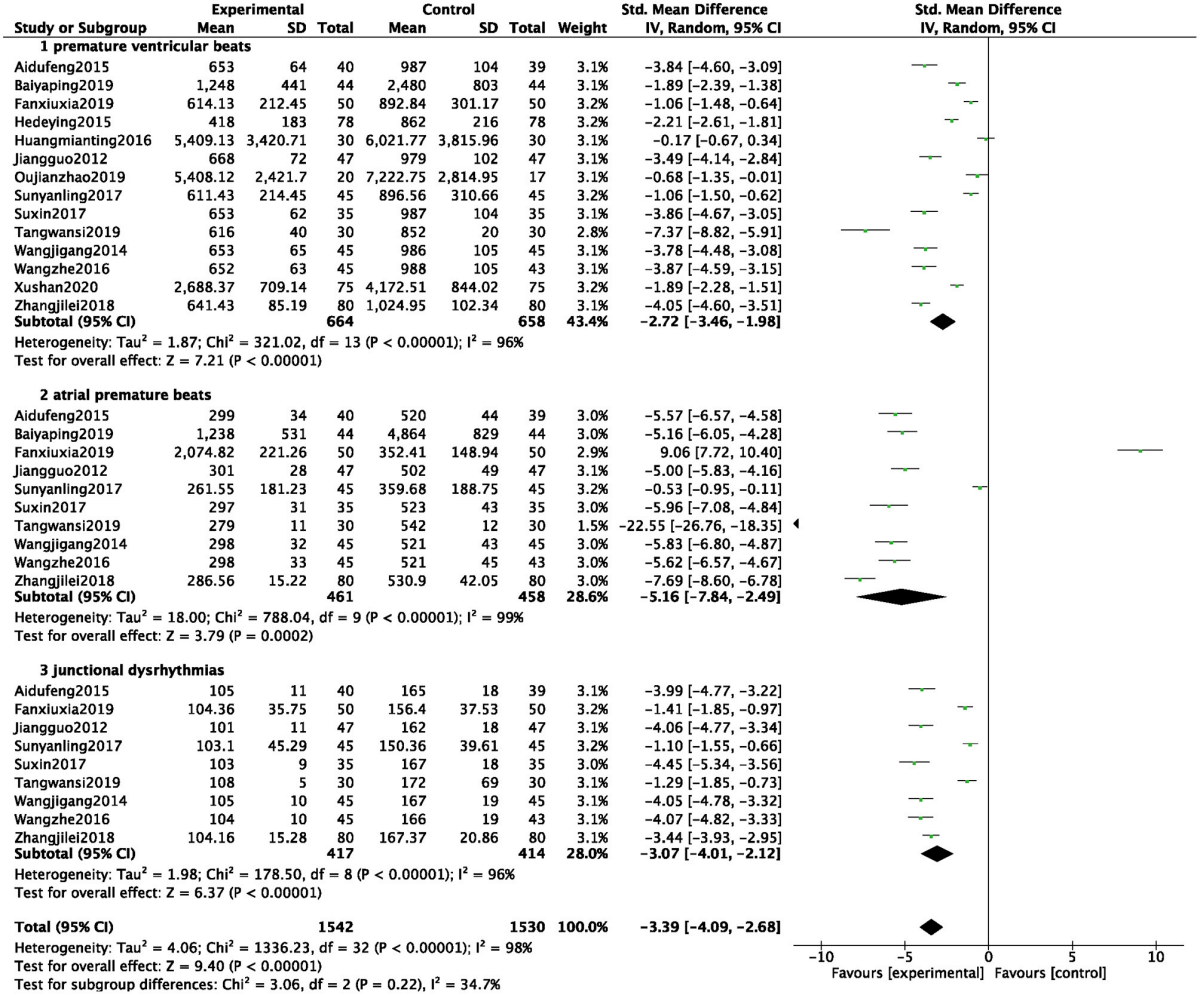
Forest map of the incidences of arrhythmia between experimental group and control group.

A total of 14 studies were included (6, 7, 12, 14, 15, 17, 21, 25–28, 32, 35, 42), and 3,072 patients were analyzed and evaluated, including BB group (*n* = 1,530) and ZGCD + BB group (*n* = 1,542). There was a large statistical heterogeneity among the studies (*I*^2^ = 98%); therefore, a random-effects model was used. The statistical results revealed that incidences of arrhythmia in the ZGCD + BB group was significantly less than that in the BB group, and the difference was statistically significant (MD = −3.39, 95% CI: −4.09 to −2.68, *P* < 0.01). Further subgroup analysis revealed that premature ventricular beats, atrial premature beats, and junctional dysrhythmias had 14, 10, and 9 studies, respectively. The results indicated that incidence of different pathological arrhythmias in the ZGCD + BB group was significantly lower than that in the BB group, as shown in Figure 4.

#### HR of Arrhythmia

**Figure 5:**
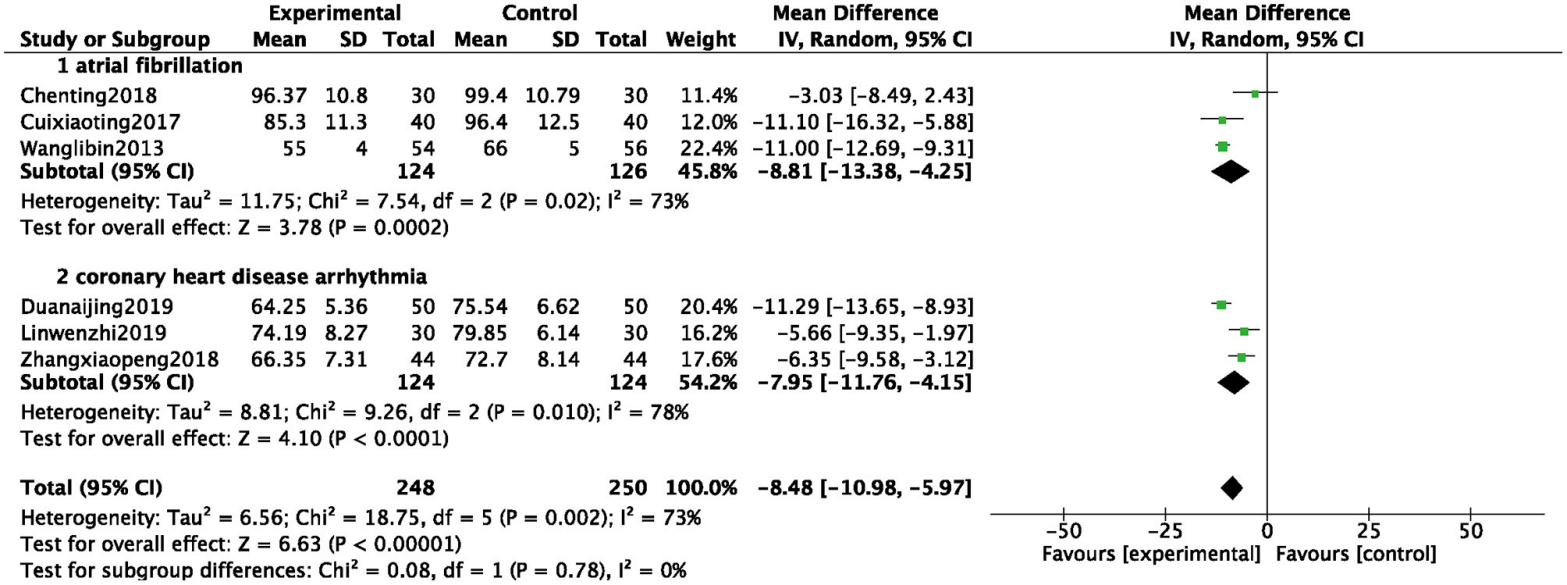
Forest map of the HR of arrhythmia between experimental group and control group.

A total of 6 studies were included (8, 11, 18, 29, 38, 43), and 498 patients were analyzed and evaluated, including BB group (*n* = 250) and ZGCD + BB group (*n* = 248). There was a large statistical heterogeneity among the studies (*I*^2^ = 73%), therefore, a random-effects model was used. The statistical results revealed that the HR in the ZGCD + BB group was significantly slowed down than that in the BB group, and the difference was statistically significant (MD = −8.48, 95% CI: −10.98 to −5.97, *P* < 0.01). Subgroup analysis demonstrated that there were 3 studies on atrial premature beats and coronary heart disease. The results proved that HR of different pathological arrhythmias in the ZGCD + BB group was significantly lower than that of the BB, as shown in Figure 5.

#### TCM Syndrome Score

**Figure 6:**
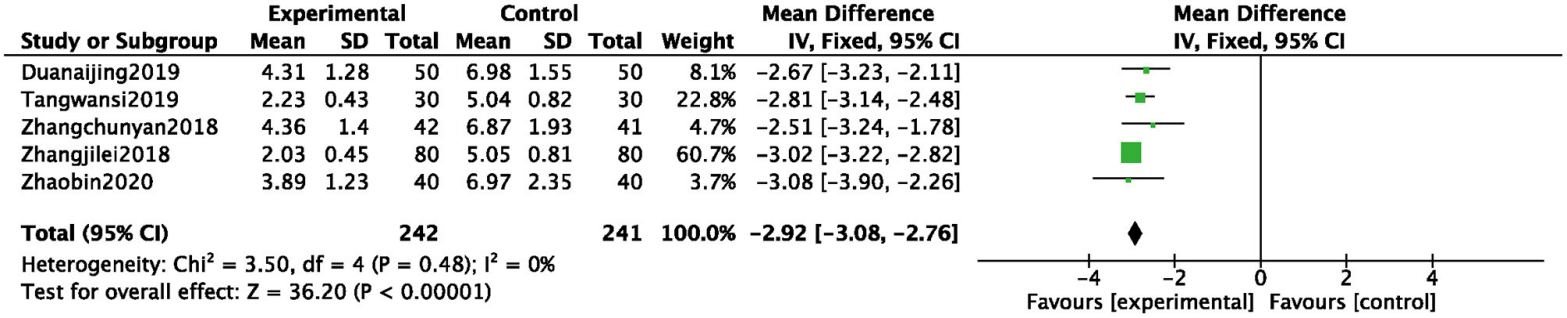
Forest map of TCM syndrome score between experimental group and control group.

A total of 5 studies (11, 27, 37, 41, 42) were included, and 483 patients were analyzed and evaluated, including BB group (*n* = 241) and an ZGCD + BB group (*n* = 242). There was statistical heterogeneity among them (*I*^2^ = 0%); therefore, a fixed-effects model was used. The statistical results revealed that the TCM syndrome score of the ZGCD + BB group was significantly lower than that of the BB group, and the difference was statistically significant (MD = −2.92, 95% CI: −3.08 to −2.76, *P* < 0.01), as shown in Figure 6.

#### Adverse Events

**Figure 7:**
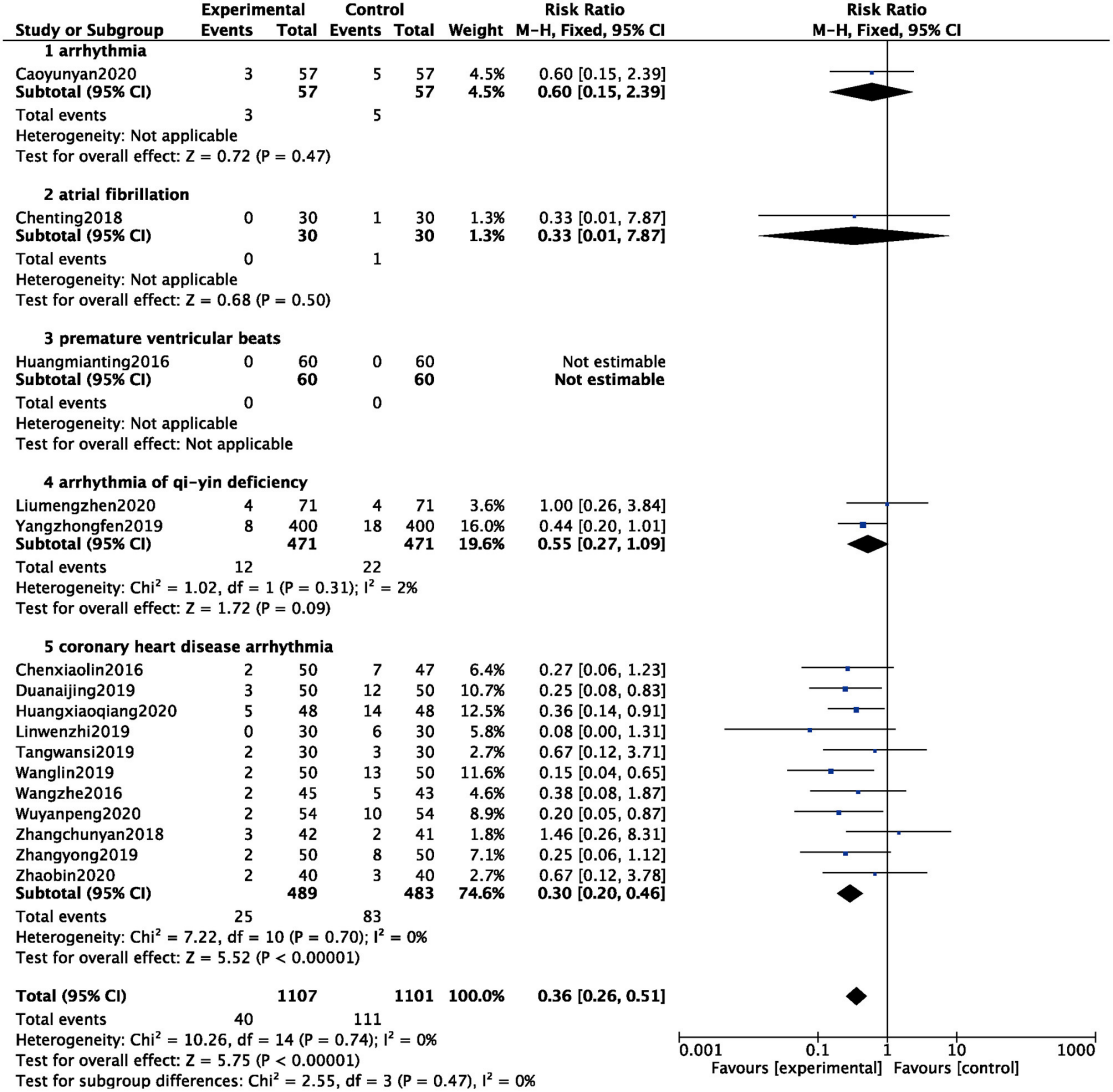
Forest map of adverse events between experimental group and control group.

A total of 16 studies counted the occurrence of adverse events (8–11, 15, 16, 18, 19, 27, 30, 32, 34, 36, 37, 40, 41), and 2,208 patients were analyzed and evaluated, including 1,101 cases in the BB group and 1,107 cases in the ZGCD + BB group. There was no statistical heterogeneity among the studies (*I*^2^ = 0%); therefore, a fixed-effect model was used. The results showed that the incidence of adverse events in the BB group was higher than that in the ZGCD + BB group, and the difference was statistically significant (RR = 0.36, 95% CI: 0.26–0.51, *P* < 0.01). Subgroup analysis revealed that arrhythmia, atrial fibrillation, premature ventricular beats, arrhythmia of qi-yin deficiency, and coronary heart disease arrhythmia had 1, 1, 1, 2, and 11 items, respectively. The results prompted that adverse events of different pathological arrhythmias in the ZGCD + BB group were significantly lower than those in the BB only group, as shown in Figure 7.

#### Publication Bias

**Figure 8:**
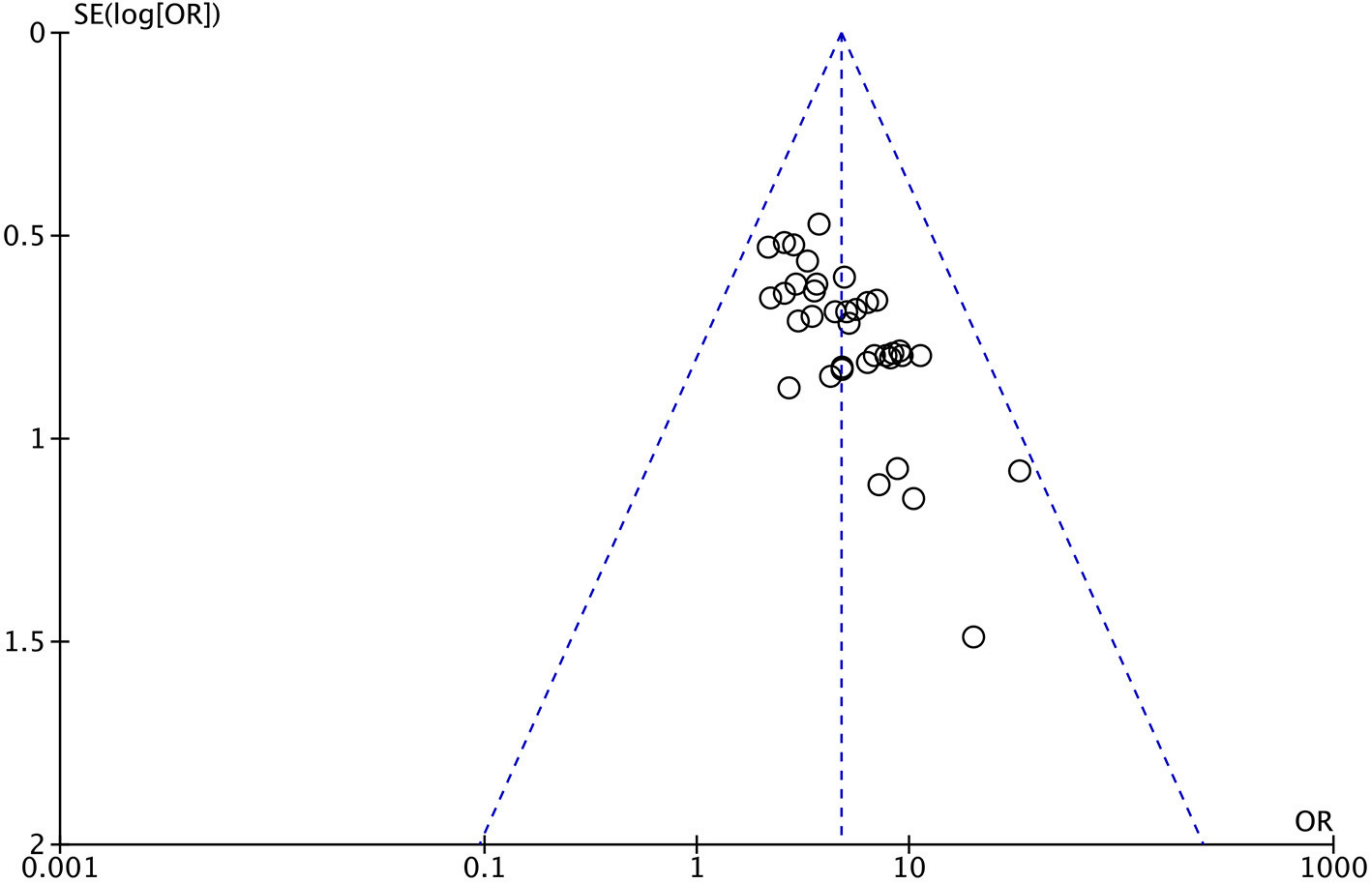
Funnel chart of total efficacy.

An inverted funnel plot was constructed for the total efficacy of the included studies, as illustrated in Figure 8. As shown, the graphic distribution of the graph is not symmetrical and does imply a bias.

## Discussion

Metoprolol is a commonly used drug for the treatment of arrhythmias in the clinic, but it has some limitations, including efficacy and safety. Therefore, increasing number of clinical practices are combining CM and TM for arrhythmia treatment, and ZGCD is the most commonly used prescription among TM. There are also some clinical reports that ZGCD combined with CM has advantages in terms of total efficacy and arrhythmia control. However, availability of high-quality research-based medical evidence remains a challenge.

This is the first metanalysis investigating ZGCD + BB may exert better effectivenes of arrhythmias. Our systematic evaluation indicated that ZGCD + BB has advantages in the treatment of arrhythmia in terms of total efficacy, arrhythmia control, HR of arrhythmia, and TCM syndrome scores. As a TM prescription, ZGCD has the characteristics of multiple components, targets, and pathways in the treatment of diseases. This may be an important reason why ZGCD supports metoprolol and enhances its effectiveness. The mechanism of ZGCD in the treatment of arrhythmia is remains unclear; some reports have shown that ZGCD can reduce HR, prolong MApD, and reduce Tp-e/QT to decrease the occurrence of ventricular arrhythmias (45). ZGCD may be related to the protection of the myocardium by effectively blocking the opening of potassium channels in hypoxic cardiomyocytes (46). Furthermore, modern pharmacological studies have demonstrated that the three main active components of ZGCD, glycyrrhizic acid, total ginsenosides of ginseng, and total saponins of *Ophiopogon japonicus*, could significantly reduce the automaticity and excitability of isolated rat atrial muscle and prolong the refractory period to inhibit arrhythmia (47). ZGCD has a unique curative effect in the treatment of arrhythmias and a two-way, benign regulatory effect. Its modified and subtracted prescriptions are effective clinically in China, demonstrating the research value and prospects of TM in the treatment of arrhythmias (48).

In addition, our results proved that the ZGCD + BB group had significantly reduced incidence of adverse events compared with the BB. As known, adverse events are an important cause of failure during pre-market clinical trials of drugs and the withdrawal of drugs after marketing. CM, including antiarrhythmic drugs, has more adverse events, which seriously affect drug effectiveness and cause secondary injuries to patients. However, as a TM prescription, ZGCD not only has a good effect on arrhythmias, but is also safe. No obvious ZGCD-related adverse events have been observed in publicly published research reports or in the adverse drug reaction notifications of the China National Medical Products Administration. In addition, it can significantly reduce adverse events, such as nausea, vomiting, constipation, dizziness, headache, and bradycardia. In short, ZGCD combined with metoprolol appears safe and effective, and is worthy of further consideration for clinical utility.

This study followed the principles of evidence-based medicine. We carefully evaluated the quality of each study, used subgroup analysis to explore heterogeneity, detected publication bias, and discussed possible influencing factors to provide reliable evidence for clinical practice and decision-making. Our meta-analysis is unprecedented and innovative and includes many studies and comprehensive evaluation indicators. However, the following limitations are also present: ① The studies are published in the Chinese language, exclusively, with low quality, and may be biased; ② All studies refer to terms referencing the word “random, ” but only 11 studies referenced the random number table method; ③ Because of the inconsistent dosage forms of intervention drugs, none of the studies used blind methods; ④ Differences between individual patients may also lead to bias, such as gender, age, underlying diseases, and treatment of underlying diseases; ⑤ The plot is not symmetrical and does imply a bias. It is likelihood of bias in both patient selection, and non-blinded nature of an intervention; ⑥ In different studies, the compatibility dose composition of TM compounds is different, which may cause bias.

## Conclusion

ZGCD + BB appeared to demonstrate good efficacy and fewer adverse reactions compared to BB in the treatment of arrhythmia. The addition of this TM may represent a useful complementary therapy to standard approaches. However, our findings must be cautiously evaluated because of the small sample size and the low quality of the citied clinical trials that lack strict clinical design. High-quality, suitably powered and double-blinded RCTs are required to confirm these findings. Further mechanistic investigations on the underlying biology of ZGCD are warranted and may yield important biological insights into arrhythmogenesis.

## Data Availability Statement

The original contributions presented in the study are included in the article/Supplementary Material, further inquiries can be directed to the corresponding authors.

## Author Contributions

YY contributed to carry out the protocol, drafted the manuscript, and carried out the acquisition of data and analysis. F-LG, QH, RZ, and X-YZ participated in the data extracting. GL and PL were in charge of quality control. QS and S-JY designed and managed this protocol. All authors contributed to the article and approved the submitted version.

## Funding

This work was supported by State Administration of Traditional Chinese Medicine Project (2019XZZX-XXG006), Sichuan Provincial Department of Science and Technology Project (2021YFH0150), and Sichuan Provincial Administration of Traditional Chinese Medicine Project (2021MS107).

## Conflict of Interest

The authors declare that the research was conducted in the absence of any commercial or financial relationships that could be construed as a potential conflict of interest.

## Publisher's Note

All claims expressed in this article are solely those of the authors and do not necessarily represent those of their affiliated organizations, or those of the publisher, the editors and the reviewers. Any product that may be evaluated in this article, or claim that may be made by its manufacturer, is not guaranteed or endorsed by the publisher.
