# Supplementary material for: Randomized Controlled Trials of Zhigancao Decoction Combined With Metoprolol in the Treatment of Arrhythmia: A Systematic Review and Meta-Analysis
Source: Front Cardiovasc Med. 2022 Feb 23;9:795903. doi: 10.3389/fcvm.2022.795903 (PMC8904740; doi:10.3389/fcvm.2022.795903)
Supplement: Supplementary file 1 [file Data_Sheet_1.PDF]

炙甘草汤联合美托洛尔治疗冠状动脉粥样硬化性心脏病心律失常

艾都凤
(武汉市中医医院汉阳分院内科,武汉 430051)

摘要:目的 探究炙甘草汤与美托洛尔联合治疗冠状动脉粥样硬化性心脏病心律失常的效果。**方法** 选取 79 例冠状动脉粥样硬化性心脏病心律失常患者作为研究对象,并按随机数字表法分为观察组和对照组,给予观察组(40 例)炙甘草汤联合美托洛尔治疗,对照组(39 例)则单纯使用美托洛尔治疗,比较 2 组的治疗效果。**结果** 观察组的治疗总有效率为 90.0%,对照组为 71.8%,观察组的治疗总有效率明显高于对照组,$P<0.05$。观察组心律失常改善情况优于对照组,$P<0.05$。**结论** 冠状动脉粥样硬化性心脏病心律失常采用炙甘草汤与美托洛尔联合治疗的方法,临床效果显著,能有效改善心律失常情况。

关键词: 炙甘草汤;美托洛尔;冠心病;心律失常
中图分类号: R541.4 **文献标志码:** A **文章编号:** 1009-8194(2015)02-0034-02
DOI: 10.13764/j.cnki.lcsy.2015.02.014

冠状动脉粥样硬化性心脏病心律失常,又叫冠心病心律失常,是冠心病中比较常见的并发症之一,也是临床常见的心血管病症。主要是由心脏传导系统和起搏病变引发病理性异常起搏的一种症状[1]。目前,临床常用于治疗该病症的药物有炙甘草汤、美托洛尔等。若采取中西药结合治疗的方式,则能实现优势互补,取得良好的临床效果。武汉市中医医院汉阳分院采用炙甘草汤与美托洛尔联合治疗冠心病心律失常取得了较为满意的效果,报告如下。

1 资料和方法

1.1 一般资料

选取 2002 年 9 月至 2013 年 9 月本院收治的 79 例冠状动脉粥样硬化性心脏病心律失常患者作为研究对象。所有患者均符合临床诊断标准,按随机数字表法分为观察组和对照组。

观察组 40 例,女性 17 例、男性 23 例;年龄 40~75 岁,平均年龄(56.4±4.8)岁;病程 6 个月~2 年,平均病程(11.1±1.7)个月;12 例房室交界性期前收缩、18 例室性期前收缩、10 例房性期前收缩。

30 对照组 39 例,女性 16 例、男性 23 例;年龄 41~73 岁,平均年龄(55.8±5.1)岁;病程 7 个月~2 年,平均病程(10.8±1.3)个月;13 例房室交界性期前收缩、17 例室性期前收缩、9 例房性期前收缩。

2 组患者的性别、年龄、病程、类型等一般资料比较差异无统计学意义,$P>0.05$,具有可比性。

1.2 治疗方法

所有患者先给予常规药物治疗,包括服用降压药物、钙离子拮抗剂、硝酸酯类药物、抗凝药物等,而不再使用其他抗心律失常药物[2]。然后给予观察组炙甘草汤联合美托洛尔治疗,其中,炙甘草汤药剂方如下:炙甘草 15 g,大枣 10 g,人参 6 g,阿胶(炖化)9 g,桂枝 9 g,麻子仁 10 g,生地黄 10 g,麦冬 10 g,生姜 6 g;用清水煎服,每日 1 剂。另外,联合美托洛尔治疗,25 mg·次$^{-1}$,2 次·d^{-1},以 4 周作为 1 个治疗疗程。对照组则单独使用美托洛尔治疗,服用方法和疗程与观察组相同。

观察比较 2 组的临床治疗效果以及治疗前后心律失常的改善情况。

1.3 疗效评定

显效:患者的临床症状,如胸闷心悸、气短、乏力等消失,进行 ECG、Holter 检查显示基本恢复正常,发作次数减少 90%以上。

有效:患者的临床症状有所减轻,进行 ECG、Holter 检查显示有明显改善,发作次数减少 50%~90%。

无效:患者的临床症状无任何改善,进行 ECG、Holter 检查显示无改变,甚至加重,发作次数减少低于 50%。

收稿日期: 2014-08-26

总有效率=(显效+有效)例数/总例数×100%。

1.4 统计学方法

所有数据的统计分析使用 SPSS13.0 软件，用 $\bar{x}\pm s$ 表示计量资料，并用 t 检验，用 χ^2 检验计数资料，$P<0.05$ 表示差异有统计学意义。

2 结果

2.1 心律失常改善情况

2 组经治疗后，心律失常均得到有效改善，且观察组心律失常改善情况优于对照组，差异具有统计学意义($P<0.05$)，见表 1。

表 1 2 组心律失常改善情况比较 $\bar{x}\pm s$，次·24 h^{-1}

组别	n	房室交界期前收缩		房性期前收缩		室性期前收缩	
		治疗前	治疗后	治疗前	治疗后	治疗前	治疗后
观察组	40	258±25	105±11	1 008±88	299±34	1 868±155	653±64
对照组	39	268±35	165±18	1 009±90	520±44	1 870±164	987±104
t		−1.370	19.369	−1.111	27.910	−0.146	13.332
P		0.175	0.000 1	0.912 0	0.000 0	0.881 0	0.000 2

2.2 临床疗效比较

观察组的临床治疗总有效率为 90.0%，对照组为 71.8%，观察组的治疗总有效率明显高于对照组，且差异具有统计学意义($P<0.05$)，见表 2。

表 2 2 组临床疗效比较 例

组别	n	显效	有效	无效	总有效率/%
观察组	40	16	20	4	90.0*
对照组	39	12	16	11	71.8

* $P<0.05$($\chi^2=4.25$)与对照组比较。

3 讨论

心律失常是心血管常见症状，也是冠心病的主要并发症之一。随着我国人口老龄化进程的加快，以及人们生活方式的改变，患冠心病的患者数量呈逐年上升趋势，因此，心律失常患者也随之增加。患有冠心病心律失常轻则影响生活质量，引起心悸、乏力等症状，重则危机生命安全，引发猝死[3]。对冠心病心律失常进行及时有效的治疗是关键。但选择何种药物才能有效治疗冠心病心律失常是临床研究的重点。

当前，临床很多采用炙甘草汤与美托洛尔联合治疗冠心病心律失常，并收效良好，得到广大医学者的普遍认可。其中，炙甘草汤具有益气复脉、滋阴补血等效果，从而有利于提高心肌的耐缺氧能力，增加心脏冠状动脉的血流量，因而能促进心脏节律恢复正常[4]。而美托洛尔能减慢异位起搏点的频率和兴奋传导的速度，从而达到抗心率失常的功效。

本研究中，观察组应用炙甘草汤与美托洛尔联合治疗冠心病心律失常效果满意，在改善心律失常方面优于单独用药的对照组，且临床疗效也明显高于对照组，比较 2 组 2 项指标均差异较大，$P<0.05$，具有统计学意义。可见，采用炙甘草汤与美托洛尔联合治疗冠状动脉粥样硬化性心脏病心律失常，临床效果显著，能有效改善心律失常情况。

参考文献：

[1] 姜果. 炙甘草汤联合美托洛尔治疗冠心病心律失常疗效观察[J]. 中国医药指南，2012，10(1)：214-215.

[2] 谭瑜. 心悸患者应用炙甘草汤加味治疗的临床效果观察[J]. 中国临床研究，2014，6(14)：119-120.

[3] 张红霞. 炙甘草汤治疗冠心病心律失常临床观察[J]. 光明中医，2011，26(4)：743-744.

[4] 马媛，朱创洲. 炙甘草汤加减治疗冠心病合并心律失常 60 例[J]. 现代中医药，2011，31(2)：4-5.

(责任编辑：刘大仁)

中西医结合治疗心律失常的临床疗效分析与探讨

白亚萍

（宝鸡市第三人民医院，陕西 宝鸡 721000）

【摘 要】目的：探讨采用中西医结合治疗心律失常（期前收缩、心房纤颤、心房扑动）的临床疗效。方法：将我院2016年6月至2018年6月收治的心律失常患者88例随机分为治疗组和对照组，各44例。对照组采用倍他乐克治疗；治疗组在对照组基础上配合炙甘草汤。比较两组患者的临床疗效及24小时心律失常发生次数。结果：治疗组临床总有效率（93.18%）显著高于对照组（72.73%），差异有统计学意义（P<0.05）；治疗后，治疗组24小时动态心电图检查心律失常发作次数明显少于对照组，差异有统计学意义（P<0.05），24小时心律失常症状发作次数明显减少。结论：对于各种心律失常患者，临床应用倍他乐克片配合炙甘草汤治疗，临床疗效显著，是一种较为可靠的临床治疗方案。

【关键词】倍他乐克片；炙甘草汤；心律失常

【中图分类号】R54 【文献标识码】A 【文章编号】1005-0019（2019）24-143-01

心律失常指心脏活动的起源和（或）传导障碍导致心脏搏动的频率和（或）节律异常。心律失常是冠心病的常见并发症，临床上常见室性早搏、房性早搏等，多于劳累、情绪激动时诱发，病变进展严重时会危及生命[1-2]。心电图检查是重要的诊断依据。临床治疗需根据患者病因进行药物治疗或非药物治疗。因此，探讨冠心病合并心律失常的有效方法有重要的临床意义。

1 资料与方法

1.1 一般资料

将我院2016年6月至2018年6月临床治疗的88例心律失常患者作为研究对象，

纳入标准：临床反复出现心悸、气短，胸闷等，影响患者日常生活；行24小时动态心电图提示室性早搏总数或房性早搏总数大于10000个；且心功能在III级以上，没有肺部疾病者；患者及家属均知情同意。

将上述患者随机分为治疗组、对照组，每组44例。治疗组中，男性18例，女性26例；年龄41-78岁，平均年龄（58.12±5.23）岁；病程0.5-9年，平均病程（6.52±1.23）年。对照组中男性20例，女性24例；年龄42-79岁，平均年龄（58.22±5.03）岁；病程1-11年，平均病程（6.32±1.13）年。两组患者的一般资料比较，差异无统计学意义（P<0.05），具有可比性。本研究经医学伦理委员会批准。

1.2 治疗方法

对照组患者给予倍他乐克片治疗。

具体方法：口服倍他乐克片（厂家：阿斯利康制药有限公司；批准文号：国药准字H32025392），初始剂量：12.5-25mg/，2次/日；于1周后加至治疗剂量25-50mg/次，2次/日；持续服用4周。

治疗组在对照组的基础上配合炙甘草汤，方剂成分：炙甘草12g，生姜9g，人参6g，生地50g，桂枝9g，阿胶6g，麦冬10g，麻仁10g，大枣10枚。将上述中药用水煎服，1剂/日，早晚分服，连服4周。

1.3 观察指标及疗效判定标准

（1）参照《常见心律失常病因、严重程度及疗效制定标准》[3]，对两组患者的临床疗效进行观察比较。

显效：临床症状及体征基本消失，心律失常发生次数减少>90%，且心电图检查恢复正常；

有效：临床症状有一定程度缓解，心律失常发生次数减少50-90%，且心电图有所改善；

无效：临床症状无任何改善，甚至出现恶化，心律失常发生次数减少<50%。将显效率、有效率纳入总有效率。

（2）比较两组患者24小时动态心电图房性早搏、室性早搏发生次数。

1.4 统计学方法

此次研究数据应用SPSS19.0统计学软件进行处理，计量（临床疗效）、计数资料（心律失常症状发生次数）表示为（均数±标准差）（$\bar{x}\pm s$）、（%），以t、χ^2检验，P＜0.05表示差异有统计学意义。

2 结果

2.1 两组患者的临床疗效比较

治疗后，治疗组患者总有效率为93.17%，明显高于对照组的72.71%，差异有统计学意义（P<0.05）。详见表1。

表1 两组患者的临床疗效比较（n=44，n/%）

组别	显效	有效	无效	总有效率
治疗组	25/56.82	16/36.36	3/6.82	93.18
对照组	18/40.91	14/31.82	12/27.27	72.73
χ^2	-	-	-	6.510
P	-	-	-	0.011

2.2 两组患者治疗前后心律失常症状发生次数比较

治疗前，两组患者24小时房性早搏、室性早搏发生次数无明显差异（P>0.05）；治疗后，两组心律失常症状发生次数明显减少（P<0.05），且治疗组心律失常症状发作次数明显少于对照组，差异有统计学意义（P<0.05）。详见表2。

表2 两组患者治疗前后心律失常症状发生次数比较（n=44，$\bar{x}\pm s$）

组别	房性早搏（次）		室性早搏（次）	
	治疗前	治疗后	治疗前	治疗后
治疗组	10340±2873	1238±531	6380±2002	1248±441
对照组	10842±3029	4864±829	6420±2315	2480±803
t	0.7976	24.4313	0.0935	8.920
P	0.4273	0.000	0.9258	0.000

注：组内治疗前后比较，aP<0.05.

3 讨论

冠心病是造成人类死亡的主要疾病之一，该类患者体内脂质代谢出现异常，动脉粥样硬化致动脉管腔狭窄，机体血流不畅，心肌细胞缺血缺氧，引发心律失常、心绞痛、心肌梗塞等。中老年患者常常有高血压、糖尿病、吸烟、肥胖等冠心病危险因素，为冠心病的主要发病人群，该病的典型症状包括心悸、乏力、胸闷、呼吸困难[4]。相关研究指出[5]，冠心病、心肌炎、电解质紊乱、内分泌失调、植物神经功能紊乱等均可诱发心律失常。冠心病心律失常包括急性再灌注心律失常和慢性心律失常，各种心律失常均可引起冠状动脉血流量降低，尤其对于有冠心病患者，各种心律失常都可以诱发或加重心肌缺血，主要表现为心绞痛、急性心力衰竭、急性心肌梗死等。有关学者认为[6]，心律失常是冠心病病情不稳定的临床表现之一，同样是造成冠心病患者病情急剧恶化或死亡的危险因素。中医将该病归于“心悸”“怔忡”“胸痹”等范畴。历代名医认为，心悸为本虚标实之证，其本为心气不足，心阳虚衰，阴血亏虚。本研究中的炙甘草汤，出自《伤寒论》，具有益气滋阴，通阳复脉之功，主治阴血阳气虚弱，心脉失养证，方中炙甘草、人参、大枣益心气、补脾气，以资气血生化之源；阿胶、麦冬、麻仁滋心阴、养心血、充血脉，共为臣药；佐以桂枝、生姜辛行温通，以温心阳，通血脉，诸厚味滋腻之品得姜、桂则滋而不腻，用法中加清酒煎服，以清酒辛热之功温通血脉，以行药力，是为使药；诸药合用，滋而不腻，温而不燥，使气血充足，阴阳调和，则心动悸、脉结代皆得其平。若患者失眠多梦，可加酸枣仁、柏子仁以增强养心安神定悸之力，或加龙齿、磁石重镇安神；偏于心气不足者，重用炙甘草、人参；偏于阴血虚者，重用生地、麦门冬；心阳偏虚者，易桂枝为肉桂，加附子以增强温心阳之力；阴虚而内热较盛者，易人参为南沙参，并减去桂、姜、枣、酒，酌加知母、黄柏，则滋阴液降虚火之力更强[7-8]。

西药倍他乐克片是选择性β受体阻滞剂，具有降低交感神经兴奋性、稳定心率、减少心输出量，改善心肌损伤、降低收缩压及保护心脏功能的作用，抗心律失常效果良好，且能预防猝死[9]。其与炙甘草汤联合应用治疗冠心病心律失常，可显著改善心律失常症状的发作次数。本研究结果显示，治疗组患者总有效率高于对照组，差异有统计学意义（P<0.05），治疗后两组心律失常发作次数明显减少，治疗组24小时房性早搏、室性早搏发生次数均少于对照组，差异有统计学意义（P<0.05），表明倍他乐克联合炙甘草汤治疗心律失常临床疗效显著，可有效改善心律失常患者的症状，减少发作次数。

综上所述，对于各种心律失常患者临床应用倍他乐克片配合炙甘草汤治疗，临床疗效显著，降低心律失常症状发生次数，是一种较为可靠的临床治疗方案，值得推广。

参考文献

[1] 李伟.对室性心律失常患者实施中西医结合治疗的效果分析[J].中国卫生产业，2014(20):184-185.

[2] 黄潭荣.中西医结合治疗冠心病心律失常临床分析[J].深圳中西医结合杂志，2016(6):27-28.

[3] 马涛，董一兰.中西医结合治疗原发性高血压合并快速性心律失常的临床观察探讨[J].中西医结合心血管病电子杂志，2019,7(06):171.

[4] 迟玉双.浅析中医治疗冠心病心律失常的疗效及安全性[J].中国卫生标准管理，2015(24):139-140.

[5] 谢咏镰.中西医结合治疗老年冠心病心律失常效果分析[J].深圳中西医结合杂志，2016,26(1):52-53.

[6] 赵艳丽.中西医结合治疗急性ST段抬高心肌梗死溶栓后心律失常的临床疗效[J].中国实用医药，2013,8(21):170-171.

[7] 温春瑜，程红，陈颖颖.炙甘草汤治疗气阴两虚型冠心病室性心律失常的临床效果分析[J].中医临床研究，2018,v.10(07):90-91.

[8] 刘振葳.炙甘草汤辩证论治心律失常的理论探讨与临床分析[D].北京中医药大学，2011.

[9] 王向涛.中西医结合治疗老年室性心律失常的疗效分析[J].中医临床研究，2016(34).

作者简介：白亚萍（1972-），汉族，陕西宝鸡人，主治医师。研究方向：中医内科，心血管方面

万方数据

【中西医结合】

炙甘草汤联合美托洛尔治疗缺血性心律失常的效果

曹云艳
（郑州大学附属郑州中心医院心肺功能科，河南 郑州 450000）

【摘要】 **目的：**观察炙甘草汤联合美托洛尔治疗缺血性心律失常的效果。**方法：**选取 114 例缺血性心律失常患者为研究对象，按照随机抽签法分为对照组和观察组各 57 例。对照组采用美托洛尔治疗，观察组采用炙甘草汤联合美托洛尔治疗。对比两组临床疗效、不良反应情况、心电图变化情况及中医症状积分情况。**结果：**观察组治疗总有效率为 94.74%，高于对照组的 71.93%，差异有统计学意义（P<0.05）。观察组不良反应发生率为 5.26%，低于对照组的 8.77%，但差异无统计学意义（P>0.05）。观察组患者 ST 段压低、ST 段下降持续时间显著低于对照组，中医症状积分明显低于对照组，差异均有统计学意义（P<0.05）。**结论：**采用炙甘草汤联合美托洛尔治疗缺血性心律失常效果显著，能够有效改善患者 ST 段压低，缩短 ST 段下降持续时间，且不增加不良反应。

【关键词】 炙甘草汤；美托洛尔；缺血性心律失常
doi: 10. 3969 / j. issn. 1672-0369. 2020. 01. 046
中图分类号： R541.4 **文献标志码：** B **文章编号：** 1672-0369（2020）01-0114-03

心律失常是临床常见的一种心血管疾病，发病率较高，可单独发病也可与其他疾病伴发。缺血性心律失常通常是由于动脉粥样硬化导致冠状动脉狭窄或闭塞，多发于老年人群，且发病迅速、病死率较高，一旦发病若无法得到及时有效地治疗，会严重威胁患者的生命安全[1]。临床通常采用西药治疗，虽然取得了一定疗效，但无法满足患者的实际需求，延长了康复时间。王永成等[2]采用炙甘草汤联合美托洛尔治疗缺血性心律失常，疗效显著。为了更好地探讨炙甘草汤联合美托洛尔的疗效，本文选取 114 例缺血性心律失常患者为研究对象，现报告如下。

1 资料与方法

1.1 一般资料 选取 2016 年 2 月至 2019 年 5 月郑州大学附属郑州中心医院收治的 114 例缺血性心律失常患者为研究对象，按照随机抽签法分为对照组与观察组各 57 例。纳入标准：经实验室和影像学检查确诊为缺血性心律失常[3]；无药物禁忌证；伴发的非心血管疾病得到控制，且处于相对稳定期。排除标准：入院时仍在服用其他抗心律失常药物者；严重肺功能不全者。本研究经郑州大学附属郑州中心医院伦理委员会批准；患者家属知情本研究，并签署了知情同意书。对照组：男 32 例，女 25 例；年龄 63~82 岁，平均（72.5 ± 8.4）岁；心律失常类型：阵发性心动过速 19 例，窦性心律失常 12 例，快速性心律失常 15 例，缓慢性心律失常 11 例。观察组：男 31 例，女 26 例；年龄 65~82 岁，平均（73.5 ± 8.9）岁；心律失常类型：阵发性心动过速 12 例，窦性心律失常 14 例，快速性心律失常 16 例，缓慢性心律失常 15 例。两组一般资料比较，差异无统计学意义（P>0.05），有可比性。

1.2 方法 对照组采用酒石酸美托洛尔缓释片（西南药业股份有限公司，国药准字 H20033191；50 mg/ 片）口服治疗，50 mg/ 次，2 次 /d，连续用药 2 周。观察组在对照组基础上联合炙甘草汤治疗，组方为：炙甘草 15 g、火麻仁、生姜、麦门冬、人参、阿胶各 10 g、大枣 10 枚。症状加减：肝肾阴虚者添加鳖甲 15 g、龟板 10 g；心虚者添加茯苓 10 g、柏子仁 10 g；水肿少尿者添加葶苈子 15 g、茯苓 10 g；心血瘀阻者添加川芎 6 g、红花 10 g、丹参 15 g；失眠者添加酸枣仁 10 g、合欢皮 5 g。煎服方法：将全部中药用 500 mL 冷水浸泡 30 min。大火烧开后，用小火煎 1 h，取汤汁 300 mL 口服，早晚各 1 次，150 mL/ 次，连续用药 2 周。

1.3 观察指标 （1）比较两组临床疗效。判定标准，显效：患者冠状动脉、脑动脉供血不足等症状完全消除；有效：患者冠状动脉、脑动脉供血不足等症状有所改善；无效：患者冠状动脉、脑动脉供血不足等症状未改善，病情有所加重。治疗有效率 =（显效 + 有效）例数 / 总例数 × 100%。（2）比较两组不良反应发生情况，如心动过缓、胃肠道不适、低血压、恶心等。（3）比较两组患者治疗前后心电图变化情况，如 ST 段压低、ST 段下降持续时间。（4）比较两组患者治疗前后中医症状积分情况。

作者简介：曹云艳（1981.12-），女，汉族，河南省郑州市人，本科，主治医师，研究方向：中西医结合治疗心脏病；电话：13673679558。

按照患者唇甲青紫、气短乏力、心悸、胸闷 4 项主症进行评估。无（0 分）：患者不存在唇甲青紫、气短乏力、心悸、胸闷症状；轻度（1 分）：患者有轻微唇甲青紫、气短乏力、心悸、胸闷症状；中度（2 分）：患者有明显的唇甲青紫、气短乏力、心悸、胸闷症状；重度（3 分）：患者唇甲青紫、气短乏力、心悸、胸闷症状严重。得分范围为 0~12 分，得分越高表示病情越严重

1.4　统计学方法　采用 SPSS 21.0 软件进行统计分析。计量资料以（$\bar{x} \pm s$）表示，比较采用 t 检验，计数资料比较采用 χ^2 检验。以 $P<0.05$ 为差异有统计学意义。

2　结果

2.1　两组临床疗效比较　观察组治疗有效率为 94.74%，明显高于对照组的 71.93%，差异有统计学意义（$P<0.05$）。见表 1。

2.2　两组不良反应发生率比较　观察组不良反应发生率为 5.26%，低于对照组的 8.77%，但差异无统计学意义（$P>0.05$）。见表 2。

2.3　两组患者治疗前后心电图变化情况比较　治疗前，两组患者 ST 段压低、ST 段下降持续时间比较，差异无统计学意义（$P>0.05$）；治疗后，两组 ST 段压低、ST 段下降持续时间均显著低于治疗前，且观察组低于对照组，差异有统计学意义（$P<0.05$）。见表 3。

2.4　两组患者治疗前后中医症状积分比较　治疗前，两组患者中医症状积分比较，差异无统计学意义（$P>0.05$）；治疗后，两组中医症状积分明显低于治疗前，且观察组低于对照组，差异有统计学意义（$P<0.05$）。见表 4。

3　讨论

心律失常是临床上常见的心血管疾病，主要表现为冠状动脉、脑动脉供血不足以及心功能不全等症状，多由触发灶和致心律失常基质共同作用所致，亦与钾离子通道和钙离子通道失活有关[4]。目前，临床对缺血性心律失常患者以西药治疗为主，在控制病情发展的同时会导致一系列不良反应，临床应用受限。

中医认为，缺血性心律不齐属于“胸痹”“心悸”等范畴，通常是由气血、阴阳虚损，导致脉络淤滞，心脉失养，造成气滞、血淤、寒凝、痰湿等痹阻心脉[5]。发病部位在心、肝、脾、肺、肾等，所以临床治疗以活血化淤、通脉止痛为主，方能改善患者心肌功能，减轻临床症状[6]。

美托洛尔是一种 β 受体阻滞剂，可选择性阻断中枢性 β 受体，有效降低交感神经张力，起到

表 1　两组临床疗效比较 [n（%）]

组别	显效	有效	无效	治疗有效率
对照组（n=57）	14（24.56）	27（47.37）	16（28.07）	41（71.93）
观察组（n=57）	28（49.12）	26（45.61）	3（5.26）	54（94.74）
χ^2 值				10.674
P 值				0.001

表 2　两组不良反应发生率比较 [n（%）]

组别	心动过缓	胃肠道不适	低血压	恶心	发生率
对照组（n=57）	2（3.51）	1（1.75）	1（1.75）	1（1.75）	5（8.77）
观察组（n=57）	1（1.75）	0（0.00）	1（1.75）	1（1.75）	3（5.26）
χ^2 值					0.134
P 值					0.714

表 3　两组患者治疗前后心电图变化情况比较（$\bar{x} \pm s$）

组别	ST 段压低（mm）		ST 段下降持续时间（s）	
	治疗前	治疗后	治疗前	治疗后
对照组（n=57）	3.59 ± 0.93	2.37 ± 0.64*	8.97 ± 2.83	6.62 ± 1.99*
观察组（n=57）	3.62 ± 0.91	0.05 ± 0.01*	8.91 ± 2.61	2.31 ± 0.98*
t 值	0.174	27.365	0.118	14.669
P 值	0.431	0.000	0.453	0.000

注：与治疗前比较，*$P<0.05$

表 4 两组患者治疗前、后中医症状积分比较（分，$\bar{x} \pm s$）

组别	时间	唇甲青紫	气短乏力	心悸	胸闷
对照组（n=57）	治疗前	2.1 ± 0.4	2.2 ± 0.5	2.0 ± 0.3	2.0 ± 0.4
观察组（n=57）		2.0 ± 0.5	2.1 ± 0.4	1.9 ± 0.4	2.1 ± 0.6
t 值		1.133	1.109	1.456	1.015
P 值		0.130	0.135	0.074	0.156
对照组（n=57）	治疗后	1.5 ± 1.3*	1.1 ± 0.5*	1.5 ± 0.6*	1.9 ± 0.9
观察组（n=57）		0.9 ± 0.1*	0.3 ± 0.1*	0.6 ± 0.4*	0.7 ± 0.2*
t 值		8.595	18.127	9.423	9.827
P 值		0.000	0.000	0.000	0.000

注：与治疗前比较，*$P<0.05$

降压、控制心率的作用[7]。炙甘草汤又称为复脉汤，来源于《伤寒论》，在治疗缺血性心律失常时，具有益气养血、滋阴复脉的效果[8]。炙甘草作为君药，具有甘温益气、和中缓急、通经利血的功效；人参、大枣、阿胶、麦门冬及火麻仁作为臣药，能够起到滋阴养血、舒筋凉血的作用；生姜作为佐药，具有温阳通脉、舒筋通络的功效。现代药理学研究发现[9]，通过将上述中药配合使用，能够起到抗心律失常、增加冠状动脉流量及抗血栓等作用。另外，本研究按照患者具体症状对处方进行相应加减：对失眠患者，通过添加酸枣仁、合欢皮，可起到养肝宁心、安神解郁的作用；对肝肾阴虚患者，通过添加鳖甲、龟板，可起到滋阴潜阳的功效；对心虚患者，通过添加茯苓、柏子仁，可起到健脾宁心、养心安神的作用；对心血瘀阻患者，通过添加川芎、红花及丹参，可起到活血祛瘀、改善血液的作用[10]。炙甘草汤联合美托洛尔治疗缺血性心律失常疗效显著，这一结果与文献报道相吻合[11]。本研究结果显示，观察组患者临床治疗有效率为 94.74%，高于对照组的 71.93%，差异有统计学意义；观察组患者不良反应发生率为 5.26%，低于对照组的 8.77%，但差异无统计学意义；观察组患者 ST 段压低、ST 段下降持续时间均显著低于对照组，中医症状积分明显低于对照组，差异均有统计学意义。表明炙甘草汤联合美托洛尔治疗缺血性心律失常患者能够起到协同作用，提高疗效。

综上所述，对缺血性心律失常患者，采用炙甘草汤联合美托洛尔治疗效果显著，可改善患者 ST 段压低，缩短 ST 段下降持续时间，不会增加不良反应发生风险。

参考文献

[1] 卓书江，李光智，王转转，等 . 卡维地洛联合美托洛尔治疗老年高血压合并室性心律失常的疗效及对血清 hs-CRP 的影响 [J]. 中西医结合心脑血管病杂志，2018，16（2）：193-195.
[2] 王永成，豆娟娟，马度芳，等 . 经方炙甘草汤合潜阳封髓丹对室性早搏患者自主神经失衡的影响 [J]. 中华中医药杂志，2018，33（5）：205-209.
[3] 俞慧，周生玲，侯媛媛 . 心律失常相关疾病的诊断与治疗 [M]. 北京：军事医学科学出版社，2010：112-114.
[4] 何王兆博，沈晓旭，杨涛，等 . 炙甘草汤对比西药抗心律失常药物治疗室性期前收缩临床随机对照试验的 Meta 分析 [J]. 中国中医急症，2018，27（4）：576-581.
[5] 郭连英，王菊美，安菊岩，等 . 炙甘草汤联合含阿霉素新辅助化疗方案治疗乳腺癌疗效及对心脏功能的影响 [J]. 现代中西医结合杂志，2017，26（26）：3917.
[6] 毋文利，王萍 . 稳心颗粒联合美托洛尔和门冬氨酸钾镁盐治疗老年缺血性心律失常临床效果及安全性分析 [J]. 解放军医药杂志，2018，30（4）：79-82.
[7] 齐晓贵，徐海波，辛凤萍，等 . 美托洛尔对高血压性心脏病患者不同时间段的 QT 间期离散度及室性心律失常发生率影响研究 [J]. 山西医药杂志，2017，46（22）：2763-2765.
[8] 赵新华，周艺，王莹，等 . 曲美他嗪联合炙甘草汤治疗老年慢性心力衰竭合并窦性心动过缓的临床研究 [J]. 中西医结合心脑血管病杂志，2018，16（16）：90-92.
[9] 王宏志，赵敏，李亚蒙，等 . 丹红注射液联合酒石酸美托洛尔对冠心病心律失常患者血流动力学及心电图的影响 [J]. 心血管康复医学杂志，2018，27（3）:1426-1428.
[10] 申兴勇，袁平 . 加减炙甘草汤对乳腺癌化疗致心脏毒性的临床观察及对心功能的保护作用分析 [J]. 癌症进展，2018，16（1）：106-109.
[11] 王兆博，沈晓旭，杨涛，等 . 炙甘草汤对比西药抗心律失常药物治疗室性期前收缩临床随机对照试验的 Meta 分析 [J]. 中国中医急症，2018，27（4）：576-581.

编辑：申蓝

DOI: 10.13194/j.issn.1673-842x.2018.03.047

炙甘草汤联合美托洛尔治疗持续性心房颤动临床研究

陈婷，陈颖，范秀花，常征利，杜飚
（衢州市柯城区人民医院，浙江 衢州 324000）

摘 要：目的：观察炙甘草汤联合美托洛尔治疗持续性房颤的临床疗效，并探究其可能的作用机理。方法：采用平行、随机、对照试验方案，选择符合持续性房颤（气阴两虚证）诊断标准患者60例，随机分为治疗组和对照组各30例，给予基础治疗的同时，治疗组给予炙甘草汤联合美托洛尔治疗，对照组单用美托洛尔治疗，疗程12周。比较治疗前、后每例患者的左房前后径（LAD1）、左室射血分数（LVEF）、静息时心率、运动后心率、步行距离及AFEQT评分改善情况；检测高敏C反应蛋白（hypersensitive C-reactive protein，hs-CRP）、N末端B型利钠肽前体（N-terminal prohormone of brain natriuretic peptide，NT-proBNP）、醛固酮（aldosterone，Ald）水平变化情况。并记录治疗前后三大常规，肝、肾功能等安全性指标与不良反应事件。结果：两组患者临床疗效比较，治疗组总有效率93.33%，对照组总有效率76.67%，治疗组优于对照组（$P<0.05$）。两组均可有效控制心室率，降低LAD1、提高LVEF及6 min步行距离，尤其在控制静息时心率及提高6 min步行距离方面治疗组优于对照组（$P<0.05$）。治疗组患者治疗后AFEQT评分较治疗前显著提高（$P<0.05$）。治疗组患者血浆hs-CRP、NT-proBNP、Ald水平均较治疗前下降，对照组NT-proBNP及Ald水平较治疗前下降，差异有统计学意义（$P<0.05$）。结论：炙甘草汤联合美托洛尔治疗持续性房颤可有效控制心室率，改善患者心功能及生活质量，其作用机制可能与抑制炎症反应、影响心房重构有关。

关键词：炙甘草汤；美托洛尔；持续性房颤；生活质量
中图分类号：R541.75 **文献标志码**：A **文章编号**：1673-842X(2018) 03-0163-04

Clinical Research of *Zhigancao* Decoction Combined with Metoprolol in Treating Persistent Atrial Fibrillation

CHEN Ting, CHEN Ying, FAN Xiuhua, CHANG Zhengli, DU Biao
(Quzhou Kecheng Hospital, Quzhou 324000, Zhejiang, China)

Abstract: *Objective*: To observe effect of *Zhigancao* Decoction combined with metoprolol on persistent atrial fibrillation and to investigate the possible action mechanisms. *Methods*: In a parallel, randomized trial, 60 patients with VD (syndrome of Qi and Yin Deficiency)were enrolled and divided into the treatment group and control group, 30 in each group. While both groups were given routine treatment, the treatment group was given *Zhigancao* Decoction combined with metoprolol and the control group was given metoprolol. The ventricular rates at rest, the ventricular rates after walk, the distances of 6-minute walk, LAD1, LVEF, the scores of AFEQT, hypersensitive C-reactive protein (hs-CRP), N-terminal prohormone of brain natriuretic peptide (NT-proBNP)and aldosterone (Ald) were recorded respectively for each patient before the treatment and 12 weeks after the treatment, as well as security indicators of blood, urine and excrement tests, liver, kidney and thyroid functions, and adverse events. *Results*: Twelve weeks after the treatment, the total effective rate of the treatment group was 93.33%, while that of the control group was 76.67%, which suggested that the treatment group worked better ($P<0.05$). The ventricular rates and LAD1 after the treatment decreased significantly, while the distances of 6-minute walk and LVEF increased significantly in both groups ($P<0.05$); especially the ventricular rates at rest and the distances of 6-minute walk in treatment group were superior to those in control group ($P<0.05$); The score of AFEQT in treatment group increased significantly after the treatment ($P<0.05$); The levels of plasma hs-CRP, NT-proBNP and Ald were decreased significantly than before in the treatment group ($P<0.05$), while the levels of plasma NT-proBNP and Ald were decreased significantly in control group ($P<0.05$). *Conclusion*: The treatment of persistent atrial fibrillation with *Zhigancao* Decoction combined with metoprolol is

基金项目：衢州市指导性科技计划项目（2016134）
作者简介：陈婷（1983-），女，江苏无锡人，副主任中医师，博士，研究方向：心血管内科疾病。

effective in decreasing the the ventricular rates and improving patients' cardiac functions and qualities of life. The mechanism of action could be related to the inhibition of inflammatory response and atrial remodeling.

Keywords: *Zhigancao* Decoction; metoprolol; persistent atrial fibrillation; quality of life

心房颤动(atrial fibrillation, AF)是一种以快速、无序心房电活动为特征的室上性快速性心律失常。持续性房颤(persistent AF)是指持续时间超过7 d的房颤。房颤发作时心房泵血功能基本丧失,心排出量显著降低,引起心力衰竭[1]。与此同时,心房内、特别是左心耳内易形成血栓,增加脑栓塞的风险,严重影响患者的生活质量(quality of life, QOL)。控制心室率是药物治疗持续性房颤主要目标之一[2],但西药在治疗的同时具有明显的致心律失常作用及不良反应,限制了临床的使用。中医药治疗房颤发挥出了优势和特色,炙甘草汤出自《伤寒论》,是治疗心动悸、脉结代的经典方剂,本研究发现炙甘草汤联合美托洛尔治疗持续性房颤疗效显著,现报道如下。

1　临床资料

1.1　一般资料

采用平行、随机、对照方案,收集从2015年12月—2016年12月我院门诊及住院诊断持续性房颤患者60例,采用随机数字表法分为治疗组和对照组各30例。治疗组男16例,女14例;平均年龄(62.1±8.9)岁;平均病程(3.7±1.4)年;原发病为冠心病15例,高血压性心脏病10例,特发性房颤5例。对照组男17例,女13例;平均年龄(63.7±9.5)岁;平均病程(3.9±1.7)年;原发病为冠心病17例,高血压性心脏病9例,特发性房颤4例。两组一般资料有可比性。

1.2　纳入标准

1.2.1　西医诊断标准

符合2015年《心房颤动:目前的认识和治疗建议》的诊断标准[2]。

1.2.2　中医诊断标准

符合《中药新药临床研究指导原则(第二辑)》[3]中治疗心悸临床研究指导原则,中医辨证属气阴两虚者,症见:心悸气短,动则加剧,乏力口渴,舌淡或稍红,苔白,脉沉细弱或结。

1.3　排除标准

心功能Ⅳ级、急性心肌梗死、低血压、严重肝肾功能不全、电解质紊乱、高度房室传导阻滞、预激综合征、慢性肺部疾病患者;瓣膜病、甲状腺功能亢进引起房颤;正在服用抗心律失常药物在5个半衰期内或对本试验中药物有过敏史的患者;有感染性疾病、恶性肿瘤等影响CRP水平的疾患。

2　方法

2.1　治疗方法

基础治疗:两组患者均给予原发病的基础治疗,如降压、降脂、降糖、抗凝、抗血小板等药物治疗,均不得使用其他抗心律失常药。对照组:口服酒石酸美托洛尔(阿斯利康公司生产,批号:1603A33),从12.5 mg/d开始,逐渐增至50.0 mg/d。治疗组:在对照组的基础上服用炙甘草汤(炙甘草30 g,党参30 g,生地50 g,阿胶15 g,麦冬20 g,桂枝20 g,麻仁30 g,生姜10 g,大枣15 g)水煎服,每日1剂。疗程12周。

2.2　观测指标及疗效评定

2.2.1　安全性观测

(1)一般检查项目:体温、血压、脉搏、呼吸。(2)三大常规,肝、肾功能检查,以上指标于治疗前1周内和治疗后1周内各检测记录1次。(3)不良反应:包括不良反应的发生时间、原因、表现程度(1~4级)以及是否影响试验的进行和处理措施。

2.2.2　疗效性观测

①观察期间每月门诊随访1次,常规作心电图,治疗前、后各作1次动态心电图。②治疗前后静息时心率、运动后心率和运动距离测定:采用6 min步行法[4],患者静坐15 min后测定1 min静息时心率,在50 m走廊内来回走动,可站立休息,记录6 min内步行距离及1 min运动后心率。③治疗前、后行心脏超声心动图检查评估心功能:测定左心房前后径(LAD1)、左心室射血分数(LVEF)。④治疗前、后患者在清晨空腹清醒时,窦性心律情况下抽取外周静脉血,测定血浆高敏C反应蛋白(hypersensitive C-reactive protein, hs-CRP)、N末端B型利钠肽前体(N-terminal prohormone of brain natriuretic peptide, NT-proBNP)、醛固酮(aldosterone, Ald)。⑤治疗前、后采用AFEQT量表[5]评估房颤对生活质量的影响:从症状、日常活动、治疗焦虑和治疗满意度4个维度进行评估,前3个维度组成总体评分,0~100分,分数越高说明生活状态越好,精神负担相对较轻。

2.3　中医疗效判定标准

参照《中药新药临床研究指导原则(第二辑)》中治疗心悸临床研究指导原则拟定。①显效:临床症状消失,静息时心室率保持在60~80次/min,轻微活动≤100次/min。②有效:临床症状大部分消失,静息时心室率80~90次/min,120次/min≥轻微活动后>100次/min。③无效:临床症状和心室率未达到上述标准。[4,6]

2.4　统计学方法

采用SPSS17.0统计软件进行分析。定量资料采用均数($\bar{x}$)描述平均水平,采用标准差(s)描述变异程度;使用两独立样本t检验进行组间比较,组内治疗前后采用配对t检验。等级资料(即疗效指标)的组间比较则采用两独立样本的秩和检验;$P<0.05$表示具有统计学差异。

3　结果

3.1　两组患者临床疗效比较

两组比较,$Z=-2.187$,$P=0.029$。见表1。

表1　两组临床疗效比较

组别	例数	显效	有效	无效	总有效率(%)
治疗组	30	15	13	2	93.33
对照组	30	8	15	7	76.67

3.2　两组患者治疗前后左房前后径、左室射血分数、静息时心率、运动后心率、步行距离比较

见表2。

3.3　两组患者治疗前后hs-CRP、NT-proBNP、Ald比较

见表3。

表2　两组患者治疗前后左房前后径、左室射血分数、静息时心率、运动后心率、步行距离比较（$\bar{x}\pm s$）

组别	时间	例数	左房前后径（mm）	左室射血分数（%）	静息时心率（次/min）	运动后心率（次/min）	6 min步行距离（m）
治疗组	治疗前	30	37.63±4.63	49.93±8.33	100.00±13.38	133.00±14.57	332.03±40.87
	治疗后	30	34.97±4.70*	56.37±7.80*	78.37±10.00*△	96.37±10.80*	378.70±40.47*△
对照组	治疗前	30	37.27±4.06	49.20±9.08	102.10±13.85	130.43±15.57	327.47±43.52
	治疗后	30	34.47±4.13*	54.80±7.81*	83.97±10.71*	99.40±10.79*	364.57±42.10*

注：与本组治疗前比较，*$P<0.05$；与对照组治疗后比较，△$P<0.05$

表3　两组患者治疗前后hs-CRP、NT-proBNP、Ald比较（$\bar{x}\pm s$）

组别	时间	例数	hs-CRP（mg/L）	NT-proBNP（ng/L）	Ald（ng/L）
治疗组	治疗前	30	5.93±2.12	292.73±123.76	108.30±44.92
	治疗后	30	4.67±1.09*△	165.27±76.60*	88.87±35.14*
对照组	治疗前	30	6.00±1.93	272.83±121.714	111.80±47.05
	治疗后	30	5.23±1.07	182.33±54.95*	93.53±33.37*

注：与本组治疗前比较，*$P<0.05$；与对照组治疗后比较，△$P<0.05$

3.4　两组患者治疗前后AFEQT评分比较

见表4。

表4　两组患者治疗前后AFEQT评分比较（分，$\bar{x}\pm s$）

组别	时间	例数	AFEQT
治疗组	治疗前	30	59.60±11.13
	治疗后	30	67.17±12.27*
对照组	治疗前	30	60.53±10.27
	治疗后	30	65.27±11.45

注：与本组治疗前比较，*$P<0.05$

3.5　安全性指标

两组患者治疗前、后均进行三大常规及肝肾功能检查，其中对照组出现1例心动过缓，属于美托洛尔不良反应，其余指标无明显异常。两组不良反应情况比较无统计学差异（$P>0.05$）。

4　讨论

心房颤动属于中医学“心悸”“怔忡”等范畴，临床常出现心悸、头晕、胸闷等不适症状，并且大幅增加了缺血性卒中的风险，从而也加重患者的精神负担，严重影响了患者的生活质量。持续性房颤的治疗一直是临床上一大难题[7]，治疗策略主要有药物及导管消融手术治疗，但后者存在消融术式较复杂，成功率较低，复发率高，心肌穿孔和血栓栓塞等严重并发症风险高等情况[8-10]，临床疗效乃至预后尚不明确[11-12]。因此，药物治疗是持续性房颤治疗的重要策略，通过控制心室率起到改善临床症状及生活质量，降低诱发心动过速性心肌病风险的作用。总体而言，β受体阻滞剂是最广泛应用的心室率控制药物，但有使心衰失代偿、加重阻塞性肺疾病等不良反应。面对AF的发病机制尚不明确，药物不良反应多的难题，中药治疗AF有多靶点、多途径的优势。炙甘草汤又名复脉汤，源于汉代张仲景《伤寒论·辨太阳病脉证并治》，原文曰：“伤寒脉结代，心动悸，炙甘草汤主之”。炙甘草汤重用炙甘草为甘温益气、缓急养心的君药；用生地、麦冬、麻仁、阿胶以滋阴养血，资生化之源；用人参、大枣，健脾益气，共为臣药；桂枝、生姜、清酒为佐，以温心阳通血脉。诸药合用，甘润、辛燥并用，温而不燥，滋而不腻，益心气、补心血，滋心阴、温心阳，使气血充沛，血脉畅通，脉始复常，故又名复脉汤。本研究发现炙甘草汤联合美托洛尔治疗持续性房颤疗效显著，优于单用美托洛尔。

房颤持续存在，心房收缩功能丧失，引起心房电重构和结构重构的过程使心房逐步扩大，血栓栓塞风险也可能增加。房颤是心衰发展的独立危险因素。过快的心室率使心室充盈时间缩短、心排出量降低、血压下降、冠状动脉血液灌注量减少而诱发或加重心衰。对于已经存在心室舒张功能降低的患者，由于其心室充盈更加依赖心房收缩，故一旦房颤发作，心排出量降低会更为明显，心功能的恶化也会更为显著。此外，当房颤的心室率持续超过130次/min时，可能导致心动过速性心肌病，其发生与心肌的能量耗竭、重构、缺血等因素有关[13]。因此，控制心室率是持续性房颤治疗的基本目标之一，充分的心室率控制可使LVEF明显增加[14]。大多数患者AF发作时常出现心悸、胸闷等不适症状，或引起缺血性脑卒中，严重影响患者的生活质量，而生活质量已成为房颤临床试验及医疗实践过程中的一项重要评价指标[2]。本研究发现炙甘草汤联合美托洛尔治疗持续性房颤，可有效控制心室率，降低LAD1、提高LVEF及6 min步行距离，尤其在控制静息时心率及提高6 min步行距离方面优于单用美托洛尔，从而显著改善持续性AF患者生活质量。

房颤引起心房重构，肾素-血管紧张素-醛固酮系统（RAAS）被激活[15]，发生细胞凋亡、细胞因子释放、炎症、氧化应激等一系列级联反应[16]，大量释放的Ald可促进成心肌纤维细胞分裂增殖导致心肌纤维化，增加房颤的风险。研究发现，房颤时心房肌组织存在炎性细胞浸润，提示炎症与房颤之间可能存在相关性[17]。血清BNP浓度可以准确反映心脏受到的牵张力，心房发生结构重构，心房肌细胞肥大、纤维化和炎症浸润使其张力升高，从而激活心房肌细胞合成、释放BNP。由此说明，CRP、BNP及Ald既是影响AF患者心房重构的重要因子，也是反映AF发生、进展及预后的重要监测因子。本研究发现，炙甘草汤联合美托洛尔治疗可降低持续性房颤患者血浆hs-CRP、BNP及Ald水平，尤其可明显降低hs-CRP水平。由此我们推论，通过抑制炎症反应，影响心房重构可能是炙甘草汤联合美托洛尔治疗持续性房颤的机理之一。◆

参考文献

[1] Packer DL, Bardy GH, Worley SJ, et al. Tachycardia-induced cardiomyopathy: a reversible form of left ventricular dysfunction[J]. Am J Cardiol, 1986, 57: 563-570.

[2] 曹克将，陈柯萍，陈明龙，等. 心房颤动：目前的认识和治疗建议（2015）[J]. 中华心律失常学杂志，2015，19（5）：321-384.

[3] 郑筱萸. 中药新药临床研究指导原则（第二辑）[S]. 北京：中国医药科技出版社，1995：91-94.

[4] Gordon H, Michad J. The 6 minute walk: a new measure of exercise capacity in patients wlth chronic heart failure[J]. Can Med Assoe J, 1985, 132: 919-922.

[5] Spertus J, Dorian P, BubienR, et al. Development and validation of the Atrial Fibrillation Effect on Quality of-Life (AFEQT) questionnaire in patients with atrial fibrillation[J]. Circ Arrhythm Electrophysiol, 2011, 4(1): 15-25.

DOI: 10.13194/j.issn.1673-842x.2018.03.048

高血压早期肾损害中西医研究进展

包伟[1],黄晓佳[1],顾宁[2]
(1.南京中医药大学,江苏 南京 210023;2.南京中医药大学第三附属医院,江苏 南京 210001)

摘 要: 高血压是遗传和环境因素引起的进行性心血管综合征,长期血压增高引起肾内小动脉及细小动脉病变,导致肾脏发生缺血性改变,即高血压肾损害,是终末期肾脏疾病(ESRD)的重要病因,该文从祖国医学对高血压早期肾损害的认识、高血压肾损害的早期诊断及中西医结合治疗等方面,综述了各位学者的研究,旨在为临床和科研工作人员提供参考。

关键词: 高血压早期肾损害;中西医;研究进展
中图分类号: R544.1 **文献标志码:** A **文章编号:** 1673-842X(2018) 03-0166-04

Research Progress of Chinese and Western Medicine for Early Renal Damage Induced by Hypertension

BAO Wei[1], HUANG Xiaojia[1], GU Ning[2]
(1.Nanjing University of Chinese Medicine, Nanjing 210023, Jiangsu, China; 2. Third Affiliated Hospital of Nanjing University of Traditional Chinese Medicine, Nanjing 210001, Jiangsu, China)

Abstract: Hypertension is sexual cardiovascular syndrome caused by genetic and environmental factors, hypertensive nephropathy, the syndrome clinically resulting from intrarenal arteriolar and small arterial lesion leading to renal ischemia, has been the critical cause for end-stage renal disease (ESRD). Here is to make a review on the progress in early diagnosis of hypertensive nephropathy, treatment with integrated traditional Chinese and Western medicine and the recognition of modern Chinese medicine. The purpose is to provide reference for clinical and scientific research workers.

Keywords: hypertension; early renal damage; Chinese and Western medicine; research progress

高血压肾损害是指长期高血压(> 5年且血压> 150/100 mmHg)引起小动脉硬化,造成肾脏结构和功能的改变[1]。高血压对肾脏的损害是循序渐进的,早期肾损害经过治疗可以逆转,但常因症状隐匿而被忽视,一旦出现肌酐(Cr)升高、蛋白尿等临床症状,已经是严重的肾功能损害,目前高血压肾损害已成为终末期肾病(End stage renal disease, ESRD)的主要原因之一,本文就近年来对高血压早期肾损害的诊治研究进展作出综述如下。

[6] 胡大一,马长生.心脏病学实践 2002——规范化治疗[M].北京:人民卫生出版社,2002:4316-4381.
[7] 刘朝硕,邱接,刘启明.运动与心房颤动关系的研究进展[J].中国介入心脏病学杂志,2015,23(7):409-411.
[8] Cappato R, Calkins H, Chen SA, et al. Updated worldwide survey on the methods, efficacy, and safety of catheter ablation for human atrial fibrillation [J]. Circ Arrhythmia Electrophysiol,2010,3: 32-38.
[9] Cappato R, Calkins H, Chen SA, et al. Prevalence and causes of fatal outcome in catheter ablation of atrial fibrillation[J].J Am Coll Cardiol,2009,53: 1798-1803.
[10] Belhassen B. A 1 per 1,000 mortality rate after catheter ablation of atrial fibrillation an acceptable risk? [J].J Am Coll Cardiol, 2009,53: 1804-1806.
[11] Oral H, Chugh A, Good E, et al. Radiofrequency catheter ablation of chronic atrial fibrillation guided by complex electrograms[J].Circulation,2007,115: 2606-2612.
[12] Pappone C, Oreto G, Rosanio S, et al. Atrial electroanatomic remodeling aftercircumferential radiofrequency pulmonary veinablation-efficacy of an anatomic approach in a large cohort of patients with atrial fibrillation[J].Circulation,2001,104: 2539-2544.
[13] Shinbane JS, Wood MA, Jensen DN, et al. Tachycardia-induced cardiomyopathy: a review of animal models and clinical studies [J].J Am Coll Cardiol,1997,29: 709-715.
[14] Weerasooriya R, Davis M, Powell A, et al. The Australian intervention randomized control of rate in atrial fibrillation trial[J].J Am Coll Cardiol,2003,41: 1697-1702.
[15] Goette A, Staack T, Rocken C, et al. Increased expression of extracellular signal-regulated kinase and angiotensin-converting enzyme in human atria during atrial fibrillation[J].J Am Coll Cardiol,2000,35: 1669-1677.
[16] Iravanian S, Duley SC. The renin-angiotensin-aldosterone system (RAAS) and cardiac arrhythmias[J].Heart Rhythm, 2008,5: S12-S17.
[17] Frustaci A, Chimenti C, Bellocci F, et al. Histological substrate of atrial biopsies in patients with lone atrial fibrillation[J]. Circulation,1997,96: 1180-1184.

基金项目: 国家中医药管理局"国家中医临床研究基地业务建设第二批科研专项课题"(JDZX2015103);南京市卫生和计划生育委员会"南京市中医药转化医学基地项目"(ZYYZH1304)

作者简介: 包伟(1992-),男,江苏苏州人,硕士研究生,研究方向:中医及中西医结合心血管疾病临床研究。
通讯作者: 顾宁(1963-),男,江苏南京人,教授、主任医师,博士研究生导师,博士,研究方向:中医及中西医结合心血管疾病临床与基础研究。E-mail: jsguning@163.com。

考虑到体内环境复杂，与体外研究存在一定的差异，因此尚需进行动物和临床实验进一步验证联合用药时药物代谢动力学与耐药突变体选择的关系。

参 考 文 献

［1］ Juan-Ignacio A, Ana GC, Paloma GH, et al. Vancomycin MICs did not creep in Staphylococcus aureus isolates from 2002 to 2006 in a setting with low vancomycin usage. Journal of Antimicrobial Chemotherapy, 2008, 62(4):773-775.
［2］ 王琴，潘静. 痰热清注射液的药理作用和临床应用. 华北国防医药，2010, 22(1):41-43.
［3］ 张世有，李冬生. 痰热清治疗社区获得性肺炎 60 例临床观察. 黑龙江中医药，2011, 40(3):19-20.
［4］ 宋志香，李刘坤，李兴广. 中药痰热清注射液治疗 MRSA 感染性肺炎的临床观察研究. 中华医院感染学杂志，2010, 20(11): 1596-1598.

［收稿日期：2016-02-02］

中西医结合治疗冠心病心律失常的疗效观察

陈晓琳

【摘要】 目的　探讨炙甘草汤加减联合酒石酸美托洛尔治疗冠心病心律失常的临床疗效。**方法**　97 例冠心病心律失常患者，随机分为观察组 (50 例) 和对照组 (47 例)。观察组口服炙甘草汤加减联合酒石酸美托洛尔，对照组单纯口服酒石酸美托洛尔，两组患者治疗时间均为 4 周。观察两组患者治疗后临床疗效及不良反应发生情况。**结果**　治疗 4 周后，观察组总有效率为 86.00%, 对照组为 70.21%, 比较差异具有统计学意义 ($P<0.05$)；观察组患者不良反应发生率为 4.00%, 低于对照组的 14.89%($P<0.05$)。**结论**　炙甘草汤加减联合酒石酸美托洛尔治疗冠心病心律失常疗效显著，且不良反应发生率低，值得临床推广使用。

【关键词】 冠心病；心律失常；炙甘草汤；酒石酸美托洛尔

DOI：10.14164/j.cnki.cn11-5581/r.2016.09.116

心律失常是冠心病常见并发症之一，可单独发病，也可与心力衰竭、心绞痛同时发病，严重时可能会危及患者的生命安全[1]。祖国医学认为其属于“心悸”、“怔忡”等范畴。《伤寒论·辨太阳病证并治》中云：“伤寒，脉结代，心动悸，炙甘草汤主之。”《丹溪手镜·悸》中云：“有气虚者，由阳明内弱，心下空虚，正气内动，心悸脉代，气血内虚也，宜炙甘草汤补之”。故作者予以炙甘草汤加减联合酒石酸美托洛尔口服治疗本病，观察其临床疗效，现将治疗效果介绍如下。

1　资料与方法

1.1　一般资料　选取 2013 年 1 月 ~2015 年 1 月就诊于本院的 97 例冠心病心律失常患者，随机分为观察组 (50 例) 和对照组 (47 例)。观察组男 28 例，女 22 例；年龄最大 74 岁，最小 40 岁，平均年龄 (59.8±6.7) 岁；室上性早搏 36 例，室性早搏 14 例。对照组男 28 例，女 19 例；年龄最大 76 岁，最小 41 岁，平均年龄 (61.3±7.4) 岁；室上性早搏 32 例，室性早搏 15 例。两组患者一般资料比较差异无统计学意义 ($P>0.05$), 具有可比性。

1.2　纳入及排除标准　纳入标准：符合《实用内科学》[2] 冠心病诊断标准，并经 24 h 动态心电图或心电图检查诊断为心律失常；并且中医证型属气阴两虚型；治疗前所有患者均签署知情同意书。排除标准[3]：其他疾病导致的心律失常；严重的室性心动过速、室上性心动过速、慢 - 快综合征和心房扑动等；急性心肌梗死导致的心律失常；排除 1 周内服用其他抗心律失常药物。

1.3　治疗方法　两组患者均给予酒石酸美托洛尔口服，25~50 mg/ 次，2 次 /d。观察组在此基础上给予炙甘草汤加减用药，具体方药如下：生地黄 30 g、炙甘草 15 g、生姜 9 g、党参 15 g、麦冬 15 g、阿胶 15 g、桂枝 10 g、丹参 15 g、茯苓 15 g、白术 10 g、大枣 6 枚组成。随症加减：心阳虚甚者加附子，将党参换为人参；阴虚火旺者加黄柏、知母；失眠者加夜交藤、酸枣仁；恶心呕吐者加陈皮、半夏；气滞胸闷者加瓜蒌、薤白。水煎服，1 剂 /d, 早晚各服 1 次，共服用 4 周。

1.4　观察指标及疗效判定标准[4]　比较两组临床疗效及不良反应发生情况。显效：治疗后患者的临床症状明显改善，早搏减少 >90%, 心电图检查结果显示为正常；有效：治疗后患者的临床症状有所改善或发作间隙期延长，90% ≥早搏减少≥ 50%, 心电图检查结果明显改善；无效：治疗后患者的临床症状和心电图检查结果无明显变化，早搏减少 <50%。总有效率 = 显效率 + 有效率。

1.5　统计学方法　采用 SPSS13.0 统计学软件对数据进行统计分析。计量资料以均数 ± 标准差 ($\bar{x}\pm s$) 表示，采用 t 检验；计数资料以率 (%) 表示，采用 χ^2 检验。$P<0.05$ 为差异具有统计学意义。

2　结果

2.1　两组患者临床疗效比较　观察组总有效率为 86.00%、对照组为 70.21%, 比较差异具有统计学意义 ($P<0.05$)。见表 1。

表 1　两组患者临床疗效比较［n(%)］

组别	例数	显效	有效	无效	总有效
观察组	50	28(56.00)	15(30.00)	7(14.00)	43(86.00)[a]
对照组	47	18(38.30)	15(31.91)	14(29.79)	33(70.21)

注：与对照组比较，[a]$P<0.05$

作者单位：118000　丹东市中医院

2.2 两组患者不良反应发生率比较 治疗期间，观察组出现头晕1例、恶心1例，经调理或停药后不良反应症状很快得到缓解，不良反应发生率为4.00%；对照组出现头晕4例、恶心2例、心动过缓1例，停药后或减少药物剂量不良反应症状同样得到缓解，不良反应发生率为14.89%。两组比较差异有统计学意义(P<0.05)。

3 讨论

冠心病心律失常的发病率现呈逐年上升的趋势，已经严重影响了患者的生活质量，甚至危及生命。美托洛尔属Ⅱ类阻断β肾上腺素能受体，是常见的抗心律失常药物，其通过降低起搏细胞的自律性、延长室上性传导时间、阻断交感神经活血增加等作用，从而使心率减慢；还能够改善缺血区的血供情况，减少心肌耗氧量，从而达到抗心律失常作用[5]。

《证治准绳·惊悸恐》曰："心悸之由，不越二种，一者虚也，二者饮也。"指出了心悸的发病机制。本研究选用炙甘草汤加减治疗气阴两虚型心律失常具有益气滋阴、温阳通脉之功效。炙甘草汤出自《伤寒论》，本研究在原方基础上加丹参、茯苓、白术以增强活血化瘀，健脾祛湿之功效。观察组采用炙苷草汤加减联合酒石酸美托洛尔治疗本病的临床疗效明显优于对照组，且治疗过程中不良反应发生率也显著低于对照组(P<0.05)，值得临床推广使用。

参 考 文 献

［1］ 滕祖训.炙甘草汤加减治疗冠心病心律失常的临床价值分析.中国疗养医学，2014，1(4):330-331.
［2］ 陈灏珠.实用内科学.北京：人民卫生出版社，2001:1359.
［3］ 陈湘君.中医内科学.上海：上海科学技术出版社，2001:72-79.
［4］ 郜小燕.炙甘草汤加减联合西药治疗冠心病心律失常的临床分析.中西医结合心血管病杂志，2014，2(12):39-40.
［5］ 姜果.炙甘草汤联合美托洛尔治疗冠心病心律失常疗效观察.中国医药指南，2012，10(1):214-215.

［收稿日期：2015-12-14］

舒芬太尼复合不同剂量地佐辛在老年术后患者自控静脉镇痛中应用分析

朱莉娅

【摘要】 目的 研究舒芬太尼联合不同剂量地佐辛在老年术后患者自控静脉镇痛中的临床应用效果。**方法** 90例老年腹部手术患者，随机分成A、B、C三组，各30例。A组给予注射舒芬太尼治疗；B组在A组基础上给予0.1 mg/kg地佐辛治疗；C组在A组基础上给予0.2 mg/kg地佐辛治疗。对比三组患者术后疼痛等级评分、镇静评分以及不良反应发生率。**结果** 治疗后，B、C两组术后疼痛等级评分均低于A组，差异有统计学意义(P<0.05)；B、C两组镇静评分高于A组，差异有统计学意义(P<0.05)；C组不良反应发生率高于A、B两组，差异有统计学意义(P<0.05)。**结论** 舒芬太尼联合0.1 mg/kg地佐辛治疗老年患者术后自控静脉镇痛效果最好。

【关键词】 舒芬太尼；地佐辛；自控静脉镇痛
DOI：10.14164/j.cnki.cn11-5581/r.2016.09.117

外科手术后的疼痛刺激是引发并发症、影响患者康复进程的主要危险因素之一[1]。术后剧烈的疼痛会加重患者体内脏器的负荷，引发脏器继发性功能损伤，从而会导致围术期并发症的发生[2]。本研究就舒芬太尼联合不同剂量地佐辛在老年术后患者自控静脉镇痛中的临床应用效果进行统计和分析，报告如下。

1 资料与方法

1.1 一般资料 选取2014年7月~2015年8月本院进行腹部外科手术老年患者90例，随机分为A、B、C三组，每组30例。A组中男16例，女14例，年龄58~86岁，平均年龄(77.83±9.33)岁；B组中男15例，女15例，年龄55~84岁，平均年龄(77.44±9.67)岁；C组中男14例，女16例，年龄59~97岁，平均年龄(77.58±12.67)岁。三组患者年龄、性别等一般资料比较差异无统计学意义(P>0.05)，具有可比性。

1.2 方法 A组给予1 μg/kg舒芬太尼(宜昌人福药业有限公司，国药准字H20054256)治疗；B组在A组基础上给予0.1 mg/kg地佐辛(扬子江药业集团有限公司，国药准字H20080328)治疗；C组在A组基础上给予0.2 mg/kg地佐辛进行治疗。三组均静脉注射。

1.3 评价标准 统计对比三组患者术后疼痛等级评分、镇静评分以及不良反应发生率。其中，患者术后疼痛程度评分，满分10分。0分为无疼痛感觉，10分为疼痛感特别剧烈。

1.4 统计学方法 采用SPSS16.0统计学软件对数据进行统计分析。计量资料以均数±标准差($\bar{x}\pm s$)表示，采用t检验；计数资料以率(%)表示，采用χ^2检验。P<0.05为差异具有统计学意义。

2 结果

2.1 三组患者术后疼痛等级评分比较 治疗后，A组疼痛等级评分为(7.68±1.31)分，B组为(4.18±1.68)分，C组为(4.16±1.76)分，B、C两组疼痛等级评分比较差异无统计学意义(P>0.05)；A组疼痛等级评分明显高于B、C两组，差异有统计学意义(P<0.05)。

2.2 三组患者治疗后镇静评分比较 治疗后，A组镇静评分为(1.68±0.31)分，B组为(3.58±0.68)分，C组为(3.60±0.16)

作者单位：472000 河南省三门峡市中医院麻醉科

炙甘草汤联合美托洛尔对慢性房颤患者心室率和血浆 Hcy 水平的影响

崔晓婷
(北京市和平里医院,北京 100013)

［摘要］ 目的　观察炙甘草汤联合美托洛尔对慢性房颤患者心室率和血浆同型半胱氨酸(Hcy)水平的影响。方法　将 80 例慢性房颤患者随机分为治疗组与对照组各 40 例,2 组均以美托洛尔 25 mg 口服 2 次/d 作为基础治疗,治疗组同时给予炙甘草汤 1 剂/d 口服。2 组疗程均为 8 周。观察 2 组治疗前后超声心功能指标、中医症候评分、心室率及血浆 Hcy 水平变化情况。结果　治疗后治疗组 LVEF、CO、CL 及 E/A 水平均较治疗前及对照组明显改善(P 均 < 0.05),而对照组上述指标均无明显变化(P > 0.05);治疗后治疗组心悸、胸闷、头晕、乏力评分和对照组心悸、胸闷、头晕评分均明显降低(P 均 < 0.05),且治疗组各项评分均明显低于对照组(P 均 < 0.05)。治疗后治疗组心室率、血浆 Hcy 水平和对照组心室率均明显降低(P 均 < 0.05),且治疗组心室率、血浆 Hcy 水平均明显低于对照组(P 均 < 0.05)。结论　炙甘草汤联合美托洛尔可有效改善慢性房颤患者心功能及中医症候,降低心室率及 Hcy 水平。

［关键词］ 炙甘草汤;美托洛尔;慢性房颤;心室率;Hcy
doi:10.3969/j.issn.1008-8849.2017.02.017
［中图分类号］ R541.75　［文献标识码］ B　［文章编号］ 1008-8849(2017)02-0162-03

心房颤动是临床最常见的快速性心律失常,其可引起心悸、乏力、胸闷、胸痛、呼吸困难、头晕、晕厥等症状和体征,部分患者无任何症状,甚至以急性脑卒中为首发症状,还可引起心力衰竭和死亡等严重并发症,其致残率和致死率高[1]。既往研究认为,慢性房颤的发生与遗传、离子通道及电生理特性异常等因素有关[2]。但近年来有研究证实,炎症和氧化应激反应参与了慢性房颤的发生和维持过程[3]。本研究观察了炙甘草汤联合美托洛尔对慢性房颤患者的心室率及相关炎性指标的影响,现将结果报道如下。

1　临床资料

1.1　一般资料　选取我院 2012 年 2 月—2015 年 9 月收治的 80 例慢性房颤患者作为研究对象,均符合《内科学》[4]第 7 版中"慢性房颤"的诊断标准;符合《中药新药临床研究指导原则》[5]中气阴两虚证的辨证标准,主要表现为心悸、胸闷痛、面色苍白、短气、头晕、乏力,舌淡红、苔少或薄白,脉细弱或弦细;病程≥3 个月;年龄 18～70 岁。排除初发型房颤或阵发性房颤持续时间 <48 h 者;对美托洛尔或中药过敏者;由急性心肌梗死、心肌炎、甲亢、起搏器置换、心胸外科手术等因素引起的房颤者;合并严重肝肾、造血系统等疾病者;合并严重急性感染、风湿病、心功能Ⅳ级、活动性出血等疾病者。随机分为 2 组:治疗组 40 例,男 22 例,女 18 例;病程 1～4(2.8 ± 1.2)年;NYHA 心功能分级[6]:Ⅰ级 13 例,Ⅱ级 18 例,Ⅲ级 9 例;合并冠心病 15 例,扩张型心肌病 5 例,高血压 13 例,糖尿病 9 例。对照组 40 例,男 23 例,女 17 例;病程 1～4.5(2.6 ± 1.3)年;NYHA 心功能分级:Ⅰ级 11 例,Ⅱ级 21 例,Ⅲ级 7 例;合并冠心病 13 例,扩张型心肌病 4 例,高血压 12 例,糖尿病 10 例。2 组年龄、性别、心功能分级、病程及合并疾病情况比较差异均无统计学意义(P 均 > 0.05),具有可比性。

万方数据

1.2　治疗方法　2 组均给予酒石酸美托洛尔片(阿斯利康制药有限公司生产,国药准字 H32025391)25 mg 口服,2 次/d。治疗组在此基础上加用中药炙甘草汤,组成:炙甘草 12 g、桂枝 9 g、麦冬 12 g、麻仁 12 g、生地 24 g、阿胶 6 g、党参 6 g、生姜 9 g、大枣 6 枚,1 剂/d,加米酒 200 mL 水煎去渣,阿胶烊化,分 3 次温服。2 组疗程均为 8 周。

1.3　观察指标

1.3.1　超声心功能指标　采用三维超声心动图测定 2 组患者治疗前后左室射血分数(LVEF)、心排血量(CO)、心排血指数(CL)、二尖瓣快速充盈期与心房收缩期血流速度比值(E/A)。

1.3.2　中医症候评分　对 2 组治疗前后心悸、胸闷、头晕、乏力症候进行评分,按照病情严重程度分别记为 0 分(无症状)、2 分(症状轻或偶尔出现)、4 分(症状重而间断出现)、6 分(症状显著或持续出现),得分越高中医症候越明显[7]。

1.3.3　心室率和血浆同型半胱氨酸(Hcy)水平　2 组均于治疗前后各佩戴 1 d 动态心电仪,采用专业分析软件测定患者 24 h 心室率。同时于治疗前后清晨采集 2 组空腹静脉血各 5 mL,离心后冷冻保存,采用放射免疫法测定血浆 Hcy 水平,具体操作步骤严格按照使用说明书进行。

1.4　统计学方法　采用 SPSS 15.0 软件包进行数据处理。计量资料采用 $\bar{x} \pm s$ 表示,组间比较采用 t 检验;计数资料比较采用卡方检验。均以 $P < 0.05$ 为差异有统计学意义。

2　结　　果

2.1　2 组治疗前后超声心功能指标比较　2 组治疗前 LVEF、CO、CL 及 E/A 水平比较差异均无统计学意义(P 均 > 0.05);治疗后对照组上述指标均无明显改变(P > 0.05),治疗组上述指标均较治疗前明显改善(P < 0.05),且显著优于同期对照组(P < 0.05)。见表 1。

表1 2组治疗前后超声心功能指标比较($\bar{x}\pm s$)

组别	n	时间	LVEF/%	CO/(L/min)	CL/(L/s·m²)	E/A
治疗组	40	治疗前	46.2±3.5	3.1±0.4	42.5±5.9	0.62±0.11
		治疗后	58.3±2.9①②	6.3±0.6①②	55.6±6.2①②	0.96±0.21①②
对照组	40	治疗前	47.1±3.2	3.1±0.5	42.3±6.1	0.63±0.10
		治疗后	50.1±3.0	4.2±0.8	45.2±5.8	0.71±0.18

注:①与治疗前比较,$P<0.05$;②与对照组比较,$P<0.05$。

表2 2组治疗前后中医症候评分比较($\bar{x}\pm s$,分)

组别	n	时间	心悸	胸闷	头晕	乏力
治疗组	40	治疗前	4.6±1.5	3.7±1.2	2.8±0.9	3.6±1.4
		治疗后	1.3±0.5①②	0.8±0.2①②	0.6±0.2①②	1.2±0.8②
对照组	40	治疗前	4.5±0.8	3.8±1.0	3.0±1.0	3.8±1.2
		治疗后	2.8±0.5①	2.2±0.8①	1.8±0.8①	3.4±0.9②

注:①与治疗前比较,$P<0.05$;②与对照组比较,$P<0.05$。

2.2 2组治疗前后中医症候评分比较 2组治疗前各项中医症候评分比较差异无统计学意义($P>0.05$);治疗后治疗组心悸、胸闷、头晕、乏力评分和对照组心悸、胸闷、头晕评分均明显降低(P均<0.05),且治疗组各项评分均明显低于对照组(P均<0.05)。见表2。

2.3 2组治疗前后心室率和血浆Hcy水平比较 2组治疗前心室率、血浆Hcy水平比较差异均无统计学意义($P>0.05$);治疗后治疗组心室率、血浆Hcy水平和对照组心室率均明显降低($P<0.05$),且治疗组心室率、血浆Hcy水平均明显低于对照组($P<0.05$)。见表3。

表3 2组治疗前后心室率和血浆Hcy水平比较($\bar{x}\pm s$)

组别	n	心室率/(次/min)				血清Hcy水平/(μmol/L)			
		治疗前	治疗后	t	P	治疗前	治疗后	t	P
治疗组	40	120.6±13.5	85.3±11.3	3.821	0.027	24.3±6.8	14.5±5.2	3.152	0.028
对照组	40	118.4±12.7	96.4±12.5	3.185	0.038	23.8±5.9	20.1±5.6	1.350	0.096
t		1.046	-2.804			1.328	-2.620		
P		0.826	0.034			0.495	0.036		

3 讨 论

房颤是临床最常见的心律失常,是以心室电活动极度紊乱而损及机械功能为特点的室上性快速性心律失常,心电图表现为P波消失,代之以频率350~600次/min极不规则的小f波,R-R间期由于房室结隐匿性传导而不规则,心室率快慢不一[8]。房颤发生时,由于心房机械活动不同步、心室反应不规则,会导致心排血量减少,从而增加心血管疾病的病死率。对于房颤的发病机制,现代医学尚未完全清楚,多认为与多发子波折返、局灶驱动等机制有关,近年研究证实,炎症和氧化应激反应可能参与了房颤的发生、发展过程,其对房颤的病理形成过程起着重要作用[9]。

近年来,Hcy作为氧化应激反应的标志性产物备受关注。Hcy是存在于血浆中的一种含硫氨基酸,是甲硫氨酸代谢的中间产物。目前人们已证实Hcy是心脑血管疾病的危险因素,高Hcy血症与冠心病关系密切,Hcy每升高5 μmol/L,男性患冠心病的危险将增加60%,女性将增加80%[10]。同时,高Hcy还可促进氧自由基和过氧化氢的生成,损伤血管内皮,促进动脉平滑肌细胞增生,激活血小板的黏附和聚集,使机体处于一种凝血功能增强的状态,引起动脉血管炎症反应。有研究证实,高Hcy血症与房颤的发生密切相关,前者可在自身氧化过程中形成大量氧化物和自由基,产生继发性氧化应激反应,从而进一步增加心房肌氧负荷参与心室重构;而通过抗氧化剂等治疗可降低血清Hcy水平,从而改善房颤症状,这也间接证实高Hcy对房颤有诱导和促发作用[11]。本研究也显示,2组治疗前血浆Hcy水平均远高于正常值,提示房颤的发生与高Hcy有着一定相关性。

中医认为慢性房颤主要因为阴血不足、血脉无以充盈,加之阳气不足,无力鼓动血脉,脉气不相顺接,故脉结代;阴血不足,心体失养,或心阳虚弱,不能温养心脉,故心动悸。炙甘草汤为中医治疗心动悸、脉结代的代表方剂,其有明确的抗心律失常作用,可明显延长心律失常的潜伏时间、缩短心律失常的维持时间和降低心律失常的病死率[12];其对心肌生理有明显影响,可明显降低大鼠离体右心房肌自律性和左心房肌兴奋性,明显延长左心房肌功能不应期(FRP)[13];可明显降低大鼠右心房自律性,明显抑制肾上腺素诱发的离体豚鼠左心房肌和乳头状肌的自律性和兴奋性[14];此外,炙甘草汤还具有一定抗炎、抗过敏及增强免疫力等作用,这可能也对房颤患者的发生机制产生影响[15]。

本研究发现,治疗后治疗组LVEF、CO、CL及E/A水平均较治疗前明显改善,对照组上述指标均无明显变化;治疗后治疗组心悸、胸闷、头晕、乏力评分和对照组心悸、胸闷、头晕评分均明显降低,且治疗组各项评分均明显低于对照组。治疗后治疗组心室率、血浆Hcy水平和对照组心室率均明显降低,且治疗组心室率、血浆Hcy水平均明显低于对照组。这提示单纯给予美托洛尔可控制慢性房颤心室率,但不能有效改善慢性房颤患者心功能状况,而联用炙甘草汤后可有效改善患者心功能,且可明显改善中医症候,降低血浆Hcy水平,其具体治疗机制还有待进一步研究证实。

[参 考 文 献]

[1] 宋芝萍,顾俊,陈跃光. 阿托伐他汀对慢性心功能不全合并阵发性房颤预后的影响[J]. 国际心血管病杂志,2011,38(2):126-128

[2] 丰明俊,周宏林,陈晓敏. 心房颤动相关离子通道基因变异研究进展[J]. 中国心脏起搏与心电生理杂志,2008,22(6):562-

万方数据

吉西他滨或长春瑞滨联合顺铂治疗转移性三阴性乳腺癌的疗效比较

王洪涛,黄远丽,董 超,熊建群
(华中科技大学同济医学院附属荆州医院,湖北 荆州 434020)

[**摘要**] 目的 比较吉西他滨或长春瑞滨联合顺铂治疗转移性三阴性乳腺癌(TNBC)的有效性和安全性,为临床治疗提供参考依据。方法 选择60例转移性TNBC患者,随机分为吉西他滨组和长春瑞滨组各30例。吉西他滨组给予吉西他滨联合顺铂治疗,长春瑞滨组给予长春瑞滨联合顺铂治疗。治疗4个周期后比较2组临床疗效、毒副反应及近期生存情况。结果 治疗4个周期后,2组总有效率和临床获益率比较差异均无统计学意义(P均>0.05);2组毒副反应发生率比较差异无统计学意义($P>0.05$);2组1,2年生存率比较差异均无统计学意义(P均>0.05)。结论 吉西他滨或长春瑞滨联合顺铂均是治疗转移性TNBC的有效方案,且毒副反应均以轻中度为主。

[**关键词**] 三阴性乳腺癌;吉西他滨;长春瑞滨;顺铂
doi:10.3969/j.issn.1008-8849.2017.02.018
[**中图分类号**] R737.9 [**文献标识码**] B [**文章编号**] 1008-8849(2017)02-0164-03

乳腺癌是临床中极为常见的一种妇科恶性肿瘤,手术切除联合放化疗是治疗乳腺癌的重要措施,可显著改善患者生存率。三阴性乳腺癌(TNBC)指的是人表皮生长因子、孕激素受体和雌激素受体均为阴性的乳腺癌,该类型乳腺癌占全部乳腺癌的20%左右,以年轻女性为多发群体[1]。临床研究显示,三阴性乳腺癌极易出现远处转移,复发率高,患者预后较差[2]。由于TNBC患者体内无人表皮生长因子、孕激素受体和雌激素受体表达,导致靶向治疗及内分泌治疗无效,当紫杉类、蒽环类一线治疗耐药后,目前主要以全身治疗为主[3]。近年来,吉西他滨或者长春瑞滨联合顺铂被广泛用于转移性TNBC的治疗,且取得了一定疗效,不过临床中尚未形成明确标准化疗方案[4]。基于此,本研究分析了吉西他滨或长春瑞滨联合顺铂治疗经紫杉烷类药物和/或蒽环类药化疗后无效的复发转移TNBC的临床疗效,旨在为临床治疗提供参考。现报道如下。

1 临床资料

1.1 一般资料 选择2010年1月—2013年12月本院收治的60例转移性TNBC患者作为研究对象,均经组织病理学检查确诊,且经免疫组化法证实人表皮生长因子、孕激素受体和雌激素受体均为阴性。患者既往未接受过吉西他滨或者长春瑞滨化疗;对紫杉烷类药物和蒽环类药耐药;至少有1个可进行测量的病灶;Kamofsky评分≥60分[5],且生存期在3个月以上。排除标准:严重心、肝、肾等脏器官功能不全或凝血功能障碍者;难耐受化疗者;伴有活动性感染、消化不良或者慢性便秘者。本研究经医院伦理委员会研究同意,并与患者及家属签署知情同意书。根据随机数字表法分为吉西他滨组和长春瑞滨组各30例。2组年龄、TNM分期、发病部位等资料比较差异无统计学意义(P均>0.05),见表1。

1.2 方法 治疗前做好详细体格检查,以确定患者可耐受化疗,并给予5-羟色胺3等预防呕吐。吉西他滨组予吉西他

564
[3] 官媛.炎症和氧化应激标志物在犬心房颤动模型中的变化及意义[J].医学研究杂志,2010,39(8):62-65
[4] 贺立山,翁孝刚.内科学[M].7版.西安:第四军医大学出版社,2008:425-426
[5] 中华人民共和国卫生部.中药新药临床研究指导原则(第1辑)[S].1993:28-31
[6] 上月正博,伊藤修.图解心脏康复指南[M].天津:天津科技翻译出版有限公司,2014:224-226
[7] 国家中医药管理局.中医病证诊断疗效标准[S].南京:南京大学出版社,1994:186
[8] 朱献慧,王诗章,张忆敬.老年退行性瓣膜病常见临床特点及并发心律失常的临床分析[C].国际心脏研究会中国分会学术会议暨中国病理生理学会心血管专业委员会学术会议,2004
[9] 张静,李学文.炎症因素及氧化应激与心房颤动心肌重构的相关性研究[J].山西医科大学,2014,23(5):565-567
[10] 占文明.冠心病患者冠脉病变与血浆同型半胱氨酸水平等易患因素的相关性研究[D].昆明:昆明医科大学,2013
[11] 王三宝.高同型半胱氨酸致动脉粥样硬化的氧化应激机制及Lutein的干预作用研究[D].郑州:郑州大学,2015
[12] 张辰浩.炙甘草汤治疗冠心病PCI术后缓慢性心律失常63例疗效观察[J].现代中西医结合杂志,2013,22(20):2227-2229
[13] 谭现花.炙甘草汤对反复力竭运动大鼠心肌保护作用的研究[J].山东体育科技,2013,35(4):79-81
[14] 龚一萍,沈炜毅.复脉汤对急性心肌缺血大鼠肌钙蛋白及心肌超微结构的影响[J].山西中医学院学报,2014,14(6):15-17
[15] 陈金红.炙甘草汤治疗心律失常研究进展[J].医学美学美容,2015,22(2):767

[**收稿日期**] 2016-06-18

万方数据

DOI:10.19368/j.cnki.2096-1782.2019.09.072

炙甘草汤联合美托洛尔治疗冠心病心律失常的疗效评价

段爱晶
德州市立医院内科，山东德州　253000

[摘要] **目的** 探索炙甘草汤联合美托洛尔治疗冠心病心律失常患者的临床效果。**方法** 选择该院就诊的冠心病心律失常患者作为探究对象(例数:100 例;时间:2017 年 7 月—2018 年 8 月),实施信封随机分组模式进行分组,50 例/组,分别给予美托洛尔、炙甘草汤联合美托洛尔治疗,比较 2 组临床效果、心率、QTd、中医证候评分、不良反应发生情况。**结果** 观察组治疗效果:96.00%、对照组结果:74.00%(χ^2=9.490 2、P=0.002 1); 观察组治疗后心率:(64.25±5.36)次/min、QTd:(42.22±15.24)ms,对照组结果分别为:(75.54±6.62)次/min、(50.25±15.30)ms,(t=9.372 3、P=0.000 1、t=2.629 3、P=0.009 9);观察组治疗后中医证候评分:(4.31±1.28)分,对照组:(6.98±1.55)分,(t=9.392 0、P=0.000 1);观察组不良反应发生率:6.00%,对照组:24.00%(χ^2=6.352 9、P=0.011 7)。**结论** 炙甘草汤联合美托洛尔治疗冠心病心律失常效果显著。

[关键词] 炙甘草汤;美托洛尔;冠心病心律失常;疗效评价
[中图分类号] R256.21　　**[文献标识码]** A　　**[文章编号]** 2096-1782(2019)05(a)-0072-03

Efficacy Evaluation of Zhigancao Decoction Combined with Metoprolol in the Treatment of Patients with Coronary Heart Disease Arrhythmia

DUAN Ai-jing
Department of Internal Medicine, Dezhou Municipal Hospital, Dezhou, Shandong Province, 253000 China

[Abstract] Objective To explore the clinical effects of Zhigancao Decoction combined with metoprolol in the treatment of patients with coronary heart disease and arrhythmia. **Methods** Patients with coronary heart disease arrhythmia who were treated in our hospital were selected as the target of investigation (number of cases: 100 cases; time: July 2017 to August 2018). Envelope random grouping mode was implemented for grouping, 50 cases per group. Metoprolol, Zhigancao Decoction combined with metoprolol were given to the two groups respectively. Clinical effects, heart rate, QTd, TCM syndrome scores, and adverse reactions in two groups were compared. **Results** The therapeutic effect was 96.00% in the observation group, and 74.00% in the control group (χ^2=9.490 2, P=0.002 1). After treatment, the heart rate of the observation group was (64.25±5.36) times/min, QTd was (42.22±15.24) ms. The results in the control group were: (75.54±6.62) times/min, (50.25±15.30) ms,(4t=9.372 3, P=0.000 1, t=2.629 3, P=0.009 9). TCM syndrome scores after treatment in the observation group was (4.31±1.28)points, and (6.98±1.55)points in the control group (t=9.392 0, P=0.000 1). Incidence of adverse reactions was 6.00% in the observation group, and 24.00% in the control group (χ^2=6.352 9, P=0.011 7). **Conclusion** Zhigancao Decoction combined with metoprolol is effective in the treatment of arrhythmia in coronary heart disease.

[Key words] Zhigancao Decoction; Metoprolol; Coronary heart disease arrhythmia; Efficacy evaluation

冠心病心律失常是较为常见的心脏性疾病,其主要以胸闷、气短作为临床表现,病情严重甚至可能还会导致患者出现猝死的现象[1],严重影响我国人口质量,甚至对患者的生存期限会造成一定的缩短现象, 因此,经过临床诊断确诊后需要选择较为合适的方式进行治疗,确保患者的病情稳定,从而为后续治疗奠定基础,。该文研究针对冠心病心律失常患者 (例数:100 例;时间:2017 年 7 月—2018 年 8 月)分别选择不同手段进行治疗,比较不同方式治疗结果的差异性,现报道如下。

1 资料与方法

1.1 一般资料

选择该院就诊的冠心病心律失常患者作为探究对象 (例数:100 例; 时间:2017 年 7 月—2018 年 8 月),实施信封随机分组模式进行分组,50 例/组。本研究符合医学实验伦理学原则,经医院伦理学委员会批准,且 2 组均符合纳入及排除标准,纳入标准:①经临床综合检查,证实为冠心病心律失常;②存在胸闷、气短等临床症状;③对研究知情同意。排除标准:①不配合研究者;②早搏不多者;③器官衰竭或病情危重者;④临床资料不全者。

对照组男性/女性之比=30:20,平均年龄:(62.03±3.74)岁;观察组男性有 31 例,女性占总例数 38.00%(19/50),平均年龄为(61.25±3.87)岁;通过 SPSS21.0 系统分析,上述资料差异无统计学意义(P>0.05)。

1.2 方法

对照组:美托洛尔治疗:口服美托洛尔(珠海经济

[作者简介] 段爱晶(1978-),女,山东德州人,本科,主治医师,研究方向:中西医结合心血管。

特区生物化学制药厂、国药准字 H20057290)，剂量为 25~100 mg，2 次/d；

观察组：炙甘草汤联合美托洛尔治疗：选择炙甘草汤治疗，主药方：10 g 阿胶、10 g 生姜、10 g 桂枝、10 g 大枣、10 g 火麻仁、10 g 人参、15 g 麦门冬、50 g 生地黄、60 g 炙甘草；之后针对患者具体病情进行加减药物治疗，即气滞血瘀者，加以 15 g 川芎、15 g 丹参；气虚者，加以 40 g 黄芪；失眠者，加以 20 g 龙骨；头晕者；加以 10 g 菊花、10 g 川芎；将上述药物进行煎熬，1 剂/d，每剂分早晚服用，同时口服美托洛尔，2 次/d，剂量为 25~100 mg；2 组均治疗 14 d。

1.3 观察指标

观察 2 组临床效果、心率、QTd、中医证候评分、不良反应发生情况。

临床效果：以显效、有效、无效作为评估工具，其中显效表示患者心悸、头晕、乏力等临床症状已经消失，脸色恢复正常，无力感消失；有效表示患者心悸、头晕、乏力等临床症状逐渐消失，但是脸色依旧苍白、无力；无效表示上述指标均未达到标准；

不良反应发生情况：以恶心、便秘、过敏性皮疹作为评估指标。

1.4 统计方法

数据分析采用 SPSS 21.0 统计学软件，计量资料采用($\bar{x}\pm s$)描述，行 t 检验，计数资料采用百分比(%)描述，进行 χ^2 检验，$P<0.05$ 为差异有统计学意义。

2 结果

2.1 临床效果

观察组临床效果高于对照组，差异有统计学意义($P<0.05$)，见表 1。

表 1 2 组临床效果[n(%)]

组别	显效	有效	无效	治疗效果
观察组	41(82.00)	7(14.00)	2(4.00)	48(96.00)
对照组	35(70.00)	2(4.00)	13(26.00)	37(74.00)
χ^2 值				9.490 2
P 值				0.002 1

2.2 心率、QTd

观察组在心率、QTd 中与对照组进行对比($P<0.05$)，见表 2。

表 2 2 组心率、QTd($\bar{x}\pm s$)

组别	治疗前心率(次/min)	治疗后心率(次/min)	治疗前 QTd(ms)	治疗后 QTd(ms)
观察组	97.54±5.69	64.25±5.36	61.22±15.24	42.22±15.24
对照组	97.55±6.36	75.54±6.62	60.26±16.54	50.25±15.30
t 值	0.008 3	9.372 3	0.301 8	2.629 3
P 值	0.993 4	0.000 1	0.763 4	0.009 9

2.3 中医证候评分

观察组中医证候积分优于对照组，差异有统计学意义($P<0.05$)，见表 3。

表 3 2 组中医证候评分[($\bar{x}\pm s$)，分]

组别	治疗前	治疗后
观察组	9.41±0.24	4.31±1.28
对照组	9.24±0.22	6.98±1.55
t 值	3.692 2	9.392 0
P 值	0.000 4	0.000 1

2.4 不良反应发生情况

观察组在恶心、便秘、过敏性皮疹等不良反应发生情况中与对照组进行对比，差异有统计学意义($P<0.05$)，见表 4。

表 4 2 组不良反应发生情况[n(%)]

组别	恶心	便秘	过敏性皮疹	不良反应率
观察组	1(2.00)	1(2.00)	1(2.00)	3(6.00)
对照组	2(4.00)	4(8.00)	6(12.00)	12(24.00)
U 值/χ^2 值		0.55		6.352 9
P 值		0.585		0.011 7

3 讨论

冠心病心律失常是常见疾病，病情加重甚至可能还会诱发患者出现其他类型疾病，影响我国人口质量问题，还会降低患者的生活质量，给患者家庭带来严重的负担。中医学中认为其属于“心悸”“胸痹”范畴[2]，病机为阴阳亏虚、心神不安，故，选择有效方式治疗尤其重要。

美托洛尔是早期临床首选治疗药物，其也是 β 受体阻滞剂，在口服治疗过程中，可消除异位节律[3]，改善缺血区心肌组织的血液供应情况，达到相应的抗心律失常的目的。但是单独使用疗效并不是十分确切，甚至可能还会延误患者的病情，造成病情加重的现象[4]，同时，长期使用美托洛尔进行治疗，会产生较多的不良反应，因此，临床一般选择中西医结合治疗，以期达到较佳的应用效果。

炙甘草汤具有益气滋阴、通阳复脉的功效，主药方由阿胶、生姜、桂枝、大枣、火麻仁、人参、麦门冬、生地黄、炙甘草等药物组成，之后根据患者的具体病情进行加减药物治疗，其中，大枣可脾胃健旺；桂枝、生姜可温心阳、通血脉；阿胶具有滋腻之性；火麻仁、麦门冬可滋养阴血[5]；甘草调和诸药，多种药物联合治疗，可阴阳调和、气血充足，进而恢复心动节律。炙甘草汤具有一定的正性肌力以及纠治室性早搏的作用[6]，同时还能够有效增强心肌抗缺氧能力，进一步改善患者的冠脉血流量。

上述结果中可以看出，观察组治疗效果高于对照组，差异有统计学意义($P<0.05$)，说明，在治疗期间，可以有效改善患者的临床症状[7]，稳定患者的病情，对患者的治愈效果逐渐提升，继而提高患者的生活质量；观察组心率、QTd 改善程度优于对照组，差异有统计学意义($P<0.05$)，提示，美托洛尔联合炙甘草汤治疗，能够对患者的心率、QTd 指标起到一定的改善效

果,逐渐缓解患者的心率[8],使得 QTd 指标能够逐渐趋于正常,为后续治疗奠定相应的基础性[9];观察组中医证候积分优于对照组,差异有统计学意义($P<0.05$),说明,通过美托洛尔联合炙甘草汤治疗,可使得中医证候积分逐渐获得有效改善;观察组不良反应发生例数低于对照组,差异有统计学意义($P<0.05$),提示在治疗期间,安全有效,不良反应发生例数较少,可有效使得患者病情逐渐趋于稳定,对后续治疗以及预后起到较佳的效果。该文研究数据与陈晓琳学者在《中西医结合治疗冠心病心律失常的疗效观察》中的研究数据相似,治疗效果高达 98%,不良反应发生例数低至 2 例,故,该文存在参考价值。

综上所述,针对冠心病心律失常患者选择炙甘草汤联合美托洛尔治疗,效果显著。

[参考文献]

[1] 苗灵娟, 杨永枝, 邢海燕,等. 炙甘草汤联合胺碘酮治疗气阴两虚冠心病室性心律失常疗效观察[J].陕西中医, 2017, 38(2):169-170.

[2] 韩芬, 戴国华, 赵晨, 等. 386 例冠心病心律失常住院患者中医药治疗情况调查分析[J].西部中医药, 2015, 28(3):86-89.

[3] 谢胜明. 胺碘酮联合炙甘草汤加减治疗快速型心律失常临床观察[J].中国中医急症, 2015, 24(7):1256-1257.

[4] 张辰浩. 炙甘草汤治疗冠心病 PCI 术后缓慢性心律失常 63 例疗效观察[J].现代中西医结合杂志, 2013, 22(20):2227-2229.

[5] 崔晓婷. 炙甘草汤联合美托洛尔对慢性房颤患者心室率和血浆 Hcy 水平的影响[J].现代中西医结合杂志, 2017, 26(2):162-164.

[6] 孙艳玲, 王云振. 炙甘草汤治疗气虚血瘀型冠心病合并室性心律失常[J]. 吉林中医药,2017, 37(9):927-930.

[7] 王哲. 炙甘草汤联合美托洛尔片治疗冠状动脉粥样硬化性心脏病心律失常[J].中国中医药现代远程教育, 2016, 14(9):91-93.

[8] 毕颖斐, 毛静远.炙甘草汤在缓慢性心律失常治疗中的应用及探讨[J].天津中医药, 2016, 33(8):465-466.

[9] 陈晓琳.中西医结合治疗冠心病心律失常的疗效观察[J].中国现代药物应用, 2016, 10(9):158-159.

(收稿日期:2019-02-01)

(上接第 71 页)

肿瘤药均有协同作用,具有较好疗效,但容易发生骨髓抑制。洛铂属烷化剂(广义),可通过链间的 AC 和 GG 交联抑制肿瘤基因 C-mye 的表达, 研究显示,将洛铂与吉西他滨联合使用,可进一步促进和惩 DNA,并抑制核核糖苷酸还原酶(RNR)活性,进而促进肿瘤标志物水平降低。

该研究结果显示, 观察组患者疾病缓解率 67.44%明显高于对照组 39.53%, 差异具有统计学意义 ($\chi^2=15.655\ 4$,$P<0.05$)。治疗后观察组患者 NSE、CYFRA21-1、CEA 水平分别为 (20.45±1.16)ng/mL、(4.78±1.23)ng/mL、(28.67±5.92)ng/mL,对照组分别为(24.78±1.67)ng/mL、(8.56±1.67)ng/mL、(42.45±6.34)ng/mL, 观察组患者低于对照组, 差异有统计学意义(t=13.964 0、11.950 9、10.417 3,$P<0.05$)。两组患者不良反应发生率 46.51%vs60.47%对比差异有统计学意义($\chi^2=3.916\ 7$,$P<0.05$)。观察组患者疾病缓解率和 NSE、CYFRA21-1、CEA 水平以及不良反应发生率均优于对照组,学者田春艳[9]等探讨了吉非替尼联合吉西他滨和顺铂化疗方案治疗晚期非小细胞肺癌临床疗效及对血清肿瘤标志物的影响,结果发现研究组经化疗 2 个疗程后临床疗 效客观有效率为 73.58%,对照组为 47.17%,研究组明显高于对照组($\chi^2=8.949$,$P=0.003$) 与该研究结果一致,研究说明吉西他滨联合洛铂化疗可有效提高非小细胞肺癌化疗效果,并降低肿瘤标志物水平,且安全较高,因此值得临床推广。

[参考文献]

[1] 崔丽萍,张红,郑西卫.吉西他滨联合洛铂化疗对非小细胞肺癌患者血清 CEA、CYFRA21-1 及 NSE 指标监测的临床意义研究[J].宁夏医学杂志,2018(11):1-3.

[2] 吴明彬,谢卫民,谢金龙,等.洛铂和顺铂分别联合吉西他滨治疗晚期肺癌的疗效比较[J].保健医学研究与实践,2018,15(4):61-63.

[3] 刘艳萍.洛铂、顺铂联合药物治疗晚期肺癌的疗效对比研究[J].实用癌症杂志,2018,33(1):69-71.

[4] 杜剑平,王峰,江丰收,等.吉西他滨联合洛铂治疗复发性难治性乳腺癌临床疗效观察[J].解放军医药杂志,2017,29(6):58-61.

[5] 赵英男,夏晖.洛铂联合长春瑞滨对 NSCLC 术后辅助化疗疗效及安全性研究[J].实用癌症杂志,2017,32(6):972-974.

[6] 全吉钟,冯燕,王冬旭,等.吉西他滨联合洛铂或顺铂一线治疗老年晚期非小细胞肺癌患者的疗效[J].中国老年学杂志,2014,34(5):1181-1182.

[7] 刘晓文.吉西他滨联合洛铂治疗晚期转移性乳腺癌的临床研究[J].中国现代药物应用,2016,10(4):145-146.

[8] 张健,邵丽华.洛铂联合吉西他滨治疗晚期非小细胞肺癌的临床研究[J].中国实用医药,2016,11(36):16-18.

[9] 田春艳,李馥郁,杨晋,等.吉非替尼联合吉西他滨和顺铂化疗方案治疗晚期非小细胞肺癌临床疗效及对血清肿瘤标志物的影响[J].解放军医药杂志,2018,30(4):25-28.

(收稿日期:2019-02-02)

中药炙甘草汤治疗气虚血瘀型冠心病合并心律失常临床分析

范秀霞
鹿泉区中医院　河北省鹿泉市　050200

【摘　要】目的：调查与研究中药炙甘草汤治疗气虚血瘀冠心病合并心律失常的临床意义。方法：调研2016年3月-2018年3月我院收诊的100例气虚血瘀型冠心病合并心律失常的患者，随机分为采用西药治疗的对照组及中药治疗的观察组；每组50例患者；对照组给予酒石酸美托洛尔缓释片干预治疗；观察组给予西药+中药炙甘草汤干预；对比两组患者经不同治疗后临床疗效及炎症因子变化。结果：经中西药干预后，观察组的显效率、有效率、总有效率均优于对照组，P<0.05差异显著。两组患者经治疗干预后，全部患者于治疗前比较室性早搏、房性早搏、交界性早搏每日发生次数均有所下降；P<0.05。观察组患者治疗后各项早搏每日发生次数、各项炎症因子改善状况均优于对照组，P<0.05差异显著。结论：炙甘草汤在干预气虚血瘀型冠心病合并心律失常患者中取得较好成效，预后取得医师及患者满意，值得医师推广。

【关键词】炙甘草汤；气虚血瘀型；冠心病合并心律失常

冠心病合并心律失常是指冠状动脉血管发生动脉粥样硬化病变导致血管狭窄或阻塞从而导致心肌缺血缺氧等表现，而合并发生不同程度的心律失常可进一步加重冠心病的病情，若未及时治疗严重危害患者的生命。中医研究发现，冠心病合并心律失常主要表现为气虚血瘀型，临床常用酒石酸美托洛尔缓释片等西药治疗，临床中有较好的疗效，但预后差，严重影响患者的日常生活。炙甘草汤是补益剂的一种，益气滋阴，通阳复脉，在气血双补中效果显著[1-2]。为证实中药炙甘草汤治疗气虚血瘀冠心病合并心律失常在临床应用中效用，本院抽调100例气血两虚型冠心病心律失常为本次研究对象，观察西药联合炙甘草汤治疗的效果；报道如下。

1 资料与方法

1.1 一般资料

调研2016年3月~2018年3月我院收诊的100例气虚血瘀型冠心病合并心律失常的患者，随机分为采用西药治疗的对照组及中药治疗的观察组；每组50例患者。其中男性44例，女性56例，年龄：41～74岁，平均年龄：53.72±1.72岁，病程1年~4年。两组患者一般资料比较无明显差异，P>0.05在统计学中无意义。

纳入与排除标准：

（1）全部患者签署知情同意书并本次研究取得我院医学伦理会审批通过。

（2）排除沟通障碍者、癌症患者[3]。

1.2 方法

<< 上接172页

玉竹，性味甘寒，养阴生津，加强生地、麦冬益胃养阴之效，为臣药。冰糖味甘凉，调和诸药为使。诸药合用共凑养阴益胃之效。加减：呕逆，恶心加少量茱萸、代赭石，少量茱萸辛热，降逆止呕。代赭石苦寒，降逆止呕，与益胃汤合用，滋阴养胃，降逆除烦，止呕。大便秘结加何首乌、当归，何首乌甘温，益精润肠。当归辛甘而温，补血活血，润肠通便，与益胃汤合用，养阴益胃，补血益精，润肠通便之功效。皮肤干燥，加桂枝、黄芪、当归，桂枝辛甘，温经通脉，助阳化气，阴无阳不生。黄芪当归补气生血，益胃汤甘凉养阴，阴生阳长，阴阳调和，气血充足，以滋养肌肤脉络，皮肤干燥自愈。汗多气短，气虚加当归，益气健脾。五味子性温味甘酸，生津止渴。饭后脘腹胀满者，加陈皮、神曲理气消食。

脾胃阳虚证：

此证多见于十二指肠溃疡，急慢性胃炎，胃下垂，慢性肠炎，慢性盆腔炎，子宫下垂。

症候：脘腹隐痛，时作时止，喜按喜暖，饮食时稍有不慎，极易呕吐，吐酸腐味，嗳气泛酸，肠鸣便稀，饮食减少，小便减少，精神疲乏，四肢怕冷，面浮肿，白带清稀而腹部隐疼，舌淡苔薄白，脉濡软无力或沉弱。

脾胃虚寒，中阳不振，升降失调，水谷腐熟运化无力，故饮食稍有不慎极易呕吐，时作时止，纳呆。脾虚则运化失职，故浮肿，白带清晰，肠鸣便稀，阳气生外寒，肌肤失于温润，面色晄白，四肢不温，倦怠乏力，喜温畏寒，中焦虚寒，气不化津，津液不能上承，故口干不能欲饮。治宜温中健脾，和胃降逆。理中汤加減

人参、白术、炙甘草、干姜 水煎服或蜜和成丸

本方中凡脾胃虚寒所致吐利，冷痛或脾虚失血，小儿慢惊，胸痹等，证虽不同，其因无不有脾胃阳虚所致，治宜温中祛寒，补益脾胃，内经曰：寒淫所胜，平以辛热。以干姜为君，性味辛热，归脾胃经，温中祛寒，扶阳抑阴，止呕降逆。脾胃湿土，中虚不运，必生寒湿。白术性味苦温，燥湿健脾，是为佐药。病属虚证，虚则补之，人参性味甘温，入脾肺经，补中益气，培补后天之本，气旺而阳复，为臣药。炙甘草性味甘温，补脾益气，调和诸药，为使药。药仅四味，温补并行，药少力专，可使寒气去，阳气复，中气得补，健运有权，中焦虚寒。诸证可除

此方为温补方剂，主要用于脾胃虚寒证，运化升降失职，除见吐利冷痛为主证，畏寒肢冷，舌淡苔白，脉沉迟，或沉细，均可以此方加减用之，此方药性偏于温燥，故外感发热或阴虚者忌用。加减：虚寒甚者加附子、肉桂，性味辛甘味热，助干姜助阳祛寒之效。兼气滞停饮，加枳壳、茯苓，理气化饮。脐上悸动由下焦肾寒上泛，祛白术加肉桂，肉桂辛甘而热，温肾散寒。呕吐多者，祛白术之雍滞加生姜，降逆和胃。泄痢多者，仍用白术健脾止泻。心悸者，为寒湿凌心，加茯苓甘淡利湿宁心。渴不欲饮，为脾不化湿，津液不布，加重白术用量，助脾运化。脾胃虚寒，加附子性味辛热，合理中丸为附子理中丸，温阳驱寒，益气健脾。因风冷想乘，脘腹疼痛，霍乱吐利转筋。

参考文献

[1] 李莉莉，浅析中医脾胃学说[J]. 中医学报，2010.25(01):79-80.
[2] 黄帝内经[M]. 上海:人民出版社，2012.
[3] 中医治法与方剂[M]. 人民卫生出版社，2011.

万方数据

表 1：经干预后患者治疗效果比较 (n/%)

组别	n	显效	有效	无效	总有效率
对照组	50	26（40.00）	16（33.00）	8（16.00）	42（84.00）
观察组	50	28（56.00）	19（38.00）	3（6.00）	47（94.00）[a]

注：与对照组比较，[a]P<0.05，差异显著。

表 2：两组患者各项早搏每日发生次数 [($\bar{x}\pm s$)，次])

组别	n	时间	室性早搏	房性早搏	交界性早搏
对照组	50	治疗前	2259.47±359.54	2035.97±210.98	391.85±66.66
		治疗后	892.84±301.17	352.41±148.94	156.40±37.53
观察组	50	治疗前	2305.16±413.10	2015.76±201.69	4011.65±65.36
		治疗后	614.13±212.45	2074.82±221.26[a]	104.36±35.75[a]

注：与对照组比较，[a]P<0.05，差异显著。

表 3：炎症因子治疗前后的数据统计 ($\bar{x}\pm s$)

组别	n	时间	脑钠肽（ng/ML）	脂联素 (ng/L)	MMP-9(ng/L)
对照组	50	治疗前	912.12±7.23	3.97±0.98	195.85±46.66
		治疗后	200.84±7.17	5.41±0.54	100.40±37.12
观察组	50	治疗前	908.16±8.10	3.76±1.21	201.05±45.36
		治疗后	374.13±9.15[a]	6.82±0.78[a]	128.36±33.20[a]

注：与对照组比较，[a]P<0.05，差异显著。

全部患者给予餐后 10Min 口服美托洛尔缓释片，50mg/ 次，早晚各服用一次。

美托洛尔缓释片生产厂家：阿斯利康制药有限公司；国家准字：J20100098。

观察组的中医炙甘草汤的主要组成成分：干地黄（30 克）炙甘草（12 克） 麦门冬（10 克）大枣（10 枚） 麻仁（10 克） 桂枝（9 克）生姜（9 克）人参（6 克） 阿胶（6 克）；全部中药均为同一组医师煎制而成，200ml/ 剂，分餐前早中晚三次服用[4]。全部患者均服用 2 个疗程（共 6 周）。

1.3 临床疗效观察指标

观察两组患者经不同药物治疗的临床疗效，本次疗效评定参考《中药新药临床研究指导原则》。诊断患者临床表现改善状况（评定内容：各项早搏每日发生次数）。统计与观察两组患者各种炎性因子的改善情况（评定内容：脑钠肽（BNP）、脂联素（APN）、基质金属蛋白酶 -9（MMP-9）等）。

1.4 统计方法

数据用 SPSS 20.0 统计分析，计量资料 ($\bar{x}\pm s$) 表示，t 检验，计数资料（%）表示，X^2 检验。P<0.05 为差异有统计学意义。

2 结果

2.1 治疗的效果比较

经中西药干预后，观察组的显效率、有效率、总有效率均优于对照组，P<0.05 差异显著，详情见表 1。

2.2 两组患者各项早搏每日发生次数

两组患者经治疗干预后，全部患者于治疗前比较室性早搏、房性早搏、交界性早搏每日发生次数均有所下降；P<0.05；观察组患者治疗后各项早搏每日发生次数均优于对照组，P<0.05 差异显著；详情见表 2。

2.3 炎症因子治疗前后的数据统计

治疗后，两组患者的脑钠肽、MMMP-9 均明显降低，脂联素明显升高；P<0.05；与对照组比较，观察组各项炎症因子的数据统计均显著改善，P<0.05 差异显著，详情见表 3。

3 讨论

中医认为冠心病合并心律失常为“胸痹”“心悸”等范畴，临床主要表现为胸痛、心悸、乏力、舌质暗沉等；且气虚血瘀型是最常见的症型[5]。

酒石酸美托洛尔缓释片是 β1 肾上腺素能受体阻滞剂，在治疗心绞痛中有较好成效，可减少心梗发生率，但预后较差[6]。炙甘草汤是《伤寒论》中有益气滋补，通阳复脉的效用，因此又名复脉汤。炙甘草有缓急养心、甘温益气的效用，可扩张冠状动脉，增加心肌收缩，有效调节心脏传导，减少早搏发生；同时炙甘草还可改善心肌缺血、抑制血栓形成，改善患者机体微循环。而干地黄、麻仁、阿胶等有滋阴补血的效用，大枣有补脾养心、补气血、滋阴的效用；生姜、桂枝、人参等可温养通脉，促进气血通畅，改善患者气虚血瘀的状况，从而起到定剂安神的作用[7]。全部配方配伍用药，标本兼制，在临床治疗中有显著疗效[8]。但是，中药配比与煎制需要熟练掌握药效的中医细致配置，因此，医院还需加强对中医的培训，避免用药剂量不足等引发的不良事件。

本次研究表明，经中西药干预后，观察组的显效率、有效率、总有效率均优于对照组，P<0.05 差异显著。两组患者经治疗干预后，全部患者于治疗前比较室性早搏、房性早搏、交界性早搏每日发生次数均有所下降；P<0.05。观察组患者治疗后各项早搏每日发生次数、各项炎症因子改善状况均优于对照组，P<0.05 差异显著。炙甘草汤可有效的预防及干预患者心悸、心痛的临床症状，达到安心复脉、益气养阴、活血化瘀的作用，且预后较好，患者满意度较高。

综上所述：炙甘草汤在干预气虚血瘀型冠心病合并心律失常患者中取得较好成效，预后取得医师及患者满意，值得医师推广。

参考文献

[1] 温万春. 冠心病心律失常患者采用炙甘草汤加减方法治疗的效果评价 [J]. 中西医结合心血管病电子杂志, 2018, 6(01): 143+145.

[2] 董江涛. 加减炙甘草汤治疗冠心病伴发房早及室早疗效观察 [J]. 实用中医药杂志, 2018, 34(05): 516-517.

[3] 黄瑞霞, 何美娟等. 炙甘草汤剂治疗糖尿病性心肌病的疗效及安全性分析 [J]. 糖尿病新世界, 2018, 21(10): 51-52.

[4] 李杰, 王艳. 观察加味炙甘草汤结合常规疗法治疗气血两虚型冠心病心律失常的效果 [J]. 现代医学与健康研究电子杂志, 2018, 2(03): 9-10.

[5] 廉国理. 炙甘草汤用于治疗冠心病心律失常的临床疗效和价值 [J]. 世界最新医学信息文摘, 2018, 18(66): 159+164.

[6] 时敏, 么传为, 睢勇等. 益气活血贴穴位贴敷治疗气虚血瘀型冠心病不稳定型心绞痛临床观察 [J]. 新中医, 2018, 50(09): 182-184.

[7] 孙艳玲, 王云振. 炙甘草汤治疗气虚血瘀型冠心病合并室性心律失常 [J]. 吉林中医药, 2017, 37(09): 927-930.

[8] 俞军山. 炙甘草汤加减治疗冠心病合并心律失常的效果观察 [J]. 中西医结合心血管病电子杂志, 2018, 6(27): 164-165.

万方数据

炙甘草汤联合美托洛尔治疗心律失常的效果研究

管　慧
（贵州省黔南布依族苗族自治州中医医院，　贵州　黔南布依族苗族　558000）

【摘　要】　目的：分析炙甘草汤联合美托洛尔治疗心律失常的效果研究。方法：选取我院2018年2月至2019年12月诊治的80例心律失常患者为对象，根据随机数字法分成对照组和实验组，对照组40例，采取美托洛尔治疗，实验组40例，采取炙甘草汤联合美托洛尔治疗，对比两组患者临床疗效。结果：实验组总有效率（95.00%）高于对照组（70.00%），组间对比，P<0.05。结论：炙甘草汤联合美托洛尔治疗心律失常临床疗效确切，因此值得推广应用。

【关键词】　炙甘草汤；美托洛尔；心律失常；联合用药
【中图分类号】R715　　【文献标志码】A　　【文章编号】1005－0019（2020）19－084－01

Effect of Zhigancao Decoction Combined with Metoprolol on Arrhythmia

Guan Hui
Traditional Chinese Medicine Hospital of Qiannan Buyi and Miao Autonomous Prefecture，guizhou qiannan 558000

Abstract：Objective：To analyze the effect of Zhigancao Decoction combined with metoprolol on arrhythmia. Methods：80 patients with arrhythmia diagnosed and treated in our hospital from February 2018 to December 2019 were selected as the object，and were divided into control group and experimental group according to random number method. 40 patients in control group were treated with metoprolol and 40 patients in experimental group were treated with Zhigancao Decoction combined with metoprolol. The clinical effects of the two groups were compared. Results：the total effective rate of the experimental group（95.00%）was higher than that of the control group（70.00%），and the comparison between groups was P<0.05. Conclusion：Zhigancao Decoction combined with metoprolol has definite clinical effect on arrhythmia，so it is worth popularizing and applying.

Key words：Zhigancao Decoction；Metoprolol；Arrhythmia；Combination medication

前言

心律失常是冠心病常见并发症之一，是由于心脏起搏以及传导系统发生病变，造成病理性异常起搏，从而引发的。有相关研究文献表示[1]，西医可在短期内纠正，但不良反应较多，因此，需找寻有效、安全的治疗方案。此次将我院2018年2月至2019年12月诊治的80例心律失常患者为对象，分析炙甘草汤联合美托洛尔治疗心律失常的效果研究。现报告如下。

1　资料与方法

1.1　一般资料　选取我院2018年2月至2019年12月诊治的80例心律失常患者为对象，根据随机数字法分成对照组和实验组。纳入标准：均符合心律失常诊断标准[2]。排除标准：严重其他原发疾病者；严重外伤史者；中途转院者；精神障碍疾病者；认知功能障碍者。对照组40例：男26例，女14例，年龄50－75岁，平均年龄（67.61±7.36）岁；实验组40例：男27例，女13例，年龄51－76岁，平均年龄（67.55±6.78）岁。此次探讨观察得到本院伦理委员会的审批，两组患者均自愿参与并签署知情同意书。两组患者进行对比，P>0.05，具有可比性。

1.2　方法　对照组采取美托洛尔治疗，美托洛尔初始剂量为6.25毫克/次，每天3次，之后根据病情调整剂量，调整幅度为6.25－12.5毫克/次，每天2次，最大剂量<300－400毫克/日，连续治疗1个月。

实验组采取炙甘草汤联合美托洛尔治疗，美托洛尔用法与对照组一致，炙甘草汤方药组成：党参6克、炙甘草6克、生姜3片、生地20克、大枣10枚、麦冬10克、麻仁10克、阿胶10克、桂枝10克。用水煎服，每天1剂，分早晚服用，连续治疗1个月。

1.3　观察指标及判定标准　对比两组患者临床疗效。疗效标准：（1）显效：临床症状明显改善或消失，心律失常发作次数减少90%以上，心电图检查结果显示正常。（2）有效：临床症状有所改善，心律失常发作次数减少50－89%，心电图检查结果显示正常。（3）无效：临床症状和心电图均无变化，心律失常发作次数减少<50%。

1.4　统计学处理　统计处理软件：SPSS21.0。P<0.05为有统计学意义。

描述性统计：计量资料采用（$\bar{x}\pm s$）描述，t检验，计数资料采用百分比（%）描述，组内及组间比较进行卡方分析。

2　结果

两组患者临床疗效对比　实验组总有效率（95.00%）高于对照组（70.00%），组间对比，P<0.05。见表1。

表1　两组患者临床疗效对比　例（%）

组别(n)	显效	有效	无效	总有效率
对照组(n=40)	17(42.50%)	11(27.50%)	12(30.00%)	28(70.00%)
实验组(n=40)	25(62.50%)	13(32.50%)	2(5.00%)	38(95.00%)
χ^2 值	－	－	－	8.658
P值	－	－	－	P<0.05

3　讨论

心律失常是由于窦房结异常激动，或产生于窦房结外异常激动，导致传导阻滞。缓慢，传导障碍引发心脏搏动异常，是常见的心血管疾病，可单独发病，也可以与其他心血管疾病伴发，极易突然发作导致猝死。

在中医学中，心律失常是属于"心悸"、"心痛"、"胸痹"等范畴，根据《伤寒论》中记载[3]，脉结代，心动悸，认为活血化瘀、益气养阴为主要治疗方法。炙甘草汤方药组成为党参、炙甘草、生姜、生地、大枣、麦冬、麻仁、阿胶、桂枝等中药，具有益气活血化瘀、滋阴养血的功效，据现代药学研究，甘草、麦冬、生地等药材均对心律失常有效，疗效明显，可抗心肌缺氧、缺血，扩张冠动脉，改善血液循环，改善微循环，抗凝效果明显，抑制血栓形成。

此次分析炙甘草汤联合美托洛尔治疗心律失常的效果研究，分析结果显示：实验组总有效率（95.00%）高于对照组（70.00%），组间对比，P<0.05。

综上所述，炙甘草汤联合美托洛尔治疗心律失常临床疗效确切，因此值得推广应用。

参考文献

[1]　李书琴．美托洛尔、炙甘草汤加减联合治疗气阴两虚型冠心病心律失常的效果研究［J］．中西医结合心血管病电子杂志，2019，7（32）：52．
[2]　万志敏，万美萍．美托洛尔联合炙甘草汤加减治疗气阴两虚型冠心病心律失常的效果观察［J］．基层医学论坛，2019，23（19）：2777－2778．
[3]　吴利娜，秦建宁，丁辉．美托洛尔联合炙甘草汤加减治疗气阴两虚型冠心病心律失常疗效及安全性评价［J］．长春中医药大学学报，2018，34（06）：1193－1196．

中风实施中医特色护理对康复效果影响分析

郭会敏
（山东省聊城市冠县中医院，　山东　聊城　252500）

【摘　要】　目的：研究分析中医特色护理对中风患者康复效果的影响。方法：将110例中风患者分成两组，其中常规组55例实施常规西医护理，研究组55例实施中医特色护理。结果：研究组护理总有效率高于常规组；研究组NIHSS评分低于常规组，且QOL评分高于常规组（p<0.05）。结论：相较于常规西医护理而言，中医特色护理措施能够进一步提高中风患者的康复效果，值得在临床中大范围应用。

【关键词】　中医特色护理；中风；康复效果
【中图分类号】R969.4　　【文献标志码】A　　【文章编号】1005－0019（2020）19－084－02

Guo Huimin

Abstract：Objective：To study and analyze the effect of characteristic nursing of traditional Chinese medicine on rehabilitation of stroke patients. Methods：110 patients with stroke were divided into two groups，of which 55 patients in the routine group received routine western medicine nursing and 55 patients in the study group received traditional Chinese medicine characteristic nursing. Results：the total effective rate of nursing in the study group was higher than that in the routine group；the NIHSS score in the study group was lower than that in the routine group，and the QOL score was higher than that in the routine group（P < 0.05）. Conclusion：compared with the conventional western medicine nursing，the characteristic nursing measures of traditional Chinese medicine can further improve the rehabilitation effect of stroke patients，which is worthy of wide application in clinical.

Key words：TCM characteristic nursing；apoplexy；rehabilitation effect

中风是一种发病急骤且死亡率极高的脑血管疾病，极易导致患者偏瘫、失语，降低其生活质量，严重威胁着患者的安全健康，早发现、早治疗有助于患者疾病早日转归，但是临床有效治疗之后的康复指导工作在帮助患者预防后遗症、减少并发症、改善生活质量等方面，意义重大[1－2]。

1　一般资料与方法

1.1　一般资料　本文110例中风患者于2019年02月－－2020年02月纳入。遵循随机、双盲、对照的原则，予以分组探讨，其中一组名为常规组，另一组名为研究组。每个小组分别55例。研究组包括31例男性和24例女性，年龄63－88岁，均龄（74.3±11.5）岁；常规组包括30例男性和25例女性，年龄64－89岁，均龄（74.5±11.7）岁。各组基础资料经临床统计学检验，结果P值>0.05，具有较高的研究价值。

1.2　方法　常规组患者实施常规西医护理：照顾患者日常生活起居；遵医嘱为患者合理用药治疗；密切观察患者病情变化等等。而研究组患者则接受中医特色护理，即（1）药浴。选取红花、黄芪、赤芍以及牡丹皮等可以活血、养血、益气的中药材，首先对患处进行熏蒸，待到药液之后再淋洗或者是浸泡洗，可优化全身气血循环，促使组织细胞再生，旨在驱寒、温经、除湿、祛风。（2）针刺。首先指导患者保持仰卧，主穴为水沟穴、中冲穴，然后辨证加穴，如果患者肢体行动不便，则加髀关穴、足三里；如果患者吞咽异常，则加廉泉穴、百劳穴；如果患者失语，则加亚门穴、廉泉穴；如果患者口喎，则加颊车穴、合谷穴。（3）推拿按摩。推印堂穴，擦腰骶部，揉膀胱经以及太阳穴，向下按压督脉穴。向着血气走向路线或者是经脉循环路线进行按摩，帮助患者活血、行气、通络、舒经，增强患者肌力。（4）艾灸。选取足三里、三阴交、委中穴以及风池穴等穴位并进行艾灸，帮助患者舒经通络，改善血循环。

1.3　观察指标　（1）护理效果评定。NIHSS评分改善70%以上为显效；NIHSS评分改善25%－69%为有效；NIHSS评分改善<25%为无效。总有效率=显效+有效。（2）使用神经功能缺损量表（NIHSS）评估患者神经功能缺损程度，分值越高越严重；使用QOL（生活质量量表）评估患者生活质量，总分60分，总分越高越好。

1.4　数据统计处理　汇总并分析观察主体的研究数据，并利用SPSS22.0软件对研究数据进行统计。用均数±标准差（$\bar{x}\pm s$）表达近似服从正态分布的计量资料；用例数或构成比（%）表达计数资料或等级资料。当P值低于0.05时，说明存在较高的检验价值。

2　结果

2.1　研究组与常规组护理总有效率对比见表1

表1　研究组与常规组护理总有效率对比

小组	病例	显效	有效	无效	总体疗效
研究组	55	32	21	2	96.36%
常规组	55	28	17	10	81.82%
X^2	－				10.484
p	－				<0.05

2.2　研究组与常规组护理前后NIHSS评分以及QOL评分对比见表2

（下转第085页）

万方数据

炙甘草汤加减治疗室性早搏的临床研究*

何德英 靳文学
(重庆市中医院,重庆 400021)

中图分类号:R541.7+3 文献标志码:B 文章编号:1004-745X(2015)11-2035-03
doi:10.3969/j.issn.1004-745X.2015.11.054

【摘要】 **目的** 观察炙甘草汤加减联合美托洛尔治疗室性早搏的临床疗效。**方法** 将 156 例患者按随机数字表法分为两组,各 78 例。对照组采用酒石酸美托洛尔治疗,观察组在对照组基础上加用炙甘草汤加减治疗,两组治疗 4 周。**结果** 观察组总有效率为 91.03%,高于对照组的 73.08%($P<0.05$)。心电图、24 h 动态心电图室性早搏减少均明显优于对照组($P<0.05$)。**结论** 炙甘草汤加减配合酒石酸美托洛尔治疗室性早搏疗效显著,无明显不良反应。
【关键词】 炙甘草汤 室性早搏 气阴两虚 张仲景

Effect of *Zhigangcao Decoction* on Ventricular Premature Beat *HE Deying, JIN Wenxue. Chongqing Traditional Chinese Medicine Hospital, Chongqing* 400021, *China*

【Abstract】 **Objective:** To observe the effect of *Zhigancao Decoction* plus metoprolol on ventricular premature beat. **Methods:** 156 cases were randomly divided into the control group and the treatment group with 78 patients in each group. Two groups were treated by the metoprolol tartrate. The treatment group was added the *Zhigancao Decoction*. After 4 weeks, some indicies were compared in twoa groups. **Results:** The total effectvie effective rate in the treatment group with 91.03% was higher significantly than that in the control group with 73.08% ($P<0.05$). Some indices in the treatment group were superior to those in the control group including the electrocardiogram and 24 h dynamic electrocardiogram($P<0.05$). **Conclusion:** It is effective of *Zhigancao Decoction* plus metoprolol on ventricular premature beat which is no obvious adverse reaction.
【Key words】 *Zhigancao Decoction*; Ventricular premature; Qiyinliangxue syndrome; *ZHANG zhongjing*

室性早搏作为心律失常之一,可见于器质性心脏病患者,也可见于无器质性心脏病的健康者。室性早搏属于中医学“心悸”“怔忡”范畴。西医治疗室性早搏能取得一定疗效,但副作用较多,应用受到限制。近年来,笔者应用炙甘草汤加减联合酒石酸美托洛尔治疗室性早搏获得了良好疗效。现报告如下。

1 资料与方法

1.1 病例选择 中医诊断标准参考《中药新药临床研究指导原则》[1]。排除急性心肌梗死、低血压、急性心力衰竭、严重心力衰竭(心功能Ⅲ~Ⅳ级)、Ⅱ度或Ⅲ度房室传导阻滞严重、心动过缓、心源性休克、肝肾功能不全者。

1.2 临床资料 选取重庆市中医院心内科 2012 年 1 月至 2015 年 3 月诊断为室性早搏的患者 156 例,均符合病例选择标准。按照随机数字表法分为两组,各 78 例。其中男性 80 例,女性 76 例;年龄 18~75 岁;病程 6~15 个月;其中合并病毒性心肌炎 50 例,冠状动脉粥样硬化性心脏病 48 例,高血压性心脏病 14 例、肺源性心脏病 11 例,风湿性心脏病者 7 例,甲状腺功能亢进 6 例,单纯功能性室性早搏 20 例。两组临床资料差异无统计学意义($P>0.05$)。

1.3 治疗方法 两组均停用其他治疗心律失常药物,在常规治疗的基础上对照组服用酒石酸美托洛尔(阿斯利康制药),每次 12.5 mg,每日 1 次;观察组在对照组基础上加用炙甘草汤加减方:炙甘草 18 g,生晒参 10 g,桂枝 15 g,麦冬 15 g,生地黄 30 g,阿胶 6 g(烊化),麻子仁 15 g,葛根 15 g,黄精 15 g,甘松 15 g,苦参 15 g,生姜 9 g,大枣 10 枚。随证加减:偏重阴虚者生地黄大剂量使用,加黄精等,若生地黄大剂量使用,可佐以炒山楂防止泄泻;兼阳虚者加肉桂、制附子等;兼痰湿者加瓜蒌、法半夏、茯苓等;兼心悸、失眠严重者加酸枣仁、五味子、煅牡蛎、煅龙骨、柏子仁、百合;兼肝气郁结者加柴胡、香附、郁金。水煎服,取汁 300 mL,每日 1 剂,分 3 次温服。两组均治疗 4 周。

1.4 观察指标 两组服药前后均进行血常规、尿常规、肝肾功能、心电图或 24 h 动态心电图检查,观察患者心率、血压及服用药物后有无药物不良反应。

1.5 疗效标准 显效:服药后主要症状消失,如心悸、乏力、气短等,发作次数减少≥90%,24 h 动态心电图、心电图检查基本正常。有效:服药后主要症状减轻,发作次数减少≥50%但<90%,24 h 动态心电图、心电图

*基金项目:重庆市科学技术委员会课题(cstc2015jcsf0066,cstc2014jcyjA10065);重庆市卫计委课题(ZY201402049)

检查结果有明显改善。无效：服药后主要症状没有改善或者加重，发作次数减少<50%，24 h 动态心电图、心电图等检查结果无变化或加重[1]。

1.6　统计学处理　采用 SPSS22.0 统计软件处理。计数资料采用 χ^2 检验。$P<0.05$ 为差异有统计学意义。

2　结　果

2.1　两组临床疗效比较　见表 1。结果示治疗组总有效率高于对照组（$P<0.05$）。

表 1　两组临床疗效比较[n(%)]

组 别	n	显效	有效	无效	总有效
观察组	78	36(46.15)	35(44.87)	7(8.97)	71(91.03)$^{\triangle}$
对照组	78	29(37.18)	28(35.90)	21(26.92)	57(73.08)

与对照组比较，$^{\triangle}P<0.05$。

2.2　两组心电图检测结果比较　治疗前观察组、对照组治疗前 24 h 动态心电图室性早搏次分别为（2368±246）次、（2344±230）次，治疗后两组分别为（418±183）次、（862±216）次。两组均较治疗前改善，治疗组改善优于对照组（$P<0.05$）。

3　讨　论

从中医学角度来看，室性早搏临床表现以心悸、胸闷、乏力、头晕、脉结代为主要症状。姬水英等[2]研究认为凡证见心动悸，脉结代，证属阴血不足、阳气虚弱的室性早搏患者中医辨证属于炙甘草汤证，使用炙甘草汤随证加减均能取得显著效果，特别是以病毒性心肌炎后遗症之室性早搏，效果更为显著。炙甘草汤，又名“复脉汤”，是《伤寒论》治疗心动悸、脉结代的名方。方中以炙甘草为君，“通血脉，利气血”，补中益气，而昌气血生化之源，缓急养心，其为治疗心中动悸，脉结代之要药，为复脉之本；人参大补元气，大枣益脾养心，三药合用，补益脾胃，为复脉之源。生地黄滋阴养血，宜用大剂，以复心阴养心脉；阿胶滋阴补血润燥，为养阴清热要药，麦冬清心润肺益胃，麻子滋阴养液润燥。生地黄、阿胶、麦冬、麻仁 4 药合用，养心阴、滋心血，共同起到充养血脉，滋阴养血之功效，并能充养脉之实体。桂枝配生姜有温经通脉、振奋心阳之功效。诸药合用则阴阳得平，复脉而宁心定悸。现代药理研究表明，炙甘草汤中所含有的甘草酸、麦冬总皂苷和人参总皂苷 3 种主要成分能显著降低实验大鼠离体心房肌自律性和兴奋性，能够明显延长实验大鼠离体心房肌功能的不应期，并且对肾上腺素诱发实验大鼠离体乳头肌自律性和心律失常还有一定的抑制作用[3]。有研究表明，炙甘草可有效拮抗乙酰胆碱和阻滞钠通道，麦冬具有抗心律失常、营养心肌、增加冠状动脉血流量、改善左心室功能的作用，人参、生地黄有抗心肌缺血，增强心肌收缩力和增加心输出量的作用[4]。中药黄精具有降脂、降压、抗动脉粥样硬化，以及增加冠脉血流量的作用。中药甘松中分离出的的缬草酮具有细胞膜的稳定作用，缬草酮可与心肌细胞膜上离子通道中的特异蛋白相合，起到抑制 Na 离子内流和促进 K 离子外流，从而起到降低心肌细胞自律性的作用；同时还能延长心房肌、心室肌和传导系统的动作电位时间，打断心脏折返激动，从而起到有效治疗各种心律失常的作用[5]。中药葛根分离出的有效成分葛根素具有扩张冠状动脉血管，改善心肌供血，可明显减少因心肌缺血导致的乳酸升高，还可减慢心率，降低心肌耗氧量，增强心肌收缩力。另有研究证实，葛根素具有 β 受体拮抗剂样作用，对心脏 β1 受体具有一定选择性，有抗心律失常等多种作用[6]。苦参性苦寒，归心肝经，现代药理研究[6]表明其具有抗心律失常和抗心肌缺血作用，苦参碱对乌头碱、哇巴因、氯化钡、肾上腺素诱发的实验性心律失常模型有一定的对抗作用，苦参水煎醇沉液、苦参总碱能减轻脑垂体后叶素引起的急性心肌缺血，抑制 ST 段下降和 T 波低平等心电图缺血性变化。故葛根素及苦参均不失为治疗冠心病室性早搏的良药。张晓云等[7]发现炙甘草汤可拮抗因缺血缺氧诱发的心律失常。陈兰英等[3]研究发现，炙甘草汤能提高大鼠的免疫功能。甄耀辉等[8]发现炙甘草汤可延长室性早搏的潜伏期。王宏利等[9]研究发现炙甘草汤对提高心肌细胞的耐缺血缺氧能力有显著作用，可以增加心脏冠脉血流量。

笔者体会，炙甘草汤使用时应当注意以下几点。1）药物用量：炙甘草和生地黄用量宜大，但生地黄用量宜大于炙甘草，如生地黄大剂量使用，可佐以山楂防止泄泻。方中以炙甘草为主药，其通经脉、利血气的作用为历代医家及现代药理研究所证实，用量宜大，至少用到 18 g，可逐渐加量至 20~30 g 方能获得比较好的抗心律失常疗效，若其量较大时，可倍加茯苓，既可宁心，亦可避其肿满；生地黄用量宜大，可用至 50~120 g/d。2）加减化裁。心律失常有“脉结代，心动悸”之症，但患者有阴阳虚损之不同，故宜加减化裁，方能获得满意疗效。若属气阴两虚者可原方照用；若属心气虚明显，见心悸短气、动则加剧、脉缓弱而结代者可加重人参，协同炙甘草为主药，亦可加黄芪；若偏阴虚显著者，见心悸而烦、口干难寐、舌尖红赤、脉细弱而结代者，则重用生地黄与炙甘草为主，而减少或去姜、桂；若兼心肾阳虚者，适当增加桂姜剂量，减少或去地、麦胶麻等阴柔之品，以温通心阳，复其血脉。心悸甚者可加茯苓，并重用其量，亦可加酸枣仁、远志；气滞血瘀，心绞痛者，可加延胡索、丹参、郁金、降香等。冠心病所致脉结代、心动悸，以气虚为主，兼气滞血瘀，痰浊阻遏者，可用本方加黄芪，合瓜蒌薤白半夏汤、血府逐淤汤加减；由风心病所致的脉结代、属于气阴两虚者可用本方，选加驱风湿药，加防己、秦艽、茯苓、白术等；3）把握适应症。柯

雪凡认为，炙甘草汤对于不同类型的心律不齐疗效迥异，其最佳疗效为病毒性心肌炎后遗症之心律不齐，风湿性心脏病之心律不齐则次之，而对于冠心病及高血压所致的心律不齐效果不明显[10]。此经验有助理解《伤寒论》炙甘草汤条文及其应用。刘丛群[11]认为，炙甘草汤对早搏疗效甚好，尤其是室性早搏。本研究中，笔者用炙甘草汤加减治疗室性早搏78例，其中病毒性心肌炎致室性早搏25例，占观察病例较大达30%以上，获得了满意疗效。4)煎服法：加清酒煎服，尤以米酒、黄酒为佳。清酒辛热，以行药力，可畅通血道，温阳复脉。但若器质性心脏病患者不耐酒力，需慎用。

炙甘草汤是治疗心律失常的经典方剂，其治疗效果显著，被广泛应用于对心律失常的治疗。本研究结果显示，炙甘草汤加减配合酒石酸美托洛尔治疗室性早搏总有效率明显优于单纯西医治疗，治疗后24 h动态心电图心律失常室性早搏次数低于单纯西医治疗，且无明显不良反应，较之单纯使用西药治疗心律失常，起到了减毒增效的作用，弥补了西药治疗心律失常之疗效有限而副作用较大的不足。

参考文献

[1] 中华人民共和国卫生部. 中药新药临床研究指导原则[M]. 北京：中国医药科技出版社，2002：68-69.
[2] 姬水英，牛菲，马娟娟，等. 炙甘草汤临床新用[J]. 云南中医中药杂志，2012，33(9)：32-33.
[3] 陈兰英，陈奇，刘荣华，等. 炙甘草汤主要有效成分对心肌电生理特性的影响[J]. 中草药，2001，32(22)：134
[4] 昆山，王秀琴，董一凡. 现代临床中药学[M]. 北京：中国中医药出版社，1998：417，545.
[5] 张文蘅，陈虎彪. 甘松属植物化学和药理学研究进展[J]. 中国野生植物资源，1999，18(3)：11-14.
[6] 郑虎占，董泽宏，佘靖. 中药现代研究与应用[J]. 北京：学苑出版社，1998：4308.
[7] 张晓云，胥爱文，马建伟，等. 炙甘草汤对缺血缺氧诱发豚鼠心律失常的电生理效应[J]. 中国新药与临床药理，2008，19(2)，103-104.
[8] 甄耀辉，邓启华. 炙甘草汤对大鼠室性早搏拮抗作用实验研究[J]. 实用中医药杂志，2009，25(5)：280-281.
[9] 王宏利. 炙甘草汤治疗冠心病心律失常48例[J]. 光明中医，2009，24(12)：2275.
[10] 柯雪帆.《伤寒论》研究札记[J]. 中医药学刊，2002，20(4)：398.
[11] 刘丛群，孙强，衣春风. 炙甘草汤治疗心律失常的临床体会[J]. 中国现代药物应用，2009，3(5)：181-182.

(收稿日期 2015-06-29)

参附注射液治疗慢性肾脏病并发心衰的临床观察

王洪霞 张晶晶 徐建龙 梁 莹 杨丛旭△
(中国中医科学院西苑医院，北京 100091)

中图分类号：R692 文献标志码：B 文章编号：1004-745X(2015)11-2037-03
doi：10.3969/j.issn.1004-745X.2015.11.055

【摘要】**目的** 观察参附注射液治疗慢性肾脏病并发心衰患者的临床疗效。**方法** 将58例患者按照随机数字表法分为治疗组30例，对照组28例。对照组采用西医常规对症治疗，治疗组在对照组基础上加用参附注射液静滴。两组均以14 d为1个疗程。观察患者肾功能和心功能及心室结构的变化。**结果** 治疗后两组患者的心功能，左室舒张功能、收缩功能，肾功能均得到好转，治疗组改善更明显($P<0.05$)。**结论** 参附注射液能够改善慢性肾脏病患者并发的心衰，并改善肾功能。

【关键词】 参附注射液 慢性肾脏病 心衰

慢性肾脏病患者中，约有30.7%患有心力衰竭1年以上，其中40%是以心力衰竭为初发疾病[1]。同时心力衰竭也往往伴有肾脏损伤，病死率随着肾脏损害程度逐渐增加，肾功能恶化又是心力衰竭的独立危险因素[2]。肾功能恶化和心力衰竭相互影响，形成恶性循环，最终导致心脏和肾脏功能的共同损害，使得临床治疗常常顾此失彼，导致了存在心脏病和肾脏病患者的病死率要远远高于罹患一种疾病患者。因此有效控制心衰、改善心衰患者预后，是降低死亡率的主要环节。笔者采用参附注射液治疗慢性肾脏病并发心衰患者，取得了良好疗效。现报告如下。

1 资料与方法

1.1 病例选择 纳入标准：符合2002年美国肾脏病基金会的肾脏病预后质量倡议(KDOQI)工作组提出的

△通信作者(电子邮箱：yang_congxu@163.com)

广州中医药大学学位论文原创性声明

本人郑重声明：所呈交的学位论文，是个人在导师的指导下，独立进行研究工作所取得的成果。除文中已经特别加以注明引用的内容外，本论文不含任何其他个人或集体已经发表或撰写过的作品成果。对本文的研究做出重要贡献的个人和集体，均已在文中以明确方式标明并致谢。本人完全意识到本声明的法律结果由本人承担。

学位论文作者签名：黄勉婷

日 期：2016 年 05 月 12 日

关于学位论文使用授权的声明

本人完全了解广州中医药大学有关保留使用学位论文的规定，同意学校保留或向国家有关部门机构送交论文的复印件和电子版，允许被查阅和借阅。本人授权广州中医药大学可以将本学位论文的全部或部分内容编入有关数据库进行检索，可以采用影印、缩印或其他复印手段保存和汇编本学位论文。

（保密论文在解密后应遵守此规定）

论文作者签名：黄勉婷　论文导师签名：[illegible]

日 期：2016 年 05 月 12 日

万方数据

Y3144467

摘　要

目的：

本课题通过应用中医及现代医学理论，探索经方炙甘草汤加减联用倍他乐克治疗功能性频发性室性早搏（气阴两虚型）的疗效及安全性,为功能性频发性室性早搏的临床用药提供新的依据、探索新的思路。

方法：

从 2015 年 2 月 10 日至 2016 年 1 月 9 日新会中医院住院及门诊病人中筛选出的频发性功能性室性早搏患者（气阴两虚型）60 例，采用严格的前瞻性、随机、双盲、对照研究方法，随机分成对照组（30 例）、治疗组（30 例），对照组采用倍他乐克(每次 12.5mg,每日两次)治疗；治疗组加用炙甘草汤加减，疗程均为 4 周。观察治疗前后的临床症状（心悸、胸闷、气短等）、早搏次数的变化，并监测安全性指标。最后，通过运用 SPSS17.0 软件得出研究数据的统计分析结果。

成果：

1、综合疗效比较：治疗 4 周后，治疗组总有效率为 76.67%，对照组为 50.00%，两组对比差异明显(P<0.05)，综合疗效前者优于后者。

2、室性早搏次数变化比较：治疗后两组早搏次数均减少，治疗组减少更明显。

3、中医症状疗效比较：观察中医症状（心悸、胸闷、气短、五心烦热、乏力、自汗、盗汗、口渴），经计分、统计得出两组治疗后中医症状积分均下降(P<0.05)，治疗组下降更明显（P<0.05）；中医症状总有效率分别为 86.67%和 66.67%，且差异具有统计学意义(P<0.05)。

4、安全性方面：两组皆未出现与试验药物明显相关的不良反应及实验室指标异常。

结论：

与单纯应用西药治疗对比，经方炙甘草汤加减合倍他乐克可更有效改善功能性频发性室性早搏患者的中医临床症状，减少早搏的发作次数。但确切的结论尚有待更大规模的随机、对照前瞻性研究结果证实。

关键词： 炙甘草汤加减；倍他乐克；功能性室性早搏；临床研究

万方数据

Study on Modified Zhigancao Decoction combined with Metoprolol in patients with functional ventricular premature

Speciality:Clinical intergrated Chinese and western mdeicine
Author:Huang Mianting
Tutor:Luo Zhihua

Abstract

Objective:

The purpose of this study is to explore the clinical efficacy and safety of classical prescription Modified Zhigancao Decoction combined with Metoprolol in the treatment of functional frequent ventricular premature of Type Qi and Yin Deficiency and provide new basis and explore new ideas for clinical administration by use of Traditional Chinese Medicine(TCM) theory and modern medicine theory.

Methods:

Performed a strict foresighted double-blind and random control study of 60 patients with functional ventricular premature beats of Type Qi and Yin Deficiency.The patients were chose from outpatients or inpatients of Xinhui Hospital of Traditional Chinese Medicine over the period from February 10,2015 to January 9,2016,and divided randomly into treatment group(n=30) and control group(n=30).Patients in control group were received Metoprolol(12.5mg each time, 2 times one day),and patients in treatment group were received Modified Zhigancao Decoction more,treatment durations for both groups were 4 weeks.Observed the change on clinical symptom(palpitation、chest distress and dyspnea etc.),premature ventricular contractions beats and monitor security indicators before and after treatment.At last,the results of statistic analysis for the data of study were given by using SPSS17.0 software.

Results:

1.The comparison of comprehensive efficacy:The total effective rate was 76.67% in the treatment group and 50.00% in the control groups after treatment of 4 weeks.The difference between the two groups was significant($P<0.05$).The

万方数据

comprehensive efficacy of the treatment group was better than that in control group.

2. The comparison of premature ventricular contractions: Two groups of patients with the premature ventricular contractions of 24 hours were both reduced after treatment (P<0.05), the treatment group was better than that in control group (P<0.05).

3. The comparison of clinical symptoms efficacy: Observed the TCM symptoms (palpitation, chest distress, dyspnea, dysphoria in chestpalms-soles fatigue, spontaneous perspiration, night sweat, thirst), Two groups of patients with clinical symptoms scores were both decreased by compuring the TCM symptom scores and conducting the statistic analysis, but the change was significantly greater in the treatment group (P<0.05). The total effective rate of TCM symptom was 88.67% in the treatment group and 66.67% in the control groups and the interblock cure effective diversity had statistical significance (P<0.05).

4. Safety: Two groups had not been found adverse reactions associated with test drugs and laboratory abnormalities.

Conclusion:

Contrast with the application by the western medicine only, classical prescription Modified Zhigancao Decoction combined with metoprolol could improve the patients' TCM clinical symptoms and Holter monitoring performance, reduce the frequency of premature ventricular beats happen more effectively. But the exact results have yet to be confirmed by a larger randomized, controlled prospective study.

Keywords: Modified Zhigancao Decoction ;Metoprolol ;
Functional Ventricular Premature ;Clinical research

万方数据

目　　录

广州中医药大学学位论文原创性声明
摘　　要……I
Abstract……II
目　　录……IV
引　　言……1
第一章　文献研究……3
　第一节　现代医学研究概况……3
　　一、流行病学……3
　　二、病因及发病机制……3
　　三、西医治疗……5
　　四、西医治疗的局限性……6
　第二节　中医研究概况……7
　　一、病名探讨……7
　　二、病因病机……7
　　三、辨证分型研究……9
　　四、中医治疗……9
第二章　临床研究……11
　第一节　研究对象……11
　　一、病例来源……11
　　二、诊断标准……11
　　三、纳入标准……13
　　四、排除标准……14
　　五、病例剔除、脱落及中断标准……14
　第二节　研究方法……14
　　一、样本量估算……14
　　二、分组方法……15
　　三、治疗方案……15
　　四、观察指标方法……15
　　五、剔除、脱落情况及统计处理……16
　第三节　研究结果……16
　　一、一般资料比较……16

万方数据

二、实验观察项目比较......17
三、安全性评价......19
第三节 讨论......20
一、功能性频发性室性早搏概况......20
二、炙甘草汤中医研究现状......20
三、炙甘草汤现代药理研究......21
四、炙甘草汤治疗心律失常应用举隅......22
五、总结及展望......22
结　语......23
参考文献......24
附　录......28
致　谢......30

万方数据

引　言

社会发展日新月异，竞争越显激烈，加之人们不良生活、饮食习惯等诸多因素影响，使心律失常发病率日趋上升，威胁民众的身体健康。而室性早搏(premature ventricular contraction，PVC)是常见的一种心律失常。健康人的一生中，最少发作一次PVC的比率，常规心电图检查是0.78%~6%，而在Holter心电图中较高，为39%~80%[1]。PVC可出现在伴或不伴有器质性心脏病的患者，部分频发PVC却无症状，而有些患者临床症状虽明显，异位搏动却较少。一般认为，频发PVC可增加交感神经的兴奋性，改变心室的不应期，发生心悸、胸闷、焦虑等不适；而伴有器质性心脏病的频发PVC危害较大，如有冠心病、心肌肥厚等基础疾病的患者可诱发心绞痛、室速，可降低心功能，引起心衰，升高心脏性猝死发病率，严重影响生命健康。相关资料显示，约占83%的猝死病人曾发作过PVC[2]。既往认为，功能性PVC是“良性”的，无须特殊处理，而近些年有关研究表明，频发的功能性PVC并非完全良性，亦须特殊干预[3]。上世纪，心动过速性心肌病虽是公认的可逆性疾病，但孤立性频发性PVC导致的左室功能紊乱在之前并未报道。而自2000年，S Chugh[4]等研究报导了一例患者，因患频发性PVC、且有扩张型心肌病，经过导管射频消融术治疗之后，心功能恢复正常，初次证实频发PVC（尤其是>10000次/24h）可引起心动过速性心肌病，临床表现类似扩张型心肌病，从而揭示在一些有心衰的患者，室早性心肌病可能为左室功能紊乱的潜在性可逆病因。综上所述，积极治疗功能性频发性PVC，对于改善病人症状，预防心动过速性心肌病有重要意义。

西医治疗非器质性 PVC 主要针对症状，通过改善生活方式，应用镇静剂或抗心律失常药，或行射频消融术。但药物治疗部分患者疗效不明显，停用药物后极易复发，副作用明显，可导致心律失常[5]、增加死亡率的危险性。临床上抗心律失常药物（antiarrhythmic drug,AAD）不断推陈出新，却尚无一种有效、安全、副作用少、应用广泛的 AAD，而射频消融术疗效虽明确，患者常因其有创性及高昂的价格而首选药物治疗。

历代名家及专著里无 PVC 病名相关记载，参照其临床特征，可归属于心悸、怔忡、心动悸等范畴。古今医家对上述疾病的病因、发病机制及辨证论治的研究，总结本病病位主要在心，与肝、脾、肺、肾脏腑失调相关，病机多相杂出现，总以气阴两虚为主的本虚标实病证多见。

综合以上几点，本病中医治疗予补气滋阴为法，予仲景名方炙甘草汤加减，联合西药倍他乐克治疗，观察其临床疗效及各项检查指标变化，通过统计分析，研究中西合璧对于改善 PVC 的临床症状、减少早搏次数的有效性，并分析其安全性。

万方数据

万方数据

第一章 文献研究

第一节 现代医学研究概况

一、流行病学

室性早搏简称室早，为心脏希氏束分支以下部位提早激动，从而诱发心室提前除极，可单独、或成对、成串出现。病情轻微者无临床症状,部分有心悸、胸闷不适，较为严重者可触发室速、室扑或室颤，有致命的危险性。功能性 PVC 指心脏结构、功能均正常的患者出现频发 PVC[6]，多见于青壮年，且女性发病率高于男性，多数器质性疾病所致 PVC 在运动后早搏次数增加，症状加重，而功能性 PVC 在运动后反而症状减轻，早搏次数减少，且于安静状态下易于诱发。上世纪 80~90 年代，诸多研究表明，功能性 PVC（PVC 次数>10000 次/24 小时）患者在随防 10 年后，如患者心脏结构、功能没有异常变化，无须特殊处理，为良性 PVC。1998 年，Duffee 等[7]报道 10 例原发性抗张型心肌病患者，射血功能低下伴频发 PVC，经药物治疗，PVC 次数较前减少，心功能显著改善，证实心肌病为 PVC 所致。2000 年 S Chugh 等[4]报导频发 PVC 患者经射频消融术治疗后，成功逆转心肌病，证实频发 PVC 可导致心动过速性心肌病。功能性 PVC 并非如此良性，即使无明显临床症状，亦须特殊干预。

二、病因及发病机制

（一）病因

PVC 可见于正常人，或伴有器质性心脏病者,如冠心病、心肌病、甲状腺功能亢进性心脏病、二尖瓣脱垂、病毒性心肌炎等。

功能性 PVC 多出现在青壮年，常因情绪激动、失眠、抑郁、过度疲劳、过量使用酒、茶、烟等诱发，也可无明显诱因。

各类药物如洋地黄、拟交感神经类药物、麻醉药等可诱发 PVC。此外，非洋地化的患者应用利尿剂时，应密切监测电解质，避免低钾血症，诱发 PVC。亦有报道称左氧氟沙星、利福平、复方甘草酸苷、罗格列酮[8]、复方樟柳碱注射液[9]可诱发 PVC。

而心内直视手术[10]、拔牙[11]等诱发 PVC 亦有报道。此外，缺钾、缺镁、低血糖、自主神经功能紊乱也可引起 PVC。Dogan M[12]等研究发现，女性在排卵期雌二醇达到峰值，PVC 次数下降，提示雌激素差异可能对室性心律失常有影响。

（二）发病机制

包括下列五个研究靶点：触发激动、折返现象、异常自律性、平行收缩、机械反馈学说[13]，此外还有其他研究靶点。大部分人误认为自律性 PVC 最为多见，而近年来相关数据显示，触发激动及折返现象较为多发[14]。

1. 触发激动

触发激动于1973年由Cranefield提出[15]，指激动动作电位开始，至复极后的时期，

万方数据

膜电位自发的一种振荡性的除极活动，当这种除极活动达到一定程度时，便可引起发复激动，导致期前收缩。包括早期后除极、延迟后除极，实验标本中证明，其诱发因素有缺氧、高浓度儿茶酚胺、洋地黄中毒等。早期后除极发生在动作电位复极的早期，即2位相或3位相，而延迟后除极则发生于复极的4位相。Zimik S[16]等通过心脏组织模型结合计算机模拟研究发现，早期后除极引起PVC的因素包括以下几个方面：早期后除极细胞的复极储备能力；产生早期后除极细胞的大小；早期后除极细胞之间的耦合强度；早期后除极细胞群中纤维母细胞的存在。尽管复极储备能力降低对早期后除极的形成及促进PVC来说是必要的，但极低的复极储备能力亦可使早期后除极电位降低，从而延缓PVC的产生时间或避免PVC。

2. 折返现象

折返是由于心脏内某部位产生的激动经过环形通路折回后再次兴奋该部位所致。任何部位的折返的产生，皆因该部位存在着不同特性的两条或多条径路所致。折返为心律失常较为重要的发病机制。陈小贞等[17]以冠状动脉灌注兔左室楔形组织标本，记录其内、外膜两侧心肌细胞的动作电位及跨壁心电图变化，发现缓慢的电紧张扩布可再一次激活兴奋性已恢复的外膜侧心肌细胞，从而产生动作电位，并激活内膜侧心肌，诱发折返激动。

3. 异常自律性

自律性指心肌自发地产生除极性激动。而心肌缺血缺氧、电解质紊乱等多种因素影响皆能使原先没有自律性的心肌细胞自律性明显增强，从而影响心律，诱发早搏。

4. 平行收缩

室早型平行收缩，系指心脏内存在两个节奏位点：窦房结和心室，两者各自阻止对方的冲动传入自身的起搏中心，竞争性地控制心室。目前其发生机制尚不明确，通常认为与传入阻滞、传出阻滞相关，传入阻滞系指冲动无法传导，或延迟进入起搏点。传出阻滞指平行收缩的起搏点传出遇到阻滞，无法激动心肌细胞。

5. 机械反馈学说

机械反馈学说认为心肌细胞存在着牵张激动通道，增加左室血容量可激活更多的牵张通道，因而心脏扩大患者更易发生室性心律失常。

6. 频发 PCV 引起心肌病的可能发病机制

目前频发PVC的动物模型不易建立，故频发PCV诱发心肌病的机制尚不明确，部分研究显示其可能为心动过速性心肌病的一个类型，亦可能与心动过速性心肌病机制不同。其发病可能的机制如下：

（1）PVC 起源部位众多，如心尖、基底部。相关资料显示，左束支传导阻滞形态的 PVC 引起心动过速性心肌病可能与长期快速性右室心尖部起搏，导致心肌重构，心脏

万方数据

扩大，进而导致心功能下降有关。

（2）频发 PVC 引起心室失去同时收缩能力，尤其当 QRS 波完全为异常激动时，亦可引起心脏扩大，进一步使心功能下降。

（3）此外，虽不等同于心动过速性心肌病，但两者并非完全不同，如长期频发 PVC 引起心动过速可极大消耗心肌细胞能量储备，冠脉血液流量减少，以致心肌细胞钙操纵异常，细胞外基质重构，此外，β 肾上腺素反应能力下降亦可引起心功能不全。此在心动过速性心肌病中也有类似表现。

（4）极少部分学者研究表示，PVC 诱导心肌病可能是心动过缓导致的左室功能不全所致。因为每一个室早都不能产生有效的心脏搏出，早搏时患者脉搏明显减弱，对于每天有 30000~40000 次室早的患者来说，相当于减少于三分之一的心脏有效搏出，患者的平均心率实际低于正常人，这与严重的心动过缓导致的后果相似，如病态窦房结综合征或房室传导阻滞。

（5）而频发 PVC 后的长代偿间歇，亦损害血流动力学，使心室容量负荷增加，激活交感神经系统，与“不规则心律诱发的心肌病”相似。

三、西医治疗

首先评估有否并存结构性心脏病(Structural Heart Disease，SHD)。对于心律失常伴有临床症状者，常规 12 导联心电图有助于评估是否存在心脏瘢痕（Q 波或碎裂 QRS 波群）、Q-T 间期、心室肥大和 SHD 的其他迹象。Xu W[18]等研究了 112 名 PVC 患者 7 天动态心电图、NT-proBNP 检查，发现起源于左室乳头肌的肌束的 PVC 相对良性，而源于三尖瓣环的 PVC 可能引起心脏功能障碍。而症状性 PVC 患者、频发 PVC（负荷＞10%）患者或疑有 SHD 的患者，须加测超声心动图以了解、评估心室结构和功能。

（一）无症状的 PVC 患者

大多数无症状的功能性 PVC 患者预后良好，多数患者左室射血功能无异常，可通过改变生活方式、随访、定期评估心脏功能处理。对于检查提示阶段性左心室收缩功能下降、心室容量增加的患者，无症状的频发 PVC 需要治疗，首先寻找病因，处理原发病。

长期频发性 PVC(特别是每日大于 1 万次早搏)患者可致心肌病，称为室性早搏性心肌病，其临床特征类似扩心病，应作心脏彩超、动态心电图随访观察，了解 PVC 负荷随时间而波动。特别是对于 24 小时 PVC≥5%的患者，尽管无症状，也需密切监测，防止 PVC 引起心肌病。相关专家表示，尽管部分 PVC 患者没有出现左室收缩功能降低，依然可引起微小的血流动力学及相关生化指标改变。相关研究表明，无论患者有无临床症状，射频消融术治疗皆可获得收益[19]。

（二）有症状患者

万方数据

1. 消除病因、诱因

对于有症状的频发 PVC 患者，如伴 SHD，应尽快寻找病因、诱因，对因处理，评估其风险其致命性；如无 SHD，应当先排除生理学因素如过度通气、排除饮酒或咖啡等刺激性食物、失眠，如去除以上因素仍不能恢复且严重影响日常生活、工作才需正规治疗。治疗以消除症状为目的。

2. 药物治疗

通过解除患者顾虑，排除生理性病因后，如仍有焦躁、不安情绪，可试用镇静剂或少量β受体阻滞剂。因心律明显增快的患者交感神经兴奋性多有或轻或重的增强，所以在治疗中，如无明显的禁忌症，应当优先考虑使用β受体阻滞剂[20]，为PVC的一线治疗药物，但因可能导致房室传导阻滞、诱发哮喘等不良反应，进一步限制了它的临床应用范围。如患者不适宜运用β受体阻滞剂，且无结构性心脏病，可考虑非二氢吡啶类钙通道阻滞剂作为β受体阻滞剂的替代药物，为Ⅱb类推荐[21]。Ⅰa类AAD容易诱发威胁生命的心律失常，增加SHD的病死率，现临床基本不用。Ⅰb类副作用相对少见，相对安全，临床可适当选用。ⅠC类临床效果较好，却因负性肌力及负性传导作用而使临床应用受到诸多限制。Ⅲ类AAD如胺碘酮治疗PVC疗效最好，但可引起甲功、肝功异常，肺纤维化等副作用，目前多用于伴有SHD、心功能不全的患者，注意应于治疗前评估相关实验室指标，指导临床应用。

相关试验表明：他汀类可减少伴有缺血性心脏病患者的 PVC 病发率，而伴有心功能不全者如合用 AECI 或 ARB，亦能减少 PVC 的病发率[22]。近年来，AAD 临床研究进展不大，采用非 AAD 治疗或协助治疗已成为心律失常研究中的一大闪光点。

3. 射频消融术

导管消融疗效好、安全性高、创伤较小，近 20 年来发展迅猛，是唯一可根治 PVC 的治疗手段。2006 年 ACC/AHA/ESC 室性心律失常和猝死预防指南[23]中对 PVC 的射频消融治疗有明确的建议：Ⅱa 类推荐，频发的症状性单形性 PVC，药物治疗无效，药物不耐受，或者不愿意长期接受药物治疗；Ⅱb 类推荐，无症状性 PVC 患者行导管消融术以预防或治疗心动过速性心肌病；Ⅲ类推荐，不推荐射频消融治疗无症状的相对非频发性 PVC。消融手术成功后，左室射血分数的改善率在 47%到 100%之间。影响消融成功率的因素主要有早搏的起源部位、发作频率，发作时血流动力学状况及术者的经验等，而多项研究表明消融成功高，7 成以上患者 PVC 消除，且并发症较少[24]。

四、西医治疗的局限性

（一）1989 年著名的 CAST 研究[25]首次提到 AAD 可导致心律失常。而目前应用的诸多 AAD 几乎均可导致心律失常，其发病率与检测手段密切相关，Holter 心电图监测其致心律失常发病率约为 10%，而经电生理检测统计结果得出，其发病率竟达 20%以

万方数据

上[26]。

（二）AAD 疗效有限，总有效率只有 30%～60%[27]。长期使用易产生耐药物，且药物反复更替作用不明显，症状反复。

（三）某些 PVC 患者基础心率偏慢，使 AAD 的运用明显受限。

（四）射频消融术：亦非绝对安全，因其可能复发、引起其他致命性并发症，加之价格高昂，诸多因素令多数患者望而生畏。据研究分析，射频消融术的复发率为 2.8%，另 0.9%的人出现相关并发症，而严重并发症如脑梗死、脑出血、心梗等也会导致死亡，死亡率在 0.1%左右[23]。

第二节　中医研究概况

一、病名探讨

根据PVC相关临床表现，可归属于心悸、怔忡、心动悸范畴。心悸病名首次出现在《黄帝内经》，《素问·遗篇本病论》论及天气变化可致心悸不安、惊恐畏惧情绪。《素问》曰:“参伍不调者病”；同时对典型的心悸脉象作了详细的记录，首次论述严重心悸时脉搏的变化与病情预后紧密相关：如果人一呼脉四动以上，而一吸脉四动以上，为死证，而脉搏停止跳动，或忽快忽慢，亦为死证。

东汉张仲景《伤寒杂病论》初次提出“惊悸”、“心中悸”、“心动悸”等相关病名，开创心悸辨证论治先河。“寸口脉动而弱,动则为惊,弱则为悸”，动为动荡不宁，弱为脉濡弱不畅，提出惊则脉动,虚则心悸。“伤寒二三日，心中悸而烦者，小建中汤主之”，指出气血双亏而致悸，复被邪扰而致烦。“伤寒脉结代，心动悸，炙甘草汤主之”，提出治疗心悸名方“炙甘草汤”。

宋朝严用和初次提出心悸另一相关病名“怔忡”，并对惊悸、怔忡作了细致分析,指出前者属情志疾病，多由心虚胆怯所致,常因忽受惊吓诱发；后者多因心血耗损所致。元代朱震亨则提出两者鉴别要点为：前者为偶尔发作，后者却无时不作。

及至现代，心悸病名趋于统一、规范，近代医家将“惊悸”、“怔忡”归于心悸一病中论述，认为惊悸发病较轻，多属实证，可自行缓解；而怔忡相对严重，大多为虚证，且不能自行缓解。且惊悸发病时间较长时，可发为怔忡，两者是心悸发病中的不同病程阶段。

二、病因病机

（一）病因

1. 外感邪气

《内经》云：“风、寒、湿三气杂至，合而为痹”，痹证如发病时间长，加之外来邪气内侵于心，以致心之血脉运行受到阻碍，引起心悸，提出风、寒、湿三气为心悸的多发外在原因。此外，温疫、火毒皆能耗伤心气、心阴，使心失濡养，导致心悸。

万方数据

2. 体虚劳倦

体质虚弱，禀赋不足；或过度劳累，忧虑少寐；或脾虚、纳呆致气血生化不足，或致心失所养，从而导致心悸。

3. 七情内伤

平素胆怯，忽受惊吓，易扰乱心神，而致心悸。甚至惊慌不能自已，渐至无所惊吓，怔忡亦可自发。忧虑伤脾，或郁而化火，灼津生痰，痰火扰心，以致心神不宁，发为心悸。此外长期抑郁、大怒可引起气机逆乱冲心，发为心悸。

4. 药食不当

《扁鹊心书》提出服用冰凉、坚硬之品可伤及脾胃，引起阴阳气机运动失调，郁于中焦，发为怔忡。平素嗜食肥甘厚腻，或药物过量如附子、细辛等，或误服有毒药物，可损伤脾胃，脾胃运化失职，可耗伤心阴、损耗心气，而致心悸。

（二）病机

心悸病机较为复杂，古今医家对其研究呈百花齐放、百家争鸣之态。

《素问》云："诸病胕肿，疼酸惊骇，皆属于火"，说明火热可致惊骇。《伤寒论》谓："伤寒脉结代,心动悸,炙甘草汤主之。"由汤药反推证型,此处"心动悸"病位当在心，病机为阴、阳、气、血皆虚,导致心失濡养，脉搏跳动无力。唐代孙思邈《千余要方》指出"阳气外击,阴气内伤,伤则寒,寒则虚,虚则惊掣心悸"，提出心悸因虚致病的病机。元代朱丹溪指出心悸当"责之虚与痰"，强调血虚、痰火为怔忡致病的根源。明末《景岳全书·怔忡惊恐》曰"虚微动亦微，虚甚动亦甚"，认为怔忡为阴虚劳损所致。清代王清任《医林改错》记载，如有心悸，运用归脾方、安神方无明显效果，予血府逐瘀汤可有百发百中之效，王清任认为瘀血内阻可致心悸。

现代医家多数认为心悸病位在心，与五脏相关，为本虚标实之证，本虚为阴、阳、气、血虚损，气为阳，血为阴，心气及心阳推动血液运行，心血心阴则润养心神，有一方虚则心失濡养，心搏失去跳动而为病，病机总以气阴两虚多见，而标实为气滞、血瘀、痰浊、水饮夹杂。

邓铁涛教授[28]认为正虚（心气虚、心阴虚）为心悸之本，痰瘀互结为标，认为心悸当从心、脾两脏立论治疗。杨思进教授[29]则认为，心悸病机为虚实两端，虚者多以气虚、阳虚为主，而实则为痰湿、瘀血兼夹错杂。刘建和教授[30]提出心悸病机多为少阳不和，且有宿根（宿痰）。华明珍教授[31]认为心肾同归少阴，心位于上而肾位于下，心阳依赖肾阳温煦，而心阴亦靠肾阴滋养，滋养心阴，需滋肾阴，温补心阳，需补肾阳，主张心悸当从肾论治。邓悦教授[32]认为心悸的病机有虚实两方面，虚证为气血阴阳不足，心失濡养，发为心悸，实证则为气滞血瘀、痰浊水饮、痰火扰心以致心神不宁。申啸笑[33]将岭南相关医案中符合心悸诊断标准的病案资料录入，使用SPSS统计分

万方数据

析进行数据分析，研究得出岭南心悸患者主要病机为气血阴阳不足，虚证为主，其中气虚最多见，而痰湿、火热、瘀血是导致心悸的重要因素。

三、辨证分型研究

刘贯龙[34]等根据《伤寒杂病论》涉及心悸的20余条条文，结合古今文献，阐述了张仲景对心悸的脏腑辨证，将其分为：心阳气虚证、心阴阳两虚证、心脾气血两虚证、心脾阳虚证、心肾阳虚证、饮停胃肠证、肝气郁滞证、肺气郁闭证。姜瑞雪[35]等对中医药治疗心悸的相关报道进行统计研究，从中筛选出辨证分型中较公认的证型，提取证素,将出现频次较高的证素依据中医基础理论归纳总结，得出心悸以虚证为主，其比例由高到低依次为：气虚、阴虚、阳虚、血虚；实证主要为瘀血、火热、痰浊、水饮。总结归纳其证型分类为：气滞血瘀、痰阻心脉、心火炽盛、心血虚、水气凌心、以及心之气、血、阴、阳虚损共八类。周仲瑛教授强调脏腑整体辨证，认为心悸不单指心之为病，肝、脾胃、肺、肾功能失调皆可致心悸[36]；并在其主编的《中医内科学》中将心悸分为心虚胆怯证、心血不足证、阴虚火旺证、心阳不振证、水饮凌心证、瘀阻心脉证、痰火扰心证[37]。

四、中医治疗

目前，大量的临床研究结果显示：现代医家以中医整体观念、辨证论治为法，运用经方汤剂、中成药口服制剂或注射剂治疗PVC，取得良好效果，不良反应少，临床应用前景广阔。其中汤剂以经方为主的加减治疗得到诸多医家青睐，如炙甘草汤、酸枣仁汤；中成药服用方便，疗效确切，以稳心颗粒为首的口服中成药受人瞩目，稳心颗粒用于治疗心律失常因其广谱、有效、安全的特点，受到广泛认可，中国专家共识已于2015年正式发布；相对于口服药，目前中成药针剂用于治疗PVC目前临床应用较少。

（一）汤剂

宋丹[38]等收集60例PVC（气阴两虚型）患者，观察组运用仲景经方酸枣仁汤加减治疗，对照组予美托洛尔口服，结果显示治疗组总有效率(90.00%)优于对照组(70.00%)，经统计学检测具有显著性差异。李志勇[39]运用加味桂枝龙骨牡蛎汤治疗PVC（心脾两虚夹血瘀证），观察组（56例）、对照组（30例）均予常规西药治疗，结果显示中医症状及早搏次数的改善，观察组均优于对照组，且具有统计学差异。张曦光[40]等以六经辨证为法，观察小柴胡汤加味治疗PVC五个中医证型共70例患者的临床疗效，最低有效率为40%（气血阴阳俱虚型），最高有效率为63.16%（痰饮内停型）。

（二）中成药

沈安明[41]等在基础用药之上，运用健心胶囊治疗冠心病PVC，对照组予心律平口服，两组均为42例，结果提示两组均可减少PVC次数，缓解临床症状。邹建刚[42]等

万方数据

研究参松养心胶囊治疗 PVC，治疗组（108 例）总有效率为 88.9%，优于对照组（56 例）的 71.4%，两者差异有显著意义。付华宾[43]运用稳心颗粒治疗 PVC，研究显示在改善症状方面，治疗组（56 例，口服稳心颗粒）有效率为 91.1%，明显优于对照组（56 例，口服心律平）的 78.6%，差异有统计学意义(P＜0.05)。

（三）针剂

尹克春[44]等在常规西药治疗的基础之上，以益气活血为治则，运用当归Ⅱ号注射液注射穴位治疗冠心病 PVC，三组各 30 例，在治疗心悸方面，实验组总有效率（76.7%）优于 0.9%氯化钠对照组（53.3%），但劣于胺碘酮对照组（83.3%），且具有统计学差异。李锐[45]等观察 42 例老年性 PVC 患者，在常规药物治疗基础上，观察组加用丹红注射液静脉滴注，结果显示观察组有效率（90.48%）优于对照组（64.29%），且具有统计学意义。史丰奇[46]运用丹参酮ⅡA 磺酸钠注射液治疗 PVC，对照组予胺碘酮口服，两组各 18 例，结果显示，治疗组有效率为 94.4%，对照组有效率为 83.3%，具有统计学差异。

万方数据

第二章 临床研究

第一节 研究对象

一、病例来源

此次研究病例全部来源于新会中医院心内科门诊或住院病人（住院病人不少于总例数 1/3），西医诊断为功能性频发性 PVC，中医诊断为心悸，辨证符合气阴两虚型的患者。

二、诊断标准

（一）西医诊断标准

1. 基本诊断标准：

根据最新版《实用内科学》（第 14 版）[13]中有关 PVC 的诊断标准，规定如下：

（1）症状：可无症状，亦可有心悸或心跳暂停感。频发 PVC 可引起胸闷、气短、全身无力多种症状。有些患者因频发 PVC 而致失眠，形成恶性循环增加 PVC 发作次数及频率，影响生活质量。

（2）体征：听诊可闻及早搏，早搏后有较长的代偿间歇。期前收缩的第一心音多增强，第二心音多减弱或消失。期前收缩呈二联或三联律时，可听到每两或三次心搏后有长间歇。期前收缩插入两次正常心搏间，可表现为三次心搏连续。脉搏触诊可发现间歇性脉搏缺如。

（3）心电图：①QRS 波提早出现，形态宽大畸形；②时限多数>0.12s，T 波与 QRS 主波方向相反，其前无 P 波。③发生于束支近端处的室性期前收缩，其 QRS 波群可不增宽。④PVC 后多数有完全性代偿间歇。⑤基本心率较慢时，室性期前收缩可插入两次窦性心搏之间，形成插入型 PVC。⑥偶见室性期前收缩逆传至心房的逆行 P′ 波，常出现于 PVC 的 ST 段上。

2. PVC 严重程度的分级标准

根据第六版《黄宛临床心电图学》[47]论述的 Lown 分级方法，结合 Holter 心电图将 PVC 分为 6 个级别：

0 级：无期前收缩。

Ⅰ级：偶发，每小时少于 30 次或每分钟少于 1 次。

Ⅱ级：频发，每小时多于 30 次或每分钟多于 6 次。

Ⅲ级：多源性室性期前收缩。

ⅣA 级：成对的室性期前收缩，反复出现。

ⅣB 级：成串的室性期前收缩（三个或三个以上室性期前收缩）反复出现。

Ⅴ级：期前收缩的 R 波落在前一个窦性激动的 T 波上。

（二）中医诊断标准

万方数据

1. 心悸诊断标准

参考中医药管理局发布的《中医病症诊断疗效标准》[48]里关于心悸的诊断标准制定如下：

（1）自觉心慌不安，心跳剧烈，神情紧张，不能自主，心搏或快速，或缓慢或心跳过重，忽跳忽止，呈阵发性或持续不止。

（2）伴有胸闷不适，易激动，心烦，少寐多汗，乏力，头晕等。

（3）发作常由情志刺激、劳倦过度、饮酒饱食等诱发。

（4）可见有脉象数、疾、促、结、代、沉、迟等变化。

（5）测量血压、胸片 X 线检查、心电图等有利于诊断。

2. 辨证分型标准

参照 2002 年《中药新药临床研究指导原则》[49]选择心悸气阴两虚证型为观察证型，辨证标准如下：

心悸气阴两虚证：

主症：心悸，自觉心中悸动不安，不能自主。

兼症：神疲、气短、胸闷或痛、自汗或盗汗、五心烦热、口渴等。

舌象：舌淡红或红，苔白或少苔。

脉象：脉沉细弱、细数或脉结代。

须具备主症、兼症至少 2 项标准，结合舌、脉象综合分析，才能确诊。

3. 中医症状量化评分标准

参考 2002 年《中药新药临床研究指导原则》[49]规定的相关标准，结合实际临床经验，制定中医评分标准如下：

（1）心悸：

0 分——无症状

2 分——偶发心悸，可自行缓解

4 分——心悸频发，不影响正常生活、工作

6 分——心悸持续发作，影响正常生活、工作

（2）胸闷：

0 分——无症状

1 分——偶发胸闷，可自行缓解

2 分——胸闷频发，不影响正常生活、工作

3 分——胸闷持续发作，影响正常生活、工作

（3）气短：

0 分——无症状

万方数据

1 分——活动后气短

2 分——稍活动后气短

3 分——不活动时仍有气短、喘促感

（4）乏力：

0 分——无症状

1 分——重度活动即感乏力

2 分——中度活动即感乏力

3 分——轻度活动即感乏力

（5）五心烦热：

0 分——无

1 分——轻度，不影响正常生活、工作

2 分——中度，轻微影响生活、工作

3 分——重度，严重影响生活、工作

（6）自汗：

0 分——无症状

1 分——平素皮肤微潮，轻微活动则汗出

2 分——平素皮肤潮湿，轻微活动汗出明显

3 分——平素即汗出，轻微活动则出汗，如水渍状

（7）盗汗：

0 分——无症状

1 分——睡觉时轻微出汗，部位以头部多发，偶然出现

2 分——睡觉中度出汗，胸背部潮湿，多次出现

3 分——睡觉时出现明显大汗，全身潮湿，时常发生

（8）口渴：

0 分——无症状

1 分——偶有渴感

2 分——经常口渴，饮水后可缓解

3 分——整日口渴，饮水后不缓解

舌脉象只记录不计分。

三、纳入标准

（一）符合 PVC 诊断标准，Holter 心电图提示早搏次数>720 次，Lown 分级属 II 或III级；无 SHD，且心电图无其他异常表现的正常人的 PVC。

（二）符合中医心悸诊断标准和气阴两虚证辨证标准。

万方数据

（三）年龄在18岁至60岁之间。

（四）近期使用过其他药物者，至少应停药2周。

（五）自愿参予试验且配合医师合作，签订同意书。

四、排除标准

出现以下任一表现，不能入选本试验：

（一）因冠心病、高血压、心肌炎、心肌病、心脏瓣膜病等SHD引起的PVC。

（二）年龄小于18岁、60岁以上，过敏体质或对两种及两种以上食物或药物过敏者。

（三）急性冠脉综合征患者或伴有其他心律失常如房室传导阻滞（Ⅱ度Ⅱ型以上）、持续性室速、或者非持续性室速伴有快速心室率和血流动力学紊乱者、窦性心率低于50次/分、持续性房颤者。

（四）NYHA心功能Ⅲ~Ⅳ级，且心脏彩超提示EF<45%者。

（五）LOWN分级为Ⅰ级或ⅣA级以上患者。

（六）孕妇、哺乳期，或一年以内有生育意者，或育龄期内未采取有效避孕措施患者。

（七）肝功、肾功异常（超过正常值），既往曾患支气管哮喘病史。

（八）甲功异常，伴有电解质紊乱、糖尿病，或呼吸、血液、消化、免疫、肿瘤、其他内分泌疾病等可能导致心律失常疾病者。

（九）长期大量饮用咖啡、浓茶；吸烟及饮酒者；长期失眠、焦虑、处于抑郁状态者。

（十）近三个月参予过其他药物试验者。

五、病例剔除、脱落及中断标准

（一）剔除标准：不应纳入而被误纳入者，包括误诊、未遵医嘱进行规范药物治疗、未按要求进行相关辅助检查、私自加用其他可能影响药物效果或安全性的药物。剔除比例一般不超过总纳入例数的5%。

（二）脱落标准：脱落病例指已入组但中途因某些原因退出试验者。所有试验者均可随时退出本试验。脱落病例包括患者要求中止试验；试验过程出现明显不良反应。研究人员须详细记录患者脱落的原因，且控制脱落率不超过10%。

（三）中断标准：指因受试药物以外的原因，终止本次研究的受试者。包括：研究过程中出现明显不良反应需采取应急处理者；突发意外或伤亡等患者。中断病例不能参与统计分析，且中断病例须补齐。

第二节　研究方法

一、样本量估算

按临床研究中随机对照试验需要的最小样本量进行估算，两组共需60例，按1：1比例进行随机分组。估算失访率为10%左右，因调整的样本含量=估算的样本含量/（1-失访率）^{2}，计算得出调整后的样本量为74例。

万方数据

二、分组方法

将符合纳入标准，且经排除标准严格筛选的患者，运用 SPSS17.0 中的完全随机设计程序将其平均分为两组。将随机卡片用不透明小信封密封，根据患者就诊顺序进行编辑，根据相应序号拆开信封，依据卡片的随机数字，根据其组别进行治疗。随机数字、随机分组结果、信封制作均由非课题组成员负责，保证随机分配隐匿性、正确性。

三、治疗方案

（一）治疗组：中药合西药治疗。

中药：炙甘草 30g，党参 15g，麦冬 12g，熟地黄 12g，酸枣仁 12g，生姜 9g，桂枝 9g，大枣 12g，阿胶 9g（烊化）。

服法：统一由医院煎煮室煎煮，将组方中药煎服 100ml，复煎 100ml，温服，每日一剂，早晚两次，饭后服用。

西药：倍他乐克（规格：25mg/片；生产厂家：阿斯利康；国药准字 H32025391），用量：12.5mg，每日 2 次，口服。

（二）对照组：单纯西药治疗。

（三）疗程：以四周为一个疗程。

四、观察指标方法

（一）基本资料：姓名、性别、年龄、民族、职业、住址等。

（二）一般临床资料：病史、既往史、个人史、婚育史、家族史、以往用药情况等。

（三）安全性指标：生命体征，血、尿、大便常规，心、肝、肾功能，分别于治疗前后各行一次检查，试验过程随时记录不良反应。

（四）疗效观察指标

1. 详细观察询问主要及次要临床症状（心悸、胸闷、气短、乏力、五心烦热、自汗、盗汗、口渴），有无不良反应，行一般体格检查及心脏体格检查。

2. 治疗前后各做动态心电图、常规心电图一次，观察 PVC 改善情况，治疗过程如有变化随时行常规心电图检查。

3. 根据问诊结果详细评定中医症状评分，治疗前后各行一次。

（五）疗效与安全性评价

按照 2002 年《中药新药临床研究指导原则》[49]关于 PVC 疗效标准，结合临床经验，制定如下：

1. 综合疗效判定标准：

（1）显效：心悸完全消失，动态心电图显著改善，PVC 次数较治疗之前减少 90%以上，或室早 Lown 分级指标升高 2 级；

万方数据

（2）有效：心悸基本改善，动态心电图轻微改善，PVC 次数减少 50%~90%，或室早 Lown 分级提高 1 级；

（3）无效：心悸无明显变化或加重，动态心电图 PVC 次数减少<50%，或加重。

2. PVC 次数的疗效判定标准：

（1）显效：动态心电图显著改善,PVC 次数较治疗前减少 90%以上。

（2）有效：动态心电图稍改善,PVC 次数较治疗前减少 50%~90%。

（3）无效：PVC 次数较治疗前减少未达 50%。

3. 中医临床证候疗效的判定标准:

采用尼莫地平法，即证候疗效率=（治疗前积分－治疗后积分)÷治疗前积分×100%。

显效：临床症状、体征较前有所改善，症候疗效率≥70%。

有效：临床症状、体征较前改善，症候疗效率≥30%，＜70%。

无效：临床症状、体征较前无改善，症候疗效率＜30%。

4. 安全性评价

1 级：安全，无任何不良反应，相关实验室检查无明显异常。

2 级：相对安全，出现轻度不良反应，无须行特殊处理，可继续服用试验药物，实验室安全性指标检查无明显异常。

3 级：有安全性问题，出现中度的不良反应，或安全指标实验室检查轻度异常，经治疗后能继续服药。

4 级：服药期间出现明显不良反应须立即中断试验者，或实验室安全性指标检查有明显异常结果。

五、剔除、脱落情况及统计处理

因患者自行要求中止试验、私自服用其他可能影响研究结果的药品等原因，最后纳入数据统计分析的两组各 30 例。

数据运用 SPSS17.0 软件进行分析，符合正态分布的计量资料用$\bar{x} \pm S$表示，均数差别的对比运用 t 检验，计数资料采用 X^2 检验，等级资料用秩和检验，P<0.05 则认为差异有统计学意义。

第三节　研究结果

一、一般资料比较

表 1　治疗前性别对比（f）

组别	n	男	女	X^2	P
治疗组	30	13	17	0.278	0.598
对照组	30	11	19		

万方数据

注：样本含量大于 40，各频数≥5，故运用 pearson 卡方检验。

由表 1 可以看出，P=0.598>0.05，无显著性差异，两组性别具有可比性。

表 2 治疗前年龄（岁）分布对比(f)

组别	n	18~29	30~39	40~49	50~60	X^2	P
治疗组	30	5	11	7	7	1.829	0.609
对照组	30	7	7	6	10		

注：样本含量大于 40，各频数≥5，故运用 pearson 卡方检验

由表 2 可以看出，P=0.609>0.05，提示两组的年龄段分布对比无显著差异，具有可比性。

表 3 治疗前年龄（岁）均值的对比($\bar{x}\pm S$)

组别	n	$\bar{x}\pm S$	t	P
治疗组	30	38.97±10.36	-0.574	0.568
对照组	30	40.73±13.29		

注：经 Shapiro-Wilk 正态性检验，两组年龄均符合正态分布，且 F 检验提示两组方差齐性(F=2.666，P=0.108>0.05），故采用两独立样本 t 检验统计分析。

由表 3 可以看出，t=-0.574，P=0.568>0.05，提示两组病人年龄均值对比无明显差异，具有可比性。

表 4 治疗前中医症状积分对比($\bar{x}\pm S$)

组别	n	$\bar{x}\pm S$	t	P
治疗组	30	13.13±4.58	0.240	0.811
对照组	30	12.87±4.00		

注：经 Shapiro-Wilk 正态性检验，两组治疗前的中医症状积分均符合正态分布，且 F 检验提示两组方差齐性（F=-0.891，P=0.349>0.05），故采用两独立样本 t 检验统计分析。

由表 4 可以看出，P=0.811>0.05，表明两组病人治疗前中医症状积分比较无显著差异，具有可比性。

表 5 治疗前两组患者病程对比(f)

组别	n	<1 年	1~5 年	>5 年	X^2	P
治疗组	30	6	11	13	2.234	0.327
对照组	30	10	12	8		

注：样本含量大于 40，各频数≥5，故采用 pearson 卡方检验。

由表 5 可以看出，P=0.327>0.05，提示两组病程对比无明显差异，具有可比性。

二、实验观察项目比较

（一）西医疗效分析

1. 综合疗效对比

万方数据

表 6 两组治疗前后西医总疗效对比（f, $\overline{R}$）

组别	例数	总疗效			总有效率	平均秩次	z	P
		显效	有效	无效				
治疗组	30	2	21	7	76.67%	26.38	-2.105	0.035
对照组	30	1	14	15	50.00%	34.62		

注：疗效为有序变量，治疗分组为定性变量，故采用非参数 Mann-Whitney U 检验。

由表 6 可以看出，治疗组总有效率 76.67%，对照组总有效率 50.00%，运用秩和检验，P=0.035＜0.05，说明两组西医总疗效对比有差异,治疗组疗效优于对照组。

表 7 Holter 心电图平均早搏次数对比（$\overline{x}\pm S$）

组别	治疗前	治疗后	PVC 减少次数
治疗组（n=30）	9970.50±2960.50★	5409.13±3420.71▲	4561.37±1385.26△
对照组（n=30）	9676.77±3099.48	6021.77±3815.96*	3655.00±1386.05

注：经 Shapiro-Wilk 正态性检验，两组治疗前后 Holter 心电图平均早搏次数、PVC 减少次数均符合正态分布，故采用 t 检验统计分析。两组治疗前相比，运用两独立样本 t 检验，F 检验提示两组方差齐性（F=0.027，P=0.869>0.05），经两独立样本 t 检验统计分析得出，与对照组相比，★t=0.375，★P=0.709>0.05。经配对样本 t 检验，两组治疗前后自身相对比，▲t=18.035, ▲P=0.000, *t=14.443, *P=0.000。治疗组 PVC 减少次数与对照组相比，运用两独立样本 t 检验，F 检验提示方差齐性（F=0.352，P=0.555），t 检验结果提示△t=2.533,△P=0.014。

由表 7 可看出，★P>0.05，表明两组治疗前对比无显著差异，具有可比性。▲P 值及*P 均小于 0.05，表明两组治疗均有效。△P<0.05，表明治疗组减少早搏次数更明显，两组对比有统计学差异。

（二）中医症状疗效比较

表 8 治疗前后中医症状疗效对比（f, $\overline{R}$）

组别	例数	总疗效			总有效率	平均秩次	z	P
		显效	有效	无效				
治疗组	30	14	12	4	86.67%	26.03	-2.113	0.035
对照组	30	8	11	11	63.33%	34.97		

注：疗效为有序变量，治疗分组为定性变量，故采用非参数 Mann-Whitney U 检验。

由表 8 可看出，经过治疗后，两组的中医症状均有明显改观，其总有效率分别为 86.67%和 66.67%，且治疗组总疗效高于对照组。P=0.035<0.05，提示两组总疗效比较，差异有统计学意义。

（三）治疗前后中医症状积分比较

万方数据

表9 两组治疗前后中医症状积分对比

组别	中医症状评分 M（P25,P75）		△中医症状评分（$\bar{x}\pm S$）
	治疗前	治疗后	
治疗组	12.00(10.00,17.00)	4.00(3.00,10.25)▲	6.77±3.20*
对照组	12.50(9.00,16.00)	4.00(3.00,13.25)★	5.20±2.59

注：经 Shapiro-Wilk 正态性检验，两组治疗后中医症状积分均不符合正态分布，故两治疗前后自身对比运用非参数 Wilcoxon 符合秩检验，治疗组治疗前后自身对比，▲z=-4.789，▲P=0.000；对照组治疗前后自身对比，★z=-4.795，★P=0.000。经 Shapiro-Wilk 正态性检验，两组治疗前后差值均符合正态分布，故差值对比采用两独立样本 t 检验，F 检验提示方差齐性（F=1.911，P=0.172），t 检验结果提示，*t=2.083，*P=0.042。

由表 9 可以看出，两组治疗后中医症状积分均下降，两组治疗前后经非参数配对检验统计得出，▲P、★P均小于 0.05，差异有统计学意义，提示两种治疗后对改善中医症状均有效。两组差值经两独立样本 t 检验得出，*P=0.042<0.05，提示两组中医症状积分下降程度对比，治疗组下降更明显，差异有统计学意义，表明治疗组对改善中医症状更有效。

三、安全性评价

（一）实验室安全指标分析

本试验过程中，两组患者治疗前后一般体格检查、血、尿、大便常规、心、肝、肾功能均未出现明显变化，说明本药物试验对患者实验室指标无明显不良影响。

（二）不良反应

研究过程中，60 例患者未发现明显不良反应。

万方数据

第三节 讨论

一、功能性频发性室性早搏概况

目前国内外对功能性 PVC 研究明显少于器质性 PVC，功能性频发性 PVC 自 20 世纪 Chugh 等研究以来，其可致室早性心肌病已得到共识，即使是无明显临床症状，亦须长期随访观察。目前西药 AAD 治疗 PVC 有致心律失常副作用，且全部 AAD 均有或轻或重的负性肌力作用[50]，有较多禁忌症，限制了其使用范围，影响临床效果，而部分药物无法改善者运用射频消融术，疗效虽明确，因其有创性、价格昂贵，使许多患者望而却步，迫使医务、科研人员努力寻找中医中药治疗 PVC 新途径。目前治疗功能性频发性 PVC 患者在去除诱因后，如仍有症状，首选 β 受体阻断剂治疗，但临床效果不甚理想，且部分患者无法耐受，有诸多禁忌症，而中药汤剂、中成药等在改善心悸症状方面疗效仍不可小觑。

二、炙甘草汤中医研究现状

近些年炙甘草汤用于治疗 PVC 有大量的临床药理及临床研究报道，可改善早搏次数、缓解临床症状，无明显作用。而广东作为中医药大省，群众基础好，认同度高，服药依从性较高，易被患者认同和接受。以邓铁涛教授为主的医家提出心悸的病位在心，与五脏相关，而心气虚、心阴虚为心悸之本，指出气阴两虚为心悸的总病机，而炙甘草汤出自《伤寒杂病论》，为治疗气阴两虚引起诸多临床症状的代表方。本课题结合现代研究经验，予加减治疗，探索其对改善临床症状、减少早搏次数的临床疗效。

（一）炙甘草汤出处

炙甘草汤作为伤寒病的应用，首载于《伤寒论》：“伤寒，脉结代，心动悸，炙甘草汤主之”。原方中药物及剂量、煎服法如下：“甘草（炙，四两），生姜（切，三两），人参（二两），生地黄（一斤），桂枝（去皮，三两），阿胶二两），麦门冬（去心，半升），麻仁（半升），大枣（擘，三十枚）”，“上九味，以清酒七升，水八升，先煮八味，取三升，去滓，内胶烊消尽，温服一升，日三服”。

（二）本研究方药及配伍特点

1. 以炙甘草为君药：众多医家对炙甘草汤何为君药颇有争论，一说炙甘草为君，一云生地为君。原方中炙甘草用量四两，生地重用一斤，而李杲曾于《脾胃论》论及君药药物用量最多，因此，部分人认为生地黄为君药，然而唐代以前仅有生地、干地黄，并无熟地。当时生地黄即鲜地黄，采摘后不经晒干直接药用，含水分较多。如果将原方中的鲜地黄去除水分，重量应与原方炙甘草重量相当，而炙甘草为补气之品，可益气生血，与“脉结代、心动脉”之气血不足，心失所养遥相呼应，加之方剂命名规律，以药名命名者，此药多为君药。综上所述，本方君药应为炙甘草。

2. 方药加减：本研究方中以炙甘草汤为基础方，去酒、麻仁，改用生地为熟地，易人

万方数据

参为党参，加酸枣仁。

（1）清代名医张锡纯认为，本方中药物，属生地黄用量最多，达一斤，为补肾之要药，唐代以前并无熟地，生地若用量太多则过于寒凉，所以加用清酒，用酒的热性使生地由凉变温，以温补肾阳。熟地，性温，具有滋阴养血，填精益髓之效。故去酒，改生地为熟地，以求温补肾脏之效，且古方中鲜地黄改为今之熟地黄，水分减少，其用量须减少。

（2）炙甘草汤中麻仁为火麻仁或胡麻仁，至今尚未有定论，清代医家柯琴于《伤寒来苏集》中指出，炙甘草汤古书中运用麻黄为误，当为酸枣仁半升。《本草备要》论述酸枣仁甘、润，可敛汗，宁心。《别录》注酸枣仁："久泄，虚汗烦渴，补中，益肝气，坚筋骨，助阴气"。《本草纲目》则提出酸枣仁无毒，长期服用可延年益寿。郭丽[51]等认为现代关于火麻仁、胡麻仁功用之说仍延用清代医家汪昂说法：火麻仁偏于润肠通便，胡麻仁偏于补虚，而酸枣仁有宁心安神之效，当根据临床实际辨证论治选用，若患者为心阴阳虚，兼有便秘，可予火麻仁；而心阴阳两虚，大便正常者，可予胡麻仁，如以心悸、失眠为主则可选用酸枣仁。炙甘草汤主治病症"脉结代，心动悸"为素体虚弱，外来邪气侵袭，以致正不胜邪，心之阴阳两虚，心失所养，神无依附所致。以心悸为主症，故笔者选用酸枣仁以彰显宁心安神之功。

（3）诸药配伍：方中以炙甘草为君药，补益心气，复脉定悸。党参补脾益气；麦冬滋阴润肺，兼可补气，益气养阴，心肺双补；熟地养血滋阴；酸枣仁宁心安神、滋阴，四者皆为臣药。阿胶补血、滋阴润燥；大枣补脾胃，实脾土而养营阴；姜、桂为辛温之品，通阳复脉，与补气养阴药相伍，除燥热而不失温性，充盈气血，通利脉道，此四者共为佐药。诸药并用，共奏益气滋阴、复脉之功。

三、炙甘草汤现代药理研究

（一）抗心律失常

马清华[52]等以心房颤动大鼠为模型，观察炙甘草汤对其外周血 TNF-α 表达的影响，得出炙甘草汤可使血清 TNF-α 指标显著下降，其机制可能为通过阻碍炎症因子发挥作用，改善心房颤动的心肌组织损伤，治疗房颤。李创鹏[53]等将 40 例冠心病经皮冠状动脉成形术（PTCA）术后患者分为治疗组（口服炙甘草）、对照组（口服倍他乐克），观察受试者 P 选择素、CRP、内皮素指标的变化情况，结果显示治疗组相较对照组，可明显降低 P 选择素、CRP、内皮素的水平，提示炙甘草汤对 PTCA 术后患者抗心律失常作用机制可能与抗血小板活化、保护血管内皮细胞明显相关。胡久略[54]等以心律失常大鼠为模型，研究炙甘草汤对治疗不同类型心律失常的作用，结果提示炙甘草汤可显著延迟乌头碱、氯化钙所致 PVC、室速、室颤及死亡时间；且能降低冠脉结扎导致再灌注性损伤诱发心律失常的发病率。

万方数据

（二）对心肌生理特性的作用

马建伟[55]等以豚鼠为实验模型，通过研究得出炙甘草汤能减慢缺血、缺氧状态下的心肌细胞动作电位时程缩短速度。周承志[56]等以兔子为模型，研究发现，炙甘草汤含药血清可抑制其心室肌细胞 L 型 Ca^{2+}通道电流，浓度越高，效果越强。

（三）抗心肌缺血再灌注损伤

袁杰[57]等研究缺血-再灌注损伤大鼠模型发现，炙甘草汤能增加血中超氧化物歧化酶的活性，并降低丙二醛及活性氧含量，显著升高其左心室的功能。

（四）抗衰老作用

研究表明炙甘草汤可清除小鼠的自由基、促进新陈代谢，提高大脑皮层兴奋性，并兴奋延髓呼吸中枢，增强对缺氧的耐受力，改善呼吸、循环、消化、造血等系统功能，增强免疫力、生命力，延缓衰老[58]。

四、炙甘草汤治疗心律失常应用举隅

李健[59]等在常规使用扩冠、抗血小板聚集和降血脂的西药治疗基础上予用复方丹参滴丸口服及炙甘草汤中药煎服治疗冠心病 PVC，对照组予服用胺碘酮进行对比，结果显示治疗组效果优于对照组（$P<0.01$）。张辰浩[60]研究炙甘草汤对冠心病 PCI 术后缓慢性心律失常临床疗效观察，对照组予以基础冠心病二级预防用药，治疗组在对照组基础上联合中药炙甘草汤口服 1 个月，两组疗效比较具有统计学差异，得出治疗组疗效较好。王振琴[61]予炙甘草汤合美西律治疗冠心病 PVC，与单用西药组对比，结果提示治疗组疗效较好。

五、总结及展望

西药对心律失常的电生理研究较多，有明确的作用靶点，但中药在此领域研究相对薄弱。近年相关文献对炙甘草汤现代药理研究较多，可能影响心室肌细胞的动作电位、改善内皮细胞等功能，多集中在心肌生理特性方面研究，且多为动物实验；而临床研究方面显示其有较好的抗心律失常作用，安全性高，由于观察时间所限、样本量较少、实验室观察指标单一，临床设计尚有诸多细节可待商讨。鉴于以上几个问题，在研究与开发 PVC 治疗中药时，须加强对中药药理及临床实验研究，寻找循证医学证据。远期效果需增加观察时间，扩大样本量，制定统一的临床诊断标准，设置更严格、精密的临床研究设计，减少误差，增加临床随访，提高科学性和可信度，以期得到更广泛、客观的研究结果，对于指导古方运用于临床，意义深远。

万方数据

结　语

本课题在导师的指导下，由笔者独立完成，以中医学理论为研究基础，选用炙甘草汤加减对功能性频发性 PVC（气阴两虚型）进行临床研究，结合现代诊断、治疗技术，经统计分析结果显示，治疗组（炙甘草汤加减）与对照组（倍他乐克）中医证候总有效率分别为 86.67%和 63.33%，动态心电图 PVC 次数改善总有效率分别是 76.67%、50.00%。据统计学分析，两组疗效有差异；说明治疗组不仅能改善 PVC、减少 PVC 次数，并可改善临床症状。且疗效优于单用倍他乐克。安全性评价：治疗后一般体格检查，实验室指标等未见明显异常，各组患者未出现明显不良反应，提示炙甘草汤加减合倍他乐克用于治疗功能性频发 PVC 安全、有效，为 PVC 治疗提供新的临床思路。

综上所述，本研究建立在西医药理及临床研究基础之上，以中医辨证论治为切入点，采用中、西药合用，治疗功能性频发性 PVC，有良好疗效，但仍需更大规模的前瞻性研究加以证实。

万方数据

参考文献

[1] Ng GA.Treating patients with ventricular ectopic beats[J].Heart,2006,92(11):1707-1712.

[2] 王雅莉,万启南.中医药治疗冠心病室性早搏研究进展[J].云南中医中药杂志，2008,09:50-52.

[3] Wilber DJ.Ventricular ectopic beats:not so benign[J].Heart,2009,95(15):1209-1210.

[4] Chugh SS,Shen WK,Luria DM, et al.First evidence of premature ventricular complex-induced cardiomyopathy: a potentially reversible cause of heart failure[J].J Cardiovasc El ectrophysiol,2000,11(3):328-329.

[5] 杨宝峰.药理学（第七版）[M].北京:人民卫生出版社,2010:207.

[6] 惠玲玲,张晓锋.室性早搏性心肌病[J].心脏杂志,2013,01:104-105+109.

[7] Duffee D F,Shen W K,Smith H C.Suppression of Frequent Premature Ventricular Contractions and Improvement of Left Ventricular Function in Patients With Presumed Idiopathic Dilated Cardiomyopathy[J].Mayo Clinic Proceedings,1998,73(5):430-433.

[8] 高谦,张筠,李成建.药物所致室性早搏[J].慢性病学杂志,2010,09:985.

[9] 张春雨,江立,马满玲.复方樟柳碱注射液诱发室性早搏[J].药物不良反应杂志,2006,8(3):221-222.

[10] 王朝晖.心内直视手术后频发室性早搏治疗体会[J].辽宁医学院学报,2006,27(2):93-94.

[11] 芦英.拔牙诱发严重的心律失常 2 例[J].中国现代药物应用，2009，3(20):163-163.

[12] Dogan M, Yiginer O, Omer U Z, et al. The Effects of Female Sex Hormones on Ventricular Premature Beats and Repolarization Parameters in Physiological Menstrual Cycle[J].Pacing & Clinical Electrophysiology,2016.

[13] 陈灏珠,林果为,王吉耀.实用内科学（第 14 版）[M].人民卫生出版社,2013:1366-1368.

[14] 郭继鸿.折返性室性早搏[J].临床心电学杂志,2005,01:12-13.

[15] 丁绍祥.触发活动致心律失常发生机制的探讨[J].中国循环杂志,2015(4).

[16] Zimik S,Nayak A R,Pandit R. A Computational Study of the Factors Inf

万方数据

luencing the PVC-Triggering Ability of a Cluster of Early Afterdepolarization-Capable Myocytes.[J].Plos One, 2015, 10(12).
[17] 陈小贞,杨琳,金印彬.电紧张扩布在折返性室性心律失常发生中的作用[J].中国心脏起搏与心电生理杂志,2008,05:423-426.
[18] Xu W, Li M, Chen M, et al.Effect of burden and origin sites of premature ventricular contractions on left ventricular function by 7-day Holter monitor[J].Journal of Biomedical Research, 2015, 29(6):465-474. DG
[19] Lu F,Benditt DG,Yu J,et al.Effects of catheter ablation of "asymptomatic"frequent ventricular premature complexes in patients with reduced(<48%)left ventricular ejection fraction[J].Am J Cardiol,2012,110(6):852-856.
[20] 郭继鸿.β受体阻滞剂在心律失常治疗中的应用[J].中国心脏起搏与心电生理杂志,2007,01:4-6.
[21] Pedersen C T,Kay G N,Kalman J,et al.EHRA/HRS/APHRS expert consensus on ventricular arrhythmias[J].Heart Rhythm the Official Journal of the Heart Rhythm Society,2014,11(10):e166-96.
[22] 曹克将,张凤祥.室性心律失常药物治疗进展[J].内科急危重症杂志,2011,03:135.
[23] Fuster V, Rydén LE, Cannom DS, et al. ACC/AHA/ESC 2006 guidelines for management of patients with ventricular arrhythmias and the prevention of sudden cardiac death—executive summary[J]. Journal of the American College of Cardiology, 2006, 48(5):2099-2140.
[24] 中国生物医学工程学会心脏起搏与电生理分会.射频导管消融治疗快速心律失常指南（修订版）[J].中国心脏起搏与心电生理杂志,2002,16(02):81-95.
[25] 童鸿.第65课 室性期前搏动和阵发性室性心动过速 EHRA/HRS/APHRS 专家共识[J].心电与循环,2015,34(3).
[26] Rogers W J, Epstein A E, Arciniegas J G, et al.The Cardiac Arrhythmia Suppression Trial (CAST) Investigators.Preliminary report Effect of encainide and flecainide on mortality in a randomized trial of arrhythmia suppression after myocardial infarction[J].New England Journal of Medicine, 1989,321(6):406-412.
[27] 侯应龙.抗心律失常药物的致心律失常作用[J].中国心脏起搏与心电生理杂志,2007,21(1):14-16.

万方数据

[28] 杨宝峰,蔡本志.心律失常发病机制研究进展[J].国际药学研究杂志,2010,02:81-88.

[29] 刘泽银,邹旭,罗英.邓铁涛心脾相关论治疗心悸临床经验总结[J].中国中医药信息杂志,2007,07:82-83.

[30] 刘平,李晓斌,李静等.杨思进教授治疗心悸的经验总结[J].世界最新医学信息文摘,2015,65:100-101.

[31] 龚培培,李鑫,王建国,等.刘建和教授辨治心悸(心律失常)学术思想及临证经验[J].中医药导报,2015(18):98-99.

[32] 华愫.华明珍教授从肾论治心悸的经验[J].中国中医药现代远程教育,2011,09:15-16.

[33] 王萌,邓悦.邓悦教授治疗心律失常经验[J].长春中医药大学学报,2013,29(2):219-219.

[34] 申啸笑,杨忠奇,陈一鸣,等.岭南医学流派对心悸病的辨证规律研究[J].中国中医药现代远程教育,2013,24:3-5.

[35] 刘贯龙,高洪春.试述《伤寒杂病论》论治心悸的脏腑辨证[J].现代中医药,2013,01:72-73.

[36] 姜瑞雪,朱文锋,马作峰.心悸辨证分型及证素分布的文献研究[J].中华中医药学刊,2008,01:184-186.

[37] 刘英姿,过伟峰,袁园.周仲瑛教授从脏腑相关辨治心悸的经验[J].深圳中西医结合杂志,2009,01:52-54.

[38] 周仲瑛.中医内科学[M],北京:中国中医药出版社,2007:129-131.

[39] 宋丹,丁碧云.酸枣仁汤加减治疗室性早搏的疗效观察[J].西部中医药,2012,25(3):60-61.

[40] 李志勇.桂枝甘草龙骨牡蛎汤加味治疗室性早搏 56 例临床观察[J].内蒙古中医药, 2013, 32(25):13-14

[41] 张曦光,刘臣,张艳.小柴胡汤加味治疗室性早搏的临床观察[J].四川中医,2011(6):75-76.

[42] 沈安明,谢轶群,吕军.健心胶囊治疗冠心病室性早搏临床观察[J].实用中西医结合临床,2008,8(6):11-12.

[43] 邹建刚,曹克将,李东野,等.参松养心胶囊治疗室性早搏的多中心随机对照研究[J].疑难病杂志,2007,03:138-140.

[44] 付华宾,彭志群,胡孟泉,等.稳心颗粒治疗室性早搏 56 例[J].中国中医药现代远程教育,2013,23:15-16.

万方数据

[45] 尹克春,李绍波,刘淑娟,等.当归Ⅱ号注射液穴位注射治疗冠心病室性早搏临床观察[J].广东医学,2007,28(11):1862-1864.
[46] 李锐,李学林.丹红注射液治疗老年室性早搏的临床研究[J].西部医学,2008,20(6):1202-1203.
[47] 史丰奇.丹参酮ⅡA磺酸钠注射液治疗室性早搏疗效观察(附18例报告)[J].哈尔滨医药,2014,05:284.
[48] 陈新.黄宛临床心电图学[M],北京:人民卫生出版社,2012,6:285.
[49] 国家中医药管理局.中医病症诊断疗效标准[M].南京:南京大学出版社,1994:19.
[50] 郑筱萸.中药新药临床研究指导原则[M].中国医药科技出版社,2002.
[51] 曹克将,张凤祥.室性心律失常药物治疗进展[J].内科急危重症杂志,2011,03:135.
[52] 郭丽,杨红梅,王蓉,等.论炙甘草汤中麻仁[J].四川中医,2015(6):32-34.
[53] 马清华,刘维琴.炙甘草汤对房颤大鼠外周血TNF-α表达的影响[J].中国中医急症,2012,01:68-74.
[54] 李创鹏,彭敏,刘培中.炙甘草汤对PTCA术后心律失常P选择素内皮素CRP影响临床研究[J].中医药学刊,2006,04:655-656.
[55] 胡久略,黄显章.炙甘草汤抗心律失常作用的实验研究[J].时珍国医国药,2008,05:1189-1190.
[56] 马建伟,胥爱文,温晓竞等.炙甘草汤对缺血缺氧心室乳头肌细胞电生理效应的研究[J].陕西中医,2007,06:748-750.
[57] 周承志,张道亮,王腾等.炙甘草汤含药血清对兔心肌细胞钙电流的影响[J].北京中医药大学学报,2007,07:468-471.
[58] 袁杰.炙甘草汤对大鼠在体心肌缺血-再灌注损伤后左心功能及抗氧化酶的影响[J].时珍国医国药,2008,02:411-412.
[59] 阚方旭.心血管病经方论治[M].北京:人民卫生出版社,2007:145.
[60] 李健,叶烨,吴晓新等.炙甘草汤联合丹参滴丸治疗冠心病室性早搏临床观察[J].新中医,2009,07:43-44.
[61] 张辰浩.炙甘草汤治疗冠心病PCI术后缓慢性心律失常63例疗效观察[J].现代中西医结合杂志,2013,22(20):2227-2229.
[62] 王振琴.炙甘草汤联合盐酸美西律片治疗冠心病室性期前收缩临床观察[J].河北中医,2015,07:1044-1045.

万方数据

附　录

附 录 1　英文缩略语

缩略语	英文全称	中文全称
AAD	antiarrhythmic drug	抗心律失常药物
TCM	Traditional Chinese Medicine	中医
PVC	premature ventricular contraction	室性早搏
SHD	Structural Heart Disease	结构性心脏病

万方数据

附 录 2　统计学审核证明

统计学审核证明

学号：20131123764

广州中医药大学研究生学位论文统计学审核证明

兹有 硕 士研究生 黄勉婷（导师 罗治华 ）的学位论文《炙甘草汤加减合倍他乐克治疗功能性早室性早搏的临床研究》中有关统计学方面的内容，经我部门审定合格，特此证明。

附属广东省第二中医院
中医药信息研究室

附属广东省第二中医院
中医药信息研究室
负责人：任薇　　赵自明
二零一六年五月三十一日

万方数据

致　谢

三年学习生涯匆匆而过，回首过往时光，诸多感慨。

感恩父母，养育、教导，成长点滴关怀之恩铭记心中。岁月无情，一缕缕银丝、一道道皱纹，道尽对儿女深沉的爱。父母用二十余年的时光陪伴我们，感慨今后恐无法以同等时光回报，古人教悔“百善孝为先”、圣人训“首孝悌，次谨信”，时刻提醒自己，“孝”常在心，而“顺”常于行。

三年前有幸开始师从我的导师罗治华教授，导师严谨治学，为人正派，对病患的细心、关怀，对我的敦敦教诲仍在耳畔回响。感谢他在临床工作中对我言传身教、严格要求，对我生活的关心。师恩难忘，难以言表。

感谢新会中医院领导、医护人员对我课题的指导、帮助。感谢在轮训期间的所有带教老师，感谢你们教导我临床思维方法和诊治技术，让我受益匪浅！

感谢我的师兄、师姐、师弟、师妹们，三年间，一起学习、聚餐、游玩……是你们让我在他乡感受到家一般的温暖！

最后谨向各位参与论文评审、统计学检测及答辩的专家、教授评委们表示衷心地感谢！

万方数据

炙甘草汤加减+酒石酸美托洛尔对冠心病心律失常的治疗效果及安全性探究

黄小强，金明艳
（福州市第七医院，福建 福州 350001）

【摘要】目的 冠心病心律失常患者应用炙甘草汤加减+酒石酸美托洛尔治疗的效果及安全性进行分析。方法 随机选取2018年6月～2019年6月我院收治的96例冠心病心律失常的患者，将其按照简单随机化法均分为对照组和观察组，对照组应用酒石酸美托洛尔，观察组应用炙甘草汤加减+酒石酸美托洛尔，两组治疗疗效及不良反应发生率对比。结果 观察组治疗疗效优于对照组，且观察组不良反应发生率低于对照组，差异有统计学意义（P＜0.05）。结论 冠心病心律失常患者应用炙甘草汤加减+酒石酸美托洛尔治疗效果显著，安全性较高，值得应用。

【关键词】冠心病心律失常；炙甘草汤；酒石酸美托洛尔；治疗效果；安全性
【中图分类号】R285.6 【文献标识码】A 【文章编号】ISSN.2095.6681.2020.9.168.02

冠心病心律失常会对患者生命造成非常严重的影响，临床中主要应用药物进行治疗，但在治疗过程中患者会出现各种不良反应，反而会影响其恢复，有资料显示将中药与西药结合使用有较好的治疗效果，它可以有效缓解患者的症状，降低其不良反应。本次研究主要对冠心病心律失常患者应用炙甘草汤加减+酒石酸美托洛尔治疗的效果及安全性进行分析。

1 资料与方法

1.1 一般资料

随机选取2018年6月～2019年6月我院收治的96例冠心病心律失常的患者，将其按照简单随机化法均分为对照组和观察组，对照组男26例，女22例，年龄49～70岁，平均（59.8±8.7）岁，观察组男28例，女20例，年龄51～70岁，平均年（61.5±8.2）岁，通过对比两组，差异无统计学意义（P＜0.05）。

1.2 方法

1.2.1 对照组

对照组应用酒石酸美托洛尔（批准文号：H32025391，阿斯利康制药有限公司），口服，25 mg，1天2次，连续服用1个月。

1.2.2 观察组

观察组应用炙甘草汤加减+酒石酸美托洛尔，炙甘草汤药物组成有阿胶6 g、炙甘草12 g、生姜9 g、麦门冬10 g、人参6 g、桂枝9 g、生地黄50 g、麻仁10 g、大枣10个，用水煎煮至500 mL，1天1剂，分两次服用，酒石酸美托洛尔与对照组使用方法相同，连续服用1个月。

1.3 观察指标及疗效评定标准

两组治疗疗效及不良反应发生率对比。疗效评定标准：显效：治疗后症状基本消失，心律失常发作次数明显减少；有效：治疗后症状有所改善，心律失常发作次数有所减少；无效：治疗后症状没有缓解，心律失常发作次数没有减少，甚至病情有加重。总有效率=（显效+有效）/总例数*100%。不良反应包括头晕、腹痛、呕吐、心悸、心动过缓。

1.4 统计学方法

采用统计学软件SPSS22.0处理研究数据，计量资料：（$\bar{x}\pm s$）t检验；计数资料：（n，%），x^2检验。P＜0.05：差异有统计学意义。

2 结 果

2.1 两组治疗疗效对比

观察组治疗疗效优于对照组，差异有统计学意义（P＜0.05）。如表1所示。

表1 两组治疗疗效对比[n（%）]

组别	例数	显效	有效	无效	总有效率
观察组	48	26（54.17）	18（37.50）	4（8.33）	44（91.67）
对照组	48	19（39.58）	17（35.42）	12（25.00）	36（75.00）
x^2	/	/	/	/	4.800
P	/	/	/	/	0.028

2.2 两组不良反应发生率对比

观察组头晕2例（4.17%），腹痛1例（2.08%），呕吐2例（4.17%），发生率5例（10.42%）心动过缓，对照组头晕5例（10.42%），腹痛3例（6.25%），呕吐6例（12.50%）心动过缓，发生率14例（29.17%），x^2=5.315，差异有统计学意义（P=0.021＜0.05）。

3 讨 论

冠心病在临床中较常见，其常见的并发症为心律失常，大多发生于中老年人群中，其年龄较大机体功能不断退化，患者发病时心脏的血流动力会受到影响，使其心功能出现异常并出现心肌缺血，这不仅会影响其生活质量还会对其生命造成威胁，引发患者猝死[1]。

临床中主要应用药物进行治疗，美托洛尔应用于冠心病心律失常患者的治疗中，它可以对患者的内分泌水平起到调节作用，同时还能使其心肌耗氧量降低从而为其心肌缺血区提供血液，缓解其症状，但长期服用这种药物会使患者出现不良反应影响治疗效果。中医认为冠心病心律失常是因为情志所伤或体质等原因所引起，其治疗的关键在于益气补血，炙甘草汤中药物组成有阿胶、炙甘草、生姜、麦门冬、人参、桂枝、生地黄、麻仁、大枣，其中麻仁、阿胶具有滋阴补血的作用，生姜具有温阳通脉的作用，炙甘草具有益气养心的作用，大枣具有补气补脾的作用等，将以上药物联合使用可以达到滋阴、补血、益气的

（下转181页）

万方数据

气运化失调就容易导致瘀血产生，进一步形成冠心病心绞痛[3]。止麻消痰活血汤我院名中医邢月朋老先生治疗动脉粥样硬化肢体麻木的经验方，具有益气、化浊、活血通脉之功效，近些年来我们尝试应用于冠心病病人亦取得良好效果[4]。方中的黄芪能够增益心气，当归、川芎具有活血祛瘀，行气止痛的作用，红花、丹参、桃仁能够化瘀通络，多种药物共同作用，能够有效扩张冠脉和周围血管，降低心肌耗氧[5]。止麻消痰活血汤能够有效降低血液黏滞度，改善心肌功能，降低甘油三酯、血清总胆固醇、低密度脂蛋白，升高高密度脂蛋白，改善患者血脂指标[6]。本研究中观察组有效率高于对照组；两组甘油三酯、血清总胆固醇、低密度脂蛋白相比于治疗前显著降低，且观察组显著低于对照组；高密度脂蛋白升高，且观察组显著高于对照组，提示止麻消痰活血汤可有效缓解冠心病心绞痛患者临床症状，改善患者血脂指标。

综上，止麻消痰活血汤可有效缓解冠心病心绞痛患者临床症状，改善患者血脂指标，且疗效优于常规西医治疗，值得临床推广应用。

参考文献

[1] 匡旭东,孙家庆,肖利华.冠心止痛贴配合丹七活血汤及阿托伐他汀治疗对心血瘀阻型冠心病心绞痛患者血脂代谢及血管内皮功能的影响[J].现代中西医结合杂志,2018,27(26):2927-2930.
[2] 曹　丹,余炳成.益气养阴活血汤联合西药对冠心病心绞痛血清CRP、TNF-α及血脂血液流变学水平的影响[J].吉林中医药,2017,37(9): 903-906.
[3] 王淑洁.常规西药配合自拟中药益气活血汤治疗冠心病心绞痛88例分析[J].首都食品与医药,2017,24(12):84-85.
[4] 胡　光,程小龙,曲　冰.自拟益气活血汤加减联合常规治疗对气虚血瘀型冠心病心绞痛患者血液流变学的影响[J].中医药信息,2017,34(4):86-88.
[5] 李春晖.益气活血汤联合西药治疗冠心病心绞痛疗效及对血清炎症因子水平的影响[J].湖南中医药大学学报,2017,37(9):969-972.
[6] 周　艳,袁幕洁,吉岳萍.中西医结合治疗冠心病冠脉支架植入术后再发心绞痛临床分析[J].内蒙古中医药,2017,36(5):60.

（上接 168 页）

功效[2]。本次研究结果显示观察组治疗疗效优于对照组，且观察组不良反应发生率低于对照组。

综上所述，冠心病心律失常患者应用炙甘草汤加减+酒石酸美托洛尔治疗效果显著，安全性较高，值得应用。

参考文献

[1] 万志敏,万美萍.美托洛尔联合炙甘草汤加减治疗气阴两虚型冠心病心律失常的效果观察[J].基层医学论坛,2019,23(19):2777-2778.
[2] 王　林.炙甘草汤加减联合酒石酸美托洛尔治疗冠心病心律失常的效果及不良反应分析[J].当代医学,2019,25(15):166-167.

（上接 177 页）

蛋白呈高表达，进而调节caspase-3活化，导致神经元损伤增加细胞凋亡[9]。文献报道[10、11]，鱼藤酮具有较强亲脂性可通过血脑屏障，选择性抑制线粒体复合物Ⅰ的活性，引起氧化应激和细胞凋亡，其诱导的PD模型能够重现行为学改变及病理学特征，接近人类PD疾病表现，因此本课题采用鱼藤酮进行PD大鼠模型制备，观察凋亡相关蛋白表达情况。本实验研究表明，蛭龙活血通瘀胶囊能够改善PD大鼠的行为学表现，减少凋亡相关蛋白Bax、Caspase-3在中脑黑质区域的表达，由此可见，蛭龙活血通瘀胶囊可能通过降低凋亡相关蛋白在脑内的表达从而发挥治疗帕金森病的作用。

参考文献

[1] 中华医学会神经病学分会帕金森病及运动障碍学组.中国帕金森病治疗指南(第三版)[J].中华神经科杂志.2014.47(6):428.
[2] 姜　新.虫草素对鱼藤酮诱导的PD模型多巴胺能神经元的保护作用及其抗凋亡机制研究[D].扬州大学,2017.
[3] 侯晓丽.人参皂苷-Rg3对帕金森病模型大鼠黑质多巴胺能神经元保护作用机制研究[D].扬州大学,2012.
[4] 吴勉华,王新月.中医内科学[M].第3版．北京:中国中医药出版社,2012:441-446.
[5] 李　哲,胡瀛宇.等.帕金森病中医治疗进展[J].中医药现代化.2014.16(4):909.
[6] 王　蔚,沈宏萍,杨思进,杜　渊.蛭龙活血通瘀胶囊治疗帕金森病临床疗效观察[J].光明中医．2019,34(18):2803-2805．
[7] Ferro M M,Bellissimo M L,Anselmo-Franci J A,et al.Comparison of bilaterally 6-OHDA and MPTP-lesioned rats as models of the early phase of Parkinson＇s disease：histological，neurochemical，motor and memory alteratins[J].J Neurosci Methods.2005,148(1):78.
[8] 吴建军,肖　清,刘　凯,等.细胞凋亡学说在帕金森病发病机制的理论探索［J］.中国老年学杂志.2009,29:2135.
[9] Cregan S P,Maclautin J G,Craig C G,et al.Bax-dependentcaspase-3 activiation is a key determinant in p53-induced apoptosisin neurons [J].J Neurosci,1999,19(18):7860-7869.
[10] Hollingworth R M,Ahammadsahib K I,Gadelhak G,et al.New inhibitors of complex Ⅰ of the mitochondrial electron transport chain with activity as pesticides[J].Biochem Soc Trans.1994,22(1):230-233.
[11] Li N,Ragheb K,Lawler G,et al.Mitochondrial complex Ⅰ inhibitor rotenone induces apoptosis through enhancing mitochondrial reactive oxygen species production[J].J Biol Chem.2003,278(10):8516-8525.

万方数据

美托洛尔、炙甘草汤加减联合治疗气阴两虚型冠心病心律失常的效果研究

李书琴
（武安市第一人民医院，河北 邯郸　056300）

【摘要】目的　探讨美托洛尔、炙甘草汤加减联合治疗气阴两虚型冠心病心律失常的临床效果。方法　将我院2017年1月～2019年3月收治的气阴两虚型冠心病心律失常患者60例纳为对象，随机将60例患者分成两组，各组30例。对照组用美托洛尔治疗，而观察组在采用美托洛尔治疗的同时，再使用炙甘草汤加减治疗。结果　观察组心悸、胸痛和胸闷、心烦失眠与口干以及气短乏力中医证候评分比对照组低，$P<0.05$。结论　气阴两虚型冠心病心律失常用美托洛尔和炙甘草汤加减联合治疗的效果明显。

【关键词】冠心病心律失常；气阴两虚型；美托洛尔；炙甘草汤
【中图分类号】R54　　【文献标识码】A　　【文章编号】ISSN.2095.6681.2019.32.52.01

冠心病心律失常属于心血管疾病之一，一般常以西医西药治疗，但近年多个研究指出，单纯西药治疗心血管疾病存在一定的局限性，多数患者的体内气机不能得到有效调节，病情复发率高[1]。中医理论认为冠心病心律失常的患者发病是因为体虚，常见气阴两虚型，治疗时则注重对患者补血益气和调和阴阳，促使患者体内的气机和脏器功能恢复。本次回顾性分析我院采用中西医联合治疗的气阴两虚型冠心病心律失常患者的临床情况，旨在探析美托洛尔及炙甘草汤加减联用的效果。

1　资料与方法

1.1 一般资料

资料选自我院2017年1月～2019年3月，纳入收治的气阴两虚型冠心病心律失常患者60例为对象，患者认知功能正常，未合并精神疾病史和严重肝肾功能障碍。随机将60例患者分成两组：对照组30例患者年龄56～78岁、平均（65.7±5.2）岁，男性19例和女性11例；观察组30例患者年龄55～79岁、平均（65.4±5.0）岁，男性20例和女性10例。两组一般资料比较无差异，$P>0.05$。

1.2 方法

对照组：美托洛尔：让患者服用琥珀酸美托洛尔缓释片，单次给药23.75～47.5 mg，每天服药1次。

观察组：炙甘草汤加减：组方：生地黄50 g、炙甘草15 g和生姜10 g、人参10 g、桂枝10 g以及阿胶10 g（烊化服）、火麻仁10 g与麦门冬10 g、大枣10枚，失眠加用酸枣仁12 g和合欢皮10 g，心阳不振加用生牡蛎15 g及附子10 g，肝肾阴虚加用鳖甲15 g及龟板10 g，心虚加用茯苓15 g及柏子仁10 g，水肿少尿加用葶苈子15 g和茯苓10 g，心血瘀阻加用川芎6 g及红花10 g、丹参15g，将上述每剂中草药用3000 ml水煎煮，取汁300 ml分早晚两次各150 ml温服。

1.3 评价指标和标准

持续用药8周观察疗效，根据《中医诊断学（第9版）》选择气阴两虚型冠心病心律失常的主要证候心悸、胸痛和胸闷、心烦失眠与口干以及气短乏力，用3级评分法判断症状严重程度，0分无症状，1分轻度症状，2分中度症状，3分重度症状，得分高提示症状严重。

1.4 统计学方法

统计学软件选择SPSS 24.0，计量资料用（$\bar{x}\pm s$）表示，t检验；$P<0.05$，差异有统计学意义。

2　结果

观察组心悸、胸痛和胸闷、心烦失眠与口干以及气短乏力中医证候评分比对照组低，$P<0.05$；见表1。

表1　中医证候评分（$\bar{x}\pm s$，分）

组别	心悸	胸痛	胸闷	心烦失眠	口干	气短乏力
观察组	0.3±0.1	0.6±0.3	0.7±0.5	0.4±0.1	0.3±0.2	0.2±0.1
对照组	1.4±0.2	1.5±0.2	1.6±0.1	1.8±0.3	1.9±0.5	1.8±0.4
t	26.9444	13.6720	9.6676	24.2487	16.2735	21.2548
P	0.0000	0.0000	0.0000	0.0000	0.0000	0.0000

3　讨　论

美托洛尔是选择性β1受体抑制剂，抗心律失常作用显著，用药后能竞争性的抑制β1肾上腺素能受体，降低交感神经兴奋度，起到阻止钙离子及钠离子内流，并抑制钾离子外流，达到抗心律失常的效果[2]。本次采用炙甘草汤加减方治疗气阴两虚型冠心病心律失常，本方出自《伤寒论》，用于治疗辨太阳病脉证的治疗，方中中草药合用能起到益气养血和滋阴复脉的效果，重用炙甘草，可起到甘温益气和中缓急、通经利血的作用，合用生地黄、麦门冬以及火麻仁等，可滋阴养血和舒筋温阳、通络，对患者的体内气机起到有效调节和改善，使其病情彻底恢复。

综上所述，气阴两虚型冠心病心律失常在接受常规西药治疗的同时，采用炙甘草汤加减治疗的效果明显，可依据患者的实际病情，为其提供针对性治疗方案，促使其病情得到彻底康复。

参考文献

[1]　张晓晓.中西医结合治疗痰火扰心型频发室性早搏46例临床观察[J].中国民族民间医药,2019,28(13):120-122.
[2]　何苗苗.用中西医结合疗法治疗心律失常的效果分析[J].当代医药论丛,2019,17(10):219-220.
[3]　崔中正.益气活血方配合常规西药改善冠心病心律失常的临床效果[J].临床医学研究与实践,2018,3(25):114-115.

本文编辑：李　豆

●基层临床●

炙甘草汤联合美托洛尔治疗冠心病室性心律失常临床观察

林文志

（北京市丰台区西罗园社区卫生服务中心　北京 100077）

摘要：目的：探究炙甘草汤联合美托洛尔对冠心病室性心律失常患者心率、QTd 及 QTc 间期的影响。方法：选取 2016 年 2 月 ~2018 年 10 月于我院诊治的冠心病室性心律失常患者 60 例，采用随机数字表法分为对照组和观察组各 30 例。对照组给予琥珀酸美托洛尔缓释片口服，观察组在对照组治疗基础上联合炙甘草汤治疗。对比两组临床疗效，治疗前后心率、心电图变化及不良反应发生率。结果：观察组治疗总有效率高于对照组，治疗后心率较对照组低，QTd 间期较对照组短，QTc 间期较对照组长，不良反应发生率低于对照组，差异均有统计学意义（$P<0.05$）。结论：采用炙甘草汤联合美托洛尔治疗冠心病室性心律失常患者可显著改善患者心率 QTd 及 QTc 间期时长，疗效显著。

关键词：冠心病室性心律失常；炙甘草汤；美托洛尔；心率；QTd；QTc

中图分类号：R541.7　　**文献标识码**：B　　doi:10.13638/j.issn.1671-4040.2019.07.070

冠心病心律失常是临床常见疾病，冠状动脉粥样硬化后造成心肌供血不足，当合并心律失常后，冠心病病情将进一步加重，若得不到及时有效治疗，会对患者生命安全造成影响[1]。冠心病室性心律失常以早搏为主要表现形式，患者服药后临床症状可在短时间内缓解，但可诱发新的心律失常，且药物服用量较大，不良反应多，不利于预后[2]。中医学认为室性心律失常所导致的早搏多为本虚标实之证，以虚为主，虚者气血阴阳亏虚、心神失养，气血运行不畅，治疗以益气滋阴、气阴双补为主[3]。本研究探讨炙甘草汤联合美托洛尔对冠心病室性心律失常患者心率、QTd 及 QTc 间期的影响。现报道如下：

1　资料与方法

1.1　一般资料　选取 2016 年 2 月 ~2018 年 10 月于我院诊治的冠心病室性心律失常患者 60 例，采用随机数字表法分为对照组和观察组各 30 例。观察组男 16 例，女 14 例；年龄 47~75 岁，平均（55.38±2.67）岁；病程 8~18 个月，平均（12.59± 2.43）个月；临床症状：心悸失眠 17 例，胸闷气短 20 例，头晕乏力 16 例。对照组男 17 例，女 13 例；年龄 45~78 岁，平均（56.01± 2.11）岁；病程 8~19 个月，平均（12.63± 2.27）个月；临床症状：心悸失眠 16 例，胸闷气短 22 例，头晕乏力 18 例。两组一般资料比较，差异无统计学意义（$P>0.05$），具有可比性。

1.2　诊断标准　西医符合《急性冠脉综合征急诊快速诊疗指南》[4]中冠心病相关诊断标准，经心电图检查明确诊断，24 h 内心律失常次数＞300 次，心功能≤2 级；中医符合《冠心病中医病因病机的认识与探索》[5]中气阴两虚证诊断标准，表现为心胸隐痛、心悸气短、倦怠乏力、易出汗、舌质淡红、舌体胖大且存在齿痕，苔薄白、脉虚细缓或结代。

1.3　纳入与排除标准　纳入标准：（1）符合上述中西医诊断标准；（2）存在室性期前收缩及非阵发性心动过速；（3）知情同意，自愿参与。排除标准：（1）合并肝、肾功能异常；（2）存在内分泌系统疾病，如甲状腺功能异常；（3）伴随心功能不全等其他心脏疾病；（4）无法耐受本次研究治疗。

1.4　治疗方法　所有患者均于参与本研究前 7 d 停止服用其他抗心律失常药物。对照组给予单纯西药治疗，琥珀酸美托洛尔缓释片（国药准字 J20150044）口服，47.5 mg/ 次，1 次 /d。观察组在对照组治疗基础上联合炙甘草汤治疗。炙甘草汤组方如下：阿胶（冲）、生姜、人参、麦门冬、火麻仁、桂枝各 10 g，炙甘草 15 g，生地黄 50 g，大枣 10 枚。心虚加柏子仁 10 g、茯苓 15 g；肝肾阴虚加龟板 10 g、鳖甲 15 g；失眠加合欢皮 10 g、酸枣仁 12 g；水肿尿少加茯苓 10 g、葶苈子 15 g。1 剂 /d，水煎熬取汁 300 ml，150 ml/ 次，早晚服用。两组连续治疗 8 周。

1.5　观察指标　（1）疗效：临床症状消失，心律失常发生率较治疗前减少≥90%为显效；临床症状较治疗前明显改善，心律失常发生率较治疗前减少 50%~90%为有效；临床症状未改善，心律失常发生率减少＜50%为无效[6]。总有效 = 显效 + 有效。（2）对比两组治疗前及治疗 8 周后心率变化情况。（3）分别于治疗前及治疗 8 周后采用同步十二导联心电图对患者进行检查，连续测量 3 个 QTd 间期及 QTc 间期。（4）统计两组服药期间不良反应发生率，包括恶心、窦性心动过缓等。

1.6　统计学分析　采用 SPSS22.0 统计学软件对数据进行分析。计量资料以（$\bar{x}\pm s$）表示，采用 t 检验；计数资料用%表示，采用 χ^2 检验。以 $P<0.05$ 为差异具有统计学意义。

2　结果

2.1　两组临床疗效比较　观察组治疗总有效率较

对照组高，差异有统计学意义（$P<0.05$）。见表 1。

表 1　两组临床疗效比较[例(%)]

组别	n	显效	有效	无效	总有效
观察组	30	15（50.00）	12（40.00）	3（10.00）	27（90.00）
对照组	30	6（20.00）	13（43.33）	11（36.67）	19（63.33）
χ^2					5.963
P					0.015

2.2　两组心率、QTd 及 QTc 间期比较　两组治疗前心率、QTd 及 QTc 间期比较，差异无统计学意义（$P>0.05$）；治疗后，观察组心率较对照组低，QTd 间期较对照组短，QTc 间期较对照组长，差异有统计学意义（$P<0.05$）。见表 2。

表 2　两组心率、QTd 及 QTc 间期比较（$\bar{x}\pm s$）

组别	n	心率（次 /min）		QTd 间期（ms）		QTc 间期（ms）	
		治疗前	治疗后	治疗前	治疗后	治疗前	治疗后
观察组	30	95.64± 10.43	74.19± 8.27	59.22± 16.81	41.53± 15.84	38.65± 10.02	48.33± 7.09
对照组	30	95.36± 10.28	79.85± 6.14	58.92± 17.23	49.82± 15.31	38.42± 10.27	43.69± 7.82
t		0.105	3.010	0.068	2.061	0.088	2.408
P		0.917	0.004	0.946	0.044	0.930	0.019

2.3　两组不良反应发生情况比较　观察组服药期间未出现明显不良反应；对照组出现 2 例恶心，4 例窦性心动过缓，不良反应发生率为 20.00%（6/30）。观察组不良反应发生率低于对照组，差异有统计学意义（χ^2=4.630，P=0.010）。

3　讨论

冠心病是临床常见的心脏器质性病变，心律失常是冠心病常伴症状。冠心病室性心律失常西医主要采用抗心律失常药物治疗。琥珀酸美托洛尔缓释片属 β_1 肾上腺素能受体阻滞剂，可拮抗 β_1 肾上腺素能受体，逆转交感神经的过度激活，减少 Ca^{2+}、Na^+、K^+ 流失，阻止心律失常发生，但长时间服用该药易引发新的心律失常，患者易产生不良反应，对预后造成不利影响[7~8]。

冠心病心律失常属中医学“胸痹、心悸”范畴，病因为阴虚、气虚、血虚，中医治疗主张滋阴补血，通阳复脉[9]。《伤寒杂病论》中记载：“伤寒脉结代，心动悸，炙甘草汤主之。”本研究结果显示，观察组治疗总有效率较高，不良反应发生率较低，表明与单纯西药治疗相比，炙甘草汤联合西药治疗可获得较好的临床效果，且用药安全性高，有助于促进患者预后改善。炙甘草汤中炙甘草可滋阴增液、通脉利血、甘温益气；人参与大枣联用可补脾养心、补气益肺；麦门冬、火麻仁、生地黄、阿胶可舒筋凉血、滋阴养血；生姜、桂枝可舒经通络、温阳通脉。诸药合用，可奏通阳复脉、益气滋阴之效。现代药理学研究表明麦门冬不仅可营养心肌、抗心律失常，还可增加冠脉血流量，改善心脏功能；炙甘草可抗乙酰胆碱，阻滞 Na^+ 流失；生地黄及人参可增加心输出血量，提高心肌收缩力，对抵抗心肌缺血具有重要作用[10]。观察组治疗后心率较低，QTd 间期较短，QTc 间期较长，提示炙甘草汤联合西药治疗冠心病室性心律失常可更好地稳定患者心率，改善患者 QTd 及 QTc 间期，分析原因为琥珀酸美托洛尔缓释片具有较强的靶向治疗作用，作用效果专一，炙甘草汤是中药汤剂，由各活性物质群组成，可作用于多靶点治疗，表现为多效应性，两者联合治疗时可有效控制患者心率失常症状，使患者心率得以稳定，QTd 及 QTc 间期得以改善，并逐渐趋于正常水平。综上所述，炙甘草联合琥珀酸美托洛尔缓释片治疗冠心病室性心律失常临床效果明显，可有效稳定患者心率，改善患者室性早搏等临床症状，同时缩短 QTd 间期，延长 QTc 间期，安全可靠。

参考文献

[1]苗灵娟,杨永枝,邢海燕,等.炙甘草汤联合胺碘酮治疗气阴两虚冠心病室性心律失常疗效观察[J].陕西中医,2017,38(2):169-170
[2]吴利娜,秦建宁,丁辉.美托洛尔联合炙甘草汤加减治疗气阴两虚型冠心病心律失常疗效及安全性评价[J].长春中医药大学学报,2018,34(6):1193-1196
[3]吕冬,曾维富.通心络胶囊联合美托洛尔治疗冠心病室性心律失常的临床疗效观察[J].实用心脑肺血管病杂志,2016,24(11):79-82
[4]中国医师协会急诊医师分会,中华医学会心血管病学分会,中华医学会检验医学分会.急性冠脉综合征急诊快速诊疗指南[J].中华急诊医学杂志,2016,25(4):397-404
[5]王永刚,齐婧,钟伟,等.冠心病中医病因病机的认识与探索[J].中医杂志,2015,56(17):1449-1452
[6]叶先龙.活血定痛汤联合西药治疗心脉瘀阻型冠心病心绞痛的临床效果及心功能分析[J].实用中西医结合临床,2016,16(8):14-16
[7]黄元桃.琥珀酸美托洛尔缓释片治疗心梗后室性心律失常的临床研究[J].实用临床医药杂志,2017,21(13):159-160
[8]卓书江,李光智,王转转,等.卡维地洛联合美托洛尔治疗老年高血压合并室性心律失常的疗效及对血清 hs-CRP 的影响[J].中西医结合心脑血管病杂志,2018,16(2):193-195
[9]齐晓贵,徐海波,辛凤萍,等.美托洛尔对高血压性心脏病患者不同时间段的 QT 间期离散度及室性心律失常发生率影响研究[J].山西医药杂志,2017,46(22):2763-2765
[10]陈超.复方罗布麻片结合通冠复脉汤治疗冠心病室性心律失常疗效观察[J].现代中西医结合杂志,2018,27(17):1852-1854,1892

（收稿日期：2019-05-07）

doi:10.13729/j.issn.1671－7813.Z20191251

炙甘草汤加减治疗气阴两虚型心律失常临床观察

刘孟珍，杨京梅
（北京市通州区宋庄卫生院，北京 101118）

摘要：目的 探讨炙甘草汤加减治疗气阴两虚型心律失常的临床疗效。**方法** 选取医院 2016 年 9 月—2018 年 9 月诊治的气阴两虚型心律失常患者 142 例，采用随机数字表法分为两组，对照组 71 例采用酒石酸美托洛尔治疗，观察组 71 例采用酒石酸美托洛尔联合炙甘草汤加减治疗，于治疗前后行临床症状评分、心功能检测，比较两组临床疗效、不良反应。**结果** 两组治疗后临床症状评分较治疗前显著降低（$P<0.05$）。两组治疗后左室射血分数、心脏指数、心排血量较治疗前显著增加，差异有统计学意义（$P<0.05$）。观察组治疗后临床症状评分低于对照组（$P<0.05$）。观察组治疗后左室射血分数、心脏指数、心排血量高于对照组（$P<0.05$）。观察组总有效率高于对照组（$P<0.05$）。不良反应比较，差异无统计学意义（$P>0.05$）。**结论** 炙甘草汤加减治疗气阴两虚型心律失常的疗效显著，可改善临床病症，提高心功能。

关键词：炙甘草汤加减；气阴两虚型；心律失常；酒石酸美托洛尔；临床症状评分；心功能；临床疗效；不良反应；冠状动脉

引用标注格式：刘孟珍，杨京梅．炙甘草汤加减治疗气阴两虚型心律失常临床观察[J]．实用中医内科杂志，2020，34(7)：135－138．

中图分类号：R541.7　　**文献标志码：**B　　**文章编号：**1671-7813(2020)07-0135-04

Clinical Effect Observation of Modified *Zhigancao* Decoction in Treatment for Arrhythmia of Deficiency of Both Qi and Yin Type

LIU Mengzhen, YANG Jingmei
(Songzhuang Health Center in Tongzhou District, Beijing 101118, China)

Abstract: ***Objective*** To investigate clinical effect of Modified *Zhigancao* decoction in treatment for arrhythmia of deficiency of both Qi and Yin type. ***Methods*** A total of 142 patients with arrhythmia of deficiency of both Qi and Yin type were selected in hospital from September 2016 to September 2018, and were divided into two groups by random number table method. Seventy－one patients treated metoprolol tartrate were as the control group. Seventy－one patients treated with metoprolol tartrate and Modified *Zhigancao* decoction were as the observation group. Clinical symptom scores and cardiac function were measured before and after treatment. Clinical efficacy and adverse reactions were compared between two groups. ***Results*** Clinical symptom scores decreased significantly in two groups after treatment ($P<0.05$). Left ventricular ejection fraction, cardiac index and cardiac output increased significantly in two groups after treatment ($P<0.05$). Clinical symptom scores in the observation group were lower than those of the control group after treatment ($P<0.05$). Left ventricular ejection fraction, cardiac index and cardiac output in the observation group were higher than those of the control group after treatment ($P<0.05$). The total effective rate in observation group was higher than that of the control group ($P<0.05$). There were no significant differences in adverse reactions between two groups ($P>0.05$). ***Conclusion*** Modified *Zhigancao* decoction has significant effect in treatment for arrhythmia of deficiency of both Qi and Yin type, which also can improve clinical symptoms and heart function.

Keywords: Modified *Zhigancao* decoction; deficiency of both Qi and Yin; arrhythmia; metoprolol tartrate; clinical symptom score; cardiac function; clinical efficacy; adverse reaction; coronary artery

心律失常是临床常见病，好发于中老年人[1-2]，如果不能给予对症处理，有可能进一步发展，而诱发冠心病，危及患者的生命，危害极大。中医认为[3-4]心律失常为本虚标实之证，以虚为主，即患者存在不同程度的气血亏虚、阴阳亏虚，与机体气血运行不畅、心神失养等密切相关。因而治疗方案中，可对症用药。本研究探讨了炙甘草汤加减治疗气阴两虚型心律失常的临床疗效，发现炙甘草汤加减治疗气阴两虚型心律失常的疗效显著，可改善临床病症并提高心功能，现报道如下。

作者简介：刘孟珍（1971－），女，北京人，副主任医师，学士，研究方向：中医内科。

1 资料与方法

1.1 一般资料

选取北京市通州区宋庄卫生院2016年9月—2018年9月诊治的气阴两虚型心律失常患者142例，采用随机数字表法分为两组，对照组71例，男41例，女30例，年龄51～72岁，平均(64.3±5.2)岁，病程2～13年，平均(5.8±2.6)年；观察组71例，男39例，女32例，年龄52～73岁，平均(64.8±5.1)岁，病程2～12年，平均(5.7±2.8)年。两组性别、年龄、病程等一般资料比较，差异无统计学意义(P>0.05)，具有可比性。

1.2 入选标准

符合冠心病的诊断标准[5]；心电图提示心律失常；中医辨证分型为气阴两虚型；同意本次研究；经医院伦理委员会批准。

1.3 排除标准

患有器官功能障碍；精神性疾病；药物过敏。

1.4 治疗方法

1.4.1 对照组 酒石酸美托洛尔(阿斯利康制药有限公司，国药准字H32025390)，25 mg/次，2次/d，疗程4周。

1.4.2 观察组 酒石酸美托洛尔联合炙甘草汤加减治疗，酒石酸美托洛尔的用法同对照组，炙甘草汤加减的方案如下：炙甘草30 g，生地黄30 g，党参30 g，桂枝10 g，阿胶10 g，麦冬10 g，麻仁10 g，生姜6 g，五味子15 g，大枣10枚。临证加减，心阳不足者加炮附片10 g，心悸不宁、夜寐不安、出汗者加龙骨30 g，牡蛎30 g，远志15 g，酸枣仁20 g，心脾不足者加黄芪20 g，白术15 g。每日1剂，分早晚两次服用，疗程4周。

1.5 观测指标

临床症状评分(心悸、乏力、气短、失眠、胸闷)、心功能(左室射血分数、心脏指数、心排血量)，临床疗效(显效、有效、无效、总有效)、不良反应(恶心呕吐、便秘、房室传导阻滞)。

1.6 疗效判定

临床症状评分参照《中药新药临床研究指导原则》[6]，内容包括心悸、乏力、气短、失眠、胸闷，采用0～3分评定，分数越高，临床症状越严重。临床疗效评定标准[7]，显效：临床症状消失，中医症状积分、心功能改善幅度≥90%；有效：临床症状改善，中医症状积分、心功能改善幅度≥50%；无效：临床症状无改善，中医症状积分、心功能改善幅度<50%。

1.7 统计分析

采用SPSS19.0软件行统计学分析，计量资料用均数±标准差($\bar{x}\pm s$)表示和t检验，计数资料用率(%)表示和χ^2检验，P<0.05为差异有统计学意义。

2 结果

2.1 临床症状评分

治疗后，两组临床症状评分(心悸、乏力、气短、失眠、胸闷)显著降低(P<0.05)；观察组低于对照组(P<0.05)。见表1。

表1 两组临床症状评分比较(分，$\bar{x}\pm s$)

组别	例数	时间	心悸	乏力	气短	失眠	胸闷
对照组	71	治疗前	2.26±0.28	2.08±0.46	2.11±0.17	1.99±0.36	2.15±0.14
		治疗后	1.17±0.13	1.25±0.19	1.22±0.08	1.15±0.23	1.20±0.12
		t值	29.752	14.052	39.915	16.568	43.412
		P值	0.000	0.000	0.000	0.000	0.000
观察组	71	治疗前	2.24±0.31	2.05±0.42	2.09±0.15	2.01±0.24	2.13±0.18
		治疗后	0.59±0.10	0.67±0.18	0.73±0.11	0.48±0.07	0.54±0.09
		t值	42.683	25.447	61.607	51.568	66.573
		P值	0.000	0.000	0.000	0.000	0.000
组间比较		t值	29.798	18.673	30.356	23.482	37.075
		P值	0.000	0.000	0.000	0.000	0.000

2.2 心功能

治疗后，两组左室射血分数、心脏指数、心排血量显著增加(P<0.05)；观察左室射血分数、心脏指数、心排血量高于对照组(P<0.05)。见表2。

表2 两组心功能比较($\bar{x}\pm s$)

组别	例数	时间	左室射血分数(%)	心脏指数(L/min·m^2)	心排血量(mL/min)
对照组	71	治疗前	54.23±5.84	2.54±0.29	4.28±0.85
		治疗后	59.98±6.12	2.96±0.34	4.86±0.72
		t值	5.728	7.919	4.387
		P值	0.000	0.000	0.000
观察组	71	治疗前	54.19±6.25	2.51±0.34	4.27±0.73
		治疗后	65.24±7.01	3.67±0.42	5.45±0.69
		t值	9.914	18.088	9.898
		P值	0.000	0.000	0.000
组间比较		t值	4.763	11.071	4.985
		P值	0.000	0.000	0.000

2.3 临床疗效

治疗后，观察组总有效率高于对照组(P<0.05)。见表3。

表3 两组临床疗效比较[例(%)]

组别	例数	显效	有效	无效	总有效
对照组	71	36(50.70)	27(38.03)	8(11.27)	63(88.73)
观察组	71	57(80.28)	13(18.31)	1(1.41)	70(98.59)
χ^2					5.813
P值					0.016

2.4 不良反应

不良反应(恶心呕吐、便秘、房室传导阻滞)比较，差异无统计学意义(P>0.05)。见表4。

表4 两组不良反应比较[例(%)]

组别	例数	恶心呕吐	便秘	房室传导阻滞
对照组	71	1(1.41)	2(2.82)	1(1.41)
观察组	71	3(4.23)	1(1.41)	0(0.00)
χ^2		1.029	0.341	1.007
P值		0.310	0.560	0.316

3 讨论

近年来，随着饮食结构改变、社会压力增加、人口老龄化发展，心律失常病症呈现出明显的增加态

势[8-9]，严重影响着公众的身心健康，已引起社会的广泛关注。心律失常是冠心病的常见并发症[10-11]，也是造成患者猝死的常见病因[12-13]，因而，对于该病，应该早诊断、早治疗。西医用药中，美托洛尔较为有效，这是一种选择性 β-受体阻滞药，无内源性拟交感活性，可通过降低心脏做功达到减少心肌需氧的作用，还可延长心肌不应期、窦房结恢复时间、右房至希氏束的传导时间和房室结不应期的电生理特性，效果显著[14-15]。但单独应用西药治疗，有可能产生不良反应，还可能存在一定程度剂量依赖性，应该予以重视[16-17]。

中医学者认为[18-19]冠心病心律失常的病机在于气血不畅、阴阳不足，可通过活血化瘀、益气养阴治疗，将最大限度改善患者的预后，效果显著。本研究的结果显示，两组治疗后临床症状评分（心悸、乏力、气短、失眠、胸闷）较治疗前显著降低；两组患者治疗后左室射血分数、心脏指数、心排血量较治疗前显著增加；观察组治疗后临床症状评分低于对照组。观察组治疗后左室射血分数、心脏指数、心排血量高于对照组；观察组患者总有效率高于对照组。不良反应（恶心呕吐、便秘、房室传导阻滞）比较，差异无统计学意义，说明炙甘草汤加减治疗气阴两虚型心律失常的疗效显著，可改善临床病症，提高心功能。

炙甘草汤加减方中，炙甘草甘温益气、通经脉利气血、取其益气滋阴、通阳复脉之功为主药，党参补脾益肺、补血生津增加炙甘草益气生津之功，生地黄滋阴清热、凉血补血，桂枝温通经脉、助阳化气，阿胶补血滋阴、润燥止血，麦冬养阴润肺、益胃生津、清心除烦，麻仁润肠通便、疏通血脉，生姜辛散温通助桂枝温通经脉之功，五味子益气生津、补肾宁心、收敛心气，大枣补中益气、养血安神，诸药合用共奏阴阳调和、益气滋阴、补血复脉之功[20-21]。

这与诸多研究结果相似。曾文新[7]研究结果显示观察组总有效率高于对照组。观察组心悸症状消失时间、气短消失时间、乏力消失时间均短于对照组，两组中医症状积分、左室舒张末期内径、左室收缩末期内径、左室射血分数均优于治疗前，且观察组上述指标优于对照组，提示胺碘酮联合炙甘草汤加减治疗冠心病心律失常的疗效优于单用胺碘酮的疗效，且心悸、气短、乏力症状的消失时间较短，对于心功能改善的效果更加。赵明哲等[20]研究结果显示实验组冠心病心律失常患者治疗的整体总有效率明显高于对照组，实验组心律失常总有效率明显高于对照组，提示冠心病心律失常患者接受炙甘草加减与西药联合治疗，可提高其治疗效果，改善预后。俞军山[21]研究结果显示实验组治疗效果显著优于参照组，实验组不良反应少于参照组，提示炙甘草汤加减治疗针对性治疗冠心病合并心律失常患者的临床症状较好，值得临床中的推广以及应用。陈小刚[22]研究结果显示治疗后与对照组相比，试验组总有效率更高，提示联用盐酸胺碘酮和炙甘草汤加减方治疗冠心病合并心律失常的临床效果确切。王强[23]研究结果显示观察组临床疗效显著高于对照组，观察组治疗后室性期前收缩朗氏分级情况明显优于对照组，观察组不良反应发生率显著低于对照组，提示炙甘草汤加减治疗在室性心律失常中具有较好的临床疗效，能够有效改善临床症状，减少不良反应，值得推广使用。张延庆[24]研究结果显示，对照组和观察组总有效率存在明显差异，且心悸积分、气短积分以及神倦乏力积分也存在明显的差异，观察组更优，提示针对冠心病合并心律失常患者使用炙甘草汤加减法进行治疗，临床效果显著。张美弟[25]研究显示观察组总有效率高于对照组，提示采用炙甘草汤加减联合常规西药治疗气血两虚型冠心病心律失常患者疗效确切，不良反应少，具有重要的临床应用价值。王炳辉[26]研究显示试验组治疗后乳酸脱氢酶（LDH）、肌酸激酶（CK）、天门冬氨酸氨基转移酶（AST）水平均优于对照组，试验组总有效率优于对照组，提示炙甘草汤加减联合曲美他嗪治疗缓慢性心律失常临床效果显著。温万春[27]研究显示对照组的治疗总有效率与治疗组比较，治疗组高于对照组，提示冠心病心律失常患者采用炙甘草汤加减方法治疗有利于显著改善临床症状，提高治疗效果，具有积极的临床使用和推广意义。

综上，炙甘草汤加减可扩张患者冠状动脉，发挥抗心律失常、抗心肌缺血的作用，能有效增强机体心肌收缩功能，疗效显著。但此次研究的样本量有限，可进一步扩大样本量，以提高研究价值。

参考文献

[1] 阳成俊，刘青平. 动态心电图在老年冠心病心肌缺血和心律失常患者诊断中的应用[J]. 医疗装备，2018，31(24)：131-132.

[2] 李春华. 培哚普利联合美托洛尔治疗老年心力衰竭合并室性心律失常效果分析[J]. 中华心脏与心律电子杂志，2018，6(4)：225-227.

[3] 刘平艳. 探讨利用中医方法治疗冠心病心律失常所取得的临床效果[J]. 中医临床研究，2018，10(34)：99-100.

[4] 杨毓强. 中医辨证联合稳心颗粒治疗冠心病心律失常的疗效观察[J]. 中国社区医师，2019，35(12)：106-107.

[5] 中华医学会心血管病学分会，中华心血管病杂志编辑委员会. 冠心病诊断与治疗指南[J]. 中华心血管病杂志，2007，35(3)：195-206.

[6] 郑筱萸. 中药新药临床研究指导原则[S]. 北京：中国医药科技出版社，2002.

[7] 曾文新. 炙甘草汤加减治疗冠心病心律失常的临床价值分析[J]. 中国实用医药，2019，14(13)：122-124.

[8] KIM J，KIM H. Influence of ambient temPerature and diurnal temPerature range on incidence of cardiac arrhythmias[J]. International Journal of Biometeorology，2017，61(3)：407-416.

[9] KHARBANDA R K，BLOM N A，HAZEKAMP M G，et al. Incidence

and risk factors of Post - oPerative arrhythmias and sudden cardiac death after atrioventricular sePtal defect (AVSD) correction: UP to 47, years of follow - uP[J]. International Journal of Cardiology, 2018,252(1):88 - 93.
[10] 张娇,许鹏,张清涛,等. 胺碘酮联合美托洛尔治疗冠心病合并心律失常临床效果观察[J]. 解放军医药杂志,2018,30(10):70 - 72.
[11] 凌克,李华,冯明,等. 老年2型糖尿病合并冠心病患者心率变异性与心律失常的关系[J]. 河南医学研究,2018,27(9):1553 - 1556.
[12] 黄波,周菁. 致心律失常性右室心肌病室性心律失常干预策略[J]. 实用心电学杂志,2019,28(2):127 - 130.
[13] 闫丽荣,陈柯萍,戴研,等. 埋藏式心脏复律除颤器在肥厚型心肌病心脏性猝死高危患者一级预防中的临床疗效分析[J]. 中国循环杂志,2019,34(3):256 - 260.
[14] 陈亚君,乔雯. 胺碘酮和美托洛尔治疗室性心律失常患者的疗效观察[J]. 实用中西医结合临床,2018,18(11):14 - 16.
[15] 夏琨,孙东明,张勇,等. 美托洛尔对急性心律失常患儿的疗效及对血清心肌酶和炎症因子的影响[J]. 东南大学学报(医学版),2018, 37(6):1037 - 1040.
[16] 单海慧,张丽红. 美托洛尔治疗老年高血压性心脏病室性心律失常的疗效[J]. 中国老年学杂志,2018,38(12):2857 - 2858.
[17] 孙希鹏,诸国华,罗鸿宇,等. 一项多中心、随机、双盲、平行对照临床研究——评价酒石酸美托洛尔片联合通脉养心丸治疗室性早搏的有效性和安全性[J]. 首都医科大学学报,2019,40(3):389 - 395.
[18] 刘平艳. 探讨利用中医方法治疗冠心病心律失常所取得的临床效果[J]. 中医临床研究,2018,10(34):99 - 100.
[19] 王月,侯平. 参仙升脉口服液治疗冠心病合并缓慢性心律失常的效果分析[J]. 山西医药杂志,2018,47(17):1999 - 2002.
[20] 赵明哲,陆晓亚. 炙甘草汤加减联合西药治疗冠心病心律失常的效果评价[J]. 中西医结合心血管病电子杂志,2018,6(27):174 - 175.
[21] 俞军山. 炙甘草汤加减治疗冠心病合并心律失常的效果观察[J]. 中西医结合心血管病电子杂志,2018,6(27):164 - 165.
[22] 陈小刚. 联用盐酸胺碘酮和炙甘草汤加减方治疗冠心病合并心律失常的效果研析[J]. 当代医药论丛,2019,17(7):229 - 230.
[23] 王强. 炙甘草汤加减治疗室性心律失常的效果及不良反应发生率分析[J]. 中国农村卫生,2018,145(19):58 - 59.
[24] 张延庆. 炙甘草汤加减治疗冠心病合并心律失常的临床效果评价[J]. 世界最新医学信息文摘,2018,18(53):168 - 171.
[25] 张美弟. 炙甘草汤加减联合常规西药治疗气血两虚型冠心病心律失常的临床疗效观察[J]. 中国现代药物应用,2019,13(19):109 - 110.
[26] 王炳辉. 炙甘草汤加减联合曲美他嗪治疗缓慢性心律失常的临床疗效分析[J]. 中国现代药物应用,2018,12(3):67 - 68.
[27] 温万春. 冠心病心律失常患者采用炙甘草汤加减方法治疗的效果评价[J]. 中西医结合心血管病杂志(电子版), 2018,6(1):143 - 145.

(上接第118页)

参考文献

[1] 崔莹. 益气养阴法对心律失常病人心脏功能的影响[J]. 中西医结合心脑血管病杂志,2019,17(11):1664 - 1667.
[2] 刘亚军,宋鑫. 美托洛尔对冠心病心绞痛病人心肌缺血总负荷及心率变异性的影响[J]. 中西医结合心脑血管病杂志,2019,17(12):1869 - 1871.
[3] 董慧琴. 温阳复脉汤联合富马酸比索洛尔治疗冠心病心律失常55例[J]. 中国中医药科技,2019,26(2):289 - 290.
[4] 曹腊梅,张建春,张宝田,等. 养心定悸法对心律失常伴失眠病人睡眠质量及5 - HT、DA的影响[J]. 中西医结合心脑血管病杂志,2019,17(13):2011 - 2014.
[5] 车晓倩. 自拟稳心定悸汤联合美托洛尔治疗室性早搏50例[J]. 浙江中医杂志,2019,54(4):279.
[6] 王胜强,赵爱荣,李吉刚. 养心通汤联合西医治疗冠心病冠状动脉介入术后的临床疗效及对病人炎性因子的影响[J]. 中西医结合心脑血管病杂志,2018,16(16):2355 - 2357.
[7] 李海良,张克清. 参芪复脉汤联合西药治疗慢性心衰疗效及对患者心功能的影响[J]. 陕西中医,2019,40(2):163 - 166.
[8] 李蒙,姜钧文,徐刚. 稳心复律汤联合缬沙坦治疗高血压合并快速性心律失常疗效及其对血压和心率变化的影响[J]. 中国中医药科技,2019,13(2):289 - 290.
[9] 李海滨,李征,胡月,等. 灯盏生脉胶囊治疗冠心病及慢性心力衰竭的系统评价[J]. 中西医结合心脑血管病杂志,2019,17(7):967 - 971.
[10] 张雨晴,何庆勇,刘旭东,等. 基于国家专利数据库的治疗心律失常的中药配伍规律的数据挖掘研究[J]. 世界科学技术—中医药现代化,2018,21(3):383 - 388.
[11] 徐向宇. 参松养心胶囊联合美托洛尔治疗冠心病心律失常疗效评价[J]. 上海医药,2019,40(4):21 - 23.
[12] 李嵩,程子轩. 参松养心胶囊联合美托洛尔治疗冠心病心律失常有效性及安全性的Meta分析[J]. 世界中西医结合杂志,2019,14(6):761 - 767.
[13] 程航,彭锴. 依那普利联合硝苯地平或美托洛尔对高血压合并稳定型心绞痛病人血压、内皮功能的影响[J]. 中西医结合心脑血管病杂志,2019,17(12):1906 - 1908.
[14] 宋梦莹,刘莹,邓达治. 参松养心胶囊联合酒石酸美托洛尔治疗冠心病合并心律失常的临床疗效及安全性[J]. 医学综述,2019,25(1):184 - 188.
[15] 祖德金,张兰芹. 美托洛尔联合芪参益气滴丸治疗慢性心力衰竭合并心房颤动的临床疗效[J]. 中西医结合心脑血管病杂志,2019,17(8):1226 - 1228.
[16] 付玲,邹飞虎,罗玲. 定悸复脉汤抗室性心律失常的疗效观察[J]. 中国中医急症,2019,28(6):1073 - 1075.
[17] 崔萌萌,姚淮芳. 室上性快速型心律失常的中医药治疗研究进展[J]. 中医药临床杂志,2018,30(1):165 - 167.
[18] 韩芬,戴国华,郑文光,等. 386例冠心病心律失常住院患者中医药治疗情况调查分析[J]. 西部中医药,2015,28(3):86 - 89.
[19] 庞庆荣,陈守强,毕文霞,等. 名老中医治疗快速性心律失常用药规律的varclus聚类分析[J]. 世界中西医结合杂志,2015,10(6):748 - 750.
[20] 居励之,樊华,王佑华. 快速型心律失常的中医药研究进展[J]. 中西医结合心脑血管病杂志,2019,17(11):1642 - 1644.

美托洛尔联合炙甘草汤加减治疗气阴两虚型冠心病心律失常的效果观察

梅永现
（元氏县医院，河北 石家庄 051130）

【摘要】目的：观察与研究美托洛尔联合炙甘草汤加减治疗气阴两虚型冠心病心律失常的效果。方法：纳入我院 2017 年 10 月 -2019 年 10 月期间 64 例气阴两虚型冠心病心律失常患者，以"随机单双号"方式将纳入主体分为两组，单号 32 例为对比组给予美托洛尔治疗，双号 32 例为研究组采取美托洛尔联合炙甘草汤加减治疗，对比治疗效果。结果：研究组总有效率（96.88%）高于对比组（81.25%），差异显著，P ＜ 0.05；研究组各项症状评分均低于对比组，差异显著，P ＜ 0.05。结论：给予气阴两虚型冠心病心律失常患者美托洛尔联合炙甘草汤加减治疗，治疗效果显著，有助于改善患者症状评分，可在临床中大力推广与应用。

【关键词】美托洛尔；炙甘草汤；气阴两虚型；冠心病心律失常；治疗效果
【中图分类号】R259 【文献标识码】A 【文章编号】2095−4808（2020）09−170−01

冠状动脉粥样硬化心脏病是临床常见疾病，并且近年来患病率呈现上升趋势，冠心病可致多种心律失常，可出现心动过速、心动过缓、心律不齐等多种症状，不仅会影响患者心功能，降低患者的生活质量，严重时还会造成患者死亡，危及生命安全，因此，及时有效的治疗对患者具有重要的现实意义[1]。本次研究纳入 64 例气阴两虚型冠心病心律失常患者，采取分组方式进行研究，实施不同治疗，旨在提高患者临床受益。

1. 资料与方法

1.1 一般资料，纳入我院 2017 年 10 月 –2019 年 10 月期间 64 例气阴两虚型冠心病心律失常患者，以"随机单双号"方式将纳入主体分为两组，单号 32 例为对比组：男 17 例、女 15 例，年龄 35–78 岁，均值数（51.8 ± 7.4）岁；双号 32 例为研究组：男 18 例、女 14 例，年龄 34–77 岁，均值数（52.2 ± 7.5）岁；纳入研究对象过程中剔除对研究所选药物过敏，以及配合度、依从性较差患者，对两组患者基本资料信息用统计学软件计算差异较小，P ＞ 0.05，研究可行。

1.2 方法，单号 32 例为对比组，给予美托洛尔治疗，治疗过程：酒石酸美托洛尔缓释片，每次 25–50mg，每天 2–3 次，共治疗 20 天。双号 32 例为研究组，采取美托洛尔联合炙甘草汤加减治疗，治疗过程：美托洛尔用药剂量、次数、时间均与对比组一致，炙甘草汤基础处方：人参 6g、阿胶 6g、生姜 10g、桂枝 10g、麦门冬 12g、麻仁 12g、炙甘草 15g、干地黄 20g、大枣 10 颗。对于睡眠质量较差患者加合欢皮、酸枣仁各 10g；对于胸闷腹痛情况严重患者加半夏、延胡索各 6g；对于水肿尿少患者加茯苓、葶苈子各 10g；上述药物水煎服，每日一剂，分两次服用，共治疗 20 天。

1.3 观察指标，对比组、研究组治疗效果和中医症状评分观察与分析，其中，显效：各项临床症状与表现基本全部消失，心律失常发作次数、时间减少超过 90%；有效：各项临床症状与表现较治疗前有所缓解，心律失常发作次数、时间减少在 50%–89% 之间；无效：各项临床症状与表现、心律失常发作次数、时间与治疗前相比无任何改善迹象，甚至有加重迹象[2]。中医症状评分每项 0–3 分，包括胸闷、胸痛、心悸、乏力，分值高，症状严重[3]。

1.4 统计学分析，研究数据资料用 SPSS24.0 软件处理，计量资料（$\bar{x} \pm s$），行 t 检验；计数资料（n，%），行 x2 检验，P ＜ 0.05，有统计学意义。

2. 结果

2.1 治疗效果对比，研究组总有效率（96.88%）高于对比组（81.25%），差异显著，P ＜ 0.05，见表 1。

表 1 治疗效果对比（n，%）

组别	例数（n）	显效	有效	无效	有效率（%）
对比组	32	14	12	6	（26）81.25%
研究组	32	21	10	1	（31）96.88%
x2	--	--	--	--	4.0100
P	--	--	--	--	0.0452

2.2 中医症状评分对比，研究组各项症状评分均低于对比组，差异显著，P ＜ 0.05，见表 2。

表 2 中医症状评分对比（$\bar{x} \pm s$）

组别	例数（n）	胸闷	胸痛	心悸	乏力
对比组	32	1.22 ± 0.13	1.27 ± 0.12	1.21 ± 0.11	1.19 ± 0.10
研究组	32	0.78 ± 0.09	0.79 ± 0.08	0.77 ± 0.07	0.74 ± 0.08
t	--	15.7419	18.8271	19.0899	19.8777
P	--	0.0000	0.0000	0.0000	0.0000

3. 讨论

冠心病心律失常主要是由心脏起搏、传导系统异常等因素造成，目前，临床治疗本病以抗心律失常药物为主，旨在缓解疾病进展，其中，美托洛尔是常用药物，属于 β 受体阻滞剂，当其进入机体后，会选择性阻断 β1 受体，进而减慢心率，延缓房室传导时间，改善心律失常，但是，长期服用该药物会引起其他毒副作用[4]。中医认为，冠心病心律失常属于"胸痹"范畴，大多是由气虚、阴虚造成，治疗应该以益气养阴、活血通络为主，炙甘草汤出自《伤寒论》，是一种气血双补剂，人参具有补气补益、回阳救逆功效；阿胶、麦门冬具有补血滋阴、养脾扶肝功效；桂枝具有温经通阳、消痹化气功效；生姜、地黄具有发表散寒、补血滋阴功效；炙甘草则能够调和诸药，缓和药性，将药物作用发挥最大[5]。

研究结果显示：研究组总有效率（96.88%）高于对比组（81.25%），差异显著，P ＜ 0.05；研究组各项症状评分均低于对比组，差异显著，P ＜ 0.05，说明美托洛尔联合炙甘草汤加减治疗气阴两虚型冠心病心律失常疗效确切。

综上，给予气阴两虚型冠心病心律失常患者美托洛尔联合炙甘草汤加减治疗，治疗效果显著，有助于改善患者症状评分，可在临床中大力推广与应用。

参考文献

[1] 赵金龙．美托洛尔联合炙甘草汤加减方治疗气阴两虚型冠心病心律失常的效果 [J]. 当代医药论丛 ,2019,17(16):205-206.
[2] 万志敏，万美萍．美托洛尔联合炙甘草汤加减治疗气阴两虚型冠心病心律失常的效果观察 [J]. 基层医学论坛 ,2019,23(19):2777-2778.
[3] 王林．炙甘草汤加减联合酒石酸美托洛尔治疗冠心病心律失常的效果及不良反应分析 [J]. 当代医学 ,2019,25(15):166-167.
[4] 吴利娜，秦建宁，丁辉．美托洛尔联合炙甘草汤加减治疗气阴两虚型冠心病心律失常疗效及安全性评价 [J]. 长春中医药大学学报 ,2018,34(06):1193-1196.
[5] 张继磊．炙甘草汤联合美托洛尔治疗冠心病心律失常的疗效观察 [J]. 实用中西医结合临床 ,2018,18(09):17-19.

原发性高血压病中医证素分布组合的特征探讨

王爱青
（山东省曹县中医院，山东 菏泽 274400）

【摘要】目的：探讨在高血压病症中中医证素分布组合的特征，以此指导中医临床辩证。方法：对我院在 2017 年 2 月至 2019 年 12 月高血压患者调查，根据调查的结果建立数据，分析高血压病患者中医证素组合分布情况，高血压病症患者单证素和 2 个证素相兼组合分布情况。结果：高血压患者中两个证素相兼数最为常见，在占的比例中可以高达 60.4%，其次是单证素，占比也高达 21.6%；两个证素相兼中较高的是气血 + 血瘀和阳亢 + 气虚，这个占比方面，阳亢 + 阴虚占比在 19.4%，气虚 + 血淤占比上在 16.4%。结论：中医证素组合分布的情况来看，高血压患者两个证素相兼数最为常见。

【关键词】原发性高血压；临床分布；中医证素
【中图分类号】R544 【文献标识码】A 【文章编号】2095−4808（2020）09−170−02

高血压病是指由于体循环动脉血压升高所致的一种综合征，常见头晕、头痛、胸闷、乏力等症为主要临床特征，可并发心脑肾等脏器不同程度的器质性损害，预后不良。现结合临床体会，对原发性高血压病中医证素分布组合的特征探讨如下。

1. 资料和方法

1.1 一般资料，对我院在 2017 年 2 月至 2019 年 12 月收治的高血压患者进行调查，收集患者 120 例，其中有 9 例诊断中没有明确的中医证候诊断病例，合格的病例数为 111 例，占总体比例的 92.5%。年龄最大 84 岁，最小在 39 岁，平均年龄（45.6 ± 6.8）岁。有吸烟病史患者 41 例，无吸烟病史患者 49 例，被动吸烟患者 21 例；高血压患者最长病史 55 年，最短病史 30 天，平均（16.2 ± 1.）年。

1.2 方法

1.2.1 病例选择

1.2.1.1 西医诊断，参照《2004 年中国高血压防治指南》（实用本）临床诊断为高血压病。（2）中医诊断参照《中医病证诊断疗效标准》诊断为眩晕、头疼等。

1.2.2.2 症状和体征判别 根据《中国中医学主题词表》和《中医临床诊断术语》，结合文献查阅高血压压患者临床表现特征，结合专家论证和咨询，获得高血压 36 个特征和症状：中医兼症包括：苔黄、舌红、面红、急躁易怒、痛有定处、面色晦黯、肌肤甲错、舌质黯或有瘀斑（点）、肢重体麻、便溏而尿清长、舌淡苔润、脉细数、舌红少苔、五心烦热、脉细、起立时眼前昏暗、舌胖或有齿印、少气懒言、神疲乏力等；中医主症包括头痛、头晕。

1.3 统计学方法，采用 SPSS13.0 统计软件，应用中医证素组合方法进行统计描述。P ＜ 0.05 为差异有统计学意义。

2. 结果

中医证素组合分布的情况来看，高血压患者中两个证素相兼数最为常见。在占的比例中可以高达 60.4%，除了这个占主要情况，其次是单证素，占比也高达 21.6%，一般的情况中很难有着四个证素情况出现。根据表 2 情况分析，在单一证候要素中，主要出现频率的是阴虚证素，出现率高达 33.3%，其次是阳亢和痰浊，这两个在占比上高达 20.8%。两个证素相兼中较高的是气血 + 血瘀和阳亢 + 气虚，这个占比方面，阳亢 + 阴虚占比在 19.4%，气虚 + 血淤占比上在 16.4%。

3. 讨论

高血压病属中医的"眩晕"、"头痛"等范畴，高血压病的病因主要为长期精神紧张或恼怒忧思，致肝郁化火；恣食肥甘或饮酒过度，致痰浊内生；劳欲过度或年老体衰，致肝肾亏损。上述病因使肝肾阴阳失调，邪于清窍发为本病。病理变化为肝气郁滞，郁久化火，肝阳上亢，风阳升动，上扰清窍，而见头痛、眩晕、急躁易怒、口苦等症。饮食不节，脾胃损伤，聚湿生痰，痰浊内阻，清阳不升，浊阴不降，胸阳不展，而见头晕、头重、胸闷等症。劳欲伤肾或年老肾亏，肾精亏耗，肝肾阴虚，阴不敛阳，虚阳上扰，而见头隐痛、眩晕、耳鸣、腰酸等症。病程迁延，久病入络，瘀血内停，阻滞脉络，血脉瘀滞，可见头痛如针刺，或胸闷刺痛，手足麻木，舌青紫。

（下转第 172 页）

cm^2、(0.8±1.2) cm^2。上述四类节软骨缺经过测量尺评估的结果分别是 (4.3±1.6) cm^2、(6.0±2.0) cm^2、(3.0±2.0) cm^2、(3.0±1.1) cm^2，术中测量平均值是 6.0cm^2，平均差值是 0.6～3.0cm^2，与 MRI 测量的结果比较有统计学意义，$P < 0.05$。具体测量 120 处软骨缺损的过程中，有 80 处的术中测量结果高于 MRI 测量的结果，20 处的两种测量结果基本一致无显著差异，另 20 处术中测量的结果小于 MRI 的测量结果。

3. 讨论

随着我国医疗技术水平不断提高，临床中磁共振软骨成像术已经取得了显著的发展，而临床中对于软骨特定序列、技术等报道也越来越多，并且不断更新，这些新的技术甚至为临床医师提供了软骨生化状态定量信息。但在实际操作中，我国临床中目前仍然选择标准序列与常规技术作为检测软骨缺损面积的主要手段，有部分资料提示新的技术虽然已经逐步得到应用，但是实际的应用效果也需要通过一定的时间来验证，而术前通过 MRI 检查，来预测软骨缺损面积也还未具体明确其检测结果的准确度。在本次研究中，所选的常规 MRI 软骨检测序列预测软骨缺损面积，在技术与设备方面的要求不高，所以该方法可以作为一种普遍性的检查方式但是相对于关节镜下检查，可以更佳清晰地观察到患者的软骨变性，提高测量的准确性

通过本次研究，MRI 测量软骨缺损的面积值大多低于手术中测量的结果，说明了 MRI 检测到小面积软骨缺损周围大多有严重的剥脱、软骨裂隙以及变性存在，术中清理这些区域后测量的结果往往高于 MRI 的测量结果。这对患者的手术可以提供一些治疗决策，如：患者股骨髁软骨缺损面积低于 3cm^2～4cm^2，可应用微骨折技术进行治疗；患者的缺损面积更大则采取该技术的效果较差，所以更适合选择骨软骨移植，或者软骨细胞移植手术进行治疗。若术前如果 MRI 测量结果显示内侧股骨髁软骨缺损区 4cm^2，则按上述手术的选择方案，主治医师可能会采取微骨折技术治疗，但是根据本此研究的结果，该处的软骨缺损面积大于 4cm^2，所以应考虑采取骨软骨移植，或者软骨细胞移植术治疗。为了降低 MRI 测量系统的误差，所以一般选择 2 经验丰富的影像科医生对膝关节软骨缺损面积进行独立的 MRI 二维图像测量评估。另外，MRI 测量时间和手术时间间隔长，在此期间部分变性软骨组织极有可能继续发展，也会对术中最终测量结果形成影响。为了减少上述影响因素，MRI 图像采集和手术治疗的间隔期应当严格控制与 12 月以内。临床中有多个资料提示，患者软骨缺损面积会随时间推移而逐渐发展，进度大约为每年进展 0.4～4.2%，所以经过 MRI 检测之后需要即可准备手术，这样才能有效减少诊断结果的误差 [3]。

综上所述，虽然 MRI 检测软骨缺损面积，评估值多低于手术中的测量值，但是各解剖部位 MRI 测量值和术中测量值的百分比数据对于术前制定治疗决策有重要意义。

【参考文献】

[1] 陈浩，孙岩，秦卫，等 . 3D-SPACE、3D-TrueFISP 序列测量膝关节软骨缺损面积的比较研究 [J]. 中国中西医结合影像学杂志，2016，14(03):284-286.

[2] 曾哲真 . 术前 MRI 测量评估膝关节软骨缺损面积临床分析 [J]. 中国卫生标准管理，2015，06(31):164-165.

[3] 胡仁健，张水先，林文银 . MRI 测量在膝关节软骨缺损面积评估中的意义 [J]. 重庆医学，2013，42(08):899-900+903.

炙甘草汤、美托洛尔（缓释片）联合治疗冠心病心律失常临床分析

蒲庆平

（盐亭县富驿镇中心卫生院　四川绵阳　621601）

【摘要】目的：探讨炙甘草汤联合美托洛尔治疗冠心病心律失常的效果。方法：选取我院 2015 年 7 月—2016 年 7 月收治的 56 例冠心病心律失常患者为研究对象，将 56 例患者随机均分 A 组和 B 组，A 组采用炙甘草汤联合美托洛尔进行治疗，B 组单纯使用美托洛尔进行治疗，分析两组疗效。结果：A 组治疗效果显著率为 82%（23/28），好转率为 18%(5/28)，无无效者；B 组治疗效果显著率为 50%（14/28），好转率为 25%（7/28），无效率为 25%（7/28）；A 组治疗效果显著率 82% 高于 B 组治疗效果显著率 50%。结论：炙甘草汤联合美托洛尔有效治疗冠心病心律失常患者症状，应推广使用。

【关键词】炙甘草汤、美托洛尔、冠心病、疗效

【中图分类号】R541　【文献标识码】A　【文章编号】1007-8231（2016）28-0060-02

冠心病心律失常即冠状动脉粥样硬化心脏病，心律失常是冠心病的并发症之一，也是主要临床表现之一 [1]。按心律失常的表现形式，冠心病心律失常可以分为窦性心律失常、异位性心律失常以及传导障碍等，冠心病心律失常患者经常性表现为心跳缓慢或停滞、无力、记忆力下降、晕厥等病症，严重者会突发性死亡，因此，研究救治冠心病心律失常患者的药物刻不容缓，本研究主要探讨炙甘草汤联合美托洛尔治疗冠心病心律失常的效果，现报告如下。

1. 一般资料和方法

1.1 一般资料

选取我院 2015 年 7 月—2016 年 7 月收治的 56 例冠心病心律失常患者为研究对象，经我院检查，56 例患者临床病症表现为皮肤灰白、头晕、心悸、记忆下降、心跳缓慢、偶发性晕厥等，经心电图检查，56 例患者 T 波异常、ST 段下移，经诊断，符合冠心病心律失常研究标准。56 例患者中男 32 例，女 24 例，最大年龄 72 岁，最小年龄 61 岁，平均年龄（66±4.7）岁，最长病程 4 年，最短病程 1 年，平均病程（2.5±1.3）年。

将 56 例患者均分 A 组和 B 组。A 组男 16 例，女 12 例，平均年龄（66±4.5）岁，平均病程（2.5±1.4）年。B 组男 16 例，女 12 例，平均年龄（66±4.3）岁，平均病程（2.5±1.5）年。A、B 两组经性别、年龄、病程一般资料比较无明显差异，具有统计学意义（$P > 0.05$）。

1.2 方法

A 组采用炙甘草汤联合美托洛尔进行治疗：

（1）炙甘草汤为中药，最初来自《伤寒论》，由炙甘草、生姜、桂枝、人参、干地黄、阿胶、麦门冬、麻仁、大枣等药材组成 [2]，配合药量分别为 12 克、9 克、9 克、6 克、30 克、6 克、10 克、10 克、10 枚。炙甘草汤有效治疗血气不足、阳气不旺、心悸、脉虚等病症，临床上常用来治疗冠心病、风湿性心脏病等患者。炙甘草汤有恢复血气、疏通血脉、滋阴补阳等功效。

（2）美托洛尔为西药，白色片状药物，它常用于治疗心绞痛、心律失常、高血压等患者[3]。美托洛尔用量过多可导致低血压、窦性心动过缓等症状，糖尿病、甲亢及孕妇慎用[4]。

（3）炙甘草汤每日给患者一剂，煎服，一日早中晚服用三次；美托洛尔稀释后给患者静脉输液，一天一次，一次2.5mg剂量。

B组单独采用美托洛尔静脉输液治疗。

1.3 疗效判定标准

以患者临床症状消失或减少为疗效判定标准，临床症状包括头晕、心悸、脸色灰白、浑身乏力等，最终结果统计计算A、B两组的治疗显著率、好转率，对比两组疗效。

（1）效果显著：患者无头晕、无心悸现象，脸色恢复正常，无力感消失，能生活自理。

（2）效果好转：患者无头晕、无心悸现象，但仍然脸色苍白、无力。

（3）无效：患者所有临床症状均没有消失。

1.4 统计学方法

本次研究A、B两组数据均采用SPSS 20.0进行分析统计，组间比较采用t检验，计数资料采用x^2检验，以$P<0.05$表示差异有统计学意义。

2.结果

（1）A组经炙甘草汤联合美托洛尔治疗，效果显著23例，显著率82%（23/28）；效果好转5例，好转率18%(5/28）。无无效者。

（2）B组经美托洛尔治疗，效果显著14例，显著率50%（14/28）；效果好转7例，好转率25%（7/28），无效7例，无效率25%（7/28）。

（3）A组治疗效果显著率82%高于B组治疗效果显著率50%。

3.讨论

科技的发展为人们带来了高品质的生活，但个人生活习惯不当、个人健康不注重护理导致了一些疾病，如人们日常饮食习惯以高糖高盐高脂肪为主，就会诱发各种疾病，冠心病作为其一，近年来我国冠心病病发率呈现上升的趋势，病发人群也逐年呈现低龄化[5]，而心律失常是冠心病的主要并发症，患者通常表现为心跳缓慢、心悸、浑身乏力、头痛头晕等[6]，这些症状都严重影响了患者的生活质量，更给患者家属带来负担，研究治疗冠心病心律失常患者的药物刻不容缓，更是医学领域中主要研究的重点问题之一，本次研究证实，炙甘草汤联合美托洛尔有效治疗冠心病心律失常病症，有效改善患者的生活质量，因此，应当推广炙甘草汤联合美托洛尔治疗冠心病心律失常患者。

【参考文献】

[1] 冯育均，向光全．炙甘草汤加味治疗冠心病心律失常的疗效观察[J]．中国医药指南，2015，13(36):217-218.
[2] 王艳霞．炙甘草汤辅助治疗心律失常临床观察[J]．现代养生B，2015，6(5):244-245.
[3] 李文安．稳心颗粒联合美托洛尔治疗冠心病合并心律失常的疗效观察[J]．中国医药科学，2016，6(6):22-23.
[4] 曲艳红．老年冠心病病发心律失常采用胺碘酮联合美托洛尔治疗的临床效果观察[J]．中国实用医药，2016，11(2):146-147.
[5] 李水刚．中西医结合治疗冠心病合并室性早搏[J]．吉林中医药，2016，36(1):46-47.
[6] 王巍巍，刘博雷．稳心颗粒联合美托洛尔治疗老年冠心病心律失常的疗效观察[J]．临床医学，2016，36(1):45-46.

小肠内排列术与传统肠梗阻手术治疗广泛粘连性肠梗阻的疗效

吴林
（四川省巴中市巴中华慈医院　四川巴中　636000）

【摘要】目的：主要探讨在广泛性粘连肠梗阻患者治疗之中应用小场内排列手术和传统梗阻术治疗的效果。方法：选取我院行手术治疗的46例肠梗阻病人，按治疗方式间的差异分成传统组和排列组，传统组采用传统的肠梗阻手术治疗，排列组采用小肠内的排列手术治疗，统计治疗疗效。结果：两组患者并发症的发生率之间有着显著的差别，组间对比有着明显差异，$P<0.05$。结论：在广泛性粘连肠梗阻患者治疗之中应用小场内排列手术，不容易发生并发症，具有应用的价值。

【关键词】小场内排列术；传统肠梗阻术；粘连性肠梗阻
【中图分类号】R615　【文献标识码】A　【文章编号】1007-8231（2016）28-0061-02

粘连性的肠梗阻属于临床上常见的急腹症，在各类肠梗阻患者中占比为25%～40%。过去，临床主要使用传统的肠梗阻术治疗，这种手术可以缓解患者临床症状，将粘连解除，但是术后会引起新粘连与创面，影响了患者康复[1]。所以建议使用小肠内的排列手术治疗，基于此，本文分析了在广泛性粘连肠梗阻患者治疗之中应用小场内排列手术和传统梗阻术治疗的效果，具体总结为。

1.研究资料和治疗方法

1.1 研究的资料

选取2015年10月—2016年10月期间于我院行手术治疗的46例肠梗阻病人，按治疗方式间的差异分成传统组和排列组。所选患者中男性26例，女性20例；患者年龄段在24～67岁之间，平均的年龄为（43±2.04）岁。两组患者基本资料间没有显著的差异，$P<0.05$，可继续下文的比较工作。

1.2 治疗的方法

全部患者在进入手术室以后都实施气管插管的全麻。传统组使用传统的肠梗阻术进行治疗，按照影像学的检查结果对切口进行探查，不可以盲目的对粘连进行分离，在进入到腹腔以后，寻找梗阻的位置，避免破坏到腹膜和肠壁浆的膜层，将粘连松解。广泛性粘连肠梗阻病人需要实施局部切除，钝性分离粘连，小肠分离，这个过程中不可以损伤到肠壁。一些坏死肠管或是伴血运障碍患者，需要实施减压的手术，如果严重肠狭窄或肠坏死无法修补，需要实施肠吻合或是长切除的手术。

排列组患者使用小肠内的排列手术进行治疗：首先游离小肠，一些粘连成团或是血运障碍患者，需要对粘连部位进行松解，于距阑尾根部1厘米盲肠壁的位置，实施荷包的缝合，同时将阑尾切除。应用M-A管在残端开口位置实施肠内的支撑，在阑尾切除者残株开口位置插M-A管，经阑尾的根部逆行插入到小肠，在屈氏韧带距15～25厘米，把小肠每距16～20厘米进行折叠与排列，通过盘状来排列患者肠袢，对阑尾残端进行结扎，同时做荷包的缝合，在腹外对M-A管进行固定。在术后的半个月，等患者戳孔的腹壁和造口间隧道完全形成后，包埋残端，将M-A

万方数据

infection in a general population sample[J].Endoscopy,2013,35(11): 946-950.
[5] 中国中西医结合学会消化系统疾病专业委员会.功能性消化不良的中西医结合诊疗共识意见(2010)[J].中国中西医结合杂志,2011,31(11):1545-1549.
[6] 席淑华.分析慢性浅表性胃炎肝胃不和证患者采用中医柴胡疏肝散治疗的临床效果[J].医学理论与实践,2015,28(16):2176-2178.
[7] 黄振鹏,梁仲惠,刘涛,等.复方台乌片治疗慢性浅表性胃炎疗效观察[J].现代中西医结合杂志,2015,24(1):8-10.
[8] 牟来品.柴胡疏肝散加减治疗肝胃不和型慢性浅表性胃炎 4 例[J].河南中医,2013,33(9):1567-1568.
[9] Annibale B,Di Giulio E,Caruana P,et al.The long-term effects of cure of Helicobacter pylori infection on patients with atrophic body gastritis[J].Alimeng Pharmacol Ther,2002,16(10):1723-1731.
[10] 曹秀梅,朱玉清,徐冰,等.自拟通降颗粒治疗慢性浅表性胃炎 75 例的临床疗效及其机制探讨[J].中医药学报,2013,41(6):65-67.
[11] 卢保强,姜蓉,范良.疏肝和中汤治疗肝胃不和型慢性浅表性胃炎疗效观察[J].山东中医药大学学报,2014(6):559-561.
[12] 李美,孙珍.柴胡疏肝散治疗慢性浅表性胃炎的疗效观察[J].黑龙江中医药,2013,12(2):27-28.
[13] 贾晨光,何昌生.柴胡疏肝散加味治疗慢性浅表性胃炎肝胃不和证 54 例临床观察[J].中医药导报,2014,20(6):124-125.
[14] 韩秀芬.柴胡疏肝散加减治疗 46 例慢性浅表性胃炎肝胃不和证的临床疗效观察[J].中国医药指南,2016,14(19):205-206.

炙甘草汤治疗冠状动脉粥脉硬化性心脏病伴心律失常患者的临床疗效

苏 欣

【摘要】目的 探讨炙甘草汤治疗冠状动脉粥脉硬化性心脏病伴心律失常患者的临床疗效。**方法** 选取 2015 年 6 月至 2016 年 6 月于鞍山市中心医院就诊的 70 例冠状动脉粥脉硬化性心脏病伴心律失常患者作为研究对象，按随机数字表法分为试验组和对照组，各 35 例。所有患者均进行常规治疗，同时对照组患者给予美托洛尔，试验组患者在对照组基础上使用炙甘草汤进行治疗，比较两组患者心律失常改善情况及临床疗效。**结果** 治疗后，试验组患者的室性期前收缩、房性期前收缩、房室交界期前收缩次数均明显少于对照组，差异均有统计学意义（均 $P<0.05$）；试验组患者治疗的总有效率明显高于对照组，差异有统计学意义（$P<0.05$）。**结论** 采用炙甘草汤联合美托洛尔治疗冠状动脉粥脉硬化性心脏病伴心律失常临床疗效显著。

【关键词】炙甘草汤；冠状动脉粥脉硬化性心脏病；心律失常；临床疗效
【DOI】10.12010/j.issn.1673-5846.2017.03.033

冠状动脉粥脉硬化性心脏病是临床常见的一种心血管疾病，其中心律失常是其最常见的一种并发症。该疾病主要是由于心脏自身传导系统和起搏发生障碍所致，为病理性异常搏起[1]。近年来，由于对中医中药的重视，中西医联合治疗逐渐在临床上占据主导地位，主要用于治疗“心悸动、脉结代”的炙甘草汤逐已应用于冠状动脉粥脉硬化性心脏病的治疗中。本研究就炙甘草汤治疗冠状动脉粥脉硬化性心脏病伴心律失常患者的临床疗效进行分析，现报道如下。

1 资料与方法

1.1 一般资料 选取 2015 年 6 月至 2016 年 6 月于鞍山市中心医院就诊的 70 例冠状动脉粥脉硬化性心脏病伴心律失常患者作为研究对象，按随机数字表法分为试验组和对照组，各 35 例。对照组患者中，男 17 例，女 18 例，年龄 45～65 岁，平均（49.2±2.3）岁；房性早搏 13 例，室性早搏 11 例，房室交界性早搏 11 例。试验组患者中，男 18 例，女 17 例，年龄 44～64 岁，平均（48.5±2.4）岁；房性早搏 12 例，室性早搏 12 例，房室交界性早搏 11 例。两

鞍山市中心医院，辽宁鞍山 114001

万方数据

组患者性别、年龄、心律失常类型比较，差异无统计学意义（$P>0.05$），具有可比性。

1.2 **诊断标准** 中医诊断标准参考《中药新药临床研究指导原则》[2]，患者的主要症候为心悸动、胸闷动则悸发、虚数为主，辨证属气阴两虚证。西医诊断标准符合《缺血性心脏病的命名和诊断标准》[3]，患者有非阵发性室性心动过速或者室性期前收缩。

1.3 **纳入及标准** 符合诊断标准；所有患者均符合鞍山市中心医院医学伦理委员会相关要求，均签署了知情同意书。

1.4 **排除标准** 重度或急性心力衰竭、低血压、心动过缓、心源性休克；严重肝肾功功能；肿瘤患者。

1.5 **治疗方法** 所有患者均进行常规治疗，主要包括应用硝酸酯类、钙离子拮抗剂等药物。在此基础上对照组患者使用美托洛尔（阿斯利康制药有限公司，批号：1511029）进行治疗，25 mg/次，2 次/d，需根据患者心率调整剂量。试验组患者在对照组基础上使用炙甘草汤进行治疗，组方：炙甘草 12 g、麦冬 10 g、人参 6 g、阿胶 6 g、桂枝 9 g、生地黄 30 g、麻子仁 10 g、生姜 9 g 以及大枣 5 枚。其中伴下肢水肿患者加茯苓 10 g、泽泻 10 g；阴虚盗汗患者加枸杞子 10 g、麻黄根 10 g。煎煮至 100 ml，分早、晚两次服用。两组患者均治疗 10 d。

1.6 **观察指标** 比较两组患者心律失常改善情况及临床疗效。

1.7 **疗效判定标准** 显效：患者心悸、气短以及乏力等临床症状完全消失，发作次数减少超过 90%；有效：患者心悸、气短以及乏力等症状明显缓解，发作次数减少 50%～90%；无效：患者心悸、气短以及乏力等症状无改善甚至加重，发作次数减少不超过 50%[4]。总有效率（%）=（显效例数+有效例数）/总例数×100%。

1.8 **统计学分析** 采用 SPSS 17.0 统计软件进行数据分析，计量资料以$\bar{x}\pm s$ 表示，组间比较采用 t 检验，计数资料以百分率表示，组间比较采用 χ^2 检验，$P<0.05$ 为差异有统计学意义。

2 结果

两组患者治疗前室性期前收缩、房性期前收缩、房室交界期前收缩次数差异均无统计学意义（$P>0.05$）；治疗后，试验组室性期前收缩、房性期前收缩、房室交界期前收缩次数均明显少于对照组，差异均有统计学意义（均 $P<0.05$）；试验组患者治疗的总有效率明显高于对照组，差异有统计学意义（$P<0.05$）。见表 1。

3 结论

近年来，随着生活节奏加快，饮食结构改变，冠状动脉粥样硬化性心脏病的发病率逐年升高，年龄趋于低龄化。冠状动脉粥脉硬化性心脏病伴心律失常可直接影响患者生命质量，很多患者会出现心悸、乏力等症状，严重的甚至会威胁生命。因此，积极采取有效措施的治疗冠状动脉粥样硬化性心脏病导致的心律失常非常重要。

临床上对冠状动脉粥脉硬化性心脏病伴心律失常的治疗主要以扩张血管、改善心肌缺血、减慢心率和改善心功能为主，因此临床上多用 β-受体体阻滞剂治疗该病。美托洛尔临床使用最为广泛，是Ⅱ类抗心律失常药物，其主要作用为减慢心率、提高心肌细胞域值、降低兴奋传导的速度；其可以兴奋交感神经，增加钙、钠离子内流，促进钾离子外流，从而增加与膜受体的竞争性结合[5]；还可以兴奋迷走神经，容易通过血-脑屏障，降低交感神经兴奋性[6]。美托洛尔还具有抗血小板凝聚和抗高血压作用，对于由于高血压引起的心律失常也有较好临床疗效[7]，但糖尿病、支气管哮喘、严重心功能不全患者慎用。并且其对患者的临床症状改善并不明显，很多患者虽然坚持用药但仍经常出现心悸、气短以及乏力等现象[8]。因此，临床上经常使用中西医结合方法治疗冠状动脉粥脉硬化性心脏病伴心律失常。

炙甘草汤来源于《伤寒论》，主要具有益气复脉、滋阴补血的作用，临床主要用于治疗心律失常性心脏病。方中炙甘草具有补中益气作用，人参具有大补元气、安五脏的作用，大枣益养心脾，三药联用，可以起到滋补后天之本的作用，使气血生化有源[9]。

表 1 两组患者心律失常改善情况及临床疗效比较

组别	例数	室性期前收缩(次/24 h,$\bar{x}\pm s$)		房性期前收缩(次/24 h,$\bar{x}\pm s$)		房室交界期前收缩(次/24 h,$\bar{x}\pm s$)		临床疗效			
		治疗前	治疗后	治疗前	治疗后	治疗前	治疗后	显效(例)	有效(例)	无效(例)	总有效[例(%)]
对照组	35	1870±164	987±104	1011±92	523±43	267±35	167±18	15	15	5	30(85.7)
试验组	35	1869±154	653±62	1009±87	297±31	258±23	103±9	18	15	2	33(94.3)
t/χ^2 值		2.35	3.12	2.68	3.14	2.89	3.47				3.47
P 值		>0.05	<0.05	>0.05	<0.05	>0.05	<0.05				<0.05

万方数据

生地黄可以滋阴养血，麦冬清心、润肺、益气，阿胶滋阴、补血，三药联用起到滋养阴精、使脉道滑利的作用。桂枝温阳复脉、温通阳气，生姜温里散寒，诸药合用起到滋阴益气、通阳复脉的效果。现代药理学研究显示，炙甘草汤可以起到正性肌力作用，可以对抗早搏，增加心肌细胞内线粒体抗氧化能力，从而增加冠状动脉血流量。另外有研究显示，该方还可以对抗心肌缺血、缺氧，使心脏快速恢复节律，从而起到抗心律失常作用[10]。

通过应用美托洛尔降低迷走神经兴奋，减慢心律，抗心律失常，再联合应用炙甘草汤，从中医角度调和患者气血，使其血脉充盛，从而达到缓解临床症状的目的。本研究结果显示，治疗后试验组室性期前收缩、房性期前收缩、房室交界期前收缩次数均明显少于对照组，治疗的总有效率明显高于对照组。说明，使用炙甘草汤联合美托洛尔治疗冠状动脉粥脉硬化性心脏病伴心律失常，可以明显提高临床疗效，降低心律失常发生，提高患者生命质量。

参考文献

[1] 王哲.炙甘草汤联合美托洛尔片治疗冠状动脉粥样硬化性心脏病心律失常[J].中国中医药现代远程教育,2016,14(9):91-92.
[2] 谭瑜.心悸患者应用炙甘草汤加味治疗的临床效果观察[J].中国临床研究,2014,6(14):119-120.
[3] 梁朝矿,何树发,李文升,等.急诊应用胺碘酮对冠状动脉粥样硬化性心脏病快速心律失常的疗效研究[J].中国医学创新,2015,12(19):45-46.
[4] 艾都凤.炙甘草汤联合美托洛尔治疗冠状动脉粥样硬化性心脏病心律失常[J].实用临床医学,2015,16(2):34-35.
[5] 陈思法.炙甘草汤合血府逐疲汤治疗冠心病室性早搏疗效观察[J].河南中医,2012,32(6):765-766.
[6] 杨传民.美托洛尔联合步长稳心颗粒治疗心率失常疗效观察[J].中国药物经济学,2012,7(6):156-157.
[7] 王纪岗.炙甘草汤联合美托洛尔治疗冠状动脉粥样硬化性心脏病心律失常 45 例[J].河南中医,2014,34(3):418-419.
[8] 龚茨平,吴海龙,谢红光,等.美托洛尔胺碘酮与门冬氨酸钾镁治疗 50 例心肌梗死合并心率失常观察[J].上海医药,2016,37(1):28-30.
[9] 马媛,朱创洲.炙甘草汤加减治疗冠心病合并心律失常 60 例[J].现代中医药,2011,31(2):4-5.
[10] 张红霞.炙甘草汤治疗冠心病心律失常临床观察[J].光明中医,2011,26(4):743-744.

益元清肝健运汤治疗 2 型糖尿病合并高脂血症患者的临床效果

苏玉洁

【摘要】目的 探讨益元清肝健运汤治疗 2 型糖尿病合并高脂血症患者的临床效果。**方法** 选取 2015 年 4 月至 2016 年 4 月于旅顺口区中医医院接受治疗的 105 例 2 型糖尿病合并高脂血症患者为研究对象，采用随机数字表法将患者分为观察组（53 例）和对照组（52 例）。对照组患者给予西医综合方法治疗，观察组患者给予益元清肝健运汤治疗，比较两组患者治疗前后各项血糖、血脂指标变化情况。**结果** 治疗后，观察组患者餐后 2 h 血糖（2hPBG）、空腹血糖（FBG）及糖化血红蛋白（HbAlc）水平均明显低于对照组，差异均有统计学意义（均 $P<0.05$）；治疗后，观察组患者血清总胆固醇（TC）、三酯甘油（TG）、低密度脂蛋白胆固醇（LDL-C）水平均明显低于对照组，高密度脂蛋白胆固醇（HDL-C）水平明显高于对照组，差异均有统计学意义（均 $P<0.05$）。**结论** 益元清肝健运汤治疗 2 型糖尿病合并高脂血症能够有效改善患者血脂及血糖水平，提升治疗总有效率，效果显著。

【关键词】益元清肝健运汤；2 型糖尿病；高脂血症；临床效果
【DOI】10.12010/j.issn.1673-5846.2017.03.034

大连市旅顺口区中医医院，辽宁大连 116041

万方数据

炙甘草汤、美托洛尔联合治疗心律失常分析

孙俊雄

【摘要】目的 分析加减炙甘草汤联合美托洛尔治疗冠心病室性心律失常的疗效。方法 选择我院 2015 年 1 月—2018 年 1 月收治的冠心病室性心律失常患者 80 例作为研究对象。将其随机分为两组，每组各 40 例。对照组采用西药疗法—美托洛尔，观察组在对照组治疗方法的前提下，给予患者服用炙甘草汤。对两组患者治疗前和治疗后的生命体征、血常规、心电图等指标进行观察和记录，并且对比两组患者治疗前和治疗后的观察指标值。结果（1）观察组患者的治疗总有效率为 95%，对照组为 75%，观察组治疗总有效率高于对照组，差异具有统计学意义（χ^2=6.27，P=0.012 ＜ 0.05）。（2）两组患者治疗前后谷丙转氨酶、尿素氮、肌酐水平比较，差异无统计学意义（P ＞ 0.05）。结论 加减炙甘草汤联合美托洛尔治疗冠心病室性心律失常的疗效明显，可有效地改善患者的心功能，并且不会对肝肾功能造成影响，安全可靠。

【关键词】加减炙甘草汤；美托洛尔；冠心病；室性心律失常；疗效；中西结合
【中图分类号】R259　　【文献标识码】A
【文章编号】1674-9308（2019）09-0140-03
doi：10.3969/j.issn.1674-9308.2019.09.063

Analysis of Combination of Zhigancao Decoction and Metoprolol in the Treatment of Arrhythmia

SUN Junxiong Department of Integrated Chinese and Western Medicine, Concord Jingshan Hospital of Huazhong University of Science and Technology, Jingshan Hubei 431800, China

[Abstract] Objective To analyze the effect of jiajian zhigancao decoction combined with metoprolol on ventricular arrhythmia of coronary heart disease. **Methods** 80 cases of patients with ventricular arrhythmia of coronary heart disease admitted to our hospital from January 2015 to January 2018 were selected as the study subjects. They were randomly divided into two groups, 40 cases in each group. The control group was treated with metoprolol. The observation group was given zhilicorice decoction on the premise of the treatment method of the control group. The vital signs, blood routine and electrocardiogram of the two groups were observed and recorded before and after treatment, and to compare the two groups of patients before and after treatment of the observed indicators. **Results** (1) The total effective rate was 95% in the observation group and 75% in the control group. The total effective rate in the observation group was higher than that in the control group. The difference was statistically significant (χ^2=6.27, P=0.012<0.05). (2) There was no significant difference in the levels of alanine aminotransferase, urea nitrogen and creatinine between the two groups before and after treatment (P > 0.05). **Conclusion** Jiajian zhigancao decoction combined with metoprolol in the treatment of ventricular arrhythmia of coronary heart disease has obvious curative effect, can effectively improve the heart function of patients, and will not affect the liver and kidney function, safe and reliable.

[Keywords] jiajian zhigancao decoction; metoprolol; coronary heart disease; ventricular arrhythmia; curative effect; combination of Chinese and western medicine

作者单位：华中科技大学协和京山医院中西医结合内科，湖北 京山 431800

目前，常用的心律失常调整药物较多，但是长期使用容易导致病死率的增加。现在应用较为稳定的药物包括美托洛尔类、胺碘酮类，存在较多的不良反应。在中医理论，心律失常归属于中医的“心悸”“胸痹”“心痛”的范畴[1]。其致病因素主要在于心血不足，缺乏血液滋养心脏，同时合并心虚胆怯，肝肾阴虚，痰饮内停，血脉堵塞，本虚标实，导致气血不足更加严重，血脉运行也因此更加不畅[2]。本研究通过对我院冠心病收治的室性心律失常患者进行对照试验，探讨加减炙甘草汤联合美托洛尔治疗冠心病室性心律失常的疗效，并将其与常规治疗进行对比。现报告如下。

1 资料与方法

1.1 基本资料

选择我院 2015 年 1 月—2018 年 1 月收治的冠心病室性心律失常患者 80 例作为研究对象。将其随机分为两组，每组各 40 例。观察组中，男性患者 23 例，女患者 17 例，年龄 49 ～ 69 岁，平均年龄为（56.0 ± 3.5）岁；对照组中，男性患者 22 例，女性患者 18 例，年龄 49 ～ 69 岁，平均年龄为（56.1 ± 2.8）岁。两组患者的一般资料对比，差异无统计学意义（P ＞ 0.05），具有可比性。所有患者均知晓本次研究，并签署知情同意书；本研究经医院伦理委员会批准，未有基金项目支持。纳入标准：均符合冠心病室性心律失常的诊断标准。排除标准：排除严重脑肝肾疾病患者，排除其他严重原发疾病患者，排除严重外伤史患者，排除中途中断研究的患者，排除精神障碍、言语表达不清、意识模糊的患者。

1.2 方法

对照组：采用西药疗法，用药方案：美托洛尔（批准文号国药准字 H32025391，阿斯利康制药有限公司，25 mg × 20 片），起初 6.25 mg/ 次，一天 2 ～ 3 次，以后根据症状情况按照 6.25 ～ 12.5mg/ 次的幅度调整使用剂量，一天 2 次，最大剂量不应超过 300 ～ 400 mg/ 天。持续治疗 1 个疗程（1 个月为 1 个疗程）。

观察组：在对照组治疗方法的前提，给予患者服用炙甘草汤，去生姜、桂枝、阿胶、麻仁、大枣，加丹参、当归、苦参、川弓、香附、桑寄生，水煎，分 2 次服用，疗程 1 个月。根据患者具体病症进行加减：伴随心悸不宁、汗多的患者加龙骨 10 g、牡蛎 10 g，可安神。

1.3 观察指标

对患者治疗前和治疗后的生命体征、血常规、心电图等指标进行观察和记录，并且对比两组患者治疗前和治疗后的观察指标值（谷丙转氨酶、尿素氮、肌酐）。

1.4 疗效判断标准

以《中药新药临床研究指导原则》作为疗效判定标准[3]。一共包括3个级别：显效：胸痛、胸闷等症状消失或者得到明显的改善，心律失常发作次数减少至少90%，心电图检查显示正常；有效：胸痛、胸闷等症状有所改善，心律失常发作次数减少达到50% ~ 90%，心电图检查显示正常；无效：胸痛、胸闷等症状无改善，或者加重，心律失常发作次数减少不足50%，心电图显示无明显的变化。

1.5 统计学方法

采用SPSS 20.0软件对数据进行分析处理，计量资料以（均数 ± 标准差）表示，采用 t 检验；计数资料以（n，%）表示，采用 χ^2 检验，以 $P < 0.05$ 表示差异具有统计学意义。

2 结果

2.1 临床疗效

观察组患者的治疗总有效率为95%，对照组为75%，观察组治疗总有效率高于对照组，差异具有统计学意义（$P < 0.05$），见表1。

2.2 肾功能指标

两组患者治疗前后谷丙转氨酶、尿素氮、肌酐水平比较，差异无统计学意义（$P > 0.05$），见表2。

3 讨论与结论

心律失常，因为患者窦房结激动异常，或者由于激动产生于窦房结外，导致激动的传导缓慢、阻滞，或者引起激动经异常通道传导，也就是心脏活动的起源、传导障碍导致心脏搏动的频率、节律异常[4-5]。心律失常是常见的一种心血管疾病。既可单独发病亦可与心血管病伴发。许多患者容易突然发作而致猝死，可持续累及心脏而衰竭。心律失常容易引起冠状动脉血流量降低，偶发房性期前收缩可导致冠状动脉血流量减低，频发性的室性期前收缩可降低，房性心动过速时冠状动脉血流量降低，甚至于心室颤动时冠状动脉血流量可能为0[6-9]。

在中医理论，心律失常归属于中医的“心悸”“胸痹”“心痛”的范畴。根据中医名著《伤寒论》记载，脉结代，心动悸，认为益气养阴、活血化瘀为主要的治疗方向和方法。炙甘草汤去生姜、桂枝、阿胶、麻仁、大枣，加丹参、当归、苦参、川弓、香附、桑寄生，可达到滋阴养血、益气活血化瘀的功效[10]。根据现代药理学研究，甘草、人参、生地黄、麦冬、丹参、当归、苦参、川弓等药材均可抗心律失常，效果明显，并且可抗缺氧、抗心肌缺血，可扩张冠状动脉血管，从而改善血液流动循环，提高心肌耐缺氧能力，促进微循环得以改善，抗凝效果较为明显，可抑制血栓的形成。方中重用炙甘草甘温益气，通经脉，利血气，缓急养心为君[11]。人参、大枣益气补脾养心，生地、麦冬、麻仁、阿胶，滋阴养血为臣；桂枝，生姜、清酒温阳通脉为佐。诸药合用，温而不燥，滋而不腻，共奏益气养血，滋阴复脉之功[12]。本研究显示，观察组临床有效率为95%，对照组临床有效率为75%，观察组临床有效率高于对照组，差异具有统计学意义（$P < 0.05$）。两组患者治疗前后谷丙转氨酶、尿素氮、肌酐水平比较，差异无统计学意义（$P > 0.05$）。由此可见，加减炙甘草汤联合美托洛尔治疗冠心病室性心律失常的疗效明显，可有效地改善患者的心功能，并且不会对肝肾功能造成影响，安全可靠。和有关研究结果相符合。

综上所述，加减炙甘草汤联合美托洛尔治疗冠心病室性心律失常的疗效明显，可有效地改善患者的心功能，并且不会对肝肾功能造成影响，安全可靠。

表1 比较两组患者的临床疗效 n（%）

组别	例数	显效（例）	有效（例）	无效（例）	临床总有效[例（%）]
观察组	40	25	13	2	38（95）
对照组	40	20	10	15	30（75）
χ^2 值	–	–	–	–	6.27
P 值	–	–	–	–	0.012

数据错误，应该是10

表2 比较两组患者治疗前后的肝肾功能指标（$\bar{x} \pm s$）

组别	例数	谷丙转氨酶（U/L）	尿素氮（mmol/L）	肌酐（mmol/L）
观察组	40			
治疗前		33.49 ± 4.89	7.32 ± 0.90	111.90 ± 12.89
治疗后		34.19 ± 5.10	7.19 ± 0.99	112.10 ± 13.90
t 值		0.63	0.61	0.07
P 值		> 0.05	> 0.05	> 0.05
对照组	40			
治疗前		33.21 ± 4.79	7.15 ± 1.09	112.99 ± 15.00
治疗后		33.72 ± 5.49	7.35 ± 1.19	112.09 ± 13.88
t 值		0.44	0.78	0.28
P 值		> 0.05	> 0.05	> 0.05
$t_{(治疗前组间)}$ 值		0.40	0.65	0.00
$P_{(治疗前组间)}$ 值		0.69	0.52	0.10
$t_{(治疗后组间)}$ 值		0.397	0.654	0.003
$P_{(治疗后组间)}$ 值		0.692	0.515	0.998

参考文献

[1] 张春艳 . 炙甘草汤联合美托洛尔治疗老年冠心病心律失常的临床研究 [J]. 内蒙古中医药，2018，37（6）：56-57.

[2] 董江涛 . 加减炙甘草汤治疗冠心病伴发房早及室早疗效观察 [J]. 实用中医药杂志，2018，34（5）：516-517.

[3] 周贝，刘亚琳，唐健元 . 我国中药新药临床研究技术指导原则体系发布概况 [J]. 中国临床药理学杂志，2017，33（18）：1850-1852.

[4] 王珊珊，王元鑫 . 加减炙甘草汤联合美托洛尔治疗冠心病室性心律失常的疗效观察 [J]. 中外女性健康研究，2018，26（4）：54，57.

[5] 夏莉 . 炙甘草汤加减联合丹参滴丸治疗冠心病室性早搏的临床疗效 [J]. 内蒙古中医药，2016，35（17）：38.

[6] 赵秉科 . 分析冠心病心律失常患者采取炙甘草汤加减治疗的疗效 [J]. 中西医结合心血管病电子杂志，2016，4（33）：162-163.

[7] 孙国香 . 炙甘草汤联合美托洛尔缓释片治疗冠心病心律失常 40 例 [J]. 浙江中医杂志，2016，51（2）：138.

[8] 邰小燕 . 炙甘草汤加减联合西药治疗冠心病心律失常的临床分析 [J]. 中西医结合心血管病电子杂志，2014，2（12）：39-40.

[9] 滕祖训 . 炙甘草汤加减治疗冠心病心律失常的临床价值分析 [J]. 中国疗养医学，2014，23（4）：330-331.

[10] 王纪岗 . 炙甘草汤联合美托洛尔治疗冠状动脉粥样硬化性心脏病心律失常 45 例 [J]. 河南中医，2014，34（3）：418-419.

[11] 苗灵娟，杨永枝，邢海燕，等 . 炙甘草汤联合胺碘酮治疗气阴两虚冠心病室性心律失常疗效观察 [J]. 陕西中医，2017，38（2）：169-170.

[12] 刘洋，王利亚，郑相慧 . 门冬氨酸钾镁联合胺碘酮治疗冠心病室性心律失常的疗效以及对心电图 PR 间期和 QRS 时限的影响 [J]. 中国老年学杂志，2012，32（23）：5307-5308.

麝香保心丸配合厄贝沙坦治疗急性心肌梗死

周蓉

【摘要】目的 分析与探讨麝香保心丸配合厄贝沙坦治疗急性心肌梗死的有效性。方法 选取我院 2015 年 6 月—2018 年 6 月收治的 72 例急性心肌梗死患者作为研究对象，并将其分为实验组与对照组，每组各 36 例患者。其中，对照组采用常规的西药治疗方式，实验组则在对照组的基础上，予以麝香保心丸配合厄贝沙坦进行治疗。结果 实验组患者中，显效为 31 例，有效为 5 例，总有效率为 100%；对照组患者中，显效为 22 例，有效为 8 例，无效为 6 例，总有效率为 83.3%。实验组的总有效率优于对照组，差异具有统计学意义（$P < 0.05$）。结论 在临床治疗急性心肌梗死中，采用麝香保心丸配合厄贝沙坦的治疗方式，能够产生理想的治疗效果，提升患者的生活质量。

【关键词】麝香保心丸；厄贝沙坦；急性心肌梗死；疗效观察；联合治疗；心血管内科

【中图分类号】R542 【文献标识码】A

【文章编号】1674-9308（2019）09-0142-03

doi：10.3969/j.issn.1674-9308.2019.09.064

Shexiang Baoxin Pill and Irbesartan in the Treatment of Acute Myocardial Infarction

ZHOU Rong Department of Cardiology, The First People's Hospital of Jingzhou City, Jingzhou Hubei 434000, China

[Abstract] Objective To analyze and discuss the effectiveness of shexiang baoxin pill combined with irbesartan in the treatment of acute myocardial infarction. **Methods** 72 patients with acute myocardial infarction admitted to our hospital from June 2015 to June 2018 were selected as the study subjects. They were divided into experimental group and control group, 36 patients in each group. The control group was treated with conventional Western medicine. The experimental group was treated with Musk Baoxin Pill and Irbesartan on the basis of the control group. **Results** In the experimental group, 31 cases were markedly effective, 5 cases were effective, and the total effective rate was 100%. In the control group, 22 cases were markedly effective, 8 cases were effective and 6 cases were ineffective. The total effective rate was 83.3%. The total effective rate of the experimental group was better than that of the control group, with statistical significance ($P < 0.05$). **Conclusion** In the clinical treatment of acute myocardial infarction, the treatment method of shexiang baoxin pill combined with irbesartan can produce ideal therapeutic effect and improve the quality of life of patients.

[Keywords] shexiang baoxin pill; irbesartan; acute myocardial infarction; efficacy observation; combination therapy; cardiovascular medicine

在心血管内科，急性心肌梗死是一种非常常见的疾病，其主要发病原因是冠动脉持续性、急性缺氧与缺血而导致的心肌坏死，在临床上主要表现为持久、剧烈的胸骨后疼痛，且患者服用了硝酸酯类药物与休息后也无法得到缓解，还可能出现一系列并发症，如心力衰竭、心律失常、休克等症状，往往会对患者的生命安全造成一定的影响 [1]。根据相关研究表明，在欧美，急性心肌梗死的发病率最为常见，同时，这一疾病在中国的发病率近年来呈上升趋势，进而引起了各界人士的关注 [2]。因此，本文为分析与探讨麝香保心丸配合厄贝沙坦治疗急性心肌梗死的有效性，选取了我院 2015 年 6 月—2018 年 6 月收治的 72 例急性心肌梗死患者作为研究对象，将其分为实验组与对照组两组，每组各 36 例患者。现将具体情况汇报如下。

1 资料与方法

1.1 临床资料

选取我院 2015 年 6 月—2018 年 6 月收治的 72 例急性心肌梗死患者作为研究对象，所有患者都与急性心肌梗死中西医诊断标准相符合，并排除有合并严重的心肝肾与造血系统等疾病的患者、有精神障碍与精神病史无法配个治疗的患者、妊娠期或者处于哺

作者单位：荆州市第一人民医院心内科，湖北 荆州 434000

中医药应用研究

加味炙甘草汤联合美托洛尔治疗冠心病合并心律失常的疗效分析

唐婉斯,谢雪姣

湖南中医药大学,湖南长沙 410208

摘要 目的 观察加味炙甘草汤联合美托洛尔治疗冠心病合并心律失常疗效及安全性。方法 选取 2017 年 9 月—2019 年 4 月湖南中医药大学第二附属医院收治的 60 例冠心病合并心律失常患者为研究对象,随机分为两组。研究组用加味炙甘草汤联合美托洛尔治疗,对照组单用美托洛尔治疗,比较两组心律失常的改善情况、治疗总有效率、治疗前后的中医证候积分及药物不良反应发生率。结果 研究组室性期前收缩(616±40)次/24 h(t=38.069)、房性期前收缩(279±11)次/24 h(t=36.161)、房室交界期前收缩(108±5)次/24 h(t=36.656)等心律失常的改善情况均优于对照组[室性期前收缩(852±20)次/24 h(t=33.540)、房性期前收缩(542±12)次/24 h(t=25.275)、房室交界期前收缩(172±69)次/24 h(t=7.081);研究组治疗总有效率(96.7%)显著高于对照组(46.7%)(χ^2=18.468);研究组的中医证候积分主证(2.23±0.43)分(t=22.564)、次证(3.03±0.77)分(t=42.369),明显低于对照组主证(5.63±0.89)分(t=5.371)、次证(6.12±0.99)分(t=37.764),以上差异有统计学意义(P<0.05)。两组不良反应率(6.6% vs 10.0%)相比差异无统计学意义(χ^2=0.139,P>0.05)。结论 加味炙甘草汤联合美托洛尔治疗冠心病合并心律失常可显著改善症状。

关键词 冠心病;心律失常;美托洛尔;炙甘草汤

中图分类号 R256.2 **文献标志码** A doi10.11966/j.issn.2095-994X. 2019.05.11.48

Curative Effect of Jiawei Zhigancao Decoction Combined with Metoprolol Tablets in the Treatment of Coronary Atheroscierotic Heart Disease Arrhythmia

TANG Wan-si, XIE Xue-jiao

Hunan University Of Chinese Medicine,Changsha, Hunan Province, 410208 China

Abstract Objective To observe the efficacy and safety of JiaweiZhigancao Decoction combined with metoprolol in the treatment of coronary heart disease with arrhythmia. Methods Sixty patients with coronary heart disease and arrhythmia admitted to the Second Affiliated Hospital of Hunan University of Traditional Chinese Medicine from September 2017 to April 2019 were randomLy divided into two groups. The study group was treated with Jiawei Zhigancao Decoction combined with metoprolol. The control group was treated with metoprolol alone. The improvement of arrhythmia, the total effective rate of treatment, the TCM syndrome scores before and after treatment, and the adverse drug reactions occurred rate. Results The study group had ventricular premature contraction (616±40 times/24 h(t=38.069), atrial premature contraction (279±11)times/24 h(t=36.161), and atrioventricular junction contraction (108±5 times/ 24 h(t=36.656) The improvement of arrhythmia was better than that of the control group[(85±20)times/24 h before ventricular contraction (t=33.540),(542±12)times/24 h before atrial contraction (t=25.275), The contraction of the atrioventricular junction was (172±69)times/24 h (t=7.081)]; the total effective rate of the study group (96.7%) was significantly higher than that of the control group (46.7%),(χ^2=18.468); the TCM syndrome score of the study group[The main card was (2.23±0.43)points,(t=22.564)points, sub-test (3.03±0.77)points(t=42.369)], which was significantly lower than the control group [main certificate (5.63±0.89)points(t=5.371), sub-certification (6.12±0.99)points(t=37.764)]. All were statistically significant (P<0.05). There was no significant difference in the adverse reaction rate (6.6% vs 10.0%) between the two groups,the difference was not statistically significant(χ^2=0.139, P>0.05). Conclusion Jiawei Zhigancao Decoction combined with metoprolol can significantly improve the symptoms of coronary heart disease with arrhythmia.

Key words Coronary heart disease; Arrhythmia;metoprolol; Zhigancao decoction

收稿日期:2019-09-24; 修回日期:2019-10-20

作者简介:唐婉斯(1993-),女,湖南湘潭人,硕士在读,住院医师,主要从事中医内科心血管方面的研究工作。

通讯作者:谢雪姣(1976-),女,湖南株洲人,博士后,副主任医师,主要从事中医内科心血管方面的研究工作,E-mail:99511298@qq.com。

心律失常是冠心病最常见的一种并发症，该病发作突然、症状严重，严重危害患者的身心健康及生活质量。它是心脏电位发生变化的频率或节律异常而导致的心脏抽搐、胸闷及血压减少，严重时会出现昏厥或死亡[1]。单纯运用西药治疗心律失常，短时间确有疗效，但常伴有诸多不良反应，且需要加大用药剂量。该次研究采用加味炙甘草汤联合美托洛尔方法对湖南中医药大学第二附属医院2017年9月—2019年4月收治的60例冠心病合并心律失常患者进行治疗，得到了显著疗效且安全性极高，现报道如下。

1 资料与方法

1.1 一般资料

选取湖南中医药大学第二附属医院收治的60例冠心病合并心律失常患者作为研究对象，随机分为两组，各30例。研究组中男18例，女12例；年龄38~75岁，平均年龄(58.83±5.18)；病程6个月~12年，平均病程(5.96±1.23)年；心律失常类型：室性期前收缩12例，房性期前收缩10例，房室交界期前收缩8例。对照组中男16例，女14例；年龄39~76岁，平均年龄(60.58±4.56)岁，病程5个月~11年，平均病程(5.85±1.34)年；心律失常类型：室性期前收缩11例，房性期前收缩10例，房室交界期前收缩9例。两组人员基础资料如性别、年龄、病程、心律失常类型等比较差异无统计学意义(P>0.05)，所有患者及家属均在知情的情况下签署知情同意书，且该研究通过医院伦理委员会批准。

1.2 诊断标准

实验采用的中西医诊断标准均符合《缺血性心脏病的命名和诊断标准》[2]和《中药新药临床研究指导原则》[3]的相关规定；所有患者均有非阵发性室性心动过速或室性期前收缩相关症状。

1.3 纳入及排除标准

纳入标准：根据两组人员的历年病史、体征、冠脉造影、心脏超声以及心电图结果等资料显示，所有患者确诊为冠心病合并心律失常，经评估后均有用药治疗指征，患者及家属均自愿配合完成治疗和研究。

排除标准：肝肾功能严重受损、高血糖、高血压及癌症患者；自身免疫性疾病患者；严重室(上)性心动过速(缓)和心房扑动的患者；其他病情导致的心律失常者；近1周内服用过其它抗心律失常药物者。

1.4 治疗方法

所有入选患者入院后均予以抗血小板聚集、扩张冠状动脉等常规的冠心病治疗。

对照组：基础治疗外给予琥珀酸美托洛尔缓释片(国药准字J20100098；47.5 mg)口服，23.75 mg/次，1次/d，10 d为1个疗程。

研究组：基础治疗结合加味炙甘草汤联合美托洛尔治疗。美托洛尔服用方法同对照组；加味炙甘草汤成分：炙甘草60 g、生地黄50 g、麦冬15 g、火麻仁10 g、桂枝10 g、生姜3片、阿胶(烊化)6 g、党参15 g、大枣3枚，上述煎药水200 mL，早晚各服一次，1剂/d。10 d为1个疗程。

1.5 观察指标及疗效标准

(1)通过心电图(Holter或ECG)观察患者用药前后心律失常改善情况。(2)对比两组患者治疗有效率[4-5]：①显效：患者经心电图检测基本恢复正常，且病人心悸、气短以及乏力等症状完全消失，发作次数减少小于10%；②有效：患者以上症状明显改善，减少的发作次数大于50%，小于90%，心电图检测结果显著改善；③无效：患者病情以及心电图检查结果没有改善甚至严重，发作次数减少小于50%。治疗有效率=(显效例数+有效例数)/总例数×100.0%。(3)两组治疗前后的中医证候积分：主证心悸总分9分，无症状为0分、偶尔发生(≤3次)为3分、偶尔发生(3~5次)为6分、经常发生(>5次)且不适感加重为9分；次证总分15分，无症状为0分、轻度为1分、中度为2分、重度为3分。(4)两组药物不良反应比较：恶心呕吐、头晕、心率减慢等。

1.6 统计方法

数据应用SPSS 20.0统计学软件进行分析，计数资料(%)进行χ^2检验，计量资料($\bar{x}\pm s$)进行t检验，P<0.05为差异有统计学意义。

2 结果

2.1 两组患者室性、房性及房室交界期前收缩的改善情况

治疗前两组患者室性期前收缩、房性期前收缩、房室交界期前收缩等各指标对比，差异无统计学意义P>0.05)；治疗后，与对照组相比，研究组各指标显著降低，差异有统计学意义(P<0.05)，见表1。

表1 两组心律失常改善情况对比[($\bar{x}\pm s$)，次/24 h]

组别	时间	室性期前收缩	房性期前收缩	房室交界期前收缩
研究组	治疗前	1 775±166	1 079±121	258±22
	治疗后	(616±40)*#	(279±11)*#	(108±5)*#
	t值*	38.069	36.161	36.656
	P值*	0.000	0.000	0.000
对照组	治疗前	1 777±158	1 076±120	256±18
	治疗后	(852±20)*	(542±12)*	(172±69)*
	t值*	33.540	25.275	7.081
	P值*	0.000	0.000	0.000
	t值#	-30.915	-97.114	-5.011
	P值#	0.000	0.000	0.000

注：*与治疗前对比P<0.05，#与对照组对比P<0.05

2.2 两组有效率比较

治疗后，研究组患者中显效16例，有效13例，无效1例，总治疗有效率为96.7%，对照组患者中显效9例，有效

5 例,无效 16 例,总治疗有效率为 46.7%,研究组总治疗有效率显著高于对照组,差异有统计学意义(χ^2=18.468,P=0.000<0.05)。

2.3 两组中医证候积分对比

治疗前,两组中医证候积分相当(P>0.05);治疗后,研究组的中医证候积分(主证与次证)均明显低于对照组,差异有统计学意义(P<0.05)。见表 2。

表 2 两组中医证候积分比较[($\bar{x}\pm s$),分]

组别	时间	主证	次证
研究组	治疗前	7.30±1.15	12.03±0.89
	治疗后	(2.23±0.43)*#	(3.03±0.77)*#
	t 值*	22.564	42.369
	P 值*	0.000	0.000
对照组	治疗前	7.72±1.50	12.58±1.53
	治疗后	(5.04±0.82)*	(6.12±0.99)*
	t 值*	5.371	37.764
	P 值*	0.000	0.000
	t 值#	−20.821	−15.216
	P 值#	0.000	0.000

注:* 与治疗前对比 P<0.05,# 与对照组对比 P<0.05

2.4 不良反应比较

研究组和对照组均有 1 例恶心呕吐,分别有 1 例和 2 例缓慢型心律失常,不良反应率分别为 6.6%和 10.0%;两组不良反应率比较,差异无统计学意义(χ^2=0.139,P=0.709>0.05)。

3 讨论

中医没有冠心病合并心律失常病名,但是从该病的症状观察,可将该病归属于中医的“胸痹心悸”“心动悸”“怔忡”等范畴[6]。冠心病心律失常发生的主要原因是饮食不当、年老体虚和情志失调以及外邪入体导致患者心神不宁、心脉气血运行不畅,引起患者心悸、可能会引起患者出现心悸不安和心神不宁等症状,治疗的主要原则是以调血补气、调阴和阳为主[7]。此临床试验中给予研究组冠心病心律失常患者服用加味炙甘草汤联合美托洛尔治疗。炙甘草汤含炙甘草、生姜、麦门冬、麻仁、人参、阿胶、大枣、生地黄 9 味中药,张仲景在《伤寒论》中提到炙甘草具有健脾补气,复脉益心的功效;生地黄峻补真阴,补养充足;阿胶、麦冬、胡麻仁养阴,补血护心;人参、大枣益气健脾;桂枝配生姜以振奋心阳,温通血脉。上述药合用,有益气滋阴、通阳复脉的功效[8-9]。现代研究亦证实炙甘草汤的甘草酸、人参总皂苷和麦冬总皂苷,可以延缓 Na +内流,通过降低心肌细胞的自律性从而延长其不应期,防止心律失常的发生[10]。作为Ⅱ类抗心律失常的常用药物,美托洛尔可明显改善心肌细胞血供,降低心肌耗氧量,从而达到有效抗心律失常的良好效果。因此,加味炙甘草汤联合美托洛尔治疗冠心病心律失常的疗效显著。

该研究中,对照组在基础治疗外给予琥珀酸美托洛尔缓释片,研究组在对照组的基础上联合加味炙甘草汤进行治疗。结果显示:治疗后研究组室性、房性以及房室交界期前收缩指标分别为(616±40)次/24 h、(279±11)次/24 h、(109±5)次/24 h,研究组各项收缩指标均显著低于对照组,差异有统计学意义(P<0.05);研究组的中医证候积分主证、次证分别为(2.23±0.43)分、(3.03±0.77)分,研究组的中医证候积分(主证与次证)均明显低于对照组,差异有统计学意义(P<0.05)。以上数据可与张继磊[3]的研究结果对照,结果示:治疗后研究组室性、房性以及房室交界期前收缩指标分别为 (641.43±85.19) 次/24 h、(286.56±15.22)次/24 h、(104.16±15.28)次/24 h,研究组的上述指标均明显低于对照组,差异有统计学意义(P<0.05),研究组的中医证候积分主证、次证分别为(2.03±0.45)分、(2.27±0.27)分,研究组的中医证候积分(主证与次证)均明显低于对照组,差异有统计学意义(P<0.05)。这与炙甘草汤中有效成分具有健脾补气、复脉益心、补血护心、益气滋阴、通阳复脉等功效有关。同时炙甘草汤中甘草酸、人参总皂苷及麦冬总皂苷等合用时可有效缩短动作电位时程、50%及 90%复极化时间,心房肌兴奋性及自律性得到有效降低,防治心律失常的发生,其在治疗心律失常疗效独特,具有双向调节作用,并且不会对患者产生心律失常的副作用,疗效确切[9]。这与王兆博等[7]的研究结果保持一致。

综上所述,加味炙甘草汤联合美托洛尔治疗冠心病合并心律失常效果确切,可显著提高患者治疗有效率,有效改善患者心律失常的变化情况、且不会增加不良反应发生的风险,安全可靠。但该研究研究仍存在选择患者数少等问题,故对于炙甘草汤的临床应用及研究仍需进一步探究及思考,使炙甘草汤发挥更大的临床疗效。

参考文献(References)

[1] 吴连.探讨分析老年冠心病心律失常护理方法以及效果[J].临床医药文献电子杂志,2017,4(35):6828-6829.

[2] 赵资源,周晓燕,陈伟亮.稳心颗粒联合美托洛尔治疗冠心病合并心律失常疗效观察[J].实用中医药杂志,2018,34(1):84-85.

[3] 张继磊. 炙甘草汤联合美托洛尔治疗冠心病心律失常的疗效观察[J].实用中西医结合临床,2018,18(9):17-19.

[4] 王国菊,陈运海,毛丽娟,等.稳心颗粒联合美托洛尔治疗老年人冠心病心律失常的疗效及其安全性分析[J].中国生化

(下转第 150 页)

3 讨论

精神分裂症患者大多为慢性起病，患者的临床症状主要表现为生活态度懒散、性格改变、敏感多疑等，而对于部分起病较急的患者，则有可能出现突然兴奋、片段幻觉、行为紊乱等情况。精神分裂症的出现不仅影响了患者的日常生活与工作，更严重危及其身心健康，故而积极采取相应的临床治疗措施其重要性与必要性不言而喻。有研究学者指出，精神分裂症患者治疗预后情况与其自身的认知功能障碍恢复情况之间息息相关，若患者自身认知功能较为良好，那么其疾病预后、社会功能康复的能力也就更高，因此，治疗精神分裂症时，也常从这一角度切入[4-5]。近年来，非典型抗精神病类药物不断涌现，为患者选择更为高效、科学的治疗方案也成为广大医学界学习们不断探究的热点课题。

在该研究中，以60例首发精神分裂症患者而为研究对象，将其分成对照组与观察组后，分别实施利培酮单独治疗与利培酮、奥氮平联合治疗，其结果显示，联合用药更有助于缓解患者的精神病理症状、提升其认知功能。利培酮与奥氮平均为非典型抗精神病药，其中，利培酮可有效拮抗多巴胺能、IIR上腺素能、五轻色胺，有助于病情控制，同时，患者口服利培酮后，其药效能够快速于患者机体中发挥[6]。奥氮平则是通过作用5-羟色胺、多巴胺D等受体而选择性抑制患者中脑边缘系统多巴胺神经元放电，此时，纹路运动功能损伤减少，患者认知功能得以显著改善。而将上述两种药物联合运用，能够有效发挥而知之间的协同作用，药效大增，应用效果更突出。此外，从不良反应上看，观察组药物不良反应发生率明显低于对照组（3.33%<20.00%）（$P<0.05$），该结果则提示，联合运用奥氮平是能够有效降低患者的延误反应。与朱军等人[7]研究结果相似，即对照组的不良反应发生率为10%，对照组为3.3%，差异有统计学意义($P<0.05$)。这是由于，单独运用利培酮有可能导致患者出现静坐不能等锥体外系反应，进而影响其运动功能与运动协调性。但在联合使用奥氮平药物后，5~8 h即可达到血浆峰值浓度，此时药物则可通过与氧化反应结合而从肝脏中代谢，其循环代谢不会穿过血脑屏障也有效减少了药物不良反应，并避免加重患者认知功能障碍[8]。

综上所述，可得出该研究结论：临床治疗首发精神分裂症患者时联合运用奥氮平与利培酮，不仅能够有效改善患者认知功能、缓解其精神病理症状，也可以有效降低药物不良反应发生概率，进一步提升治疗效果。

参考文献(References)

[1] 张永. 奥氮平联合利培酮治疗难治性精神分裂症的疗效分析[J].当代医药论丛,2019,17(5):134-135.

[2] 郑元欣. 奥氮平与利培酮治疗首发精神分裂症病人的临床研究[J].黑龙江医药,2019,32(1):129-131.

[3] 莫小俊. 氯氮平联合利培酮治疗首发精神分裂症的临床效果观察[J].临床合理用药杂志,2019,12(5):58-59.

[4] 徐莉.利培酮、奥氮平治疗首发精神分裂症患者的随机对照研究[J].中外医疗,2018,37(35):87-89.

[5] 季爱民. 用利培酮与奥氮平对首发精神分裂症患者进行治疗的效果与安全性分析[J].当代医药论丛,2018,16(23):120-121.

[6] 孙福刚,陈雪莲,周海文.利培酮联合奥氮平治疗精神分裂症的疗效及对患者认知功能的影响[J].中国医院用药评价与分析,2018,18(11):1460-1461,1464.

[7] 朱军,谢群,陆元良,等.奥氮平联合利培酮治疗难治性精神分裂症的应用疗效及安全性观察[J].临床医药文献电子杂志,2018,5(76):27-28.

[8] 刘明秋,黄汉军.分析对比奥氮平与利培酮治疗老年精神分裂症的疗效及安全性[J].中国医院药学杂志,2018,38(15):87-90.

（上接第147页）

药物杂志,2017,37(6):325-327.

[5] 金莲子,侯平.中医药治疗冠心病合并缓慢性心律失常的研究进展[J].内蒙古中医药,2017,36(9):152-154.

[6] 王贵奇. 琥珀酸美托洛尔缓释片联合稳心颗粒治疗冠心病心律失常的临床效果观察[J].临床医学研究与实践,2017,2(4):41-42.

[7] 王兆博,沈晓旭,杨涛,等.炙甘草汤对比西药抗心律失常药物治疗室性期前收缩临床随机对照试验的Meta分析[J].中国中医急症,2018,27(4):576-581.

[8] 王思文.炙甘草汤的临床应用及实验研究[J].吉林中医药,2018,38(1):96-98.

[9] 高丹,黄兴,王哲,等.炙甘草汤治疗心律失常研究进展[J].河北中医,2017,39(8):1258-1262.

(P<0.05),由此可见,夏枯草汤联合厄贝沙坦对血脂也有良好的调节作用。观察组不良反应发生率显著低于对照组(P<0.05),说明夏枯草汤在改善厄贝沙坦的不良反应发生率方面也具有一定作用。

综上所述,夏枯草汤与厄贝沙坦联合治疗原发性高血压患者,可显著降低血脂水平,提升治疗效果,且不良反应发生较少,可在临床上推广应用。

参考文献

[1] 郭杰,余灿清,吕筠,等.中国10个地区人群高血压患病率、知晓率、治疗率和控制情况分析[J].中华高血压杂志,2016,24(4):469-474.

[2] 中国高血压防治指南修订委员会.中国高血压防治指南(2010年修订版)[J].中国实用乡村医生杂志,2012,19(12):1-15.

[3] 《中国高血压基层管理指南》修订委员会.中国高血压基层管理指南(2014年修订版)[J].中华高血压杂志,2015,23(1):24-44.

[4] 牛瑞虹,李丰,姜静.老年高血压病诊断与治疗研究进展[J].人民军医,2017,60(5):507-511.

[5] 赵鹏杰,赵旭.中西医结合治疗老年高血压病的临床研究进展[J].中国药物与临床,2016,16(2):215-216.

[6] 朱芹英,任永学,黄学莲.夏枯草汤治疗老年高血压的疗效及对ET-1的影响[J].中药材,2016,39(3):669-671.

(收稿日期:2019-04-08)

美托洛尔联合炙甘草汤加减治疗气阴两虚型冠心病心律失常的效果观察

万志敏　万美萍*

(潜江市中医院,湖北 潜江 433100)

【摘要】目的　分析美托洛尔联合炙甘草汤加减治疗气阴两虚型冠心病心律失常的临床效果。**方法**　选择我院2016年12月—2018年12月收治的冠心病心律失常(气阴两虚型)患者92例,以随机数字表法将其分为对照组(46例,美托洛尔)和研究组(46例,在对照组基础上联合炙甘草汤)。比较2组患者的临床疗效。**结果**　研究组临床总有效率显著高于对照组(P<0.05)。**结论**　美托洛尔与炙甘草汤联合治疗,可有效改善冠心病心律失常(气阴两虚型)患者的病情,值得借鉴。

【关键词】冠心病　心律失常　气阴两虚型　美托洛尔　炙甘草汤　疗效

DOI:10.19435/j.1672-1721.2019.19.072

冠心病心律失常在临床中极为常见,老年人的发病率相对较高,主要是由于心脏起搏及传导系统异常所致,轻者生活质量明显降低,重者极易猝死。美托洛尔是目前临床治疗该病常用的西药,仅可暂时缓解病情,治疗效果不尽如人意。临床有研究显示:炙甘草汤在治疗冠心病心律失常中疗效显著,可缩短患者的疗程,避免长期服用药物而引发多种毒副反应[1]。本研究选择我院收治的冠心病心律失常(气阴两虚型)患者92例为观察对象,分析了美托洛尔联合炙甘草汤治疗的临床效果,现报道如下。

1　资料与方法

1.1　一般资料　选择我院2016年12月—2018年12月收治的冠心病心律失常(气阴两虚型)患者92例,以随机数字表法将其分为对照组(n=46)、研究组(n=46)。研究组男26例,女20例,年龄42岁~80岁,平均年龄(61.56±5.25)岁;病程3个月~29个月,平均(16.52±6.26)个月。对照组男25例,女21例,年龄43岁~79岁,平均年龄(61.63±5.14)岁;病程4个月~28个月,平均(16.46±6.21)个月。2组基线资料差异不明显(P>0.05),可比较。

1.2　方法　对照组:予以美托洛尔缓释片(国药准字J20150044;生产企业:阿斯利康制药有限公司),口服,每次23.75~47.5 mg,每日1次,连续用药4周。

研究组:美托洛尔用法用量与对照组一致,予以炙甘草汤基本方(药物剂量随证加减):大枣10枚、火麻仁10 g、麦门冬10 g、阿胶(烊化)10 g、桂枝10 g、生地黄50 g、人参10 g、生姜10 g、炙甘草15 g。失眠者:加合欢皮10 g、酸枣仁12 g;肝肾阴虚者:加龟板10 g、鳖甲15 g;心阳不振者:加附子10 g、生牡蛎15 g;心虚者:加柏子仁10 g、茯苓15 g;心血瘀阻者:加丹参15 g、红花10 g、川芎6 g;水肿少尿者:加茯苓10 g、葶苈子15 g。每日1剂,水煎服,分2次服用,连续用药4周。

1.3　观察指标　对比2组临床疗效,其具体评价标准:心律失常发作次数减少90%,头晕、胸闷、心悸等症状消失为显效;心律失常发作次数减少50%~89%,头晕、胸闷、心悸等症状显著好转为有效;心律失常发作次数、头晕、胸闷、心悸等症状变化不明显,甚加重为无效。总有效率=(显效+有效)/总例数×100%[2]。

1.4　统计学方法　用SPSS25.0统计学软件处理数据,计数资料采用χ^2检验,P<0.05为差异有统计学意义。

2　结果

研究组临床总有效率显著高于对照组(P<0.05)。见表1。

表1　2组治疗效果对比　例(%)

组别	例数	显效	有效	无效	总有效
研究组	46	18(39.13)	26(56.52)	2(4.35)	44(95.65)
对照组	46	12(26.09)	22(47.83)	12(26.09)	34(73.91)
χ^2					8.791
P					0.003

3　讨论

*通讯作者:万美萍

作者简介:万志敏,男,本科,主治医师。

万方数据

美托洛尔属于 β_1 受体阻滞剂，可有效减少钠离子、钙离子内流，抑制钾离子外流，抑制心律失常；但是如果长期使用美托洛尔，极易引发新的心律失常，且毒副作用较大，具有一定的局限性。冠心病心律失常属于中医"胸痹"、"心悸"等范畴，中医认为该病多由血虚、阴虚、气虚导致心神不安，心是病位所在，与肺、肾、脾、肝等脏器有着极为密切的联系，中医治疗该病主要以"益气、活血、通络"为主。

本研究结果显示，研究组临床总有效率显著高于对照组（$P<0.05$），与孙国香[3]研究结果一致，说明炙甘草汤加减与美托洛尔联合在冠心病心律失常（气阴两虚型）治疗中的有效性较高。分析原因如下：炙甘草汤中甘草为君药，具有通经利血、和中缓急功效；人参、大枣、火麻仁、麦门冬、生地黄、阿胶为臣药，具有舒筋凉血、滋阴养血、补脾养心功效；生姜以及桂枝具有舒经通络、温阳通脉功效，主要起配伍、协同作用，滋而不腻、温而不燥，可发挥显著通阳复脉、益气滋阴功效。对于失眠者，加酸枣仁以及合欢皮，可起到安神解郁、养肝宁心功效；对于心阳不振者：予以生牡蛎、附子，可起到回阳救逆、潜阳补阴的功效；心虚者：加柏子仁以及茯苓，具有养心安神、健脾宁心功效；肝肾阴虚者：加龟板以及鳖甲，具有滋阴潜阳功效。现代医学认为炙甘草汤可有效清除自由基，纠正血管内分泌紊乱状态，调节血脂，且性味温和，不会给机体带来明显的毒副反应，患者耐受性良好。

综上所述，对冠心病心律失常（气阴两虚型）患者采用炙甘草汤加减与美托洛尔联合治疗，可有效降低心律失常发作次数，缓解病情，极大地改善预后，值得临床推广。

参考文献

[1] 陈晓琳.中西医结合治疗冠心病心律失常的疗效观察[J].中国现代药物应用，2016，10（9）：158-159.

[2] 李小勇. 炙甘草汤治疗老年气阴两虚型冠心病心律失常的效果观察[J].临床合理用药杂志，2018，11（5）：23-24.

[3] 孙国香. 炙甘草汤联合美托洛尔缓释片治疗冠心病心律失常 40 例[J].浙江中医杂志，2016，51（2）：138.

（收稿日期：2019-04-18）

补中益气汤加减治疗便秘型肠易激综合征的效果评价

周　兵

（湖北省中医院，湖北 武汉 430000）

【摘要】目的 研究补中益气汤加减治疗便秘型肠易激综合征的效果。**方法** 以我院收治的便秘型肠易激综合征患者 72 例为研究对象，随机分为 2 组。36 例对照组患者接受常规药物治疗，36 例观察组在常规治疗基础上联合补中益气汤加减治疗，比较 2 组治疗效果以及症状评分情况。**结果** 观察组治疗后的总有效率显著高于对照组，观察组的各项症状评分低于对照组，差异均显著（$P<0.05$）。**结论** 针对便秘型肠易激综合征患者采用补中益气汤加减治疗临床症状改善效果极佳，疗效较好，值得临床应用。

【关键词】 肠易激综合征　便秘型　补中益气汤加减　效果

DOI：10.19435/j.1672-1721.2019.19.073

肠易激综合征是一种肠道功能紊乱性疾病，多发生于青年女性，有些患者伴有家族异常因素；肠易激综合征在临床中按照大便的性状分为腹泻型、便秘型、混合型和不定型四种类型，其中便秘型的发病率最高，病程时间长，严重影响患者的生活质量及身体健康。目前临床上对于此病的治疗没有特效方法，主要为控制疾病恶化，缓解临床症状[1]。本研究观察了中药补中益气汤加减的临床治疗效果，报道如下。

1　资料与方法

1.1　一般资料　以我院 2017 年 3 月—2019 年 1 月收治的 72 例便秘型肠易激综合征患者为研究对象，采用随机分配的方法分为观察组和对照组。对照组患者 36 例，男 12 例，女 24 例，年龄 21 岁 ~48 岁，平均年龄（35.79 ± 5.36）岁，病程 1 年 ~6 年，平均（3.67 ± 0.88）年；观察组 36 例，男 13 例，女 23 例，年龄 22 岁 ~49 岁，平均年龄（36.55 ± 5.78）岁，病程 1 年 ~7 年，平均（4.01 ± 0.97）年。2 组一般资料差异无统计学意义（$P>0.05$），具有可比性。

1.2　治疗方法　对照组患者接受常规药物治疗，即：西沙比利（口服每日 3 次，1 次 5~10 mg）、谷维素（1 次服用 2 片，1 日 3 次）等药物对症治疗，连用 2 个月。观察组患者在对照组的治疗基础上采用补中益气汤加减治疗，基础方：黄芪、党参各 20 g，当归、陈皮、甘草、柴胡各 10 g，白术、人参、芍药各 12 g，另加生姜 3 片、大枣 6 枚。对于手脚冰凉加附子 10 g；便秘严重者加何首乌、槟榔各 15 g，根据患者的具体症状实施加减治疗，用水煎 2次，每日 1 剂，早晚温服。连续服用 2 个月[2,3]。

1.3　观察指标　比较 2 组患者的治疗效果及症状评分情况。疗效评价标准：显效：疾病症状完全消失，大便形态正常；有效：偶尔出现腹胀、腹泻症状，其他基本良好；无效：大便形态无改善，症状未减轻。症状评分：分别对腹胀、便秘、腹痛进行评分，分值为 10 分，评分越低患者的症状越轻。

1.4　统计学方法　以 SPSS21.0 统计学软件分析数据，计量资料以 $\bar{x} \pm s$ 表示，采用 t 检验，计数资料采用 χ^2 检验，$P<0.05$ 为差异有统计学意义。

作者简介：周兵，男，硕士，主治医师。

万方数据

炙甘草汤联合美托洛尔治疗冠状动脉粥样硬化性心脏病心律失常 45 例

王纪岗

（嵩县人民医院，河南 嵩县　471400）

摘要：目的：观察炙甘草汤联合美托洛尔治疗冠状动脉粥样硬化性心脏病心律失常的临床疗效。方法：将 90 例冠状动脉粥样硬化性心脏病心律失常患者随机分为观察组和对照组，每组各 45 例。对照组单用美托洛尔治疗，观察组在对照组的治疗基础上加用炙甘草汤治疗，两组疗程均为 4 周。结果：观察组总有效率为 91.11%，对照组总有效率为 82.22%，两组比较差异无统计学意义（$P>0.05$）；两组三种心律失常 24 h 内发生频数改善情况比较，差异具有统计学意义（$P<0.05$）。结论：炙甘草汤联合美托洛尔治疗冠心病心律失常疗效明显。

关键词：炙甘草汤；心律失常；美托洛尔；张仲景
本文引用：王纪岗. 炙甘草汤联合美托洛尔治疗冠状动脉粥样硬化性心脏病心律失常 45 例[J]. 河南中医，2014，34(3)：418－419.
中图分类号：R541.7　**文献标志码**：B　**文章编号**：1003－5028(2014)03－0418－02

冠状动脉粥样硬化性心脏病（简称冠心病）心律失常是常见的心血管类疾病，是冠心病的常见并发症之一，是因心脏传导系统及起搏发生病变，造成的病理性的异常起搏，从而引起一系列的临床症状[1]。大量临床资料表明，西药可在短时间内纠正心律失常，但副作用较多。炙甘草汤为“伤寒脉结代，心动悸”而设[2]，本研究将 90 例冠心病心律失常患者按照住院单双号随机分为观察组和对照组，探讨冠心病心律失常的中西医疗法新途径，现将结果报道如下。

1　资料与方法

1.1　一般资料　在嵩县人民医院 2009 年 5 月至 2012 年 5 月收治的冠心病心律失常患者中选择符合诊断标准者 90 例作为观察对象，按照住院号单、双号将患者随机分为观察组和对照组，每组各 45 例。其中男 47 例，女 43 例；年龄 42～73（55±5.3）岁；病程 7～18（11.2±2.4）个月；房性早搏 31 例，室性早搏 34 例，房室交界性早搏 25 例。两组在年龄、性别、发作性质、心律失常的类型、病程等方面的差异无显著性（$P>0.05$）。中医诊断标准治疗参考《中药新药临床研究指导原则》[3]：证候以心动悸、胸闷动则悸发、虚数为主，辨证属气阴两虚证。西医诊断标准符合《缺血性心脏病的命名和诊断标准》（国际心脏病学会制定），患者有非阵发性室性心动过速或者室性期前收缩。排除标准：重度或急性心力衰竭、低血压、心动过缓、Ⅱ度或Ⅲ度房室传导阻滞严重、心源性休克、心肝肾功能不全。

1.2　治疗方法　全部患者除常规需用的降压药物、硝酸酯类药物、钙离子拮抗剂、ACEI、抗凝药物以外，停用其他抗心律失常药。对照组在常规治疗的基础上服用美托洛尔（阿斯利康制药）每次 25 mg，每天 2 次；观察组在对照组的基础上加服炙甘草汤，方药组成：炙甘草 12 g，人参 6 g，麦冬 10 g，桂枝 9 g，阿胶（炖化）6 g，生地黄 30 g，生姜 9 g，麻子仁 10 g，大枣 5 枚，水煎服，日 1 剂，早晚分服。4 周为 1 个疗程。

1.3　观察指标　服药前后查血尿常规、血液生化指标及肝肾功能，行 ECG 或 Holter 检查，记录血压、心率及药物不良反应。

1.4　疗效判定标准[2]　结合患者的症状和 ECG、Holter 等检查结果进行判定。显效：主要症状消失，如气短、心悸、乏力等，发作次数减少≥90%，Holter、ECG 检查基本正常；有效：主要症状（同上）减轻，发作次数 50%～90%，Holter、ECG 检查结果有明显改善；无效：主要症状加重或者没有改善，发作次数减少≤50%，Holter、ECG 等检查结果无变化或加重。总有效率＝（显效＋有效）/n×100%。

1.5　统计学分析　采用 SPSS 16.0 统计软件进行统计学处理，计量资料用均数±标准差（$\bar{x}\pm s$）表示，计量资料采用 t 检验，计数资料采用 χ^2 检验，以 $P<0.05$ 为差异有统计学意义。

2　结果

2.1　两组疗效比较　见表 1。

表 1　两组疗效比较　例

组别	n	显效	有效	无效	总有效率（/%）
对照组	45	17	20	8	82.22
观察组	45	32	9	4	91.11*

注：与对照组比较，$^*P>0.05$。

2.2　两组心律失常改善情况比较　见表 2。

收稿日期：2013－09－16
作者简介：王纪岗（1969－），男，河南洛阳人，医学学士，副主任医师。

表2 两组心律失常改善情况比较 ($\bar{x} \pm s$,次·24 h^{-1})

组别	n	室性早搏		房性早搏		房室交界早搏	
		疗前	疗后	疗前	疗后	疗前	疗后
对照组	45	1 869 ±154	986 ±105	1 007 ±90	521 ±43	258 ±28	167 ±19
观察组	45	1 874 ±168	653 ±65	1 010 ±99	298 ±32	267 ±34	105 ±10
t值		-0.147	13.364	-0.110	27.909	-1.371	19.371
P值		0.883	0.000	0.913	0.000	0.174	0.000

3 讨论

炙甘草汤又名“复脉汤”,是医圣张仲景著作《伤寒论》中的经典名方,有滋阴补血,益气复脉的功效,是治疗冠心病心律失常的常用方[4]。

方中重用炙甘草补中益气,缓急养心为君,《名医别录》谓其“通经脉,利气血”,为治心动悸,脉结代之要药,为复脉之根本;人参大补元气,主安五脏;大枣味甘益脾养心,人参、大枣、甘草合用,补益后天脾胃之气,脾胃健旺,则血气化源充足,可固脉之本源。生地黄滋阴养血,大剂使用,可复心阴;阿胶滋阴补血润燥,能“主心腹内崩劳极”;麦冬清心润肺益胃;麻子仁滋阴养液润燥。四药合用,滋阴养血之功颇著,能充脉之实体。桂枝配生姜有温通血脉、振奋心阳作用。诸药联用,可通阳复脉、滋阴益气。研究表明[4],炙甘草汤可明显拮抗心肌细胞缺血缺氧导致的APD50、APD90和APD的缩短,使VDD和RPF逐渐加快,可以基本恢复心脏正常的节律,有明显抗心律失常的作用。现代研究表明[5],炙甘草汤正性肌力和抗早搏的作用明显,可以提高心肌的耐缺氧能力,并且能增加心脏冠状动脉血流量。而美托洛尔属于第Ⅱ类抗心律失常药,可以提高室颤的阈值,减慢异位起搏点的频率及自主窦性心率,减慢兴奋传导速度,可改善缺血区心肌细胞的血供并降低心肌的耗氧量,从而起到显著抗心律失常的作用[6]。本项研究观察组总有效率91.11%,高于对照组的82.22%,但差异无统计学意义($P>0.05$);两组三种心律失常24 h内发生频数改善情况差异具有统计学意义($P<0.05$)。与陈思法报道[7]炙甘草汤合血府逐瘀汤治疗冠心病室性早搏可有效改善冠状动脉供血,抗心律失常作用明显,无明显不良反应,基本一致。牛广团等[8]在西医常规治疗的基础上加用炙甘草汤加减治疗室性早搏40例,取得较好疗效,有报道[9]表明炙甘草汤加减治疗窦性心动过缓疗效显著。

综上所述,采用炙甘草汤联合美托洛尔的中西医结合疗法治疗冠心病心律失常,疗效明显,能有效改善患者的临床症状,具有益气养血滋阴复脉之功效[10],降低心律失常的发作率且副作用较少,有一定的临床借鉴意义。

参考文献:

[1] 蒋丽霞,滕兴平.胺碘酮联合稳心颗粒治疗冠心病心律失常90例[J].辽宁中医杂志,2011,38(9):1844.

[2] 丁德正.炙甘草汤在精神疾病中的运用[J].河南中医,2010,30(4):325-327.

[3] 郑筱萸.中药新药临床研究指导原则[M].北京:中国医药科技出版社,2002:68-69.

[4] 张晓云,胥爱文,马建伟,等.炙甘草汤对缺血缺氧诱发豚鼠心律失常的电生理效应[J].中国新药与临床药理,2008,19(2),103-104.

[5] 王宏利.炙甘草汤治疗冠心病心律失常48例[J].光明中医,2009,24(12):2275.

[6] 卢林帆.稳心颗粒联合美托洛尔治疗冠心病心律失常疗效分析[J].中国医药指南,2011,9(12):34-35.

[7] 陈思法.炙甘草汤合血府逐瘀汤治疗冠心病室性早搏疗效观察[J].河南中医,2012,32(6):765-766.

[8] 牛广团,牛莺歌,陈柏林.炙甘草汤加减治疗室性早搏40例[J].河南中医,2007,27(10):8-9.

[9] 黄彦德.炙甘草汤加减治疗窦性心动过缓37例[J].河南中医,2009,29(10):959.

[10] 王利民,宋桂叶.炙甘草汤加减治疗病毒性心肌炎临床研究[J].河南中医学院学报,2008,23(3):35-36.

(编辑:焦凡)

郑 重 声 明

本刊已被《中国期刊全文数据库—CJFD》、《中国核心期刊(遴选)数据库》、《中国学术期刊(光盘版)》、《中国生物医学文献数据库—CBMdisc》、《中国学术期刊综合评价数据库》、《中国药学文献数据库》、《中文科技期刊数据库》等数据库收录并上网,如有作者不同意其论文被以上数据库收录,请提前告知,本刊将作特殊处理。

特此声明。

《河南中医》编辑部
